# Global health worker visit time variation: A systematic review

**DOI:** 10.1016/j.hpopen.2026.100169

**Published:** 2026-04-06

**Authors:** Peter Murphy, Wajeeha Raza, Filip Meheus, Altea Sitruk, Kratu Goel

**Affiliations:** a Centre for Health Economics, University of York, York, United Kingdom; b Department of Health Financing and Economics, World Health Organization, Geneva, Switzerland

**Keywords:** Systematic review, Meta-regression, Consultation, Visit, Duration

## Abstract

**Background:**

The accurate estimation of the time individuals spend in a health consultation can form a crucial input to measuring diagnostic quality, health workforce planning and costing of services globally. To address this, a systematic review was conducted to identify the length of health care visit times accounting for a range of potential modifiers such as the type of healthcare worker, service delivery platform, and clinical decision-making complexity.

**Methods:**

The search for visit times was conducted across seven databases and included approaches to capture the relevant grey literature. Data were extracted, quality appraised, and synthesised using *meta*-analysis. Potential sources of heterogeneity were explored using univariate subgroup analysis and *meta*-regression.

**Findings:**

The search identified 422 studies for inclusion. The average visit time excluding procedures was 19.2 min (95% CI: 16.7–21.6 min) and showed variation across country income classification, region, service delivery platform, health worker and the use of telemedicine. The *meta*-regression accounted for a degree of the residual heterogeneity compared to the univariate analysis. The models demonstrated statistically significant variation in the visit times based on factors such as the country income classification, telemedicine, and, in certain models, the service delivery platform.

**Interpretation:**

The estimated variation in visit times can help form part of the evidence base to appraise global health systems, inform health workforce planning, and for costing of services globally. Despite the identified evidence, more research is required to fill the considerable evidence gaps of visit times in lower-income countries as 80% of the studies informing the results come from high income and upper-middle income countries.

## Highlights

• Global variation in visit time across health workers and setting requires research.
• Our systematic review of global visit times identified 422 studies.
• Variation was found by country, health worker and setting.
• Vast majority of the evidence is from high income countries.

## 1 Introduction

The time a patient spends in a visit or consultation with a health worker (referred to hereafter as ‘visit time’) provides the foundation for patient care. Ensuring visit times are of sufficient length aids diagnostic quality, improves treatment outcomes, broadens service provision and can substantially improve patient satisfaction [1], [2], [3], [4], [5]. It can serve as an important indicator of health system performance through ensuring health workers spend sufficient time with patients during visits [6]. Visit times also provide an essential input for human resource planning and the costing of services [7], [8], which are particularly important for many low- and middle-income countries given the burden of human resource expenditure on health budgets [9]. Thus, identifying the length and variation in visit times across healthcare settings remains a critical piece of evidence in the planning and provision of quality care in order to ensure global health security and equity, particularly in light of recent health emergencies, such as the COVID-19 pandemic [10].

Globally, there are substantial variations in visit time guidelines and the real-world implementation of visit times. For example, the NHS in the UK recommends a minimum of 15 min per physician visit [11], while in Egypt the recommended visit time is 30 min [12]. The literature of observed visit times shows similar variation across countries and regions. A systematic review by Irving et al. [13] reported high variation in average primary care consultations ranging from less than a minute in Bangladesh to over 20 min in Sweden. The authors reported that approximately 50% of the global population have visits of 5  min or less with their primary care physician. Studies from low-resource settings were included however the results were dominated by those conducted in high-resource settings. The literature includes examples of studies assessing visit times in high-resource settings [4], [14], [15] but examples of those covering low-resource are also available. Research by Kruk et al. [16] observed variation in sick child consultations revealing visit times in Tanzania are double that of neighbouring Uganda. A review of the reproductive care services across five countries found that the total health worker time for the first antenatal care visit varied from 15 min per patient in Papua New Guinea to 60 min per patient in Bangladesh [17]. Such global variation in visit time risks limiting thorough assessment and widening health inequalities for many of the world’s poorest. Increased funding challenges [18], changing demographics [19] and migration of the health workforce [20] may exert pressure on health system quality and resilience and in-turn apply greater pressure on the time health workers spend with patients.

Within countries there is the potential for variation in visit times across disease areas and patient care needs. A study conducted in rural China observed differing visit times for patients with symptoms of angina and tuberculosis [5] and a systematic review by Tadeu et al. [21] identified trends in patients with multimorbidity and visit time. The literature indicates the length of time a patient spends with a provider can be related to whether the patient has been seen by the health provider before, the socioeconomic status of the patient, the geographic location of the practice, and health provider characteristics such as sex and age [22], [23], [24]. Overall, there is, however, limited evidence to demonstrate the range of variations in visit times across location, settings, health workers, and disease areas. Studies that have compared visit times typically focused on a specific health worker, occupational group, or are limited to primary care.

In this systematic review, we aim to address the gap in the literature by reviewing the evidence on visit times across countries, geographic regions, service delivery platforms, disease areas, health workers, and complexity of visit. It is hoped the results can provide descriptive benchmarking on a key organisational factor shaping clinical information gathering that has important implications for health system performance, service planning, costing and delivery, patient equity and safety, and diagnostic accuracy.

## 2 Methods

The protocol was registered with PROSPERO (CRD42023376859) and was conducted and reported in accordance with PRISMA guidelines [25]. A PRISMA checklist has been completed and reported in appendix A3.

### 2.1 Data sources and searches

The search strategy was designed in Ovid MEDLINE and included terms for visits, health personnel, and time factors. No language limits were applied but papers were limited to 2002 onwards to ensure relevance to current research and policy deliberations. Details of the full search strategies are contained in appendix A1. The following databases were searched on the 30/10/2022: MEDLINE® ALL (Ovid); Science Citation Index Expanded (Web of Science); CINAHL (EBSCOhost); Scopus; Cochrane Database of Systematic Reviews; Cochrane Central Register of Controlled Trials; EconLit (OVID).

Supplementary searches through reference checking and backwards citation searching of the systematic reviews identified in the primary database search were undertaken on 15/01/2023. The searches were updated on 23/07/2024.

To help identify relevant sources of grey literature, the authors searched for multi-country resource-use/costing studies through expert consultation. Email communication was sent to experts known to the authors with experience in multi-country resource and costing datasets. Any identified study was scrutinised by the review team for an explicit statement of measured visit time informing resource use/cost estimates.

### 2.2 Study selection

Two review authors (PM and WR) independently conducted title and abstract screening of a random sample of 10% of the retrieved records. The percentage agreement and a kappa statistic for assessing inter-rater agreement [26] were calculated. Any discrepancies were resolved by discussion among the two review authors and a third review author (AS). As agreement across the two reviewers was high (95% agreement and a kappa statistic of 0.76), one reviewer (PM) screened the remainder of the records.

Records were included if they reported a visit or consultation duration defined as time spent between health professional or allied health professional and a patient. Studies were limited to those in which it was clear that the reported visit time was time spent with a patient. Non-English language studies were excluded if an English language version of the study was not readily available. Randomised controlled trials and qualitative studies were excluded. The method deviated from the protocol at the full text review stage to limit the studies to those published from 2017 onwards due to the volume of identified studies and based on the assumption that more recent studies were more likely to report visit times that reflect current practices, guidelines and technological advances.

### 2.3 Data extraction and critical appraisal

The extracted information was based on central characteristics of the included studies: study design, the number of observations (i.e. patient visits), population, and country. The characteristics of the visit were extracted and included the service delivery platform, health worker categorisation, type of visit, visit and details. The disease area was extracted and categorised based on the UHC Compendium list of health programmes [27]. Data were also extracted on whether the visit was a telemedicine visit and if the visits occurred during the COVID-19 pandemic. Finally, the visit times including subgroup results and corresponding variability or uncertainty were extracted. The pro-forma can be found in appendix A2.

As the included studies were expected to be cross-sectional studies, critical appraisal was based on the Appraisal tool for Cross-Sectional Studies (AXIS) tool [28]. Studies were appraised by one reviewer (PM). Critical appraisal was undertaken to inform interpretation of the findings and to allow readers to assess the strengths of the included studies. Inclusion was not contingent on the critical appraisal rating.

### 2.4 Data synthesis

The mean visit time in minutes is used throughout the synthesis. Studies that reported a mean and no measure of the variability or uncertainty were not included in the synthesis, but the visit time was extracted. For visit times reported in the form of a grouped frequency table, the midpoint of each group was multiplied by the frequency to estimate a mean.

The synthesis occurred in two stages. The first stage was a random effects *meta*-analysis using the reported standard error; reported standard deviations and 95% CIs were converted to standard errors [29]. The *meta*-analysis was conducted in Stata, Version 18 [30]. Heterogeneity was assessed through the inspection of forest plots, the tau^2^, and I^2^ statistics [31], [32]. Upon detection of considerable heterogeneity (75% or above using I^2^ statistic) [29], exploration of the sources of heterogeneity was conducted using subgroup analysis. The subgroups were identified *a priori* as: Health worker (General practitioner; Specialist medical practitioner; Nurse; Pharmacist; Midwifery professional; Other); Service delivery platform (Community-based services; General Outpatient; First referral level; Second referral level); visit type (Initial; Follow-up); Telemedicine (In-person; Telemedicine); COVID-19 (Studies conducted during the COVID-19 pandemic, studies conducted prior to the COVID-19 pandemic^1^); World Bank country income classification (High income; Upper middle income; Lower middle income & Low income); and WHO region (Africa; Americas; Eastern Mediterranean; Europe; South-East Asia; Western Pacific). For the World Bank country income classification lower middle income and low income were combined due to the paucity of data in each group.

Multiple effect sizes from subgroup analyses were extracted from the studies and the potential multiplicity was addressed [33], [34]. For the univariate *meta*-analysis, a reductionist approach of averaging within a study was adopted which reduced the dataset to a single independent observation per study [33]. Due to a lack of the correlation structure of the summarized subgroup variables in the reported literature, the mean average of the within-study variances was estimated.

Stage two used *meta*-regression to investigate the impact of moderator variables on visit time. Meta-regression explores the impact of study-level moderator variables to explain between-study variation. It assumes independence of study estimates, observed estimates have a known sampling variance from the primary study, unexplained variance exists after inclusion of moderator variables and that a residual heterogeneity exists across studies. Like *meta*-analysis, *meta*-regression weights studies by the sample size of the underlying study. For more information see Deeks et al. [35]. The moderator variables used were the subgroups and categories identified in stage one. The multiplicity was addressed through an integrative approach using robust variance estimation methods [36]. The within-study effect-size correlation (ρ) was unknown therefore a range of ρ values were used. Regression coefficients in the *meta*-regression were termed significant if the p value was < 0.05. Analyses were conducted using complete-case data. The studies with missing moderator variables were excluded from the *meta*-regression models. No imputation of missing moderator variables was performed. Meta regression analyses were implemented in Stata [30] using the Robumeta package [37].

The funder of the study provided guidance on the protocol data collection, data analysis, data interpretation, and writing of the report.

## 3 Results

**Fig. 1:**
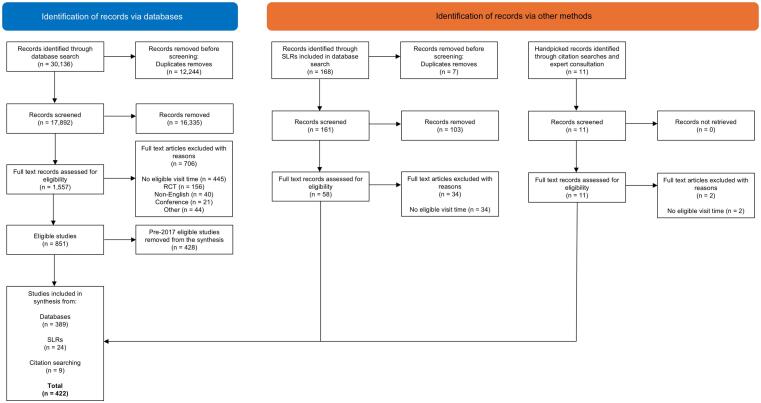
PRISMA flow diagram.

The database search retrieved 17,892 unique records following deduplication. Of these, 16,335 were excluded during title and abstract screening leaving 1,557 records for full text review. 851 met the eligibility criteria for inclusion. The updated search filter for year of publication resulted in 428 studies being removed leaving a total of 423 studies being eventually included from the database search. This included 34 systematic reviews, resulting in 389 individual studies included in the synthesis. The studies included in the identified systematic reviews were screened and resulted in an additional 24 studies. Nine studies identified through citation searching and expert consultation were also included. Multinational costing studies identified through expert consultation were limited to the Value TB [38] and the USAID PEPFAR Applying Activity-Based Costing and Management (ABC/M) to HIV Services studies [39]. Due diligence on the visit times reported in the two costing studies resulted in the studies being excluded as it was unclear if the visit time captured was health provider time spent exclusively with patients. The result is 422 studies included in the synthesis. See, Fig. 1 for the PRISMA flow diagram. A breakdown of the number of articles screened in the original and update searches can be found in appendix A1.

### 3.1 Summary of the studies

The results table presented in appendix A4 provides a summary of the studies identified in this systematic literature review. There were 81 countries^2^represented in the results. The 422 studies resulted in 1,419 visit times included in the analysis set. Visit duration was measured using a number of different methods, including recording with a video, audio, or stopwatch (32%), self-reported/questionnaire (28%), existing databases or guidelines (15%), electronic health records (11%) and software tracking (9%). For a results table of the individual studies, please see appendix A4.

### 3.2 Quality assessment

Evaluation using the AXIS tool revealed only one study [40] scored favourably across all 20 of the tool’s questions. The failure to report or address the information of non-responders resulted in many studies scoring poorly across the Methods and Results domains of the AXIS tool. The quality assessment results are reported in appendix A5.

### 3.3 Evidence map

**Fig. 2:**
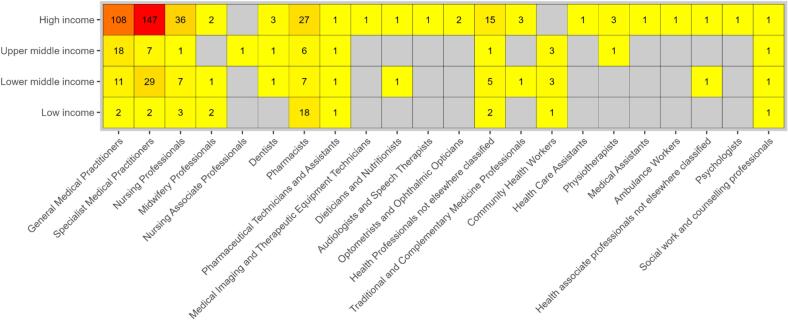
Evidence map – health worker.

The density plot in Fig. 2 indicates the gaps in the evidence on visit times. Each data point represents an individual study, or, where multiple country-specific results are provided in a single study, each country with its own result represents an individual data point. The results are reported by country, categorised by World Bank income classification [41].

The majority of the evidence identified in this study is for countries classified as high-income countries (HICs). Across health workers, the most abundant workers captured in the evidence were specialist medical practitioners (n = 185), general medical practitioners (n = 139), and nursing professionals (n = 47). Nineteen categories of health workers using the International Standard Classification of Occupations (ISCO) classification of health workforce cadres [42] were not represented in the evidence. A density plot by service delivery platform can be seen in appendix A6.

### 3.4 Univariate meta-analysis

**Fig. 3:**
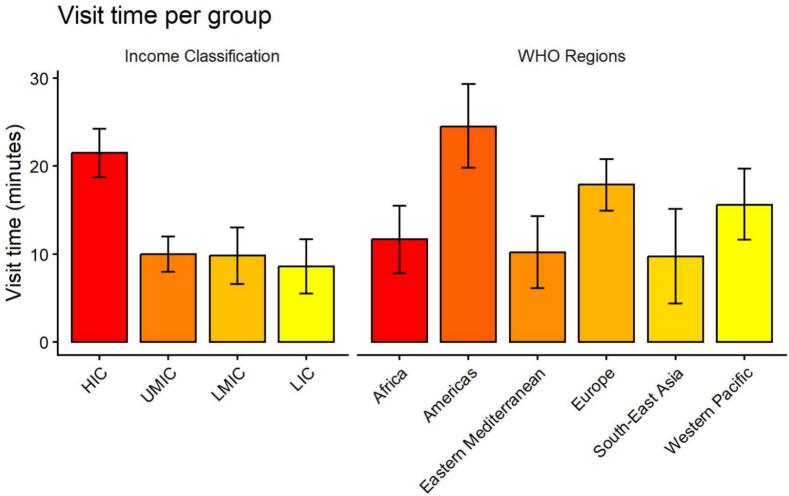
Variation in visit time across World Bank income classification and WHO region. Abbreviations: LIC, low income countries; LMIC, low-middle income countries; HIC, high income countries; UMIC, upper middle income countries; WHO, World Health Organization.

Of the 422 studies identified in the systematic review, 166 studies reported a mean visit time with uncertainty and were therefore included in the *meta*-analysis. Procedures were removed from the synthesis owing to evident heterogeneity but are reported in the summary table. The average visit time across all studies (excluding procedures) was 19.2 min (95% CI: 16.7 – 21.6). Variation was identified across income classification and WHO Region. High income countries had a visit time of 21.5 min (95% CI: 18.7 – 24.2) compared to 8.6 min (5.5 – 11.7) in LICs. Across WHO Regions, visit times ranged from 24.5 min (19.8 – 29.3) and 17.9 min (14.9 – 20.8) for the Americas and Europe, respectively. Visit times were lower for South-East Asia and Africa, estimated as 9.7 min (4.4 – 15.1) and 11.7 min (7.8 – 15.5), respectively. Fig. 3 displays the variation in visit time across World Bank Income Classification and WHO Regions. Further variation was identified across health workers, 13.4 min (11.6 – 15.2) for general practitioners and 21.3 min (14.6 – 27.9) for nurses; service delivery type, 11.9 min (5.0 – 18.8) for community-based, 15.2 min (12.9 – 17.5) for general outpatient and 23.1 min (16.0 – 30.2) for first referral; and visit type, initial was 23.8 min (17.2 – 30.3) and 15.1 min (11.2 – 18.9) for follow-up. Summary results of the *meta*-analysis including the subgroups analyses can be seen in Table 1. There was considerable evidence of heterogeneity across all analyses, I^2^ > 99%. Forest plots are reported in the appendix A7.

**Table 1:** Summary results from the univariate *meta*-analysis.

Group	n	Average (min)	Lower 95% CI (min)	Upper 95% CI (min)	Tau2	I2 (%)
All studies*	170	19.8	17.1	22.6	333	100
All studies *procedures removed	166	19.2	16.7	21.6	251	100
Procedures	6	54.1	11.1	97.2	290	99.9
**All studies with procedures removed (n = 166)**						
**WB country income classification**						
HIC	124	21.5	18.7	24.2	247	100
UMIC	20	10.0	8.0	12.0	19	99.8
LMIC	18	9.8	6.6	13.0	49	99.9
LIC	4	8.6	5.5	11.7	10	99.6
**WHO Regions**						
Africa	12	11.7	7.8	15.5	46	99.9
Americas	59	24.5	19.8	29.3	338	100
Eastern Mediterranean	12	10.2	6.1	14.3	52	99.9
Europe	49	17.9	14.9	20.8	110	100
South-East Asia	5	9.7	4.4	15.1	37	99.9
Western Pacific	31	15.6	11.6	19.7	131	99.9
**Health worker**						
General practitioner	55	13.4	11.6	15.2	46	100
Specialist physician	75	19.4	16.0	22.7	215	100
Nurse	16	21.3	14.6	27.9	184	100
Pharmacists	18	16.2	9.0	23.4	241	99.9
Midwifery professionals	3	33.5	19.5	47.5	151	99.8
Community health workers	3	11.3	4.2	18.4	39	99.6
**Service delivery platform**						
Community-based	7	11.9	5.0	18.8	84	99.9
General outpatient	66	15.2	12.9	17.5	86	100
First referral level	50	23.1	16.0	30.2	653	100
Second referral level	36	18.9	14.4	23.4	191	99.9
**Visit type**						
Initial	45	23.8	17.2	30.3	499	100
Follow-up	32	15.1	11.2	18.9	123	99.9
**Telemedicine**						
In-person	143	18.5	16.0	21.1	238	100
Telemedicine	34	13.3	10.4	16.1	70	99.9
**COVID-19**						
Non-COVID-19	150	19.2	16.4	22.0	304	100
COVID-19	21	17.0	13.1	20.9	83	99.9
**Disease**						
Child Health	12	25.3	15.8	34.7	278	99.9
Ophthalmology	7	20.7	3.4	38.0	544	99.9
Sexual and reproductive health	4	22.9	12.9	32.9	102	99.8
Surgery and anaesthetic care	19	14.8	9.0	20.7	168	99.9
Maternal and newborn health	7	16.1	13.6	18.7	12	99.4
General	34	15.1	10.9	19.2	153	100
Tuberculosis	2	6.9	4.7	9.2	2	44.2
Urology	3	14.1	6.7	21.6	43	99.3
Non-communicable disease	31	19.6	14.7	24.5	192	100
Mental health, neurologic disorders and substance use	7	19.0	12.3	25.5	77	99.9

Abbreviations: CI, confidence interval; HIC, high income country; LIC, low income country; LMIC, lower middle income country; min, minute; UMIC, upper middle income country, WB, World Bank; WHO, world health organisation.

Note, all results are reported apart from * are for the data set with procedures removed.

### 3.5 Meta-regression

The results of the *meta*-regression are presented in Table 2 and Table 3. Results are reported in the original scale i.e. in minutes. Meta regression estimations were explored: those that included the service delivery platform (models 1 to 4) and separately those that include health worker (models 5 to 8). They were not included in the same estimation due to data limitations. Many studies in the synthesis grouped the visit type (i.e., initial and follow-up) together therefore we present results exclusive of visit type due to the potential for uncaptured heterogeneity in the ‘Other’ category in appendix A8. The estimations also remove whether the study occurred during the COVID-19 pandemic, as the assumption that this may impact visit time is our hypothesis rather than being based on the literature. Data limitations did not allow the incorporation of disease in the estimation as there was a concentration of studies focussing on a few disease areas. The *meta*-regression models are described in appendix A8 and the results of all 8 are reported. The results of the meta regression were insensitive to the value of ρ, a value of 0.8 was used to generate the results.

**Table 2:** Meta regression results.

Variable	Duration (mins)	p-value	95% CI (L)	95% CI (U)	Duration (mins)	p-value	95% CI (L)	95% CI (U)
	**Service delivery platform Model 1**				**Health worker Model 1**			
Region								
HIC, UMIC	4.57	0.022	0.72	8.43	5.81	0.0033	2.09	9.53
LMIC, LIC	Reference	Reference	Reference	Reference	Reference	Reference	Reference	Reference
Service Delivery platform								
Community	−7.25	0.071	−15.24	0.75	−	−	−	−
GOP	−2.38	0.49	−9.8	5.03	−	−	−	−
1st & 2nd referral	−1.44	0.62	−7.76	4.88	−	−	−	−
Other^†^	Reference	Reference	Reference	Reference	−	−	−	−
Health worker								
GP	−	−	−	−	−2.88	0.23	−7.71	1.94
SP	−	−	−	−	−0.60	0.79	−5.07	3.88
Nurse	−	−	−	−	2.91	0.46	−5.28	11.10
Other^⁋^	−	−	−	−	Reference	Reference	Reference	Reference
Visit type								
Initial	2.94	0.12	−0.85	6.72	3.03	0.13	−1.00	7.06
Follow-up	0.7	0.65	−2.53	3.93	0.83	0.59	−2.34	4.01
Not reported^‡^	Reference	Reference	Reference	Reference	Reference	Reference	Reference	Reference
Telemedicine								
Telemedicine	−2.4	0.36	−7.72	2.92	−2.72	0.20	−6.99	1.55
In-person	Reference	Reference	Reference	Reference	Reference	Reference	Reference	Reference
Covid-19								
Covid-19	−2.99	0.21	−7.72	1.75	−3.11	0.20	−8.03	1.81
Non covid-19	Reference	Reference	Reference	Reference	Reference	Reference	Reference	Reference
Constant	12.82	0.0034	4.97	20.67	11.40	0.0010	5.00	17.79
Tau-squared	15.61	−	−	−	23.73	−	−	−
n level 1	669	−	−	−	666	−	−	−
n level 2	167	−	−	−	165	−	−	−

Abbreviations: GOP, general outpatient; GP, general practitioner; LIC, low income country; LMIC, lower-middle income country; HIC, high income country; SP, specialist physician; UMIC, upper-middle income country.

† Refers to studies in which it was not specified or it was unclear where the visit took place; herbal and traditional medicine clinics and direct to consumer remote/telemedicine clinics.

⁋ Refers to visits involving midwives, dentists, pharmacists and those in which the categorisation was unclear.

‡ Refers to studies in which it was not specified whether the visit type was initial or follow-up and studies that combined both.

Covid-19 refers to studies that were conducted during the covid-19 pandemic. This was defined as studies occurring during March 2020-December 2021 or studies that defined their data collection as happening during the covid-19 pandemic.

**Table 3:** Meta regression results for the doctor-only visits.

Variable	Duration (mins)	p-value	95% CI (L)	95% CI (U)	Duration (mins)	p-value	95% CI (L)	95% CI (U)
	**Doctor-only model 1**				**Doctor-only model 2**			
Region								
HIC, UMIC	6.09	0.0012	2.75	9.42	9.47	0.0003	4.9	14.04
LMIC, LIC	Reference	Reference	Reference	Reference	Reference	Reference	Reference	Reference
Service delivery platform								
GOP	−3.22	0.073	−6.75	0.31	−7.39	0.0001	−10.86	−3.92
1st & 2nd referral	Reference	Reference	Reference	Reference	Reference	Reference	Reference	Reference
Visit type								
Initial	1.84	0.41	−2.69	6.38	−	−	−	−
Follow-up	−0.66	0.70	−4.17	2.86	−	−	−	−
Other^†^	Reference	Reference	Reference	Reference	−	−	−	−
Telemedicine								
Telemedicine	−4.23	0.061	−8.67	0.22	−5.03	0.037	−9.73	−0.32
In-person	Reference	Reference	Reference	Reference	Reference	Reference	Reference	Reference
Covid-19								
Covid-19	−4.58	0.14	−10.82	1.66	−	−	−	−
Non covid-19	Reference	Reference	Reference	Reference	−	−	−	−
Constant	13.34	0.0002	6.98	19.71	12.17	0.0000	8.5	15.83
Tau-squared	28.07	−	−	−	805.64	−	−	−
n level 1	562	−	−	−	568	−	−	−
n level 2	133	−	−	−	135	−	−	−

Abbreviations: GOP, general outpatient; LIC, low-income country; LMIC, lower-middle income country; HIC, high income country; UMIC, upper-middle income country.

† Refers to studies in which it was not specified whether the visit type was initial or follow-up and studies that combined both.

Covid-19 refers to studies that were conducted during the covid-19 pandemic. This was defined as studies occurring during March 2020-December 2021 or studies that defined their data collection as happening during the covid-19 pandemic.

The results showed the average number of observations per study was 4.01. The minimum was one and the maximum was 62. In all models, the visit time for the income classification was higher and statistically significant in the HIC, UMIC group compared to the LMIC, LIC group. There was no statistical significance for the visit times for visit type or telemedicine. Across health workers, the visit times for general practitioners (GP) and specialist physicians (SP) were lower than nurses and other health workers. In all cases, the *meta*-regression resulted in a considerably reduced τ^2^ compared to the univariate *meta*-analysis, suggesting the covariates are explaining heterogeneity in the data.

Owing to the abundance of data for doctor visits, *meta*-regression estimations were conducted on the doctor data alone (i.e. GP + SP). The results reveal visits with doctors are shorter in general outpatient settings such as primary care than in 1st or 2nd referral centres (e.g. district or region hospitals) (p = 0.0001) and telemedicine consultations are shorter than in-person (p = 0.037). The results are presented in Table 3.

## 4 Discussion

### 4.1 Principal findings

The observed visit times and evident variation across health worker, service delivery platform, visit type and telemedicine provide crucial inputs to health planning and decision making yet considerable evidence gaps exist with 80% of the studies informing the synthesis coming from HICs and UMICs. Nevertheless, the results are important to both high-resource and low-resource settings. For HICs and UMICs this study can directly inform workforce planning, costing of services and the evaluation of health systems. In LMICs and LICs, this systematic review may help to highlight gaps in the evidence base requiring future research. Strengthening research surveillance in low-resource settings should be a priority to ensure relevant evidence is available which can ultimately inform policy and progress towards universal health coverage globally [43].

One of the objectives of this review was to assess how visit times varied according to country and geographic region. During data extraction, the country in which each included study was conducted was recorded. However, due to the limited and inconsistent availability of data for many countries, it was not feasible to incorporate country-level findings into the *meta*-analysis. As a result, detailed results for individual studies are presented in the appendix. Those interested in evidence regarding visit times for a particular country are advised to consult the relevant results table for further information. To account for geographic variation in the synthesis, we adopted an *a priori* categorisation approach, grouping studies according to World Bank income levels and WHO Regions. This method provided a structured framework for examining differences in visit times across diverse economic and regional contexts. Our results reflect the literature by showing a considerable gap between HICs and UMICs compared to LMICs and LICS. Mean visit times in the Americas appears to be driving this with a visit time of 24.5 min compared to regions such as Africa (11.7 min), Eastern Mediterranean (10.2) and South-East Asia (9.7), see Table 1 and Fig. 3) [13]. This likely reflects global patterns of workforce density [20] and general challenges in healthcare delivery in low-income settings [44]. The results of this study could direct priorities of policy makers with visit times offering a proxy for workforce adequacy. Policies must aim to address health workforce training and retention to improve access to adequate care but must be evidence based and include an assessment of the costs and benefits of using finite resources to enact the policy.

Visit time variation has important implications for quality of care and health equity as well as health workforce planning. Shorter visit times in LMICs and LICs may limit the quality of assessment and diagnosis required to deliver effective healthcare. The impact will likely be more profound in populations with the highest need. A study in a high-resource setting revealed longer visits occur for those with multimorbidity but not for the most deprived population [45]. Thus, unmet need may grow amongst the most vulnerable in countries/regions that have limited capacity. The results of this study can highlight the regions at greatest risk. Financing to support quality rather than quantity may offer a policy option to ensure the delivery of the Sustainable Development Goal (Goal 3) of ensuring healthy lives and promoting well-being for all at all ages [46].

Our study allows primary care physician results [13] to be placed amongst other health workers such as specialist physician and nursing professionals, albeit GP visit time was the only statistically significant health worker visit time in the *meta*-regression. The results of the *meta*-regression revealed the visit times amongst GPs and specialist physicians to be shorter than other health worker visit times by 2.88 and 0.60 min respectively. Nursing visit times were approximately 5.79 min longer than GP visits and 3.51 min longer than specialist physician. A comparison of the mean visit time across GPs, SPs and nurses in countries based on income classifications has been provided in the appendix (A9) to highlight the increased time nurses spend in visits compared to doctors across countries. This increased time spent with nurses may account for the similar or greater patient health and patient satisfaction when comparing nurse and physician consultations [47]. Increased visit time for nurses may have policy implications for health system planning, particularly policies such as task shifting to tackle the global health workforce shortage. Ample evidence exists advocating for task shifting from physicians to non-physician health workers, including nurses, in resource constrained settings [48]. The results of the *meta*-regression and those in appendix A9 suggest shifting visits from doctors to nurses would increase visit times but whether the relative abundance of nurse to doctors would offset this and improve access is unknown. Placing nurse visit times amongst doctor visit times can help provide policymakers with evidence to evaluate the implications of task shifting and whether it represents a good use of limited healthcare resources [49].

Initial visits were shown to be longer than follow-up visits in all models, albeit the coefficients were not statistically significant. We captured whether the visit was an initial or follow-up visit to try and capture a degree of visit complexity. Evidence has shown complexity in the form of multimorbidity to be a visit time modifier [21]. Single health issues, however, can also be complex and it was considered by the authors that the nature of the visit type as a new or follow-up patient visit could influence the visit type as it may require a more comprehensive history to be taken as well as a physical examination.

The results revealed statistically significant shorter visit times for doctors conducting visits via telemedicine compared to those in-person. A systematic review by Hajebrahimi et al. [50] found no difference in visit times when comparing telemedicine studies to in-person studies. However, Hajebrahimi based their analysis on eight studies, whereas the *meta*-regression reported in this systematic review included 34 telemedicine studies and 143 in-person studies. A theory of change analysis could be conducted to evaluate how telemedicine creates change in visit times as well as improved access, as seen during the COVID-19 pandemic [51].

In all *meta*-regression results, there were no statistically significant differences in visit times from studies conducted during the COVID-19 pandemic compared to pre-pandemic but the results did indicate shorter visits during the COVID-19 period. This may have implications for pandemic preparedness and planning [52] but the results should be interpreted with caution as the data are limited to only 34 synthesized studies conducted between March 2020 – December 2021.

Only two studies (Keyworth [53] and Zivanovich [54]) captured evidence of visit times in prehospital emergency services. During the title and abstract screening and full text screening, many prehospital emergency service studies reported outcomes as ‘length of stay’ of an individual in the emergency department. Strict criteria in the systematic review limited the evidence to time spent with health providers meaning the vast majority of emergency care studies were excluded at this stage. It could be conceived that capturing the time spent with health providers is difficult in an emergency care setting as the triage team, doctors, and nurses may have multiple discrete interactions with a patient. A future systematic review that captures the length of stay in an emergency department and explores length of stay modifiers may aid service planning and costing of services.

The univariate *meta*-analysis indicated heterogeneity in the sample, (τ^2^ = 333) which was explored through sub-group analyses but the τ^2^ remained high. Best practice dictates further heterogeneity exploration through *meta*-regression to allow multiple factors to be explored simultaneously [29]. The *meta*-regression showed a reduction in the τ^2^ (τ^2^ = 15.61 in the service delivery platform model 1), however, such a τ^2^ may still be considered high making the results uncertain [[55], [56]]. Any unaccounted-for heterogeneity may be as a result of modifiers described in the literature but not captured in this study, for example the geographic location of the practice and health provider factors such as sex and age [22], [23], [24].

The heterogeneity may also be a result of the nature of the evidence being synthesised. As the evidence was observational in nature there may be diversity of study designs, populations, and settings [57]. The results include prospective and retrospective studies as well as a range of measurement methods such as surveys and time-motion studies. Meta-analysing such studies requires careful subjective consideration of whether there is too much diversity [[29], [57]]. The authors considered it appropriate to combine in synthesis with the incorporation of a rigorous exploration of the heterogeneity whilst accounting for dependent observations [33], [36]. Systematic review without *meta*-analysis may have offered an appropriate alternative in the presence of considerable heterogeneity. Yet, without quantitative synthesis interpretation becomes more subjective and selective making it difficult to assess the weight of evidence.

The identification of global variation in visit time was considered important to help inform evidence-based policies for global health systems [43], to aid healthcare resource planning, costing of services, and informing health benefit package development [7].

### 4.2 Strengths and limitations

This is a large systematic review identifying and incorporating a considerable breadth of evidence (n = 422) and resulting in a rich *meta*-analytic dataset. The size of the sample included in the synthesis is considerably larger than those identified through similar systematic reviews albeit studies in the literature limited their visits to those with doctors [[13], [21]]. This presents a further strength of our work in that we place visit times with doctors amongst those with other health professionals. Our study also offers a novel contribution to the visit time literature in that our synthesis followed best practice and explored the abundant heterogeneity through *meta*-analysis and *meta*-regression [29], [33]. As such we consider this study a significant contribution to the evidence-based underpinning workforce planning with the aim of improving healthcare service quality and equity.

It is important to note that the study deviated from the protocol in the form of the omission of studies prior to 2017. The mid-review deviation was made for pragmatic reasons but was reported in the PRISMA diagram as per the guidelines [25]. In the *meta*-regression estimations, there was also a degree of *ex-poste* analysis in the form of the categorisation of the variables. Despite this, the modifiers were outlined *ex-ante,* reducing the likelihood of spurious findings. It is also important to note that modifiers that may have an important impact on the visit time cannot be fully explored in *meta*-regression as the study is based on aggregate data introducing the potential for aggregation bias in the results [[58], [59], [60]].

A further limitation is in the difficulty of identifying studies that explicitly report time spent exclusively between health worker and patient during a visit. Decisions on the accuracy of the evidence in capturing time spent with patients were made during the screening stage. It is therefore possible that some studies that did accurately capture the visit time in terms of time between patient and health worker were excluded and likewise studies may have been included that captured non-health provider-patient time in their results.

Finally, the search strategy was limited in the incorporation of grey literature. The decision not to comprehensively search the grey literature was for a number of reasons. First, the authors made a pragmatic decision to ensure a manageable number of studies being screened. Second, the authors were aware of a number of systematic reviews of similar topics that had been conducted in which a comprehensive search of the grey literature was conducted. As the protocol of this systematic review included searching the reference lists of any identified systematic reviews, the authors thought grey literature would be captured through this approach. The authors did attempt to search for multi-country resource-use/costing studies through expert consultation to try and identify relevant grey literature.

The overrepresentation of countries from high-income settings has offered evidence of the considerable gaps in the literature for low-income countries (see Fig. 2), however it also poses limitations in the generalisability of the results. Visit times in low-income settings may include contextual differences in the health system and demographics meaning visit time estimates may not translate to other settings. Applications to policy may also be limited at a national level by the coverage of the included data. Many of the included studies were based on subnational or selective samples and limited longitudinal data gathered at the national level may constrain the extent to which the findings translate directly into country-level policy.

Overall, this is the first study that provides a comprehensive overview and consolidation of the recent literature on visit times, across healthcare providers, service delivery platforms, and country classifications. These findings can help decision makers form more evidence-based decisions on health workforce policy and planning. Additionally, our estimates on visit times can be used by researchers conducting costing studies or developing cost-effectiveness models where data on visit times has not been collected.

## CRediT authorship contribution statement

**Peter Murphy:** Writing – review & editing, Writing – original draft, Visualization, Methodology, Investigation, Formal analysis, Data curation, Conceptualization. **Wajeeha Raza:** Writing – review & editing, Methodology, Investigation, Data curation, Conceptualization. **Filip Meheus:** Writing – review & editing, Methodology, Investigation, Funding acquisition, Data curation, Conceptualization. **Altea Sitruk:** Writing – review & editing, Methodology, Investigation, Funding acquisition, Data curation, Conceptualization. **Kratu Goel:** Writing – review & editing, Methodology, Investigation, Data curation, Conceptualization.

## Funding

This work was funded by the 10.13039/100004423World Health Organization.

## Declaration of competing interest

The authors declare that they have no known competing financial interests or personal relationships that could have appeared to influence the work reported in this paper.

## Footnotes

1 We classified studies as being conducted in the COVID-19 pandemic if it was explicitly described in the study that the research was conducted in the pandemic or if data were collected between March 2020 – December 2021.

2 Disclaimer: The term “countries” or “national” should be understood to refer to countries and areas. The designations employed and the presentation of the material in this platform do not imply the expression of any opinion whatsoever on the part of WHO concerning the legal status of any country, territory, city, or area or of its authorities, or concerning the delimitation of its frontiers or boundaries.
