# Supplementary material for: Global health worker visit time variation: A systematic review
Source: Health Policy Open. 2026 Apr 6;10:100169. doi: 10.1016/j.hpopen.2026.100169 (PMC13092665; doi:10.1016/j.hpopen.2026.100169)
Supplement: Supplementary Data 1 [file mmc1.docx]

# APPENDIX

## A1 Search Strategy

Databases included:

Listed in the protocol:

PubMEd (Medline)

Scopus

ISI Web of Science

Cochrane Systematic Reviews

CINAHL

Additional:

Cochrane CENTRAL

Econlit

Note: IBSS, Global Health, Health Economic Evaluation Database (HEED) and ProQuest Dissertation & Theses Global were all listed in the protocol; however, these were not included in the search due to no access to the databases.

Cochrane CENTRAL was included as an additional database search as was EconLit.

Results summary

**Total number of results from the database searches: 23,364**

**Total number of deduplicated results: 15,863**

**Search Results**

Ovid MEDLINE(R) <1996 to October Week 4 2022>

Via Ovid <http://ovidsp.ovid.com/>

Date range searched: 1996 to October Week 2 2022

Date searched: 30th October 2022

1 exp "Referral and Consultation"/ 63910

2 consult*.ti,ab,kf. 112531

3 Encounter.ti,ab,kf. 31918

4 Visit*.ti,ab,kf. 219512

5 Appointment.ti,ab,kf. 12925

6 or/1-5 401944

7 exp Health Occupations/ 1052683

8 exp Health personnel/ 422996

9 or/7-8 1362696

10 exp Time/ 901692

11 exp time factors/ 776419

12 duration.ti,ab,kf. 469141

13 Length.ti,ab,kf. 490221

14 Minute.ti,ab,kf. 76943

15 or/10-14 1791941

16 ((Consult* or encounter or visit* or appointment) adj (duration or length or time*)).ti,ab,kf. 2655

17 6 and 9 and 15 8967

18 16 or 17 11029

19 limit 18 to yr="2002-Current" 9806

/ = indexing term (Medical Subject Heading: MeSH)

exp = exploded indexing term (MeSH)

? = replaces 0 or 1 character

* = truncation

ti,ab,tw,kf = terms in either title, abstract, textword or keyword heading word fields

jw,in = terms in either journal word or institution fields

adj3 = terms within three words of each other (any order)

**Cochrane Database of Systematic Reviews (CDSR)**

via Cochrane Library: <https://www.cochranelibrary.com/>

Date range searched: 2002 to Oct 2022

Date searched: 30^h^ Oct 2022

Records retrieved: 36

ID Search Hits

#1 MeSH descriptor: [Referral and Consultation] explode all trees 2553

#2 ("consult*"):ti,ab,kw 1559

#3 (Encounter):ti,ab,kw 2612

#4 ("visit*"):ti,ab,kw 59363

#5 MeSH descriptor: [Appointments and Schedules] explode all trees 1146

#6 {OR #1-#5} 66096

#7 MeSH descriptor: [Health Occupations] explode all trees 24146

#8 MeSH descriptor: [Health Personnel] explode all trees 10525

#9 {OR #7-#8} 32445

#10 MeSH descriptor: [Time] explode all trees 72251

#11 MeSH descriptor: [Time Factors] explode all trees 67177

#12 ("duration"):ti,ab,kw 178224

#13 (length):ti,ab,kw 58991

#14 (minute):ti,ab,kw 43041

#15 {OR #10-#14} 316317

#16 ((Consult* or encounter or visit* or appointment) NEXT (duration or length or time*)):ti,ab,kw 1167

#17 (#6 and #9 and #15) 527

#18 (1-#17) 36

Key:

MeSH descriptor = Medical Subject Heading (MeSH) term

“” = fields from the search manager

ti,ab,kw = terms in either title or abstract or keyword fields

**Cochrane CENTRAL**

via Cochrane Library: <https://www.cochranelibrary.com/>

Date range searched: 2002 to Oct 2022

Date searched: 30^h^ Oct 2022

Records retrieved: 1624

ID Search Hits

#1 MeSH descriptor: [Referral and Consultation] explode all trees 2553

#2 ("consult*"):ti,ab,kw 1559

#3 (Encounter):ti,ab,kw 2612

#4 ("visit*"):ti,ab,kw 59363

#5 MeSH descriptor: [Appointments and Schedules] explode all trees 1146

#6 {OR #1-#5} 66096

#7 MeSH descriptor: [Health Occupations] explode all trees 24146

#8 MeSH descriptor: [Health Personnel] explode all trees 10525

#9 {OR #7-#8} 32445

#10 MeSH descriptor: [Time] explode all trees 72251

#11 MeSH descriptor: [Time Factors] explode all trees 67177

#12 ("duration"):ti,ab,kw 178224

#13 (length):ti,ab,kw 58991

#14 (minute):ti,ab,kw 43041

#15 {OR #10-#14} 316317

#16 ((Consult* or encounter or visit* or appointment) NEXT (duration or length or time*)):ti,ab,kw 1167

#17 (#6 and #9 and #15) 527

#18 (1-#17) 1624

Key:

MeSH descriptor = Medical Subject Heading (MeSH) term

“” = fields from the search manager

ti,ab,kw = terms in either title or abstract or keyword fields

Econlit

Via Ovid <http://ovidsp.ovid.com/>

Date range searched: 1996 to October Week 2 2022

Date searched: 30th October 2022

Econlit <1886 to October 20, 2022>

1 (consult* or encounter or visit* or appointment or counsel* or session).ti,ab,kw. 16101

2 (((Acupuncture or audiolog* or occupational therap* or physical therap* or physical-therap* or speech language or speech-language or speech) and language) or chiropract* or dentistry or dental or oral medicine or oral-medicine or oral surg* or oral-surg* or sanitation or medical illustration or medical-illustration or radiolog* or medic* or Physician or Doctor or Specialist or allergy or immunolog* or anesthes* or anaesthes* or anesthet* or anaesthet* or dermatology or general practic* or general-practic* or family practic* or family-practic* or primary care physic* or primary-care physic* or family physic* or geriatric* or cardiolog* or endocrinolog* or gastroenterolog* or hematolog* or oncolog* or nephrolog* or rheumatolog* or neurolog* or pediatric* or paediatric* or neonatolog* or perinatolog* or rehabilitation or psychiatr* or androlog* or gynaecolog* or gynecolog* or OBGYN or OB-GYN or OB GYN or surg* or neurosurg* or ophthalmolog* or optometr* or orthopaedic* or orthopaedic* or otolaryngology* or traumatolog* or urolog* or nurs* or pharmac* or podiatr* or psychol* or psycho- or Hygienist or technician* or denturist or care-giver or caregiver or care giver or volunteer* or counsellor).mp. or advocate.ti,ab,kw. 58815

3 (Community health extension worker or Lady health worker or Health coach or Community health advisor or Family advocate or Outreach worker or Peer counsellor or Patient navigator or Health interpreter or Public health aide or Community Health Agents or Community Health Assistant or Maternal Health Worker or Community Nutrition Worker or Maternal & Child Health Promotion Workers or Community-based Worker or Community-based Health Worker or Maternal Child Health Worker or Nutrition Worker or Mental Health Worker or Postnatal Support Worker or Community-based Skilled Birth Attendant or Lay health worker or Volunteer health worker or Village health worker or Village Malaria Worker or Female Community Health Volunteer or Voluntary Malaria Worker or Nutrition Volunteer or Community Health Volunteer or Village Health Guide or Community Drug Distributor or Village Health Helper or Mother Coordinator or Village Drug-Kit Manager or Community Reproductive Health Worker or Lay Health Visitor or Community Volunteer or Community Health Advocate or Community Health Aide or Village Health Promoter or Rural Health Worker or Traditional Midwife or Community Volunteer or Lay Counselor or Volunteer Counselor or Volunteer Peer Counselor or Peer Support Worker or Shasthyo Sebika or Agente Comunitario de Salud or Saksham Sahaya or Visitadora or Anganwadi Workers or Promotoras de Salud or Raedat or Accompagnateur or Behvarz or Kader Posyandu or Brigadistas or Colaborador Voluntario or Dai or Bidan Kampong or Dayas or Doot).ti,ab,kw. 160

4 (primary care or primary-care or secondary care or secondary-care or tertiary care or tertiary-care or hospital or practice or community).ti,ab,kw. 68434

5 or/2-4 119412

6 (duration or length or minute or Interval).ti,ab,kw. 23103

7 1 and 5 and 6 146

8 limit 7 to yr="2002-Current" 134

**Key:**

? = replaces 0 or 1 character

* = truncation

ti,ab  = terms in either title or abstract fields

jx,in = terms in either journal word or institution fields

adj3 = terms within three words of each other (any order)

**Science Citation Index Expanded**

via Web of Science, Clarivate Analytics <https://clarivate.com/>

Date range searched: 1900 – 29^h^ October 2022

Date searched: 30^th^ October 2022

Records retrieved: 6010

# Searches:

1: TS=((consult* or encounter or visit* or appointment) NEAR/1 (duration or length or minute or Interval or time)) Timespan: 2002-01-01 to 2022-10-30 Date Run: Sun Oct 30 2022 09:59:38 GMT+0000 (Greenwich Mean Time) Results: 10886

2: TS=(Acupuncture OR audiolog* OR “occupational therap*” OR “physical therap*” OR “physical-therap*” OR “speech language” OR “speech-language” OR “speech and language” OR chiropract* OR dentistry OR dental or “oral medicine” OR “oral-medicine” OR “oral surg*” OR “oral-surg*” OR sanitation OR “medical illustration” OR “medical-illustration” OR radiolog* OR medic* OR Physician OR Doctor OR Specialist OR allergy OR immunolog* OR anesthes* OR anaesthes* OR anesthet* OR anaesthet* OR dermatology OR “general practic*” OR “general-practic*” OR “family practic*” OR “family-practic*” OR “primary care physic*” OR “primary-care physic*” OR “family physic*” OR geriatric* OR cardiolog* OR endocrinolog* OR gastroenterolog* OR hematolog* OR oncolog* OR nephrolog* OR rheumatolog* OR neurolog* OR pediatric* or paediatric* or neonatolog* OR perinatolog* OR rehabilitation OR psychiatr* OR androlog* OR gynaecolog* OR gynecolog* OR OBGYN OR “OB-GYN” or OB GYN OR surg* OR neurosurg* OR ophthalmolog* OR optometr* OR orthopaedic* OR orthopaedic* OR otolaryngology* OR traumatolog* OR urolog* OR nurs* OR pharmac* OR podiatr* OR psychol* OR psycho- OR Hygienist OR technician* OR denturist OR “care-giver” OR caregiver OR “care giver” OR carer OR volunteer* OR counsellor or advocate) Timespan: 2002-01-01 to 2022-10-30 Date Run: Sun Oct 30 2022 10:06:25 GMT+0000 (Greenwich Mean Time) Results: 7005057

3: TS=(“Community health extension worker” or “Lady health worker” or “Health coach” or “Community health advisor” or “Family advocate” or “Outreach worker” or “Peer counsellor” or “Patient navigator” or “Health interpreter” or “Public health aide” or “Community Health Agents” or “Community Health Assistant” or “Maternal Health Worker” or “Community Nutrition Worker” or “Maternal & Child Health Promotion Workers” or “Community-based Worker” or “Community-based Health Worker” or “Maternal Child Health Worker” or “Nutrition Worker” or “Mental Health Worker” or “Postnatal Support Worker” or “Community-based Skilled Birth Attendant” or “Lay health worker” or “Volunteer health worker” or “Village health worker” or “Village Malaria Worker” or “Female Community Health Volunteer” or “Voluntary Malaria Worker” or “Nutrition Volunteer” or “Community Health Volunteer” or “Village Health Guide” or “Community Drug Distributor” or “Village Health Helper” or “Mother Coordinator” or “Village Drug-Kit Manager” or “Community Reproductive Health Worker” or “Lay Health Visitor” or “Community Volunteer” or “Community Health Advocate” or “Community Health Aide” or “Village Health Promoter” or “Rural Health Worker” or “Traditional Midwife” or “Community Volunteer” or “Lay Counselor” or “Volunteer Counselor” or “Volunteer Peer Counselor” or “Peer Support Worker” or “Shasthyo Sebika” or “Agente Comunitario de Salud” or “Saksham Sahaya” or “Visitadora” or “Anganwadi Workers” or “Promotoras de Salud” or “Raedat” or “Accompagnateur” or “Behvarz” or “Kader Posyandu” or “Brigadistas” or “Colaborador Voluntario” or “Dai” or “Bidan Kampong” or “Dayas” or “Doot”) Timespan: 2002-01-01 to 2022-10-30 Date Run: Sun Oct 30 2022 10:07:29 GMT+0000 (Greenwich Mean Time) Results: 8881

4: #2 OR #3 Timespan: 2002-01-01 to 2022-10-30 Date Run: Sun Oct 30 2022 10:07:48 GMT+0000 (Greenwich Mean Time) Results: 7011228

5: #1 AND #4 Timespan: 2002-01-01 to 2022-10-30 Date Run: Sun Oct 30 2022 10:08:00 GMT+0000 (Greenwich Mean Time) Results: 6010

**Key:**

TS= terms in either title, abstract, author keywords, and keywords plus fields

TI= search in title field

AB= search in abstract field

CU= search in country/region field

SO= search in publication name field

CI= search in city field

OO= search in organization field

NEAR/3  = terms within three words of each other (any order).

* = truncation

1. **Cinahl**

via EBSCOhost [www.ebscohost.com](http://www.ebscohost.com)

Date range searched: 1900 – October 2022

Date searched: 30^th^ October 2022

Records retrieved: 2968

| S6 | S5 | Limiters - Published Date: 20020101-20221031 Expanders - Apply equivalent subjects Search modes - Boolean/Phrase | Interface - EBSCOhost Research Databases Search Screen - Advanced Search Database - CINAHL Complete | 2,968 |
| --- | --- | --- | --- | --- |
| S5 | (S1 and S4) | Expanders - Apply equivalent subjects Search modes - Boolean/Phrase | Interface - EBSCOhost Research Databases Search Screen - Advanced Search Database - CINAHL Complete | 3,242 |
| S4 | (S2 OR S3) | Expanders - Apply equivalent subjects Search modes - Boolean/Phrase | Interface - EBSCOhost Research Databases Search Screen - Advanced Search Database - CINAHL Complete | 4,214,738 |
| S3 | AB (Community health extension worker or Lady health worker or Health coach or Community health advisor or Family advocate or Outreach worker or Peer counsellor or Patient navigator or Health interpreter or Public health aide or Community Health Agents or Community Health Assistant or Maternal Health Worker or Community Nutrition Worker or Maternal & Child Health Promotion Workers or Community-based Worker or Community-based Health Worker or Maternal Child Health Worker or Nutrition Worker or Mental Health Worker or Postnatal Support Worker or Community-based Skilled Birth Attendant or Lay health worker or Volunteer health worker or Village health worker or Village Malaria Worker or Female Community Health Volunteer or Voluntary Malaria Worker or Nutrition Volunteer or Community Health Volunteer or Village Health Guide or Community Drug Distributor or Village Health Helper or Mother Coordinator or Village Drug-Kit Manager or Community Reproductive Health Worker or Lay Health Visitor or Community Volunteer or Community Health Advocate or Community Health Aide or Village Health Promoter or Rural Health Worker or Traditional Midwife or Community Volunteer or Lay Counselor or Volunteer Counselor or Volunteer Peer Counselor or Peer Support Worker or Shasthyo Sebika or Agente Comunitario de Salud or Saksham Sahaya or Visitadora or Anganwadi Workers or Promotoras de Salud or Raedat or Accompagnateur or Behvarz or Kader Posyandu or Brigadistas or Colaborador Voluntario or Dai or Bidan Kampong or Dayas or Doot) | Expanders - Apply equivalent subjects Search modes - Boolean/Phrase | Interface - EBSCOhost Research Databases Search Screen - Advanced Search Database - CINAHL Complete | 10,161 |
| S2 | AB (Acupuncture or audiolog* or occupational therap* or physical therap* or physical-therap* or speech language or speech-language or speech) and language) or chiropract* or dentistry or dental or oral medicine or oral-medicine or oral surg* or oral-surg* or sanitation or medical illustration or medical-illustration or radiolog* or medic* or Physician or Doctor or Specialist or allergy or immunolog* or anesthes* or anaesthes* or anesthet* or anaesthet* or dermatology or general practic* or general-practic* or family practic* or family-practic* or primary care physic* or primary-care physic* or family physic* or geriatric* or cardiolog* or endocrinolog* or gastroenterolog* or hematolog* or oncolog* or nephrolog* or rheumatolog* or neurolog* or pediatric* or paediatric* or neonatolog* or perinatolog* or rehabilitation or psychiatr* or androlog* or gynaecolog* or gynecolog* or OBGYN or OB-GYN or OB GYN or surg* or neurosurg* or ophthalmolog* or optometr* or orthopaedic* or orthopaedic* or otolaryngology* or traumatolog* or urolog* or nurs* or pharmac* or podiatr* or psychol* or psycho- or Hygienist or technician* or denturist or care-giver or caregiver or care giver or volunteer* or counsellor or advocate) | Expanders - Apply equivalent subjects Search modes - Boolean/Phrase | Interface - EBSCOhost Research Databases Search Screen - Advanced Search Database - CINAHL Complete | 4,212,231 |
| S1 | AB ((consult* or encounter or visit* or appointment) N1 (duration or length or minute or Interval or time)) | Expanders - Apply equivalent subjects Search modes - Boolean/Phrase | Interface - EBSCOhost Research Databases Search Screen - Advanced Search Database - CINAHL Complete | 3,981 |

**Key:**

TS= terms in either title, abstract, author keywords, and keywords plus fields

TI= search in title field

AB= search in abstract field

SO= search in publication name field

CI= search in city field

OO= search in organization field

N  = terms within x words of each other (any order).

* = truncation

1. **Scopus**

via Scopus <https://www.scopus.com/>

Date range searched: 2002 – 2022

Date searched: 30^th^ October 2022

Records retrieved: 2786

( TITLE-ABS-KEY ( ( consult* OR encounter OR visit* OR appointment ) W/1 ( duration OR length OR minute OR interval OR time ) ) ) AND ( ( TITLE-ABS-KEY ( ( acupuncture OR audiolog* OR "occupational therap*" OR "physical therap*" OR "physical-therap*" OR "speech language" OR "speech-language" OR "speech and language" OR chiropract* OR dentistry OR dental OR "oral medicine" OR "oral-medicine" OR "oral surg*" OR "oral-surg*" OR sanitation OR "medical illustration" OR "medical-illustration" OR radiolog* OR medic* OR physician OR doctor OR specialist OR allergy OR immunolog* OR anesthes* OR anaesthes* OR anesthet* OR anaesthet* OR dermatology OR "general practic*" OR "general-practic*" OR "family practic*" OR "family-practic*" OR "primary care physic*" OR "primary-care physic*" OR "family physic*" OR geriatric* OR cardiolog* OR endocrinolog* OR gastroenterolog* OR hematolog* OR oncolog* OR nephrolog* OR rheumatolog* OR neurolog* OR pediatric* OR paediatric* OR neonatolog* OR perinatolog* OR rehabilitation OR psychiatr* OR androlog* OR gynaecolog* OR gynecolog* OR obgyn OR "ob-gyn" OR ob AND gyn OR surg* OR neurosurg* OR ophthalmolog* OR optometr* OR orthopaedic* OR orthopaedic* OR otolaryngology* OR traumatolog* OR urolog* OR nurs* OR pharmac* OR podiatr* OR psychol* OR psycho- OR hygienist OR technician* OR denturist OR "care-giver" OR caregiver OR "care giver" OR volunteer* OR counsellor OR advocate ) ) ) OR ( TITLE-ABS-KEY ( ( "community health extension worker" OR "lady health worker" OR "health coach" OR "community health advisor" OR "family advocate" OR "outreach worker" OR "peer counsellor" OR "patient navigator" OR "health interpreter" OR "public health aide" OR "community health agents" OR "community health assistant" OR "maternal health worker" OR "community nutrition worker" OR "maternal & child health promotion workers" OR "community-based worker" OR "community-based health worker" OR "maternal child health worker" OR "nutrition worker" OR "mental health worker" OR "postnatal support worker" OR "community-based skilled birth attendant" OR "lay health worker" OR "volunteer health worker" OR "village health worker" OR "village malaria worker" OR "female community health volunteer" OR "voluntary malaria worker" OR "nutrition volunteer" OR "community health volunteer" OR "village health guide" OR "community drug distributor" OR "village health helper" OR "mother coordinator" OR "village drug-kit manager" OR "community reproductive health worker" OR "lay health visitor" OR "community volunteer" OR "community health advocate" OR "community health aide" OR "village health promoter" OR "rural health worker" OR "traditional midwife" OR "community volunteer" OR "lay counselor" OR "volunteer counselor" OR "volunteer peer counselor" OR "peer support worker" OR "shasthyo sebika" OR "agente comunitario de salud" OR "saksham sahaya" OR "visitadora" OR "anganwadi workers" OR "promotoras de salud" OR "raedat" OR "accompagnateur" OR "behvarz" OR "kader posyandu" OR "brigadistas" OR "colaborador voluntario" OR "dai" OR "bidan kampong" OR "dayas" OR "doot" ) ) ) ) AND ( LIMIT-TO ( PUBYEAR , 2023 ) OR LIMIT-TO ( PUBYEAR , 2022 ) OR LIMIT-TO ( PUBYEAR , 2021 ) OR LIMIT-TO ( PUBYEAR , 2020 ) OR LIMIT-TO ( PUBYEAR , 2019 ) OR LIMIT-TO ( PUBYEAR , 2018 ) OR LIMIT-TO ( PUBYEAR , 2017 ) OR LIMIT-TO ( PUBYEAR , 2016 ) OR LIMIT-TO ( PUBYEAR , 2015 ) OR LIMIT-TO ( PUBYEAR , 2014 ) OR LIMIT-TO ( PUBYEAR , 2013 ) OR LIMIT-TO ( PUBYEAR , 2012 ) OR LIMIT-TO ( PUBYEAR , 2011 ) OR LIMIT-TO ( PUBYEAR , 2010 ) OR LIMIT-TO ( PUBYEAR , 2009 ) OR LIMIT-TO ( PUBYEAR , 2008 ) OR LIMIT-TO ( PUBYEAR , 2007 ) OR LIMIT-TO ( PUBYEAR , 2006 ) OR LIMIT-TO ( PUBYEAR , 2005 ) OR LIMIT-TO ( PUBYEAR , 2004 ) OR LIMIT-TO ( PUBYEAR , 2003 ) OR LIMIT-TO ( PUBYEAR , 2002 ) )

TOTAL=2786

**Updated searches**

**Ovid MEDLINE(R) <1946 to July Week 2 2024>**

1 exp "Referral and Consultation"/ 88742

2 consult*.ti,ab,kf. 146771

3 Encounter.ti,ab,kf. 39773

4 Visit*.ti,ab,kf. 272446

5 Appointment.ti,ab,kf. 16867

6 or/1-5 513461

7 exp Health Occupations/ 1893079

8 exp Health personnel/ 636881

9 or/7-8 2362250

10 exp Time/ 1426392

11 exp time factors/ 1235504

12 duration.ti,ab,kf. 639189

13 Length.ti,ab,kf. 634910

14 Minute.ti,ab,kf. 111633

15 or/10-14 2616466

16 ((Consult* or encounter or visit* or appointment) adj (duration or length or time*)).ti,ab,kf. 3307

17 6 and 9 and 15 10801

18 16 or 17 13391

19 limit 18 to dt=20221030-20240630 785

**Econlit <1886 to July 11, 2024>**

1 (consult* or encounter or visit* or appointment or counsel* or session).ti,ab,kw. 17716

2 (((Acupuncture or audiolog* or occupational therap* or physical therap* or physical-therap* or speech language or speech-language or speech) and language) or chiropract* or dentistry or dental or oral medicine or oral-medicine or oral surg* or oral-surg* or sanitation or medical illustration or medical-illustration or radiolog* or medic* or Physician or Doctor or Specialist or allergy or immunolog* or anesthes* or anaesthes* or anesthet* or anaesthet* or dermatology or general practic* or general-practic* or family practic* or family-practic* or primary care physic* or primary-care physic* or family physic* or geriatric* or cardiolog* or endocrinolog* or gastroenterolog* or hematolog* or oncolog* or nephrolog* or rheumatolog* or neurolog* or pediatric* or paediatric* or neonatolog* or perinatolog* or rehabilitation or psychiatr* or androlog* or gynaecolog* or gynecolog* or OBGYN or OB-GYN or OB GYN or surg* or neurosurg* or ophthalmolog* or optometr* or orthopaedic* or orthopaedic* or otolaryngology* or traumatolog* or urolog* or nurs* or pharmac* or podiatr* or psychol* or psycho- or Hygienist or technician* or denturist or care-giver or caregiver or care giver or volunteer* or counsellor).mp. or advocate.ti,ab,kw. 67238

3 (Community health extension worker or Lady health worker or Health coach or Community health advisor or Family advocate or Outreach worker or Peer counsellor or Patient navigator or Health interpreter or Public health aide or Community Health Agents or Community Health Assistant or Maternal Health Worker or Community Nutrition Worker or Maternal & Child Health Promotion Workers or Community-based Worker or Community-based Health Worker or Maternal Child Health Worker or Nutrition Worker or Mental Health Worker or Postnatal Support Worker or Community-based Skilled Birth Attendant or Lay health worker or Volunteer health worker or Village health worker or Village Malaria Worker or Female Community Health Volunteer or Voluntary Malaria Worker or Nutrition Volunteer or Community Health Volunteer or Village Health Guide or Community Drug Distributor or Village Health Helper or Mother Coordinator or Village Drug-Kit Manager or Community Reproductive Health Worker or Lay Health Visitor or Community Volunteer or Community Health Advocate or Community Health Aide or Village Health Promoter or Rural Health Worker or Traditional Midwife or Community Volunteer or Lay Counselor or Volunteer Counselor or Volunteer Peer Counselor or Peer Support Worker or Shasthyo Sebika or Agente Comunitario de Salud or Saksham Sahaya or Visitadora or Anganwadi Workers or Promotoras de Salud or Raedat or Accompagnateur or Behvarz or Kader Posyandu or Brigadistas or Colaborador Voluntario or Dai or Bidan Kampong or Dayas or Doot).ti,ab,kw. 173

4 (primary care or primary-care or secondary care or secondary-care or tertiary care or tertiary-care or hospital or practice or community).ti,ab,kw. 74465

5 or/2-4 132961

6 (duration or length or minute or Interval).ti,ab,kw. 25039

7 1 and 5 and 6 171

8 limit 7 to yr="2022 - 2024" 24

**CDSR**

Search Name:

Date Run: 23/07/2024 12:57:15

Comment:

Warning: Problems were found with one or more of your search lines (specific lines are identified below). For best results, you should review and edit the search lines indicated.

ID Search Hits

#1 MeSH descriptor: [Referral and Consultation] explode all trees 3404

#2 ("consult*"):ti,ab,kw 1409

#3 (Encounter):ti,ab,kw 3294

#4 ("visit*"):ti,ab,kw 69738

#5 MeSH descriptor: [Appointments and Schedules] explode all trees 1412

#6 {OR #1-#5} 77972

#7 MeSH descriptor: [Health Occupations] explode all trees 36215

#8 MeSH descriptor: [Health Personnel] explode all trees 15614

#9 {OR #7-#8} 48762

#10 MeSH descriptor: [Time] explode all trees 88321

#11 MeSH descriptor: [Time Factors] explode all trees 81650

#12 ("duration"):ti,ab,kw 208846

#13 (length):ti,ab,kw 69825

#14 (minute):ti,ab,kw 50772

#15 {OR #10-#14} 373503

#16 ((Consult* or encounter or visit* or appointment) NEXT (duration or length or time*)):ti,ab,kw 1398

#17 (#6 and #9 and #15) 665

#18 [**Error**]==> (1-#17) with Cochrane Library publication date Between Oct 2022 and Jul 2024

# Web of Science Search Strategy (v0.1)

# Database: Web of Science Core Collection

# Entitlements:

- WOS.SCI: 1900 to 2024

- WOS.AHCI: 1975 to 2024

- WOS.ESCI: 2015 to 2024

- WOS.ISTP: 1990 to 2024

- WOS.SSCI: 1956 to 2024

- WOS.ISSHP: 1990 to 2024

**Web of Science**

# Searches:

1: TS=((consult* or encounter or visit* or appointment) NEAR/1 (duration or length or minute or Interval or time)) Timespan: 2022-10-30 to 2024-06-30 Date Run: Tue Jul 23 2024 11:05:42 GMT+0100 (British Summer Time) Results: 1704

2: TS=(Acupuncture OR audiolog* OR “occupational therap*” OR “physical therap*” OR “physical-therap*” OR “speech language” OR “speech-language” OR “speech and language” OR chiropract* OR dentistry OR dental or “oral medicine” OR “oral-medicine” OR “oral surg*” OR “oral-surg*” OR sanitation OR “medical illustration” OR “medical-illustration” OR radiolog* OR medic* OR Physician OR Doctor OR Specialist OR allergy OR immunolog* OR anesthes* OR anaesthes* OR anesthet* OR anaesthet* OR dermatology OR “general practic*” OR “general-practic*” OR “family practic*” OR “family-practic*” OR “primary care physic*” OR “primary-care physic*” OR “family physic*” OR geriatric* OR cardiolog* OR endocrinolog* OR gastroenterolog* OR hematolog* OR oncolog* OR nephrolog* OR rheumatolog* OR neurolog* OR pediatric* or paediatric* or neonatolog* OR perinatolog* OR rehabilitation OR psychiatr* OR androlog* OR gynaecolog* OR gynecolog* OR OBGYN OR “OB-GYN” or OB GYN OR surg* OR neurosurg* OR ophthalmolog* OR optometr* OR orthopaedic* OR orthopaedic* OR otolaryngology* OR traumatolog* OR urolog* OR nurs* OR pharmac* OR podiatr* OR psychol* OR psycho- OR Hygienist OR technician* OR denturist OR “care-giver” OR caregiver OR “care giver” OR carer OR volunteer* OR counsellor or advocate) Timespan: 2022-06-30 to 2024-10-30 Date Run: Tue Jul 23 2024 11:06:48 GMT+0100 (British Summer Time) Results: 1311652

3: TS=(“Community health extension worker” or “Lady health worker” or “Health coach” or “Community health advisor” or “Family advocate” or “Outreach worker” or “Peer counsellor” or “Patient navigator” or “Health interpreter” or “Public health aide” or “Community Health Agents” or “Community Health Assistant” or “Maternal Health Worker” or “Community Nutrition Worker” or “Maternal & Child Health Promotion Workers” or “Community-based Worker” or “Community-based Health Worker” or “Maternal Child Health Worker” or “Nutrition Worker” or “Mental Health Worker” or “Postnatal Support Worker” or “Community-based Skilled Birth Attendant” or “Lay health worker” or “Volunteer health worker” or “Village health worker” or “Village Malaria Worker” or “Female Community Health Volunteer” or “Voluntary Malaria Worker” or “Nutrition Volunteer” or “Community Health Volunteer” or “Village Health Guide” or “Community Drug Distributor” or “Village Health Helper” or “Mother Coordinator” or “Village Drug-Kit Manager” or “Community Reproductive Health Worker” or “Lay Health Visitor” or “Community Volunteer” or “Community Health Advocate” or “Community Health Aide” or “Village Health Promoter” or “Rural Health Worker” or “Traditional Midwife” or “Community Volunteer” or “Lay Counselor” or “Volunteer Counselor” or “Volunteer Peer Counselor” or “Peer Support Worker” or “Shasthyo Sebika” or “Agente Comunitario de Salud” or “Saksham Sahaya” or “Visitadora” or “Anganwadi Workers” or “Promotoras de Salud” or “Raedat” or “Accompagnateur” or “Behvarz” or “Kader Posyandu” or “Brigadistas” or “Colaborador Voluntario” or “Dai” or “Bidan Kampong” or “Dayas” or “Doot”) Timespan: 2022-06-30 to 2024-10-30 Date Run: Tue Jul 23 2024 11:07:39 GMT+0100 (British Summer Time) Results: 1855

4: #2 OR #3 Timespan: 2022-10-30 to 2024-06-30 Date Run: Tue Jul 23 2024 11:08:06 GMT+0100 (British Summer Time) Results: 989672

5: #1 AND #4 Timespan: 2022-10-30 to 2024-06-30 Date Run: Tue Jul 23 2024 11:08:36 GMT+0100 (British Summer Time) Results: 1055

**Cinahl**

**Tue, July 23, 2024 11:20:08 AM**

| Tue, July 23, 2024 11:20:08 AM |
| --- |

| **#** | **Query** | **Limiters/Expanders** | **Last Run Via** | **Results** |
| --- | --- | --- | --- | --- |
| S6 | S5 | Limiters - Publication Date: 20221001-20240631 Expanders - Apply equivalent subjects Search modes - Proximity | Interface - EBSCOhost Research Databases Search Screen - Advanced Search Database - CINAHL Ultimate | 303 |
| S5 | (S1 and S4) | Expanders - Apply equivalent subjects Search modes - Proximity | Interface - EBSCOhost Research Databases Search Screen - Advanced Search Database - CINAHL Ultimate | 3,451 |
| S4 | (S2 OR S3) | Expanders - Apply equivalent subjects Search modes - Proximity | Interface - EBSCOhost Research Databases Search Screen - Advanced Search Database - CINAHL Ultimate | 4,426,330 |
| S3 | AB (Community health extension worker or Lady health worker or Health coach or Community health advisor or Family advocate or Outreach worker or Peer counsellor or Patient navigator or Health interpreter or Public health aide or Community Health Agents or Community Health Assistant or Maternal Health Worker or Community Nutrition Worker or Maternal & Child Health Promotion Workers or Community-based Worker or Community-based Health Worker or Maternal Child Health Worker or Nutrition Worker or Mental Health Worker or Postnatal Support Worker or Community-based Skilled Birth Attendant or Lay health worker or Volunteer health worker or Village health worker or Village Malaria Worker or Female Community Health Volunteer or Voluntary Malaria Worker or Nutrition Volunteer or Community Health Volunteer or Village Health Guide or Community Drug Distributor or Village Health Helper or Mother Coordinator or Village Drug-Kit Manager or Community Reproductive Health Worker or Lay Health Visitor or Community Volunteer or Community Health Advocate or Community Health Aide or Village Health Promoter or Rural Health Worker or Traditional Midwife or Community Volunteer or Lay Counselor or Volunteer Counselor or Volunteer Peer Counselor or Peer Support Worker or Shasthyo Sebika or Agente Comunitario de Salud or Saksham Sahaya or Visitadora or Anganwadi Workers or Promotoras de Salud or Raedat or Accompagnateur or Behvarz or Kader Posyandu or Brigadistas or Colaborador Voluntario or Dai or Bidan Kampong or Dayas or Doot) | Expanders - Apply equivalent subjects Search modes - Proximity | Interface - EBSCOhost Research Databases Search Screen - Advanced Search Database - CINAHL Ultimate | 10,981 |
| S2 | occupational therap* or physical therap* or physical-therap* or speech language or speech-language or speech) and language) or chiropract* or dentistry or dental or oral medicine or oral-medicine or oral surg* or oral-surg* or sanitation or medical illustration or medical-illustration or radiolog* or medic* or Physician or Doctor or Specialist or allergy or immunolog* or anesthes* or anaesthes* or anesthet* or anaesthet* or dermatology or general practic* or general-practic* or family practic* or family-practic* or primary care physic* or primary-care physic* or family physic* or geriatric* or cardiolog* or endocrinolog* or gastroenterolog* or hematolog* or oncolog* or nephrolog* or rheumatolog* or neurolog* or pediatric* or paediatric* or neonatolog* or perinatolog* or rehabilitation or psychiatr* or androlog* or gynaecolog* or gynecolog* or OBGYN or OB-GYN or OB GYN or surg* or neurosurg* or ophthalmolog* or optometr* or orthopaedic* or orthopaedic* or otolaryngology* or traumatolog* or urolog* or nurs* or pharmac* or podiatr* or psychol* or psycho- or Hygienist or technician* or denturist or care-giver or caregiver or care giver or volunteer* or counsellor or advocate) | Expanders - Apply equivalent subjects Search modes - Proximity | Interface - EBSCOhost Research Databases Search Screen - Advanced Search Database - CINAHL Ultimate | 4,423,632 |
| S1 | AB ((consult* or encounter or visit* or appointment) N1 (duration or length or minute or Interval or time)) | Expanders - Apply equivalent subjects Search modes - Proximity | Interface - EBSCOhost Research Databases Search Screen - Advanced Search Database - CINAHL Ultimate | 4,225 |

SCOPUS

TITLE-ABS-KEY ( ( consult* OR encounter OR visit* OR appointment ) W/1 ( duration OR length OR minute OR interval OR time ) ) AND ( ( TITLE-ABS-KEY ( ( acupuncture OR audiolog* OR "occupational therap*" OR "physical therap*" OR "physical-therap*" OR "speech language" OR "speech-language" OR "speech and language" OR chiropract* OR dentistry OR dental OR "oral medicine" OR "oral-medicine" OR "oral surg*" OR "oral-surg*" OR sanitation OR "medical illustration" OR "medical-illustration" OR radiolog* OR medic* OR physician OR doctor OR specialist OR allergy OR immunolog* OR anesthes* OR anaesthes* OR anesthet* OR anaesthet* OR dermatology OR "general practic*" OR "general-practic*" OR "family practic*" OR "family-practic*" OR "primary care physic*" OR "primary-care physic*" OR "family physic*" OR geriatric* OR cardiolog* OR endocrinolog* OR gastroenterolog* OR hematolog* OR oncolog* OR nephrolog* OR rheumatolog* OR neurolog* OR pediatric* OR paediatric* OR neonatolog* OR perinatolog* OR rehabilitation OR psychiatr* OR androlog* OR gynaecolog* OR gynecolog* OR obgyn OR "ob-gyn" OR ob AND gyn OR surg* OR neurosurg* OR ophthalmolog* OR optometr* OR orthopaedic* OR orthopaedic* OR otolaryngology* OR traumatolog* OR urolog* OR nurs* OR pharmac* OR podiatr* OR psychol* OR psycho- OR hygienist OR technician* OR denturist OR "care-giver" OR caregiver OR "care giver" OR volunteer* OR counsellor OR advocate ) ) ) ) OR ( TITLE-ABS-KEY ( ( "community health extension worker" OR "lady health worker" OR "health coach" OR "community health advisor" OR "family advocate" OR "outreach worker" OR "peer counsellor" OR "patient navigator" OR "health interpreter" OR "public health aide" OR "community health agents" OR "community health assistant" OR "maternal health worker" OR "community nutrition worker" OR "maternal & child health promotion workers" OR "community-based worker" OR "community-based health worker" OR "maternal child health worker" OR "nutrition worker" OR "mental health worker" OR "postnatal support worker" OR "community-based skilled birth attendant" OR "lay health worker" OR "volunteer health worker" OR "village health worker" OR "village malaria worker" OR "female community health volunteer" OR "voluntary malaria worker" OR "nutrition volunteer" OR "community health volunteer" OR "village health guide" OR "community drug distributor" OR "village health helper" OR "mother coordinator" OR "village drug-kit manager" OR "community reproductive health worker" OR "lay health visitor" OR "community volunteer" OR "community health advocate" OR "community health aide" OR "village health promoter" OR "rural health worker" OR "traditional midwife" OR "community volunteer" OR "lay counselor" OR "volunteer counselor" OR "volunteer peer counselor" OR "peer support worker" OR "shasthyo sebika" OR "agente comunitario de salud" OR "saksham sahaya" OR "visitadora" OR "anganwadi workers" OR "promotoras de salud" OR "raedat" OR "accompagnateur" OR "behvarz" OR "kader posyandu" OR "brigadistas" OR "colaborador voluntario" OR "dai" OR "bidan kampong" OR "dayas" OR "doot" ) ) ) AND PUBYEAR > 2021 AND PUBYEAR < 2025

Total = 683

## A2 Data extraction pro-forma

| **Publication number:** |  |
| --- | --- |
| **Reviewer name:** |  |
| **Publication title:** |  |
| **Author(s):** |  |
| **Journal/Publisher:** |  |
| **Citation:** |  |
| **Year of publication:** |  |
| **Country/countries:** |  |
| **Main objective(s):** |  |
| **Study design:** | Cross-sectional study  Case study  Descriptive study  Experimental study  Time series study  Time and motion study  Costing study  Systematic review  Other, specify: |
| **Study population:** |  |
| **Disease(s)/health condition(s):** | Primary care  Emergency care  Health Promotion  Non-communicable disease  Tuberculosis  Child Health  Nutrition  Malaria  Adolescent Health  Palliative care  Primary care  Surgery and anesthetic care  Environmental health  Rehabilitation  Maternal and newborn health  Mental health, neurologic disorders and substance use  Sexual and reproductive health  Violence prevention  HIV  Hepatitis  Immunization  Injury prevention  School health  Occupational health  Neglected tropical diseases  Healthy ageing  Disability  Other, specify: |
| **Method of measurement:** | Recording (video, audio, stopwatch)  Self-reported/questionnaire  Software tracking  Existing databases or guidelines  Other, specify: |
| **Approach:** | Normative  Descriptive |
| **Purpose of study:** | Costing  Workforce Planning  Quality Standards  Other, specify: |
| **Type of visit:** | Preventive  Initial  Follow-up  Procedure  Emergency care  Other, specify: |
| **Visit description:** |  |
| **Visit duration (minutes):** |  |
| **Service delivery platform(s):** | Community-based services  Prehospital emergency services  General outpatient services  First referral level  Second referral level (and above) |
| **Health worker type(s):** |  |
| **Additional notes:** |  |

## A3 PRISMA checklist

| **Section and Topic** | **Item #** | **Checklist item** | **Location where item is reported** |
| --- | --- | --- | --- |
| **TITLE** | | |  |
| Title | 1 | Identify the report as a systematic review. | 1 |
| **ABSTRACT** | | |  |
| Abstract | 2 | See the PRISMA 2020 for Abstracts checklist. |  |
| **INTRODUCTION** | | |  |
| Rationale | 3 | Describe the rationale for the review in the context of existing knowledge. | 2, 3 |
| Objectives | 4 | Provide an explicit statement of the objective(s) or question(s) the review addresses. | 3 |
| **METHODS** | | |  |
| Eligibility criteria | 5 | Specify the inclusion and exclusion criteria for the review and how studies were grouped for the syntheses. | 4-6 |
| Information sources | 6 | Specify all databases, registers, websites, organisations, reference lists and other sources searched or consulted to identify studies. Specify the date when each source was last searched or consulted. | 4 |
| Search strategy | 7 | Present the full search strategies for all databases, registers and websites, including any filters and limits used. | appendix |
| Selection process | 8 | Specify the methods used to decide whether a study met the inclusion criteria of the review, including how many reviewers screened each record and each report retrieved, whether they worked independently, and if applicable, details of automation tools used in the process. | 4, 5 |
| Data collection process | 9 | Specify the methods used to collect data from reports, including how many reviewers collected data from each report, whether they worked independently, any processes for obtaining or confirming data from study investigators, and if applicable, details of automation tools used in the process. | 4-6 |
| Data items | 10a | List and define all outcomes for which data were sought. Specify whether all results that were compatible with each outcome domain in each study were sought (e.g. for all measures, time points, analyses), and if not, the methods used to decide which results to collect. | 4-6 |
|  | 10b | List and define all other variables for which data were sought (e.g. participant and intervention characteristics, funding sources). Describe any assumptions made about any missing or unclear information. | n/a |
| Study risk of bias assessment | 11 | Specify the methods used to assess risk of bias in the included studies, including details of the tool(s) used, how many reviewers assessed each study and whether they worked independently, and if applicable, details of automation tools used in the process. | 5 |
| Effect measures | 12 | Specify for each outcome the effect measure(s) (e.g. risk ratio, mean difference) used in the synthesis or presentation of results. | 5 |
| Synthesis methods | 13a | Describe the processes used to decide which studies were eligible for each synthesis (e.g. tabulating the study intervention characteristics and comparing against the planned groups for each synthesis (item #5)). | 5, 6 |
|  | 13b | Describe any methods required to prepare the data for presentation or synthesis, such as handling of missing summary statistics, or data conversions. | 5, 6 |
|  | 13c | Describe any methods used to tabulate or visually display results of individual studies and syntheses. | 6, 7 |
|  | 13d | Describe any methods used to synthesize results and provide a rationale for the choice(s). If meta-analysis was performed, describe the model(s), method(s) to identify the presence and extent of statistical heterogeneity, and software package(s) used. | 6, 7 |
|  | 13e | Describe any methods used to explore possible causes of heterogeneity among study results (e.g. subgroup analysis, meta-regression). | 6, 7 |
|  | 13f | Describe any sensitivity analyses conducted to assess robustness of the synthesized results. | n/a |
| Reporting bias assessment | 14 | Describe any methods used to assess risk of bias due to missing results in a synthesis (arising from reporting biases). | n/a |
| Certainty assessment | 15 | Describe any methods used to assess certainty (or confidence) in the body of evidence for an outcome. | n/a |
| **RESULTS** | | |  |
| Study selection | 16a | Describe the results of the search and selection process, from the number of records identified in the search to the number of studies included in the review, ideally using a flow diagram. | 8 |
|  | 16b | Cite studies that might appear to meet the inclusion criteria, but which were excluded, and explain why they were excluded. | 8 |
| Study characteristics | 17 | Cite each included study and present its characteristics. | appendix |
| Risk of bias in studies | 18 | Present assessments of risk of bias for each included study. | appendix |
| Results of individual studies | 19 | For all outcomes, present, for each study: (a) summary statistics for each group (where appropriate) and (b) an effect estimate and its precision (e.g. confidence/credible interval), ideally using structured tables or plots. | appendix |
| Results of syntheses | 20a | For each synthesis, briefly summarise the characteristics and risk of bias among contributing studies. | 8, 9 |
|  | 20b | Present results of all statistical syntheses conducted. If meta-analysis was done, present for each the summary estimate and its precision (e.g. confidence/credible interval) and measures of statistical heterogeneity. If comparing groups, describe the direction of the effect. | 9-11 |
|  | 20c | Present results of all investigations of possible causes of heterogeneity among study results. | 11 |
|  | 20d | Present results of all sensitivity analyses conducted to assess the robustness of the synthesized results. | n/a |
| Reporting biases | 21 | Present assessments of risk of bias due to missing results (arising from reporting biases) for each synthesis assessed. | n/a |
| Certainty of evidence | 22 | Present assessments of certainty (or confidence) in the body of evidence for each outcome assessed. | n/a |
| **DISCUSSION** | | |  |
| Discussion | 23a | Provide a general interpretation of the results in the context of other evidence. | 11, 12 |
|  | 23b | Discuss any limitations of the evidence included in the review. | 14, 15 |
|  | 23c | Discuss any limitations of the review processes used. | 14 |
|  | 23d | Discuss implications of the results for practice, policy, and future research. | 15 |
| **OTHER INFORMATION** | | |  |
| Registration and protocol | 24a | Provide registration information for the review, including register name and registration number, or state that the review was not registered. | 3 |
|  | 24b | Indicate where the review protocol can be accessed, or state that a protocol was not prepared. | 3 |
|  | 24c | Describe and explain any amendments to information provided at registration or in the protocol. | 5 |
| Support | 25 | Describe sources of financial or non-financial support for the review, and the role of the funders or sponsors in the review. | Cover page |
| Competing interests | 26 | Declare any competing interests of review authors. | Cover page |
| Availability of data, code and other materials | 27 | Report which of the following are publicly available and where they can be found: template data collection forms; data extracted from included studies; data used for all analyses; analytic code; any other materials used in the review. | appendix |

## A4 Results Table

| **Author** | **Country** | **Method of measurement** | **Service delivery platform** | **UHCC Health worker** | **Mean visit time (min)** | **Median visit time (min)** | **Other (qualitative)** | **Uncertainty (min)** | **Tele-medicine** | **COVID-19** |
| --- | --- | --- | --- | --- | --- | --- | --- | --- | --- | --- |
| Abdu-Aguye(2) | Nigeria | Survey | Community-based services | Pharmacists | 2 | - | 1 minute (40%); 2 minutes (30%); 3 minutes (20%); 4 minutes (10%) | - | N | N |
|  | Nigeria | Survey | Community-based services | Pharmacists | 1.3 | - | 1 minute (46.7%); 2 minutes (43.3%); 3 minutes (10.0%); 4 minutes (0%) | - | N | N |
| Abdulkader(3) | Saudi Arabia | Self-reported/questionnaire | Second referral level (and above) | Specialist Medical Practitioners | 14.7 | - | - | SD, 4 | N | N |
| Abdus-Salam(4) | Nigeria | Self-reported/questionnaire | First referral level | Specialist Medical Practitioners | 13.46 | - | - | SD, 12.16 | N | N |
| Abner(5) | England | Existing databases or guidelines | General outpatient services | Nursing Professionals | 11.2 | - | Estimated from the following: the average duration of GP consultations with T2DM and cardiometabolic multimorbidity patients were respectively 5.9 (SD 7.84) and 5.7 (SD 8.38) minutes in 2000, and increased by 45% and 54%, respectively, in 2018 | - | N | N |
|  | England | Existing databases or guidelines | General outpatient services | Nursing Professionals | 10.8 | - | Estimated from the following: the average duration of GP consultations with T2DM and cardiometabolic multimorbidity patients were respectively 5.9 (SD 7.84) and 5.7 (SD 8.38) minutes in 2000, and increased by 45% and 54%, respectively, in 2018 | - | N | N |
|  | England | Existing databases or guidelines | General outpatient services | General Medical Practitioners | 8.6 | - | Estimated from the following: the average duration of GP consultations with T2DM and cardiometabolic multimorbidity patients were respectively 5.9 (SD 7.84) and 5.7 (SD 8.38) minutes in 2000, and increased by 45% and 54%, respectively, in 2018 | - | N | N |
|  | England | Existing databases or guidelines | General outpatient services | General Medical Practitioners | 8.8 | - | Estimated from the following: the average duration of GP consultations with T2DM and cardiometabolic multimorbidity patients were respectively 5.9 (SD 7.84) and 5.7 (SD 8.38) minutes in 2000, and increased by 45% and 54%, respectively, in 2018 | - | N | N |
|  | England | Existing databases or guidelines | General outpatient services | General Medical Practitioners | 9.7 | - | - | SD, 10.02 | N | N |
|  | England | Existing databases or guidelines | General outpatient services | General Medical Practitioners | 9 | - | - | SD, 9.39 | N | N |
|  | England | Existing databases or guidelines | General outpatient services | General Medical Practitioners | 9.8 | - | - | SD, 9.68 | N | N |
| Aboueid(6) | Canada | Self-reported/questionnaire | General outpatient services | General Medical Practitioners | 15 | - | - | - | N | N |
|  | Canada | Self-reported/questionnaire | General outpatient services | General Medical Practitioners | 40 | - | - | - | N | N |
|  | Canada | Self-reported/questionnaire | General outpatient services | Nursing Professionals | 20 | - | - | - | N | N |
|  | Canada | Self-reported/questionnaire | General outpatient services | Nursing Professionals | 30 | - | - | - | N | N |
|  | Canada | Self-reported/questionnaire | General outpatient services | General Medical Practitioners | 15 | - | - | - | N | N |
| Abushaala(7) | UK | Recording (video, audio, stopwatch) | First referral level | Nursing Professionals | 10 | - | - | - | Y | N |
| Ah-Kye(8) | UK | Existing databases or guidelines | Other, specify: | Specialist Medical Practitioners | 8 | - | - | - | Y | Y |
| Ahmad(9) | Malaysia | Recording (video, audio, stopwatch) | General outpatient services | General Medical Practitioners | 18.21 | - | - | - | N | N |
|  | Malaysia | Other, specify: | General outpatient services | General Medical Practitioners |  | NA |  | - | N | N |
|  | Malaysia | Recording (video, audio, stopwatch) | General outpatient services | General Medical Practitioners | 60 | - | - | - | N | N |
|  | Malaysia | Recording (video, audio, stopwatch) | General outpatient services | General Medical Practitioners | 50 | \| - \| - \| \| --- \| --- \| | - | - | N | N |
|  | Malaysia | Recording (video, audio, stopwatch) | General outpatient services | General Medical Practitioners | 18.2 | \| - \| - \| \| --- \| --- \| | - | - | N | N |
|  | Malaysia | Recording (video, audio, stopwatch) | General outpatient services | General Medical Practitioners | 25.2 | - | - | - | N | N |
|  | Malaysia | Recording (video, audio, stopwatch) | General outpatient services | General Medical Practitioners | 12 | - | - | - | N | N |
|  | Malaysia | Recording (video, audio, stopwatch) | General outpatient services | General Medical Practitioners | 16.8 | - | - | - | N | N |
|  | Malaysia | Recording (video, audio, stopwatch) | General outpatient services | General Medical Practitioners | 13.3 | - | - | - | N | N |
|  | Malaysia | Recording (video, audio, stopwatch) | General outpatient services | General Medical Practitioners | 18 | - | - | - | N | N |
|  | Malaysia | Recording (video, audio, stopwatch) | General outpatient services | General Medical Practitioners | 19.2 | - | - | - | N | N |
|  | Malaysia | Recording (video, audio, stopwatch) | General outpatient services | General Medical Practitioners | 18.7 | - | - | - | N | N |
| Alarcon-Ruiz(10) | Peru | Self-reported/questionnaire | General outpatient services | General Medical Practitioners | 12.1 | - | - | 95% CI, 11.4 - 12.7 | N |  |
|  | Peru | Self-reported/questionnaire | General outpatient services | General Medical Practitioners | 12.3 | - | - | 95% CI, 11.4 - 13.2 | N |  |
|  | Peru | Self-reported/questionnaire | General outpatient services | General Medical Practitioners | 11.3 | - | - | 95% CI, 10.2 - 12.4 | N |  |
|  | Peru | Self-reported/questionnaire | General outpatient services | General Medical Practitioners | 10.7 | - | - | 95% CI, 8.6 - 12.8 | N |  |
|  | Peru | Self-reported/questionnaire | General outpatient services | General Medical Practitioners | 14.4 | - | - | 95% CI, 12.1 - 16.8 | N |  |
| Al-Habbal (11) | Lebanon | Self-reported/questionnaire | General outpatient services | General Medical Practitioners | 14.05 | - | Assumed the 5.96 is the SD not the SE. Results also reported: Less than 5 mins (6.4%); 6-10 mins (22.3%); 11-15 mins (28.2); 16-20 mins (20.3); More than 20 mins (17.3%) | SD, 5.96 | N | N |
| Al-Harajin (12) | Saudi Arabia | Self-reported/questionnaire | First referral level | Specialist Medical Practitioners | - | NA | >=20 mins (70.7%); <20 mins (29.3%) | - | N | N |
|  | Saudi Arabia | Self-reported/questionnaire | First referral level | General Medical Practitioners | - | NA | >=20 mins (78.1%); <20 mins (21.9%) | - | N | N |
|  | Saudi Arabia | Self-reported/questionnaire | First referral level | Specialist Medical Practitioners | - | NA | >=20 mins (63.4%); <20 mins (36.6%) | - | N | N |
| Aljehani (13) | Saudi Arabia | Self-reported/questionnaire | General outpatient services | General Medical Practitioners | - | NA | <=10 mins (28.4%); 10-20 mins (57.2%); >20 mins (14.4%) | - | N | Y |
| Allen (14) | England (assumed) | Recording (video, audio, stopwatch) | First referral level | Specialist Medical Practitioners | 9.283 | - | 9 minutes 17 seconds | - | N | N |
| Alsubeeh (15) | Saudi Arabia | Self-reported/questionnaire | General outpatient services | Specialist Medical Practitioners |  | NA | 207 patients with less than 10 minutes considered revision and 160 patients with less than 10 minutes did not consider revision. Less than 10 min (n=207); 10–15 min (n=214); 16-20 min (n=96); 21-25 min (n=30); 25-30 (n=33); more than 30 min (n=32). | - | N | N |
|  | Saudi Arabia | Self-reported/questionnaire | General outpatient services | Specialist Medical Practitioners |  | NA | 214 patients with 10 - 15 minutes considered revision and 229 patients with 10 - 15 minutes did not consider revision. Less than 10 min (n=160); 10–15 min (n=229); 16-20 min (n=148); 21-25 min (n=73); 25-30 (n=78); more than 30 min (n=70). | - | N | N |
| Alzayer (16) | Saudi Arabia | Self-reported/questionnaire | First referral level | Specialist Medical Practitioners |  | NA | <10 mins (58.5%); 10-30 mins (39.2%); >30 mins (2.3%) | - | N | N |
| Andreyeva (17) | USA | Existing databases or guidelines | General outpatient services | Physiotherapists | 46.52 | - | - | SD, 18.51 | N | N |
| Ann-Yi (18) | USA | Self-reported/questionnaire | First referral level | Psychologists | 45 | - | - | - | N | N |
|  | USA | Self-reported/questionnaire | First referral level | Psychologists | 40 | - | - | - | N | N |
|  | USA | Self-reported/questionnaire | First referral level | Psychologists | 60 | - | - | - | N | N |
| Appiah (19) | USA | Self-reported/questionnaire | First referral level | General Medical Practitioners | 24 | - | - | 95% CI, 22.3 - 25.7 | N | N |
|  | USA | Self-reported/questionnaire | First referral level | General Medical Practitioners | 20.7 | - | - | 95% CI, 19.6 - 21.8 | N | N |
|  | USA | Self-reported/questionnaire | First referral level | General Medical Practitioners | 23.9 | - | - | 95% CI, 21.9 - 25.9 | N | N |
|  | USA | Self-reported/questionnaire | First referral level | General Medical Practitioners | 24.4 | - | - | 95% CI, 22.5 - 26.3 | N | N |
| Ariyo (20) | USA | Existing databases or guidelines | Community-based services | Pharmacists | 17 | - | - | - | N | N |
| Asamani (21) | Ghana | Existing databases or guidelines | General outpatient services | General Medical Practitioners | 16 | - | - | SD, 8.75 | N | N |
|  | Ghana | Existing databases or guidelines | General outpatient services | General Medical Practitioners | 9 | - | - | SD, 5.96 | N | N |
|  | Ghana | Existing databases or guidelines | First referral level | General Medical Practitioners | 15 | - | - | SD, 8.19 | N | N |
|  | Ghana | Existing databases or guidelines | General outpatient services | General Medical Practitioners | 14 | - | - | SD, 9.58; 95% CI, 10 - 17 | N | N |
|  | Ghana | Existing databases or guidelines | General outpatient services | General Medical Practitioners | 14 | - | - | SD, 8.29; 95% CI, 11 - 17 | N | N |
|  | Ghana | Existing databases or guidelines | First referral level | Midwifery Professionals | 22 | - | - | SD, 13.63; 95% CI, 18 - 26 | N | N |
|  | Ghana | Existing databases or guidelines | First referral level | Midwifery Professionals | 19 | - | - | SD, 8.2; 95% CI, 17 - 22 | N | N |
|  | Ghana | Existing databases or guidelines | First referral level | Midwifery Professionals | 16 | - | - | SD, 12.67; 95% CI, 12 - 21 | N | N |
|  | Ghana | Existing databases or guidelines | First referral level | Midwifery Professionals | 39 | - | - | SD, 13.21; 95% CI, 33 - 45 | N | N |
|  | Ghana | Existing databases or guidelines | First referral level | Midwifery Professionals | 17 | - | - | SD, 8.52; 95% CI, 14 - 19 | N | N |
|  | Ghana | Existing databases or guidelines | First referral level | Midwifery Professionals | 131 | - | - | SD, 97.23; 95% CI, 101 - 160 | N | N |
|  | Ghana | Existing databases or guidelines | First referral level | Midwifery Professionals | 30 | - | - | SD, 16.51; 95% CI, 24 - 36 | N | N |
|  | Ghana | Existing databases or guidelines | First referral level | Midwifery Professionals | 30 | - | - | SD, 18.62; 95% CI, 24 - 36 | N | N |
|  | Ghana | Existing databases or guidelines | First referral level | Midwifery Professionals | 22 | - | - | SD, 8.34; 95% CI, 19 - 24 | N | N |
|  | Ghana | Existing databases or guidelines | First referral level | Midwifery Professionals | 21 | - | - | SD, 8.83; 95% CI, 18 - 24 | N | N |
|  | Ghana | Existing databases or guidelines | First referral level | Midwifery Professionals | 23 | - | - | SD, 6.45; 95% CI, 21 - 25 | N | N |
|  | Ghana | Existing databases or guidelines | First referral level | Midwifery Professionals | 44 | - | - | SD, 12.86; 95% CI, 40 - 49 | N | N |
|  | Ghana | Existing databases or guidelines | First referral level | Nursing Professionals | 10 | - | - | SD, 3.85; 95% CI, 9 - 10 | N | N |
|  | Ghana | Existing databases or guidelines | First referral level | Nursing Professionals | 13 | - | - | SD, 5.24; 95% CI, 12 - 14 | N | N |
|  | Ghana | Existing databases or guidelines | First referral level | Nursing Professionals | 19 | - | - | SD, 6.98; 95% CI, 18 - 20 | N | N |
|  | Ghana | Existing databases or guidelines | First referral level | Nursing Professionals | 16 | - | - | SD, 9.37; 95% CI, 14 - 17 | N | N |
|  | Ghana | Existing databases or guidelines | First referral level | Nursing Professionals | 40 | - | - | SD, 14.82; 95% CI, 38 - 42 | N | N |
|  | Ghana | Existing databases or guidelines | First referral level | Nursing Professionals | 43 | - | - | SD, 21.29; 95% CI, 40 - 46 | N | N |
|  | Ghana | Existing databases or guidelines | First referral level | Nursing Professionals | 135 | - | - | SD, 55.64; 95% CI, 127 - 144 | N | N |
|  | Ghana | Existing databases or guidelines | First referral level | Nursing Professionals | 18 | - | - | SD, 8.07; 95% CI, 17 - 19 | N | N |
|  | Ghana | Existing databases or guidelines | General outpatient services | Nursing Professionals | 27 | - | - | SD, 16.74; 95% CI, 24 - 29 | N | N |
|  | Ghana | Existing databases or guidelines | General outpatient services | Nursing Professionals | 14 | - | - | SD, 8.93; 95% CI, 12 - 17 | N | N |
|  | Ghana | Existing databases or guidelines | General outpatient services | Nursing Professionals | 12 | - | - | SD, 6.54; 95% CI, 10 - 14 | N | N |
|  | Ghana | Existing databases or guidelines | General outpatient services | Nursing Professionals | 9 | - | - | SD, 6.4; 95% CI, 8 - 11 | N | N |
|  | Ghana | Existing databases or guidelines | General outpatient services | Nursing Professionals | 14 | - | - | SD, 10.82; 95% CI, 11 - 17 | N | N |
|  | Ghana | Existing databases or guidelines | General outpatient services | Nursing Professionals | 23 | - | - | SD, 11.92; 95% CI, 20 - 26 | N | N |
|  | Ghana | Existing databases or guidelines | General outpatient services | Nursing Professionals | 12 | - | - | SD, 7.88; 95% CI, 10 - 14 | N | N |
|  | Ghana | Existing databases or guidelines | General outpatient services | Nursing Professionals | 6 | - | - | SD, 3.56; 95% CI, 5 - 7 | N | N |
|  | Ghana | Existing databases or guidelines | General outpatient services | Nursing Professionals | 6 | - | - | SD, 2.95; 95% CI, 6 - 7 | N | N |
|  | Ghana | Existing databases or guidelines | General outpatient services | Dieticians and Nutritionists | 13 | - | - | SD, 11.26; 95% CI, 4 - 22 | N | N |
|  | Ghana | Existing databases or guidelines | General outpatient services | Dieticians and Nutritionists | 22 | - | - | SD, 6.83; 95% CI, 16 - 27 | N | N |
|  | Ghana | Existing databases or guidelines | General outpatient services | Dieticians and Nutritionists | 8 | - | - | SD, 4.88; 95% CI, 3 - 12 | N | N |
|  | Ghana | Existing databases or guidelines | General outpatient services | Dieticians and Nutritionists | 27 | - | - | SD, 17.54; 95% CI, 12 - 42 | N | N |
|  | Ghana | Existing databases or guidelines | General outpatient services | Dieticians and Nutritionists | 20 | - | - | SD, 10; 95% CI, 9 - 31 | N | N |
|  | Ghana | Existing databases or guidelines | General outpatient services | Dieticians and Nutritionists | 23 | - | - | SD, 18.93; 95% CI, 2 - 45 | N | N |
|  | Ghana | Existing databases or guidelines | General outpatient services | Pharmacists | 13 |  | - | SD, 5.19; 95% CI, 11 - 15 | N | N |
|  | Ghana | Existing databases or guidelines | General outpatient services | Health Professionals not elsewhere classified | 16 | - | - | SD, 8.59; 95% CI, 13 - 18 | N | N |
|  | Ghana | Existing databases or guidelines | General outpatient services | Health Professionals not elsewhere classified | 10 | - | - | SD, 5.59; 95% CI, 8 - 11 | N | N |
|  | Ghana | Existing databases or guidelines | First referral level | Health Professionals not elsewhere classified | 14 | - | - | SD, 7.39; 95% CI, 12 - 16 | N | N |
|  | Ghana | Existing databases or guidelines | General outpatient services | Health Professionals not elsewhere classified | 13 | - | - | SD, 7.72; 95% CI, 10 - 15 | N | N |
|  | Ghana | Existing databases or guidelines | General outpatient services | Health Professionals not elsewhere classified | 11 | - | - | SD, 6.64; 95% CI, 9 - 12 | N | N |
| Asan (22) | USA | Recording (video, audio, stopwatch) | General outpatient services | Specialist Medical Practitioners | 23.6 | - | - | SD, 11.2 | N | N |
| Assefa (23) | Ethiopia | Self-reported/questionnaire | First referral level | Pharmacists | 7 | - | - | SD, 4.69 | N | N |
| Atanda (24) | USA | Other, specify: | Second referral level (and above) | Specialist Medical Practitioners | NA | NA | Visit time for telemedicine vists was 17 mins & 88% of the visit time was spent with a provider = 14.96 mins | - | Y | N |
|  | USA | Other, specify: | Second referral level (and above) | Specialist Medical Practitioners | NA | NA | Visit time for inperson vists was 68 mins & 15% of the visit time was spent with a provider = 10.2 mins | - | N | N |
| Atia (25) | Libya | Self-reported/questionnaire | Community-based services | Pharmacists |  | NA | Less than a minute (85.9%); 1-5 minutes (14.1%) | - | N | N |
|  | Libya | Self-reported/questionnaire | Community-based services | Pharmaceutical Technicians and Assistants |  | NA | Less than a minute (61.2%); 1-5 minutes (38.8%) | - | N | N |
| Ayalew (26) | Ethiopia | Recording (video, audio, stopwatch) | First referral level | Pharmacists | 0.26 | - | 15.4 seconds | - | N | N |
| Ayele (27) | Ethiopia | Recording (video, audio, stopwatch) | Community-based services | Pharmacists | 2 | - | - | - | N | N |
|  | Ethiopia | Recording (video, audio, stopwatch) | Community-based services | Pharmacists | 2 | - | - | - | N | N |
| Barratt (28) | England | Recording (video, audio, stopwatch) | General outpatient services | Nursing Professionals | 10.97 | - | - | SD, 4.13 | N | N |
| Bauer (29) | USA | Other, specify: | Second referral level (and above) | Health associate professionals not elsewhere classified | 24.4 | - | - | SD, 11.31 | N | N |
|  | USA | Other, specify: | Second referral level (and above) | Health associate professionals not elsewhere classified | 25.32 | - | - | SD, 12.17 | N | Y |
|  | USA | Other, specify: | Second referral level (and above) | Health associate professionals not elsewhere classified | 25.38 | - | - | SD, 12 | N | Y |
| Beach (30) | USA | Recording (video, audio, stopwatch) | First referral level | Specialist Medical Practitioners | 30.4 | - | - | SD, 11.9 | N | N |
| Bener (31) | Turkey | Self-reported/questionnaire | General outpatient services | General Medical Practitioners | 7.95 | - | - | SD, 4.38 | N | N |
| Benski (i) (32) | Madagascar | Software tracking | General outpatient services | Midwifery Professionals | 26.2 | - | - | 95% CI, 25.5 - 26.9 | Y | N |
|  | Madagascar | Software tracking | General outpatient services | Midwifery Professionals | 25.3 | - | - | 95% CI, 24.7 - 26 | Y | N |
|  | Madagascar | Software tracking | General outpatient services | Midwifery Professionals | 22.7 | - | - | 95% CI, 22.2 - 23.2 | Y | N |
|  | Madagascar | Software tracking | General outpatient services | Midwifery Professionals | 20.5 | - | - | 95% CI, 19.9 - 21.1 | Y | N |
|  | Madagascar | Software tracking | General outpatient services | Midwifery Professionals | 30.4 | - | - | 95% CI, 30 - 30.9 | Y | N |
|  | Madagascar | Software tracking | General outpatient services | Midwifery Professionals | 20.9 | - | - | 95% CI, 20.3 - 21.4 | Y | N |
|  | Madagascar | Software tracking | General outpatient services | Midwifery Professionals | 19.6 | - | - | 95% CI, 19 - 20.2 | Y | N |
|  | Madagascar | Software tracking | General outpatient services | Midwifery Professionals | 19.9 | - | - | 95% CI, 19.2 - 20.6 | Y | N |
|  | Madagascar | Software tracking | General outpatient services | Midwifery Professionals | 19.1 | - | - | 95% CI, 18.4 - 19.9 | Y | N |
|  | Madagascar | Software tracking | General outpatient services | Midwifery Professionals | 29.2 | - | - | 95% CI, 27.8 - 30.5 | Y | N |
|  | Madagascar | Software tracking | General outpatient services | Midwifery Professionals | 25.5 | - | - | 95% CI, 25.1 - 25.9 | Y | N |
|  | Madagascar | Software tracking | General outpatient services | Midwifery Professionals | 21.7 | - | - | 95% CI, 21.7 - 22.1 | Y | N |
| Benski (ii) (33) | Madagascar | Software tracking | General outpatient services | Midwifery Professionals | 29.6 | - | - | - | Y | N |
| Berasa (34) | Ethiopia | Self-reported/questionnaire | First referral level | Pharmacists | 9 | - | - | - | N | N |
|  | Ethiopia | Self-reported/questionnaire | First referral level | Pharmacists | 5 | - | - | - | N | N |
|  | Ethiopia | Self-reported/questionnaire | First referral level | Pharmacists | 7 | - | - | - | N | N |
|  | Ethiopia | Self-reported/questionnaire | First referral level | Pharmacists | 4 | - | - | - | N | N |
|  | Ethiopia | Self-reported/questionnaire | First referral level | Pharmacists | 3 | - | - | - | N | N |
| Berk (35) | USA | Self-reported/questionnaire | General outpatient services | General Medical Practitioners | 40 | - | - | SD, 13.8 | N | N |
| Berkowitz (36) | USA | Other, specify: | General outpatient services | General Medical Practitioners | 9.14 | - | - | SD, 6.85 | N | N |
|  | USA | Other, specify: | General outpatient services | General Medical Practitioners | 17.09 | - | - | SD, 10.37 | N | N |
|  | USA | Other, specify: | General outpatient services | General Medical Practitioners | 9.8 | - | - | SD, 5.16 | N | N |
|  | USA | Other, specify: | General outpatient services | General Medical Practitioners | 16.22 | - | - | SD, 8.65 | N | N |
|  | USA | Other, specify: | General outpatient services | General Medical Practitioners | 9.21 | - | - | SD, 4.86 | N | N |
|  | USA | Other, specify: | General outpatient services | General Medical Practitioners | 18.48 | - | - | SD, 10.3 | N | N |
|  | USA | Other, specify: | General outpatient services | General Medical Practitioners | 10.57 | - | - | SD, 5.05 | N | N |
|  | USA | Other, specify: | General outpatient services | General Medical Practitioners | 16.71 | - | - | SD, 8.62 | N | N |
|  | USA | Other, specify: | General outpatient services | General Medical Practitioners | 11.46 | - | - | SD, 4.05 | N | N |
|  | USA | Other, specify: | General outpatient services | General Medical Practitioners | 11.22 | - | - | SD, 4.46 | N | N |
|  | USA | Other, specify: | General outpatient services | General Medical Practitioners | 12.7 | - | - | SD, 6.8 | N | N |
|  | USA | Other, specify: | General outpatient services | General Medical Practitioners | 15.95 | - | - | SD, 9.85 | N | N |
|  | USA | Other, specify: | General outpatient services | General Medical Practitioners | 8.37 | - | - | SD, 10.74 | N | N |
|  | USA | Other, specify: | General outpatient services | General Medical Practitioners | 14.89 | - | - | SD, 10.81 | N | N |
|  | USA | Other, specify: | General outpatient services | General Medical Practitioners | 7.43 | - | - | SD, 4.12 | N | N |
|  | USA | Other, specify: | General outpatient services | General Medical Practitioners | 15.17 | - | - | SD, 8.47 | N | N |
| Binyaruka (37) | Tanzania | Self-reported/questionnaire | General outpatient services | Pharmacists | 12.9 | - | - | - | N | N |
|  | Tanzania | Self-reported/questionnaire | General outpatient services | Pharmacists | 12.4 | - | - | - | N | N |
|  | Tanzania | Self-reported/questionnaire | General outpatient services | Pharmacists | 13.2 | - | - | - | N | N |
|  | Tanzania | Self-reported/questionnaire | General outpatient services | Pharmacists | 12.9 | - | - | - | N | N |
|  | Tanzania | Self-reported/questionnaire | General outpatient services | Pharmacists | 13.1 | - | - | - | N | N |
|  | Tanzania | Self-reported/questionnaire | General outpatient services | Pharmacists | 12.8 | - | - | - | N | N |
|  | Tanzania | Self-reported/questionnaire | General outpatient services | Pharmacists | 12.9 | - | - | - | N | N |
|  | Tanzania | Self-reported/questionnaire | General outpatient services | Pharmacists | 12.6 | - | - | - | N | N |
| Birhanu (38) | Ethiopia | Self-reported/questionnaire | General outpatient services |  | 12.5 | - | - | SD, 4.3 | N | N |
| Bissessor (39) | Australia | Software tracking | General outpatient services |  | 22.1 | - | - | - | N | N |
|  | Australia | Software tracking | General outpatient services |  | 2.5 | - | - | - | Y | N |
| Black (40) | UK | Self-reported/questionnaire | General outpatient services | Nursing Professionals | 24.9 | - | - | SD, 12.9 | N | N |
|  | UK | Self-reported/questionnaire | General outpatient services | Nursing Professionals | 27.3 | - | - | SD, 13 | N | N |
|  | UK | Self-reported/questionnaire | General outpatient services | Nursing Professionals | 19.5 | - | - | SD, 10.9 | N | N |
|  | UK | Self-reported/questionnaire | General outpatient services | Nursing Professionals | 25.7 | - | - | SD, 12.7 | N | N |
|  | UK | Self-reported/questionnaire | General outpatient services | Nursing Professionals | 23.3 | - | - | SD, 13.1 | N | N |
|  | UK | Self-reported/questionnaire | General outpatient services | Nursing Professionals | 22.8 | - | - | SD, 13.9 | N | N |
|  | UK | Self-reported/questionnaire | General outpatient services | Nursing Professionals | 25.7 | - | - | SD, 15.1 | N | N |
|  | UK | Self-reported/questionnaire | General outpatient services | Nursing Professionals | 19.4 | - | - | SD, 12 | N | N |
|  | UK | Self-reported/questionnaire | General outpatient services | Nursing Professionals | 23.3 | - | - | SD, 14.2 | N | N |
|  | UK | Self-reported/questionnaire | General outpatient services | Nursing Professionals | 22.1 | - | - | SD, 13.3 | N | N |
| Bock (41) | USA | Self-reported/questionnaire | General outpatient services | Nursing Professionals | 72.9 | - | - | - | N | N |
|  | USA | Self-reported/questionnaire | General outpatient services | Nursing Professionals | 54.2 | - | - | - | Y | Y |
| Boffa (42) | South Africa | Self-reported/questionnaire | General outpatient services | General Medical Practitioners | 9.6 | - | - | - | N | N |
|  | South Africa | Self-reported/questionnaire | General outpatient services | General Medical Practitioners | 11.3 | - | - | - | N | N |
|  | South Africa | Self-reported/questionnaire | General outpatient services | General Medical Practitioners | 9.6 | - | - | - | N | N |
| Bolívar (43) | Spain | Recording (video, audio, stopwatch) | General outpatient services | General Medical Practitioners | 8.8 | 8 | - | - | N | N |
|  | Spain | Recording (video, audio, stopwatch) | General outpatient services | General Medical Practitioners | 9.16 | - | - | SD, 3.88 | N | N |
|  | Spain | Recording (video, audio, stopwatch) | General outpatient services | General Medical Practitioners | 8.26 | - | - | SD, 3.76 | N | N |
|  | Spain | Recording (video, audio, stopwatch) | General outpatient services | General Medical Practitioners | 8.2 | - | - | SD, 3.11 | N | N |
|  | Spain | Recording (video, audio, stopwatch) | General outpatient services | General Medical Practitioners | 9.13 | - | - | SD, 4.09 | N | N |
|  | Spain | Recording (video, audio, stopwatch) | General outpatient services | General Medical Practitioners | 8.83 | - | - | SD, 3.7 | N | N |
|  | Spain | Recording (video, audio, stopwatch) | General outpatient services | General Medical Practitioners | 8.89 | - | - | SD, 4.1 | N | N |
| Bon (44) | France | Software tracking | Other, specify: | Health Professionals not elsewhere classified | 40 | - | - | - | Y | N |
|  | France | Software tracking | Other, specify: | Specialist Medical Practitioners | 6 | - | - | - | Y | N |
| Bonney (45) | Australia | Existing databases or guidelines | General outpatient services | General Medical Practitioners | 17.2 | - | - | SD, 9 | N | N |
|  | Australia | Existing databases or guidelines | General outpatient services | General Medical Practitioners | 17.5 | - | - | SD, 9.8 | N | N |
| Bowen (46) | USA | Self-reported/questionnaire | First referral level | Specialist Medical Practitioners | 15.3 | - | - | - | Y | Y |
| Brenner (47) | USA | Software tracking | General outpatient services | General Medical Practitioners | 13.07 | - | - | - | N | N |
| Breyer (48) | USA | Software tracking | First referral level | Specialist Medical Practitioners | 17.31 | - | - | 95% CI, 14.43 - 20.19 | N | N |
| Burger (49) | Netherlands | Recording (video, audio, stopwatch) | First referral level | Specialist Medical Practitioners | 15.91 | - | - | SD, 7.4 | N | N |
| Burnett-Zeiman (50) | Kenya | Recording (video, audio, stopwatch) | General outpatient services | Community Health Workers | 23 | - | - | - | N | N |
| Cabrera-Rivadeneyra (51) | Peru | Self-reported/questionnaire | Second referral level (and above) | Physiotherapists |  | NA | 2 to 25 minutes (52.4%); more than 25 minutes (47.6%) | - | N | N |
| Calvitti (52) | USA | Recording (video, audio, stopwatch) | First referral level | Specialist Medical Practitioners |  | 18.5 | The IQR is 13.1 to 25.0 mins | - | N | N |
| Castner (53) | USA | Software tracking | Second referral level (and above) | Specialist Medical Practitioners |  | 7.7 | - | SD, 29.5 | N | N |
|  | USA | Software tracking | Second referral level (and above) | Nursing Professionals | 19.2 | - | - | - | N | N |
|  | USA | Software tracking | Second referral level (and above) | Health Professionals not elsewhere classified | 38 | - | - | - | N | N |
| Chao (54) | USA |  | First referral level | Specialist Medical Practitioners | 6 | - | - | - | N | N |
|  | USA |  | First referral level | Specialist Medical Practitioners | 13 | - | - | - | N | N |
|  | USA |  | First referral level | Specialist Medical Practitioners | 7 | - | - | - | N | N |
|  | USA |  | First referral level | Specialist Medical Practitioners | 20 | - | - | - | N | N |
|  | USA |  | First referral level | Specialist Medical Practitioners | 9 | - | - | - | N | N |
|  | USA |  | First referral level | Specialist Medical Practitioners | 20 | - | - | - | N | N |
|  | USA |  | First referral level | Specialist Medical Practitioners | 30 | - | - | - | N | N |
|  | USA |  | First referral level | Specialist Medical Practitioners | 32 | - | - | - | N | N |
| Chebolu-Subramanian (55) | India | Recording (video, audio, stopwatch) | Community-based services | Community Health Workers | 19 | 17 | - | - | N | N |
|  | India | Recording (video, audio, stopwatch) | Community-based services | Community Health Workers | 31.53 | 25 | - | - | N | N |
|  | India | Recording (video, audio, stopwatch) | Community-based services | Community Health Workers | 9.38 | 8.5 | - | - | N | N |
| Cheung (56) | USA |  | Second referral level (and above) | Specialist Medical Practitioners | 43 | 42 | - | SD, 17 | Y | N |
| Chingombe (57) | Zimbabwe | Self-reported/questionnaire | First referral level | Specialist Medical Practitioners | 13 | - | - | - | N | N |
|  | Zimbabwe | Self-reported/questionnaire | First referral level | Specialist Medical Practitioners | 11 | - | - | - | N | N |
| Cho (58) | USA | Recording (video, audio, stopwatch) | First referral level | Specialist Medical Practitioners | 6.12 | - | - | - | N | N |
|  | USA | Recording (video, audio, stopwatch) | First referral level | Specialist Medical Practitioners | 7.53 | - | - | - | N | N |
|  | USA | Recording (video, audio, stopwatch) | First referral level | Specialist Medical Practitioners | 9.39 | - | - | - | N | N |
|  | USA | Recording (video, audio, stopwatch) | First referral level | Specialist Medical Practitioners | 4.7 | - | - | - | N | N |
|  | USA | Recording (video, audio, stopwatch) | First referral level | Specialist Medical Practitioners | 5.96 | - | - | - | N | N |
|  | USA | Recording (video, audio, stopwatch) | First referral level | Specialist Medical Practitioners | 7.99 | - | - | - | N | N |
|  | USA | Recording (video, audio, stopwatch) | First referral level | Specialist Medical Practitioners | 9.1 | - | - | - | N | N |
|  | USA | Recording (video, audio, stopwatch) | First referral level | Specialist Medical Practitioners | 7.54 | - | - | - | N | N |
|  | USA | Recording (video, audio, stopwatch) | First referral level | Specialist Medical Practitioners | 10.82 | - | - | - | N | N |
|  | USA | Recording (video, audio, stopwatch) | First referral level | Specialist Medical Practitioners | 6.1 | - | - | - | N | N |
|  | USA | Recording (video, audio, stopwatch) | First referral level | Specialist Medical Practitioners | 8.98 | - | - | - | N | N |
|  | USA | Recording (video, audio, stopwatch) | First referral level | Specialist Medical Practitioners | 8.66 | - | - | - | N | N |
| Ciocănel (59) | Romania | Self-reported/questionnaire | General outpatient services | Traditional and Complementary Medicine Professionals |  | NA | N/A | - | N | N |
|  | Romania | Self-reported/questionnaire | General outpatient services | Traditional and Complementary Medicine Professionals |  | NA | N/A | - | N | N |
| Compère (60) | France | Recording (video, audio, stopwatch) | First referral level | Specialist Medical Practitioners | 11.2 | - | - | SD, 5.8 | N | N |
|  | France | Recording (video, audio, stopwatch) | First referral level | Specialist Medical Practitioners | 8.35 | - | - | SD, 2.34 | N | N |
|  | France | Recording (video, audio, stopwatch) | First referral level | Specialist Medical Practitioners | 9.97 | - | - | SD, 4.83 | N | N |
|  | France | Recording (video, audio, stopwatch) | First referral level | Specialist Medical Practitioners | 12.75 | - | - | SD, 6.54 | N | N |
|  | France | Recording (video, audio, stopwatch) | First referral level | Specialist Medical Practitioners | 14.34 | - | - | SD, 6.73 | N | N |
|  | France | Recording (video, audio, stopwatch) | First referral level | Specialist Medical Practitioners | 12.29 | - | - | SD, 6.59 | N | N |
|  | France | Recording (video, audio, stopwatch) | First referral level | Specialist Medical Practitioners | 10.37 | - | - | SD, 4.94 | N | N |
|  | France | Recording (video, audio, stopwatch) | First referral level | Specialist Medical Practitioners | 8.65 | - | - | SD, 3.75 | N | N |
|  | France | Recording (video, audio, stopwatch) | First referral level | Specialist Medical Practitioners | 11.4 | - | - | SD, 5.39 | N | N |
|  | France | Recording (video, audio, stopwatch) | First referral level | Specialist Medical Practitioners | 16.18 | - | - | SD, 6.42 | N | N |
|  | France | Recording (video, audio, stopwatch) | First referral level | Specialist Medical Practitioners | 20 | - | - | SD, 5.31 | N | N |
|  | France | Recording (video, audio, stopwatch) | First referral level | Specialist Medical Practitioners | 11.06 | - | - | SD, 5.79 | N | N |
|  | France | Recording (video, audio, stopwatch) | First referral level | Specialist Medical Practitioners | 12.7 | - | - | SD, 5.81 | N | N |
|  | France | Recording (video, audio, stopwatch) | First referral level | Specialist Medical Practitioners | 11.04 | - | - | SD, 6 | N | N |
|  | France | Recording (video, audio, stopwatch) | First referral level | Specialist Medical Practitioners | 11.67 | - | - | SD, 5.53 | N | N |
|  | France | Recording (video, audio, stopwatch) | First referral level | Specialist Medical Practitioners | 11.12 | - | - | SD, 4.93 | N | N |
|  | France | Recording (video, audio, stopwatch) | First referral level | Specialist Medical Practitioners | 8.59 | - | - | SD, 5.39 | N | N |
|  | France | Recording (video, audio, stopwatch) | First referral level | Specialist Medical Practitioners | 10.36 | - | - | SD, 5.22 | N | N |
|  | France | Recording (video, audio, stopwatch) | First referral level | Specialist Medical Practitioners | 12.81 | - | - | SD, 5.74 | N | N |
|  | France | Recording (video, audio, stopwatch) | First referral level | Specialist Medical Practitioners | 14.44 | - | - | SD, 6.39 | N | N |
|  | France | Recording (video, audio, stopwatch) | First referral level | Specialist Medical Practitioners | 16.81 | - | - | SD, 6.57 | N | N |
|  | France | Recording (video, audio, stopwatch) | First referral level | Specialist Medical Practitioners | 8.41 | - | - | SD, 3.54 | N | N |
|  | France | Recording (video, audio, stopwatch) | First referral level | Specialist Medical Practitioners | 9.83 | - | - | SD, 3.98 | N | N |
|  | France | Recording (video, audio, stopwatch) | First referral level | Specialist Medical Practitioners | 13.2 | - | - | SD, 6.39 | N | N |
|  | France | Recording (video, audio, stopwatch) | First referral level | Specialist Medical Practitioners | 16.05 | - | - | SD, 5.83 | N | N |
|  | France | Recording (video, audio, stopwatch) | First referral level | Specialist Medical Practitioners | 21.06 | - | - | SD, 6.61 | N | N |
|  | France | Recording (video, audio, stopwatch) | First referral level | Specialist Medical Practitioners | 11.43 | - | - | SD, 6.77 | N | N |
|  | France | Recording (video, audio, stopwatch) | First referral level | Specialist Medical Practitioners | 12.73 | - | - | SD, 6.24 | N | N |
|  | France | Recording (video, audio, stopwatch) | First referral level | Specialist Medical Practitioners | 12.78 | - | - | SD, 5.23 | N | N |
|  | France | Recording (video, audio, stopwatch) | First referral level | Specialist Medical Practitioners | 15.97 | - | - | SD, 6.62 | N | N |
|  | France | Recording (video, audio, stopwatch) | First referral level | Specialist Medical Practitioners | 9.43 | - | - | SD, 4.67 | N | N |
|  | France | Recording (video, audio, stopwatch) | First referral level | Specialist Medical Practitioners | 18.46 | - | - | SD, 4.42 | N | N |
|  | France | Recording (video, audio, stopwatch) | First referral level | Specialist Medical Practitioners | 11.01 | - | - | SD, 5.39 | N | N |
|  | France | Recording (video, audio, stopwatch) | First referral level | Specialist Medical Practitioners | 9.66 | - | - | SD, 3.75 | N | N |
|  | France | Recording (video, audio, stopwatch) | First referral level | Specialist Medical Practitioners | 10.03 | - | - | SD, 4.71 | N | N |
|  | France | Recording (video, audio, stopwatch) | First referral level | Specialist Medical Practitioners | 8.76 | - | - | SD, 4.43 | N | N |
|  | France | Recording (video, audio, stopwatch) | First referral level | Specialist Medical Practitioners | 8.8 | - | - | SD, 3.11 | N | N |
|  | France | Recording (video, audio, stopwatch) | First referral level | Specialist Medical Practitioners | 11.51 | - | - | SD, 5.9 | N | N |
|  | France | Recording (video, audio, stopwatch) | First referral level | Specialist Medical Practitioners | 9.61 | - | - | SD, 5.15 | N | N |
|  | France | Recording (video, audio, stopwatch) | First referral level | Specialist Medical Practitioners | 10.58 | - | - | SD, 4.66 | N | N |
|  | France | Recording (video, audio, stopwatch) | First referral level | Specialist Medical Practitioners | 11.91 | - | - | SD, 4.5 | N | N |
|  | France | Recording (video, audio, stopwatch) | First referral level | Specialist Medical Practitioners | 17.29 | - | - | SD, 10.58 | N | N |
|  | France | Recording (video, audio, stopwatch) | First referral level | Specialist Medical Practitioners | 14.22 | - | - | SD, 6.26 | N | N |
|  | France | Recording (video, audio, stopwatch) | First referral level | Specialist Medical Practitioners | 15.95 | - | - | SD, 13.1 | N | N |
|  | France | Recording (video, audio, stopwatch) | First referral level | Specialist Medical Practitioners | 10.3 | - | - | SD, 5.06 | N | N |
|  | France | Recording (video, audio, stopwatch) | First referral level | Specialist Medical Practitioners | 10.74 | - | - | SD, 6.28 | N | N |
|  | France | Recording (video, audio, stopwatch) | First referral level | Specialist Medical Practitioners | 17.08 | - | - | SD, 5.24 | N | N |
|  | France | Recording (video, audio, stopwatch) | First referral level | Specialist Medical Practitioners | 9.71 | - | - | SD, 4.43 | N | N |
|  | France | Recording (video, audio, stopwatch) | First referral level | Specialist Medical Practitioners | 13.12 | - | - | SD, 7.1 | N | N |
|  | France | Recording (video, audio, stopwatch) | First referral level | Specialist Medical Practitioners | 18.52 | - | - | SD, 4.83 | N | N |
|  | France | Recording (video, audio, stopwatch) | First referral level | Specialist Medical Practitioners | 11.17 | - | - | SD, 5.87 | N | N |
|  | France | Recording (video, audio, stopwatch) | First referral level | Specialist Medical Practitioners | 12.32 | - | - | SD, 4.59 | N | N |
|  | France | Recording (video, audio, stopwatch) | First referral level | Specialist Medical Practitioners | 10.97 | - | - | SD, 5.77 | N | N |
|  | France | Recording (video, audio, stopwatch) | First referral level | Specialist Medical Practitioners | 12.34 | - | - | SD, 6.56 | N | N |
|  | France | Recording (video, audio, stopwatch) | First referral level | Specialist Medical Practitioners | 14.5 | - | - | SD, 2.12 | N | N |
|  | France | Recording (video, audio, stopwatch) | First referral level | Specialist Medical Practitioners | 8.56 | - | - | SD, 3.45 | N | N |
|  | France | Recording (video, audio, stopwatch) | First referral level | Specialist Medical Practitioners | 10.41 | - | - | SD, 3.79 | N | N |
|  | France | Recording (video, audio, stopwatch) | First referral level | Specialist Medical Practitioners | 10.94 | - | - | SD, 4.85 | N | N |
|  | France | Recording (video, audio, stopwatch) | First referral level | Specialist Medical Practitioners | 16 | - | - | SD, 6.92 | N | N |
|  | France | Recording (video, audio, stopwatch) | First referral level | Specialist Medical Practitioners | 10.27 | - | - | SD, 5.06 | N | N |
|  | France | Recording (video, audio, stopwatch) | First referral level | Specialist Medical Practitioners | 14 | - | - | SD, 8.09 | N | N |
|  | France | Recording (video, audio, stopwatch) | First referral level | Specialist Medical Practitioners | 12.4 | - | - | SD, 7.89 | N | N |
| Conley (61) | USA | Software tracking | First referral level | Specialist Medical Practitioners | 10.2 | - | - | SD, 9.4 | N | N |
|  | USA | Software tracking | First referral level | Specialist Medical Practitioners | 18.1 | - | - | SD, 15.4 | N | N |
|  | USA | Software tracking | First referral level | Specialist Medical Practitioners | 10.9 | - | - | SD, 8.5 | N | N |
|  | USA | Software tracking | First referral level | Specialist Medical Practitioners | 7.1 | - | - | SD, 5.5 | N | N |
|  | USA | Software tracking | First referral level | Specialist Medical Practitioners | 10.7 | - | - | SD, 9.7 | N | N |
|  | USA | Software tracking | First referral level | Specialist Medical Practitioners | 20.4 | - | - | SD, 18 | N | N |
|  | USA | Software tracking | First referral level | Specialist Medical Practitioners | 38.2 | - | - | SD, 28.9 | N | N |
|  | USA | Software tracking | First referral level | Specialist Medical Practitioners | 22.5 | - | - | SD, 14.8 | N | N |
|  | USA | Software tracking | First referral level | Specialist Medical Practitioners | 13.6 | - | - | SD, 10.8 | N | N |
|  | USA | Software tracking | First referral level | Specialist Medical Practitioners | 12.7 | - | - | SD, 10.2 | N | N |
| Corn (62) | USA | Recording (video, audio, stopwatch) | Community-based services | Pharmacists | 12.7 | - | - | SD, 3 | N | N |
| Crocker-Buque (63) | England | Self-reported/questionnaire | General outpatient services | General Medical Practitioners | 15.9 | - | - | - | N | N |
|  | England | Self-reported/questionnaire | General outpatient services | General Medical Practitioners | 10.4 | - | - | - | N | N |
| Crosbie (64) | Republic of Ireland | Software tracking | General outpatient services | General Medical Practitioners | 14.53 | - | - | - | N | N |
|  | Republic of Ireland | Software tracking | General outpatient services | General Medical Practitioners | 15.42 | - | - | - | N | N |
|  | Republic of Ireland | Software tracking | General outpatient services | General Medical Practitioners | 14.36 | - | - | - | N | N |
|  | Republic of Ireland | Software tracking | General outpatient services | General Medical Practitioners | 14.59 | - | - | - | N | N |
| Cui (65) | China | Existing databases or guidelines | First referral level | Specialist Medical Practitioners |  | 17 | - | - | Y | Y |
|  | China | Existing databases or guidelines | First referral level | Specialist Medical Practitioners |  | 18 | - | - | Y | N |
|  | China | Existing databases or guidelines | First referral level | Specialist Medical Practitioners |  | 13 | - | - | Y | N |
|  | China | Existing databases or guidelines | First referral level | Specialist Medical Practitioners |  | 16 | - | - | Y | N |
|  | China | Existing databases or guidelines | First referral level | Specialist Medical Practitioners |  | 21 | - | - | Y | N |
|  | China | Existing databases or guidelines | First referral level | Specialist Medical Practitioners |  | 21 | - | - | Y | N |
|  | China | Existing databases or guidelines | First referral level | Specialist Medical Practitioners |  | 26 | - | - | Y | Y |
| Dabaghzadeh (66) | Iran | Recording (video, audio, stopwatch) | Community-based services | Pharmacists | 2.8 | - | - | SD, 1 | N | N |
| Dakroub (67) | Lebanon | Other, specify: | First referral level | Health Professionals not elsewhere classified | 7.3 | - | - | SD, 6.8 | N | N |
|  | Lebanon | Other, specify: | First referral level | Health Professionals not elsewhere classified | 10.6 | - | - | SD, 9.8 | N | N |
|  | Lebanon | Other, specify: | First referral level | Health Professionals not elsewhere classified | 6.6 | - | - | SD, 5.7 | N | N |
|  | Lebanon | Other, specify: | First referral level | Health Professionals not elsewhere classified | 10.6 | - | - | SD, 11.5 | N | N |
|  | Lebanon | Other, specify: | First referral level | Health Professionals not elsewhere classified | 11.9 | - | - | SD, 12.5 | N | N |
|  | Lebanon | Other, specify: | First referral level | Health Professionals not elsewhere classified | 9.8 | - | - | SD, 10.7 | N | N |
|  | Lebanon | Other, specify: | First referral level | Specialist Medical Practitioners | 17 | - | - | SD, 13.8 | N | N |
|  | Lebanon | Other, specify: | First referral level | Specialist Medical Practitioners | 18.8 | - | - | SD, 14.3 | N | N |
|  | Lebanon | Other, specify: | First referral level | Specialist Medical Practitioners | 16.6 | - | - | SD, 13.7 | N | N |
|  | Lebanon | Other, specify: | First referral level | Specialist Medical Practitioners | 25.9 | - | - | SD, 21.6 | N | N |
|  | Lebanon | Other, specify: | First referral level | Specialist Medical Practitioners | 30.2 | - | - | SD, 23.6 | N | N |
|  | Lebanon | Other, specify: | First referral level | Specialist Medical Practitioners | 23.2 | - | - | SD, 19.7 | N | N |
| Daniel (68) | USA | Recording (video, audio, stopwatch) | First referral level | Nursing Professionals | 8.7 | - | - | - | N | N |
|  | USA | Recording (video, audio, stopwatch) | First referral level | Nursing Professionals | 9.1 | - | - | - | N | N |
|  | USA | Recording (video, audio, stopwatch) | First referral level | Nursing Professionals | 0.3 | - | - | - | N | N |
|  | USA | Recording (video, audio, stopwatch) | First referral level | Nursing Professionals | 0.5 | - | - | - | N | N |
| Darlison (69) | Australia | Self-reported/questionnaire | General outpatient services | General Medical Practitioners |  | 11.42 | 7.95 (IQR) | - | N | N |
|  | Australia | Self-reported/questionnaire | General outpatient services | General Medical Practitioners |  | 10.51 | 8.08 (IQR) | - | N | N |
|  | Australia | Self-reported/questionnaire | General outpatient services | General Medical Practitioners |  | 11.44 | 8.00 (IQR) | - | N | N |
|  | Australia | Self-reported/questionnaire | General outpatient services | General Medical Practitioners |  | 10.57 | 7.58 (IQR) | - | N | N |
|  | Australia | Self-reported/questionnaire | General outpatient services | General Medical Practitioners |  | 10.8 | 7.98 (IQR) | - | N | N |
|  | Australia | Self-reported/questionnaire | General outpatient services | General Medical Practitioners |  | 13.2 | 8.01 (IQR) | - | N | N |
|  | Australia | Self-reported/questionnaire | General outpatient services | General Medical Practitioners |  | 8.18 | 5.04 (IQR) | - | N | N |
|  | Australia | Self-reported/questionnaire | General outpatient services | General Medical Practitioners |  | 12.13 | 8.68 (IQR) | - | N | N |
|  | Australia | Self-reported/questionnaire | General outpatient services | General Medical Practitioners |  | 11.51 | 8.00 (IQR) | - | N | N |
|  | Australia | Self-reported/questionnaire | General outpatient services | General Medical Practitioners |  | 11.12 | 7.81 (IQR) | - | N | N |
|  | Australia | Self-reported/questionnaire | General outpatient services | General Medical Practitioners |  | 12.2 | 8.42 (IQR) | - | N | N |
|  | Australia | Self-reported/questionnaire | General outpatient services | General Medical Practitioners |  | 11.2 | 7.46 (IQR) | - | N | N |
|  | Australia | Self-reported/questionnaire | General outpatient services | General Medical Practitioners |  | 11.57 | 7.60 (IQR) | - | N | N |
|  | Australia | Self-reported/questionnaire | General outpatient services | General Medical Practitioners |  | 11.75 | 8.13 (IQR) | - | N | N |
|  | Australia | Self-reported/questionnaire | General outpatient services | General Medical Practitioners |  | 10.87 | 7.57 (IQR) | - | N | N |
|  | Australia | Self-reported/questionnaire | General outpatient services | General Medical Practitioners |  | 7.61 | 7.61 (IQR) | - | N | N |
| Das (70) | Bangladesh | Self-reported/questionnaire | First referral level | General Medical Practitioners | 9.1 | - | - | SD, 4.44 | N | N |
|  | Bangladesh | Self-reported/questionnaire | First referral level | General Medical Practitioners | 8.16 | - | - | SD, 3.84 | N | N |
|  | Bangladesh | Self-reported/questionnaire | First referral level | General Medical Practitioners | 9.76 | - | - | SD, 4.71 | N | N |
|  | Bangladesh | Self-reported/questionnaire | First referral level | General Medical Practitioners | 10.27 | - | - | SD, 4.3 | N | N |
|  | Bangladesh | Self-reported/questionnaire | First referral level | General Medical Practitioners | 8.13 | - | - | SD, 4.33 | N | N |
|  | Bangladesh | Self-reported/questionnaire | First referral level | General Medical Practitioners | 8.84 | - | - | SD, 4.72 | N | N |
|  | Bangladesh | Self-reported/questionnaire | First referral level | General Medical Practitioners | 9.29 | - | - | SD, 4.23 | N | N |
|  | Bangladesh | Self-reported/questionnaire | First referral level | General Medical Practitioners | 9.07 | - | - | SD, 4.36 | N | N |
|  | Bangladesh | Self-reported/questionnaire | First referral level | General Medical Practitioners | 9.12 | - | - | SD, 4.5 | N | N |
|  | Bangladesh | Self-reported/questionnaire | First referral level | General Medical Practitioners | 10.35 | - | - | SD, 4.6 | N | N |
|  | Bangladesh | Self-reported/questionnaire | First referral level | General Medical Practitioners | 8.55 | - | - | SD, 4.26 | N | N |
|  | Bangladesh | Self-reported/questionnaire | First referral level | General Medical Practitioners | 10.86 | - | - | SD, 4.51 | N | N |
|  | Bangladesh | Self-reported/questionnaire | First referral level | General Medical Practitioners | 8.54 | - | - | SD, 4.28 | N | N |
|  | Bangladesh | Self-reported/questionnaire | First referral level | General Medical Practitioners | 9.07 | - | - | SD, 4.5 | N | N |
|  | Bangladesh | Self-reported/questionnaire | First referral level | General Medical Practitioners | 9.35 | - | - | SD, 3.98 | N | N |
|  | Bangladesh | Self-reported/questionnaire | First referral level | General Medical Practitioners | 9.48 | - | - | SD, 4.5 | N | N |
|  | Bangladesh | Self-reported/questionnaire | First referral level | General Medical Practitioners | 8.41 | - | - | SD, 4.27 | N | N |
|  | Bangladesh | Self-reported/questionnaire | First referral level | General Medical Practitioners | 10.25 | - | - | SD, 4.42 | N | N |
|  | Bangladesh | Self-reported/questionnaire | First referral level | General Medical Practitioners | 8.68 | - | - | SD, 4.38 | N | N |
| Dessie (71) | Ethiopia |  | First referral level | Pharmacists | 2.11 | - | - | - | N | N |
|  | Ethiopia |  | First referral level | Pharmacists | 2.25 | - | - | - | N | N |
| DeWyer (72) | Uganda | Software tracking | Second referral level (and above) | Specialist Medical Practitioners | 30 | - | - | - | Y | N |
| Dingwall (73) | Australia | Existing databases or guidelines | General outpatient services | General Medical Practitioners | 15.2 | - | - | - | N | N |
|  | Australia | Existing databases or guidelines | General outpatient services | General Medical Practitioners | 14.5 | - | - | - | N | N |
| Donahue (74) | USA | Software tracking | First referral level | Specialist Medical Practitioners |  | NA | For 81% (13/16) of the patients, encounters were <= 10 minutes. The youngest patients (aged 3-5) ranged from 5 to 35 minutes. | - | Y | N |
| Driever (75) | Netherlands | Recording (video, audio, stopwatch) | Second referral level (and above) |  | 16.5 |  |  | SD, 5.5 |  | N |
|  | Netherlands | Recording (video, audio, stopwatch) | Second referral level (and above) |  | 15.2 |  |  | SD, 6.8 |  | N |
|  | Netherlands | Recording (video, audio, stopwatch) | Second referral level (and above) |  | 18.2 |  |  | SD, 9 |  | N |
|  | Netherlands | Recording (video, audio, stopwatch) | Second referral level (and above) |  | 13 |  |  | SD, 6.2 |  | N |
|  | Netherlands | Recording (video, audio, stopwatch) | Second referral level (and above) |  | 15.8 |  |  | SD, 7.3 |  | N |
|  | Netherlands | Recording (video, audio, stopwatch) | Second referral level (and above) |  | 16.3 |  |  | SD, 5.5 |  | N |
|  | Netherlands | Recording (video, audio, stopwatch) | Second referral level (and above) |  | 24.1 |  |  | SD, 9.3 |  | N |
|  | Netherlands | Recording (video, audio, stopwatch) | Second referral level (and above) |  | 27 |  |  | SD, 9.3 |  | N |
|  | Netherlands | Recording (video, audio, stopwatch) | Second referral level (and above) |  | 21.6 |  |  | SD, 11.3 |  | N |
|  | Netherlands | Recording (video, audio, stopwatch) | Second referral level (and above) |  | 9.1 |  |  | SD, 2.9 |  | N |
|  | Netherlands | Recording (video, audio, stopwatch) | Second referral level (and above) |  | 17 |  |  | SD, 10.6 |  | N |
|  | Netherlands | Recording (video, audio, stopwatch) | Second referral level (and above) |  | 8.1 |  |  | SD, 4 |  | N |
|  | Netherlands | Recording (video, audio, stopwatch) | Second referral level (and above) |  | 14.1 |  |  | SD, 7.8 |  | N |
|  | Netherlands | Recording (video, audio, stopwatch) | Second referral level (and above) |  | 14.3 |  |  | SD, 7.8 |  | N |
|  | Netherlands | Recording (video, audio, stopwatch) | Second referral level (and above) |  | 8.2 |  |  | SD, 3.9 |  | N |
|  | Netherlands | Recording (video, audio, stopwatch) | Second referral level (and above) |  | 9.5 |  |  | SD, 5.5 |  | N |
|  | Netherlands | Recording (video, audio, stopwatch) | Second referral level (and above) |  | 11.6 |  |  | SD, 9 |  | N |
|  | Netherlands | Recording (video, audio, stopwatch) | Second referral level (and above) |  | 9 |  |  | SD, 4.5 |  | N |
|  | Netherlands | Recording (video, audio, stopwatch) | Second referral level (and above) |  | 15.1 |  |  | SD, 9 |  | N |
| Dubois (76) | Switzerland | Self-reported/questionnaire | General outpatient services | Traditional and Complementary Medicine Professionals | 55.1 | - | - | SD, 15.8 | N | N |
|  | Switzerland | Self-reported/questionnaire | General outpatient services | Traditional and Complementary Medicine Professionals | 45.5 | - | - | SD, 6.5 | N | N |
|  | Switzerland | Self-reported/questionnaire | General outpatient services | Traditional and Complementary Medicine Professionals | 72 | - | - | SD, 16.7 | N | N |
|  | Switzerland | Self-reported/questionnaire | General outpatient services | Traditional and Complementary Medicine Professionals | 61.6 | - | - | SD, 12.5 | N | N |
| Ebbers (77) | Netherlands | Software tracking | Second referral level (and above) | Specialist Medical Practitioners |  | 52.38 | Initial oncological consultation included time spent on EHR related tasks for 40% of the total time. | - | N | N |
|  | Netherlands | Software tracking | Second referral level (and above) | Specialist Medical Practitioners | 45.56 | 54.27 | Initial oncological consultation included time spent on EHR related tasks for 40% of the total time. | SD, 12.25 | N | N |
|  | Netherlands | Software tracking | Second referral level (and above) | Specialist Medical Practitioners |  | 9.54 | Follow up consultation included time spent on EHR related tasks for 30.7% of the total time. | - | N | N |
|  | Netherlands | Software tracking | Second referral level (and above) | Specialist Medical Practitioners | 13.18 | 11.55 | Follow up consultation included time spent on EHR related tasks for 30.7% of the total time. | SD, 6.34 | N | N |
| Ekberg (78) | Australia | Recording (video, audio, stopwatch) | Other, specify: | Audiologists and Speech Therapists | 57.8 | - | - | SD, 20.3 | N | N |
| Ellington (79) | USA | Recording (video, audio, stopwatch) | General outpatient services | Nursing Professionals | 40.2 | - | - | - | N | N |
| Elliot (80) | UK | Other, specify: | General outpatient services | Nursing Professionals | 7 | - | - | - | Y | N |
| Ellis (81) | Canada | Self-reported/questionnaire | First referral level | Health Professionals not elsewhere classified |  | 10 | - | - | N | N |
| Elrggal (82) | Saudi Arabia | Self-reported/questionnaire | Community-based services | Pharmacists |  | NA | Time in minutes: up to 5 (40, 40%); More than 5 (60, 60%) | - | N | N |
| Ewelukwa (83) | USA | Electronic health records | General outpatient services | General Medical Practitioners | 31 |  |  | SD, 18 | N | N |
|  | USA | Electronic health records | General outpatient services | General Medical Practitioners | 18 |  |  | SD, 7 | N | N |
| Fang (84) | USA | Recording (video, audio, stopwatch) | Other, specify: | Dentists | 155.06 |  |  | SD, 35.63 | N | N |
| Fatigante (85) | Italy | Recording (video, audio, stopwatch) | First referral level | Specialist Medical Practitioners | 27.5 | - | - | - | N | N |
| Fesler (86) | USA | Software tracking | Second referral level (and above) | Specialist Medical Practitioners | 15.7 | - | - | SD, 10.4 | Y | N |
| Fieux (87) | France | Software tracking | First referral level | Specialist Medical Practitioners | 9 | - | - | - | Y | Y |
| Fisher (88) | Australia | Self-reported/questionnaire | Other, specify: | Traditional and Complementary Medicine Professionals |  | 72 | - | - | Y | N |
|  | Australia | Self-reported/questionnaire | Other, specify: | Traditional and Complementary Medicine Professionals |  | 43 | - | - | Y | N |
|  | Australia | Self-reported/questionnaire | Other, specify: | Traditional and Complementary Medicine Professionals |  | 75 | - | - | Y | N |
|  | Australia | Self-reported/questionnaire | Other, specify: | Traditional and Complementary Medicine Professionals |  | 45 | - | - | Y | N |
|  | Australia | Self-reported/questionnaire | Other, specify: | Traditional and Complementary Medicine Professionals |  | 60 | - | - | Y | N |
|  | Australia | Self-reported/questionnaire | Other, specify: | Traditional and Complementary Medicine Professionals |  | 30 | - | - | Y | N |
| Folayan (89) | Nigeria | Self-reported/questionnaire | First referral level | Dentists | 29.76 | - | - | SD, 10.16 | N | N |
|  | Nigeria | Self-reported/questionnaire | First referral level | Dentists | 41.2 | - | - | SD, 14.31 | N | N |
|  | Nigeria | Self-reported/questionnaire | First referral level | Dentists | 46.14 | - | - | SD, 24.05 | N | N |
|  | Nigeria | Self-reported/questionnaire | First referral level | Dentists | 37.43 | - | - | SD, 10.61 | N | N |
|  | Nigeria | Self-reported/questionnaire | First referral level | Dentists | 142.87 | - | - | SD, 80 | N | N |
|  | Nigeria | Self-reported/questionnaire | First referral level | Dentists | 43.28 | - | - | SD, 24.64 | N | N |
|  | Nigeria | Self-reported/questionnaire | First referral level | Dentists | 115.8 | - | - | SD, 34.2 | N | N |
|  | Nigeria | Self-reported/questionnaire | First referral level | Dentists | 34.39 | - | - | SD, 10.82 | N | N |
|  | Nigeria | Self-reported/questionnaire | First referral level | Dentists | 66.13 | - | - | SD, 26.88 | N | N |
|  | Nigeria | Self-reported/questionnaire | First referral level | Dentists | 64.17 | - | - | SD, 30.56 | N | N |
|  | Nigeria | Self-reported/questionnaire | First referral level | Dentists | 37.39 | - | - | SD, 5.73 | N | N |
|  | Nigeria | Self-reported/questionnaire | First referral level | Dentists | 94.5 | - | - | SD, 6.36 | N | N |
|  | Nigeria | Self-reported/questionnaire | First referral level | Dentists | 21.4 | - | - | SD, 2.07 | N | N |
|  | Nigeria | Self-reported/questionnaire | First referral level | Dentists | 110 | - | - | SD, 7.07 | N | N |
|  | Nigeria | Self-reported/questionnaire | First referral level | Dentists | 31.72 | - | - | SD, 10.62 | N | N |
|  | Nigeria | Self-reported/questionnaire | First referral level | Dentists | 49.89 | - | - | SD, 22.53 | N | N |
|  | Nigeria | Self-reported/questionnaire | First referral level | Dentists | 54.46 | - | - | SD, 27.67 | N | N |
|  | Nigeria | Self-reported/questionnaire | First referral level | Dentists | 37.41 | - | - | SD, 13.52 | N | N |
|  | Nigeria | Self-reported/questionnaire | First referral level | Dentists | 137.18 | - | - | SD, 76.55 | N | N |
|  | Nigeria | Self-reported/questionnaire | First referral level | Dentists | 40.05 | - | - | SD, 24.04 | N | N |
|  | Nigeria | Self-reported/questionnaire | First referral level | Dentists | 114.14 | - | - | SD, 28.22 | N | N |
| Ensign (90) | USA | Self-reported/questionnaire | Community-based services | General Medical Practitioners | 25 |  |  | - | N | N |
|  | USA | Self-reported/questionnaire | Community-based services | General Medical Practitioners | 28.5 |  |  | - | N | N |
| Frost (91) | USA | Recording (video, audio, stopwatch) | Community-based services | Pharmacists | 17.9 |  |  | - | N | N |
|  | USA | Recording (video, audio, stopwatch) | Community-based services | Pharmacists | 14.1 |  |  | - | N | N |
| Frye (92) | USA | Electronic health records | Second referral level (and above) | Specialist Medical Practitioners | 87.3 | - | - | - | N | N |
|  | USA | Electronic health records | Second referral level (and above) | Specialist Medical Practitioners | 82.9 | - | - | - | N | N |
| Gaffney (93) | USA | Existing databases or guidelines | First referral level | Specialist Medical Practitioners | 22.2 | - | - | - | N | N |
|  | USA | Existing databases or guidelines | First referral level | Specialist Medical Practitioners | 15.4 | - | - | - | N | N |
| Galvão (94) | Brazil | Self-reported/questionnaire | General outpatient services | General Medical Practitioners | 15 | - | - | - | N | N |
| Gan (95) | USA | Software tracking | First referral level | Specialist Medical Practitioners |  | 20 | - | - | Y | Y |
| Gao (96) | China | Self-reported/questionnaire | General outpatient services | General Medical Practitioners | 2.8 | - | - | SD, 3.2 | N | N |
| Garcia-Layana (97) | Spain | Self-reported/questionnaire | First referral level | Specialist Medical Practitioners |  | 15 | - | SD, - | N | N |
| Garg (98) | USA | Software tracking | First referral level | Specialist Medical Practitioners | 35 | - | - | SD, - | Y | Y |
|  | Australia | Recording (video, audio, stopwatch) | General outpatient services | General Medical Practitioners | 13 | - | IQR: 9-18 mins | SD, - | N | N |
| Gebramariam (99) | Ethiopia | Recording (video, audio, stopwatch) | First referral level | Pharmacists | 5.12 | - | - | SD, - | N | N |
|  | Ethiopia | Recording (video, audio, stopwatch) | First referral level | Pharmacists | 6.13 | - | - | SD, - | N | N |
|  | Ethiopia | Recording (video, audio, stopwatch) | First referral level | Pharmacists | 6.35 | - | - | SD, - | N | N |
|  | Ethiopia | Recording (video, audio, stopwatch) | First referral level | Pharmacists | 4.6 | - | - | SD, - | N | N |
|  | Ethiopia | Recording (video, audio, stopwatch) | First referral level | Pharmacists | 5.25 | - | - | SD, - | N | N |
|  | Ethiopia | Recording (video, audio, stopwatch) | First referral level | Pharmacists | 4.9 | - | - | SD, - | N | N |
|  | Ethiopia | Recording (video, audio, stopwatch) | First referral level | Pharmacists | 4.2 | - | - | SD, - | N | N |
|  | Ethiopia | Recording (video, audio, stopwatch) | First referral level | Pharmacists | 4.41 | - | - | SD, - | N | N |
| Geessink (100) | Netherlands | Recording (video, audio, stopwatch) | First referral level | Specialist Medical Practitioners | 21.6 | - | - | SD, 8.1 | N | N |
| Gidlow (101) | UK | Recording (video, audio, stopwatch) | General outpatient services | Health Care Assistants | NA | NA | <10 (10); 10.0-14.9 (19); 15.0-19.9 (32); 20.0-24.9 (19); 25.0-29.9 (15); 30.0-34.9 (1); 35.0-39.9 (4) | - | N | N |
|  | UK | Recording (video, audio, stopwatch) | General outpatient services | Health Care Assistants | NA | NA | <10 (0); 10.0-14.9 (10); 15.0-19.9 (50); 20.0-24.9 (21); 25.0-29.9 (12); 30.0-34.9 (2); 35.0-39.9 (5) | - | N | N |
| Glinkowski (102) | Poland | Electronic health records | First referral level | Specialist Medical Practitioners | 13.36 | - | - | SD, 8.63 | Y | Y |
| Goldstein (103) | USA | Electronic health records | First referral level | Specialist Medical Practitioners | 80.3 | - | - | SD, 45.4 | N | N |
|  | USA | Electronic health records | First referral level | Specialist Medical Practitioners | 69.7 | - | - | SD, 41.7 | N | N |
|  | USA | Electronic health records | First referral level | Specialist Medical Practitioners | 73.4 | - | - | SD, 40.9 | N | N |
|  | USA | Electronic health records | First referral level | Specialist Medical Practitioners | 99.4 | - | - | SD, 48.4 | N | N |
|  | USA | Electronic health records | First referral level | Specialist Medical Practitioners | 87.2 | - | - | SD, 49.2 | N | N |
|  | USA | Electronic health records | First referral level | Specialist Medical Practitioners | 71.6 | - | - | SD, 45.9 | N | N |
|  | USA | Electronic health records | First referral level | Specialist Medical Practitioners | 89.8 | - | - | SD, 47.8 | N | N |
|  | USA | Electronic health records | First referral level | Specialist Medical Practitioners | 104.3 | - | - | SD, 49.9 | N | N |
|  | USA | Electronic health records | First referral level | Specialist Medical Practitioners | 104.3 | - | - | SD, 57.8 | N | N |
|  | USA | Electronic health records | First referral level | Specialist Medical Practitioners | 86.5 | - | - | SD, 52 | N | N |
|  | USA | Electronic health records | First referral level | Specialist Medical Practitioners | 100.3 | - | - | SD, 47.1 | N | N |
|  | USA | Electronic health records | First referral level | Specialist Medical Practitioners | 129.1 | - | - | SD, 68.1 | N | N |
|  | USA | Electronic health records | First referral level | Specialist Medical Practitioners | 106.1 | - | - | SD, 52.4 | N | N |
|  | USA | Electronic health records | First referral level | Specialist Medical Practitioners | 88.3 | - | - | SD, 46.7 | N | N |
|  | USA | Electronic health records | First referral level | Specialist Medical Practitioners | 96.1 | - | - | SD, 48.6 | N | N |
|  | USA | Electronic health records | First referral level | Specialist Medical Practitioners | 123.2 | - | - | SD, 53.2 | N | N |
| Gopfert (104) | UK | Electronic health records | General outpatient services | General Medical Practitioners | 10.9 | - | - | SD, 7.8 | N | N |
|  | UK | Electronic health records | General outpatient services | General Medical Practitioners | 11 | - | - | SD, 7.8 | N | N |
|  | UK | Electronic health records | General outpatient services | General Medical Practitioners | 10.6 | - | - | SD, 7.4 | N | N |
|  | UK | Electronic health records | General outpatient services | General Medical Practitioners | 10.9 | - | - | SD, 7.6 | N | N |
|  | UK | Electronic health records | General outpatient services | General Medical Practitioners | 11.2 | - | - | SD, 7.7 | N | N |
|  | UK | Electronic health records | General outpatient services | General Medical Practitioners | 11.1 | - | - | SD, 7.6 | N | N |
|  | UK | Electronic health records | General outpatient services | General Medical Practitioners | 10.9 | - | - | SD, 7.7 | N | N |
|  | UK | Electronic health records | General outpatient services | General Medical Practitioners | 10.9 | - | - | SD, 7.9 | N | N |
|  | UK | Electronic health records | General outpatient services | General Medical Practitioners | 10.7 | - | - | SD, 8.9 | N | N |
|  | UK | Electronic health records | General outpatient services | General Medical Practitioners | 7.5 | - | - | SD, 7.5 | N | N |
|  | UK | Electronic health records | General outpatient services | General Medical Practitioners | 9.4 | - | - | SD, 9.4 | N | N |
|  | UK | Electronic health records | General outpatient services | General Medical Practitioners | 10.3 | - | - | SD, 7.7 | N | N |
|  | UK | Electronic health records | General outpatient services | General Medical Practitioners | 11.5 | - | - | SD, 7.8 | N | N |
|  | UK | Electronic health records | General outpatient services | General Medical Practitioners | 12.1 | - | - | SD, 8.9 | N | N |
|  | UK | Electronic health records | General outpatient services | General Medical Practitioners | 10.7 | - | - | SD, 7.9 | N | N |
|  | UK | Electronic health records | General outpatient services | General Medical Practitioners | 11 | - | - | SD, 7.6 | N | N |
|  | UK | Electronic health records | General outpatient services | General Medical Practitioners | 11 | - | - | SD, 8 | N | N |
|  | UK | Electronic health records | General outpatient services | General Medical Practitioners | 11.2 | - | - | SD, 7.9 | N | N |
|  | UK | Electronic health records | General outpatient services | General Medical Practitioners | 11 | - | - | SD, 7.8 | N | N |
|  | UK | Electronic health records | General outpatient services | General Medical Practitioners | 10.9 | - | - | SD, 7.8 | N | N |
|  | UK | Electronic health records | General outpatient services | General Medical Practitioners | 10.8 | - | - | SD, 7.8 | N | N |
|  | UK | Electronic health records | General outpatient services | General Medical Practitioners | 10.7 | - | - | SD, 7.6 | N | N |
|  | UK | Electronic health records | General outpatient services | General Medical Practitioners | 10.8 | - | - | SD, 7.3 | N | N |
|  | UK | Electronic health records | General outpatient services | General Medical Practitioners | 10.9 | - | - | SD, 7.5 | N | N |
|  | UK | Electronic health records | General outpatient services | General Medical Practitioners | 10.9 | - | - | SD, 7.7 | N | N |
|  | UK | Electronic health records | General outpatient services | General Medical Practitioners | 11 | - | - | SD, 8 | N | N |
|  | UK | Electronic health records | General outpatient services | General Medical Practitioners | 11 | - | - | SD, 8.2 | N | N |
|  | UK | Electronic health records | General outpatient services | General Medical Practitioners | 11.2 | - | - | SD, 8.9 | N | N |
|  | UK | Electronic health records | General outpatient services | General Medical Practitioners | 10.8 | - | - | SD, 7.4 | N | N |
|  | UK | Electronic health records | General outpatient services | General Medical Practitioners | 11 | - | - | SD, 8.1 | N | N |
|  | UK | Electronic health records | General outpatient services | General Medical Practitioners | 10.9 | - | - | SD, 8 | N | N |
|  | UK | Electronic health records | General outpatient services | General Medical Practitioners | 11.1 | - | - | SD, 8.3 | N | N |
| Gormley (105) | USA | Recording (video, audio, stopwatch) | General outpatient services | General Medical Practitioners | 13.19 | - | - | SD, 6.15 | N | N |
|  | USA | Recording (video, audio, stopwatch) | General outpatient services | General Medical Practitioners | 3.31 | - | - | SD, 6.14 | N | N |
|  | USA | Recording (video, audio, stopwatch) | General outpatient services | General Medical Practitioners | 13.17 | - | - | SD, 6.55 | N | N |
|  | USA | Recording (video, audio, stopwatch) | General outpatient services | General Medical Practitioners | 13.03 | - | - | SD, 5.71 | N | N |
| Grandizio (106) | USA | Software tracking | First referral level | Specialist Medical Practitioners | 7 | - | - | - | Y | N |
| Greenfield (107) | USA | Software tracking | First referral level | Specialist Medical Practitioners | 18.47 | - | - | SD, 9.2 | Y | Y |
|  | USA | Software tracking | First referral level | Specialist Medical Practitioners | 19.94 | - | - | SD, 10.8 | Y | Y |
|  | USA | Software tracking | First referral level | Specialist Medical Practitioners | 22.07 | - | - | SD, 12.4 | Y | Y |
|  | USA | Software tracking | First referral level | Specialist Medical Practitioners | 21.29 | - | - | SD, 11.35 | Y | Y |
|  | USA | Software tracking | First referral level | Specialist Medical Practitioners | 17.26 | - | - | SD, 9.2 | Y | Y |
|  | USA | Software tracking | First referral level | Specialist Medical Practitioners | 23.42 | - | - | SD, 12.26 | Y | Y |
|  | USA | Software tracking | First referral level | Specialist Medical Practitioners | 20.02 | - | - | SD, 10.82 | Y | Y |
| Gregório (108) | Portugal | Recording (video, audio, stopwatch) | Community-based services | Pharmacists | 3.98 | 3.1 | - | - | N | N |
|  | Portugal | Recording (video, audio, stopwatch) | Community-based services | Pharmacists | 4.52 |  | - | 95% CI, 4.2 - 5.23 | N | N |
|  | Portugal | Recording (video, audio, stopwatch) | Community-based services | Pharmacists | 3.17 |  | - | 95% CI, 2.34 - 3.59 | N | N |
|  | Portugal | Recording (video, audio, stopwatch) | Community-based services | Pharmacists | 2.34 | - | - | 95% CI, 3.03 - 3.59 | N | N |
|  | Portugal | Recording (video, audio, stopwatch) | Community-based services | Pharmacists | 4.09 | - | - | 95% CI, 3.19 - 4.59 | N | N |
| Gudeta (109) | Ethiopia | Recording (video, audio, stopwatch) | First referral level | Pharmacists | 0.819 | - | 49.13 seconds | - | N | N |
| Gunasekera (110) | Sri Lanka | Self-reported/questionnaire | First referral level | Specialist Medical Practitioners | NA | NA | <10 mins (8, 20%); 11-20 mins (24, 58%); 21-40 mins (9, 22%); >40 mins (0, 0%) | - | N | N |
|  | Sri Lanka | Self-reported/questionnaire | First referral level | Specialist Medical Practitioners | NA | NA | >10 mins (21, 52%); 10-30 mins (17, 41%); > 30 mins (3, 7%) | - | N | N |
|  | Sri Lanka | Self-reported/questionnaire | First referral level | Specialist Medical Practitioners | NA | NA | >5mins (12, 29%); 5-10 mins (23, 56%); > 10 mins (6, 15%) | - | N | N |
| Hafeez (111) | Pakistan | Self-reported/questionnaire | Second referral level (and above) | Specialist Medical Practitioners | 1.5 |  |  | - | N | N |
|  | Pakistan | Self-reported/questionnaire | Second referral level (and above) | Specialist Medical Practitioners | 2 |  |  | - | N | N |
| Hajizadeh (112) | Iran | - | First referral level | Specialist Medical Practitioners | 6.9 | 7 | - | - | N | N |
|  | Iran | - | First referral level | Specialist Medical Practitioners | 7 | - | - | - | N | N |
|  | Iran | - | First referral level | Specialist Medical Practitioners | 6.8 | - | - | - | N | N |
|  | Iran | - | First referral level | Specialist Medical Practitioners | 5.1 | - | - | - | N | N |
|  | Iran | - | First referral level | Specialist Medical Practitioners | 6.7 | - | - | - | N | N |
|  | Iran | - | First referral level | Specialist Medical Practitioners | 9.1 | - | - | - | N | N |
| Hallensleben (113) | Germany | Existing databases or guidelines | First referral level | Nursing Professionals | 44.8 | 40 | - | - | N | N |
|  | Germany | Existing databases or guidelines | First referral level | Nursing Professionals | 47.5 | 45.5 | - | - | N | N |
|  | Germany | Existing databases or guidelines | First referral level | Nursing Professionals | 38.3 | 30.4 | - | - | Y | Y |
|  | Germany | Existing databases or guidelines | First referral level | Nursing Professionals | 46.4 | 45.5 | - | - | Y | Y |
| Halloran (114) | USA | Self-reported/questionnaire | First referral level |  | NA | NA | This study has provided frequencies for ranges. Sharing these below for new patient (brackets are %):  <16 minutes 6 (5) 16–45 minutes 28 (24) 45–60 minutes 50 (43) >60 minutes 32 (28) Total average time spent with established patient follow-up visit: <16 minutes 11 (9) 16–45 minutes 75 (65) 45–60 minutes 16 (14) >60 minutes 2 (2) | - | N | N |
| Hallquist (115) | USA | Recording (video, audio, stopwatch) | Other, specify: | Specialist Medical Practitioners | 51.6 |  |  | SD, 20.6 | N | N |
|  | USA | Recording (video, audio, stopwatch) | Other, specify: | Specialist Medical Practitioners | 44.5 |  |  | SD, 6.7 | N | N |
| Halls (116) | UK | Self-reported/questionnaire | First referral level |  | 20 |  |  | - | N | N |
| Hammad (117) | Jordan | Recording (video, audio, stopwatch) | Community-based services | Pharmacists | 1.55 | - | - | - | N | N |
|  | Jordan |  | Community-based services | Pharmacists | NA | 2 | - | - | N | N |
| Hammersley (118) | UK | Recording (video, audio, stopwatch) | General outpatient services | General Medical Practitioners | 9.61 | 8.4 | - | SD, 4.53; 95% CI, 8.34 - 10.89 | N | N |
|  | UK | Recording (video, audio, stopwatch) | General outpatient services | General Medical Practitioners | 5.56 | 4.93 | - | SD, 2.72; 95% CI, 4.81 - 6.31 | Y | N |
|  | UK | Recording (video, audio, stopwatch) | General outpatient services | General Medical Practitioners | 5.94 | 5.42 | - | SD, 2.63; 95% CI, 5.15 - 6.73 | Y | N |
|  | UK | Software tracking | General outpatient services | General Medical Practitioners | NA | NA | Median time per patient, all appointments, is 67.5. Number of appointment activities per patientis 6.5. | - | Y | N |
|  | UK | Software tracking | General outpatient services | General Medical Practitioners | NA | NA | Median time per patient, all appointments, is 49.5. Number of appointment activities per patientis 6. | - | Y | N |
|  | UK | Software tracking | General outpatient services | General Medical Practitioners | NA | NA | Median time per patient, face-to-face consultations, is 63. Number of face-to-face consultations per patient is 5. | - | Y | N |
|  | UK | Software tracking | General outpatient services | General Medical Practitioners | NA | NA | Median time per patient, face-to-face consultations, is 41. Number of face-to-face consultations per patient is 4. | - | Y | N |
|  | UK | Software tracking | General outpatient services | General Medical Practitioners | NA | NA | Median time per patient, all appointments, is 64. Number of appointment activities per patientis 7. | - | N | N |
|  | UK | Software tracking | General outpatient services | General Medical Practitioners | NA | NA | Median time per patient, all appointments, is 59. Number of appointment activities per patientis 7. | - | N | N |
|  | UK | Software tracking | General outpatient services | General Medical Practitioners | NA | NA | Median time per patient, face-to-face consultations, is 47. Number of face-to-face consultations per patient is 4. | - | N | N |
|  | UK | Software tracking | General outpatient services | General Medical Practitioners | NA | NA | Median time per patient, face-to-face consultations, is 42. Number of face-to-face consultations per patient is 4. | - | N | N |
| Hanna (119) | USA | Self-reported/questionnaire | Second referral level (and above) | Specialist Medical Practitioners | 15.4 | - | - | SD, 8.63 | Y | Y |
| Hepp (120) | Germany | Software tracking | First referral level | Specialist Medical Practitioners | 9.13 | - | - | - | Y | Y |
| Hickey (121) | USA | Software tracking | Second referral level (and above) | Specialist Medical Practitioners | 10.8 | - | - | - | Y | N |
|  | USA | Software tracking | Second referral level (and above) | Specialist Medical Practitioners | 17.2 | - | - | - | Y | N |
|  | USA | Software tracking | Second referral level (and above) | Specialist Medical Practitioners | 30 | - | - | - | Y | N |
| Higgins (122) | USA | Electronic health records | General outpatient services | Pharmacists | 30.1 | - | - | - | N | N |
| Hilder (123) | New Zealand | Recording (video, audio, stopwatch) | Second referral level (and above) | Specialist Medical Practitioners | 28 | - | 13 were 15–30 mins; 6 were 30–60 min | - | N | N |
| Hirsch (124) | USA |  | Community-based services | Pharmacists | 3.4 | - | - | SD, 1.9 | Y | N |
|  | USA |  | Community-based services | Pharmacists | 2 | - | - | SD, 1.4 | N | N |
|  | USA | Recording (video, audio, stopwatch) | First referral level | Specialist Medical Practitioners | 21.4 | - | - | - | N | N |
|  | USA | Recording (video, audio, stopwatch) | First referral level | Specialist Medical Practitioners | 20.6 | - | - | - | N | N |
| Hiscock (125) | Australia | Self-reported/questionnaire | First referral level | Specialist Medical Practitioners | 51.3 | - | - | SD, 19.2 | N | N |
|  | Australia | Self-reported/questionnaire | First referral level | Specialist Medical Practitioners | 48 | - | - | SD, 16.8 | N | N |
|  | Australia | Self-reported/questionnaire | First referral level | Specialist Medical Practitioners | 48.7 | - | - | SD, 21.7 | N | N |
|  | Australia | Self-reported/questionnaire | First referral level | Specialist Medical Practitioners | 46 | - | - | SD, 21.5 | N | N |
|  | Australia | Self-reported/questionnaire | First referral level | Specialist Medical Practitioners | 43.6 | - | - | SD, 15.7 | N | N |
|  | Australia | Self-reported/questionnaire | First referral level | Specialist Medical Practitioners | 43.5 | - | - | SD, 15.3 | N | N |
|  | Australia | Self-reported/questionnaire | First referral level | Specialist Medical Practitioners | 56.4 | - | - | SD, 25.3 | N | N |
|  | Australia | Self-reported/questionnaire | First referral level | Specialist Medical Practitioners | 46.9 | - | - | SD, 25.1 | N | N |
|  | Australia | Self-reported/questionnaire | First referral level | Specialist Medical Practitioners | 50.9 | - | - | SD, 17.1 | N | N |
|  | Australia | Self-reported/questionnaire | First referral level | Specialist Medical Practitioners | 48.1 | - | - | SD, 16.6 | N | N |
|  | Australia | Self-reported/questionnaire | First referral level | Specialist Medical Practitioners | 57.1 | - | - | SD, 27.4 | N | N |
|  | Australia | Self-reported/questionnaire | First referral level | Specialist Medical Practitioners | 46.6 | - | - | SD, 27 | N | N |
|  | Australia | Self-reported/questionnaire | First referral level | Specialist Medical Practitioners | 51 | - | - | SD, 28.3 | N | N |
|  | Australia | Self-reported/questionnaire | First referral level | Specialist Medical Practitioners | 51.8 | - | - | SD, 27.4 | N | N |
|  | Australia | Self-reported/questionnaire | First referral level | Specialist Medical Practitioners | 46.6 | - | - | SD, 18.4 | N | N |
|  | Australia | Self-reported/questionnaire | First referral level | Specialist Medical Practitioners | 42.2 | - | - | SD, 17.2 | N | N |
|  | Australia | Self-reported/questionnaire | First referral level | Specialist Medical Practitioners | 54.4 | - | - | SD, 22 | N | N |
|  | Australia | Self-reported/questionnaire | First referral level | Specialist Medical Practitioners | 45.5 | - | - | SD, 20 | N | N |
|  | Australia | Self-reported/questionnaire | First referral level | Specialist Medical Practitioners | 50.5 | - | - | SD, 24.3 | N | N |
|  | Australia | Self-reported/questionnaire | First referral level | Specialist Medical Practitioners | 46.1 | - | - | SD, 26.9 | N | N |
|  | Australia | Self-reported/questionnaire | First referral level | Specialist Medical Practitioners | 49.6 | - | - | SD, 17.6 | N | N |
|  | Australia | Self-reported/questionnaire | First referral level | Specialist Medical Practitioners | 45.9 | - | - | SD, 17.6 | N | N |
|  | Australia | Self-reported/questionnaire | First referral level | Specialist Medical Practitioners | 25.5 | - | - | SD, 12.1 | N | N |
|  | Australia | Self-reported/questionnaire | First referral level | Specialist Medical Practitioners | 26.6 | - | - | SD, 11.9 | N | N |
|  | Australia | Self-reported/questionnaire | First referral level | Specialist Medical Practitioners | 27 | - | - | SD, 9.6 | N | N |
|  | Australia | Self-reported/questionnaire | First referral level | Specialist Medical Practitioners | 28.5 | - | - | SD, 9.3 | N | N |
|  | Australia | Self-reported/questionnaire | First referral level | Specialist Medical Practitioners | 29.6 | - | - | SD, 14.6 | N | N |
|  | Australia | Self-reported/questionnaire | First referral level | Specialist Medical Practitioners | 29.5 | - | - | SD, 14.9 | N | N |
|  | Australia | Self-reported/questionnaire | First referral level | Specialist Medical Practitioners | 22.6 | - | - | SD, 10.7 | N | N |
|  | Australia | Self-reported/questionnaire | First referral level | Specialist Medical Practitioners | 21.6 | - | - | SD, 9.6 | N | N |
|  | Australia | Self-reported/questionnaire | First referral level | Specialist Medical Practitioners | 30.7 | - | - | SD, 20.7 | N | N |
|  | Australia | Self-reported/questionnaire | First referral level | Specialist Medical Practitioners | 31.2 | - | - | SD, 20.8 | N | N |
|  | Australia | Self-reported/questionnaire | First referral level | Specialist Medical Practitioners | 26.9 | - | - | SD, 14.9 | N | N |
|  | Australia | Self-reported/questionnaire | First referral level | Specialist Medical Practitioners | 32.3 | - | - | SD, 14 | N | N |
|  | Australia | Self-reported/questionnaire | First referral level | Specialist Medical Practitioners | 24.3 | - | - | SD, 9.5 | N | N |
|  | Australia | Self-reported/questionnaire | First referral level | Specialist Medical Practitioners | 26.7 | - | - | SD, 9.1 | N | N |
|  | Australia | Self-reported/questionnaire | First referral level | Specialist Medical Practitioners | 28.7 | - | - | SD, 13.1 | N | N |
|  | Australia | Self-reported/questionnaire | First referral level | Specialist Medical Practitioners | 29.7 | - | - | SD, 12.3 | N | N |
|  | Australia | Self-reported/questionnaire | First referral level | Specialist Medical Practitioners | 29.6 | - | - | SD, 13.8 | N | N |
|  | Australia | Self-reported/questionnaire | First referral level | Specialist Medical Practitioners | 30.1 | - | - | SD, 13.9 | N | N |
|  | Australia | Self-reported/questionnaire | First referral level | Specialist Medical Practitioners | 25.1 | - | - | SD, 11.2 | N | N |
|  | Australia | Self-reported/questionnaire | First referral level | Specialist Medical Practitioners | 25.7 | - | - | SD, 11.8 | N | N |
|  | Australia | Self-reported/questionnaire | First referral level | Specialist Medical Practitioners | 25.5 | - | - | SD, 11 | N | N |
|  | Australia | Self-reported/questionnaire | First referral level | Specialist Medical Practitioners | 27.3 | - | - | SD, 10.6 | N | N |
| Höglander (126) | Sweden | Recording (video, audio, stopwatch) | General outpatient services | Nursing Professionals | 6.79 | - | - | SD, 6.43 | N | N |
|  | Sweden | Recording (video, audio, stopwatch) | General outpatient services | Nursing Professionals | 7.19 | - | - | SD, 5.14 | N | N |
| Hohman (127) | USA | Software tracking | First referral level | Health Professionals not elsewhere classified | NA | 36.9 | - | - | Y | N |
|  | USA | Software tracking | First referral level | Health Professionals not elsewhere classified | NA | 15.7 | - | - | Y | N |
|  | USA | Software tracking | First referral level | Health Professionals not elsewhere classified | NA | 6.2 | - | - | Y | N |
|  | USA | Software tracking | First referral level | Health Professionals not elsewhere classified | NA | 46.9 | - | - | Y | N |
| Hori (128) | Japan | Self-reported/questionnaire | First referral level | Specialist Medical Practitioners | NA | NA | 1 min (1); 3 mins (1); 5 mins (1); 10 mins (0); 15 mins (3); 20 mins (8); 30 mins (49); 45 mins (24); >60 mins (14) | - | N | N |
|  | Japan | Self-reported/questionnaire | First referral level | Specialist Medical Practitioners | NA | NA | 1 min (1); 3 mins (0); 5 mins (0); 10 mins (0); 15 mins (2); 20 mins (8); 30 mins (48); 45 mins (24); >60 mins (16) | - | N | N |
|  | Japan | Self-reported/questionnaire | First referral level | Specialist Medical Practitioners | NA | NA | 1 min (0); 3 mins (1); 5 mins (1); 10 mins (1); 15 mins (3); 20 mins (9); 30 mins (53); 45 mins (24); >60 mins (9) | - | N | N |
|  | Japan | Self-reported/questionnaire | First referral level | Specialist Medical Practitioners | NA | NA | 1 min (2); 3 mins (6); 5 mins (46); 10 mins (33); 15 mins (10); 20 mins (2); 30 mins (0) 45 mins (0); >60 mins (0) | - | N | N |
| Hsiao (129) | USA | Recording (video, audio, stopwatch) | First referral level | Specialist Medical Practitioners | 29.9 | - | - | SD, 11.6 | N | N |
|  | USA | Recording (video, audio, stopwatch) | First referral level | Specialist Medical Practitioners | 25.1 | - | - | SD, 10.7 | N | N |
| Huang (130) | USA | Electronic health records | First referral level | Pharmacists | 55 | - | - | SD, 19 | N | N |
| Humphries (131) | USA | Self-reported/questionnaire | First referral level | Specialist Medical Practitioners | 99.6 | - | Reported as 1.66 hours | - | N | N |
|  | USA | Self-reported/questionnaire | First referral level | Specialist Medical Practitioners | 240 | - | Reported as 4 hours | - | N | N |
|  | USA | Self-reported/questionnaire | First referral level | Specialist Medical Practitioners | 66 | - | Reported as 1.1 hours | - | N | N |
| Hunter (132) | USA | Recording (video, audio, stopwatch) | First referral level | Specialist Medical Practitioners | NA | 12.0333 | Median consultation time was 12 minutes and 2 seconds. The cost conversation was 33 seconds. | - | N | N |
| Hutchings (133) | Australia | Software tracking | General outpatient services | Nursing Professionals | 8.5 | - | - | - | Y | Y |
|  | Australia | Software tracking | General outpatient services | Nursing Professionals | 15 | - | - | - | Y | Y |
| Hwang (134) | USA | Software tracking | Second referral level (and above) | Specialist Medical Practitioners | NA | NA | Sixty-four consults (81%) lasted 15 minutes or less | - | Y | N |
| Idrees (135) | Pakistan | Self-reported/questionnaire | Second referral level (and above) | Specialist Medical Practitioners | 22.466 | - | This is disagregated to 9.301 mins to take histroy; 8.534 to perform physical examination; 4.833 to write prescription | SD, 10.264 | N | N |
|  | Pakistan | Self-reported/questionnaire | Second referral level (and above) | Specialist Medical Practitioners | 8.939 | - | This is disagregated to 9.301 mins to take histroy; 8.534 to perform physical examination; 4.833 to write prescription | SD, 5.953 | N | N |
| Iftikhar (136) | Pakistan | Self-reported/questionnaire | First referral level | Specialist Medical Practitioners | 6.03 | - | - | SD, 3.34 | N | N |
| Ijaz | Pakistan | Self-reported/questionnaire | Second referral level (and above) | Specialist Medical Practitioners | 6.03 | - | - | SD, 3.34 | N | N |
| Iwasa (137) | Japan | Recording (video, audio, stopwatch) | General outpatient services | Nursing Professionals | NA | 49.12 | The median time spent on a home health visit to each patient was 2,947 s (49 min 7 s) | - | N | N |
| Jabour (138) | Saudi Arabia | Recording (video, audio, stopwatch) | General outpatient services | General Medical Practitioners | 3.3 | - | - | SD, 1.86 | N | N |
|  | Saudi Arabia | Recording (video, audio, stopwatch) | General outpatient services | General Medical Practitioners | 6.39 | - | - | SD, 6.79 | N | N |
|  | Saudi Arabia | Recording (video, audio, stopwatch) | General outpatient services | General Medical Practitioners | 7.43 | - | - | SD, 6.79 | N | N |
|  | Saudi Arabia | Recording (video, audio, stopwatch) | General outpatient services | General Medical Practitioners | 2.83 | - | - | SD, 1.64 | N | N |
|  | Saudi Arabia | Recording (video, audio, stopwatch) | General outpatient services | General Medical Practitioners | 3.1 | - | - | SD, 0.8 | N | N |
|  | Saudi Arabia | Recording (video, audio, stopwatch) | General outpatient services | General Medical Practitioners | 3.05 | - | - | SD, 2.08 | N | N |
| Jácome (139) | Portugal & Spain | Self-reported/questionnaire | First referral level | Specialist Medical Practitioners | 20 | - | - | - | N | N |
|  | Portugal & Spain | Self-reported/questionnaire | First referral level | Specialist Medical Practitioners | 30 | - | - | - | N | Y |
|  | Portugal & Spain | Self-reported/questionnaire | First referral level | Specialist Medical Practitioners | 15 | - | - | - | Y | Y |
|  | Portugal & Spain | Self-reported/questionnaire | First referral level | Specialist Medical Practitioners | 20 | - | - | - | Y | Y |
|  | Portugal & Spain | Self-reported/questionnaire | First referral level | Specialist Medical Practitioners | 20 | - | - | - | N | N |
|  | Portugal & Spain | Self-reported/questionnaire | First referral level | Specialist Medical Practitioners | 30 | - | - | - | N | Y |
|  | Portugal & Spain | Self-reported/questionnaire | First referral level | Specialist Medical Practitioners | 15 | - | - | - | Y | Y |
|  | Portugal & Spain | Self-reported/questionnaire | First referral level | Specialist Medical Practitioners | 20 | - | - | - | Y | Y |
|  | Portugal & Spain | Self-reported/questionnaire | First referral level | Specialist Medical Practitioners | 20 | - | - | - | N | N |
|  | Portugal & Spain | Self-reported/questionnaire | First referral level | Specialist Medical Practitioners | 30 | - | - | - | N | Y |
|  | Portugal & Spain | Self-reported/questionnaire | First referral level | Specialist Medical Practitioners | 10 | - | - | - | Y | Y |
|  | Portugal & Spain | Self-reported/questionnaire | First referral level | Specialist Medical Practitioners | 20 | - | - | - | N | N |
|  | Portugal & Spain | Self-reported/questionnaire | First referral level | Specialist Medical Practitioners | 25 | - | - | - | N | Y |
|  | Portugal & Spain | Self-reported/questionnaire | First referral level | Specialist Medical Practitioners | 15 | - | - | - | Y | Y |
|  | Portugal & Spain | Self-reported/questionnaire | First referral level | Specialist Medical Practitioners | 5 | - | - | - | Y | Y |
| Jahromi (140) | Iran | Self-reported/questionnaire | General outpatient services | General Medical Practitioners | NA | NA | Up to 5 mins (45%); 6-10 mins (38%); 11-15 mins (11%); >15 mins (6%) | - | N | N |
|  | Iran | Self-reported/questionnaire | General outpatient services | General Medical Practitioners | NA | NA | Up to 5 mins (20%); 6-10 mins (53%); 11-15 mins (18%); >15 mins (9%) | - | N | N |
|  | Iran | Self-reported/questionnaire | General outpatient services | General Medical Practitioners | NA | NA | Up to 5 mins (35%); 6-10 mins (44%); 11-15 mins (14%); >15 mins (7%) | - | N | N |
| James (141) | India | Recording (video, audio, stopwatch) | Second referral level (and above) | Specialist Medical Practitioners | 19.85 | 17 | - | SD, 11.04 | N | N |
|  | India | Recording (video, audio, stopwatch) | Second referral level (and above) | Specialist Medical Practitioners | NA | 25 | - | - | N | N |
|  | India | Recording (video, audio, stopwatch) | Second referral level (and above) | Specialist Medical Practitioners | NA | 15 | - | - | N | N |
|  | Australia | Recording (video, audio, stopwatch) | General outpatient services | Nursing Professionals | 28.7 | - | - | - | N | N |
| Janati (142) | Iran | Self-reported/questionnaire | First referral level | Specialist Medical Practitioners | 8.08 | - | - | SD, 1.52 | N | N |
|  | Iran | Self-reported/questionnaire | First referral level | Specialist Medical Practitioners | 7 | - | - | SD, 1.33 | N | N |
|  | Iran | Self-reported/questionnaire | First referral level | Specialist Medical Practitioners | 9.36 | - | - | SD, 2.66 | N | N |
|  | Iran | Self-reported/questionnaire | First referral level | Specialist Medical Practitioners | 14.79 | - | - | SD, 2.8 | N | N |
|  | Iran | Self-reported/questionnaire | First referral level | Specialist Medical Practitioners | 5.63 | - | - | SD, 0.78 | N | N |
|  | Iran | Self-reported/questionnaire | First referral level | Specialist Medical Practitioners | 6.05 | - | - | SD, 1.21 | N | N |
|  | Iran | Self-reported/questionnaire | First referral level | Specialist Medical Practitioners | 5.48 | - | - | SD, 1.46 | N | N |
|  | Iran | Self-reported/questionnaire | First referral level | Specialist Medical Practitioners | 11.52 | - | - | SD, 3.2 | N | N |
|  | Iran | Self-reported/questionnaire | First referral level | Specialist Medical Practitioners | 8.45 | - | - | SD, 1.22 | N | N |
|  | Iran | Self-reported/questionnaire | First referral level | Specialist Medical Practitioners | 10.84 | - | - | SD, 1.68 | N | N |
|  | Iran | Self-reported/questionnaire | First referral level | Specialist Medical Practitioners | 7.46 | - | - | SD, 1.87 | N | N |
|  | Iran | Self-reported/questionnaire | First referral level | Specialist Medical Practitioners | 8.53 | - | - | SD, 1.83 | N | N |
|  | Iran | Self-reported/questionnaire | First referral level | Specialist Medical Practitioners | 8.15 | - | - | SD, 2.53 | N | N |
|  | Iran | Self-reported/questionnaire | First referral level | Specialist Medical Practitioners | 7.62 | - | - | SD, 1.66 | N | N |
|  | Iran | Self-reported/questionnaire | First referral level | Specialist Medical Practitioners | 8.52 | - | - | SD, 3.14 | N | N |
| Jandovitz (143) | USA | Software tracking | Second referral level (and above) | Pharmacists | 11.6 | - | - | SD, 8 | N | N |
| Jegal (144) | South Korea | Self-reported/questionnaire | Other, specify: | Specialist Medical Practitioners | NA | NA | <10 mins (14.5%); 10-20 mins (43.1%); 20-30 mins (21.4%); >30 mins (21.0%) | - | N | N |
|  | South Korea | Self-reported/questionnaire | Other, specify: | Specialist Medical Practitioners | NA | NA | <10 mins (21.6%); 10-20 mins (43.1%); 20-30 mins (19.6%); >30 mins (15.7%) | - | N | N |
|  | South Korea | Self-reported/questionnaire | Other, specify: | Specialist Medical Practitioners | NA | NA | <10 mins (14.1%); 10-20 mins (43.1%); 20-30 mins (21.5%); >30 mins (21.3%) | - | N | N |
| Jin (145) | China | Self-reported/questionnaire | General outpatient services | General Medical Practitioners | NA | 6 | - | - | N | N |
| Jones (146) | USA | Recording (video, audio, stopwatch) | Second referral level (and above) | Specialist Medical Practitioners | 6.6 | - | - | 95% CI, 3.3 - 13 | N | N |
|  | USA | Recording (video, audio, stopwatch) | Second referral level (and above) | Specialist Medical Practitioners | 7.3 | - | - | 95% CI, 4.6 - 11.4 | N | N |
|  | USA | Recording (video, audio, stopwatch) | Second referral level (and above) | Nursing Professionals | 19 | - | - | 95% CI, 16.4 - 22 | N | N |
|  | USA | Recording (video, audio, stopwatch) | Second referral level (and above) | Specialist Medical Practitioners | 14.3 | - | - | 95% CI, 10.2 - 19.9 | N | N |
| Joshi (147) | India | Self-reported/questionnaire | General outpatient services | Specialist Medical Practitioners | NA | NA | <5 mins (11.9%); 5-10 mins (61.5%); 10- 30 mins (25.5%); >30 mins (1.0%) | - | N | N |
| Kabeya (148) | Japan | Electronic health records | First referral level | Specialist Medical Practitioners | 10.1 | 9 | - | - | N | N |
|  | Japan | Electronic health records | First referral level | Specialist Medical Practitioners | 10.6 | - | - | SD, 5.5 | N | N |
|  | Japan | Electronic health records | First referral level | Specialist Medical Practitioners | 9.7 | - | - | SD, 5.2 | N | N |
|  | Japan | Electronic health records | First referral level | Specialist Medical Practitioners | 10 | - | - | SD, 5.4 | N | N |
|  | Japan | Electronic health records | First referral level | Specialist Medical Practitioners | 10.4 | - | - | SD, 4.8 | N | N |
|  | Japan | Electronic health records | First referral level | Specialist Medical Practitioners | 9.8 | - | - | SD, 5.3 | N | N |
|  | Japan | Electronic health records | First referral level | Specialist Medical Practitioners | 11 | - | - | SD, 5.3 | N | N |
|  | Japan | Electronic health records | First referral level | Specialist Medical Practitioners | 9.5 | - | - | SD, 5.4 | N | N |
|  | Japan | Electronic health records | First referral level | Specialist Medical Practitioners | 10.4 | - | - | SD, 5.2 | N | N |
|  | Japan | Electronic health records | First referral level | Specialist Medical Practitioners | 11.4 | - | - | SD, 4.7 | N | N |
|  | Japan | Electronic health records | First referral level | Specialist Medical Practitioners | 9.6 | - | - | SD, 5.1 | N | N |
|  | Japan | Electronic health records | First referral level | Specialist Medical Practitioners | 12 | - | - | SD, 5.5 | N | N |
|  | Japan | Electronic health records | First referral level | Specialist Medical Practitioners | 9.8 | - | - | SD, 5 | N | N |
|  | Japan | Electronic health records | First referral level | Specialist Medical Practitioners | 13.6 | - | - | SD, 6.3 | N | N |
|  | Japan | Electronic health records | First referral level | Specialist Medical Practitioners | 11.1 | - | - | SD, 6.2 | N | N |
|  | Japan | Electronic health records | First referral level | Specialist Medical Practitioners | 9.8 | - | - | SD, 5.1 | N | N |
|  | Japan | Electronic health records | First referral level | Specialist Medical Practitioners | 10 | - | - | SD, 5.2 | N | N |
|  | Japan | Electronic health records | First referral level | Specialist Medical Practitioners | 11 | - | - | SD, 4.6 | N | N |
|  | Japan | Electronic health records | First referral level | Specialist Medical Practitioners | 9.1 | - | - | SD, 4.9 | N | N |
|  | Japan | Electronic health records | First referral level | Specialist Medical Practitioners | 9.8 | - | - | SD, 5.1 | N | N |
|  | Japan | Electronic health records | First referral level | Specialist Medical Practitioners | 11.3 | - | - | SD, 5.7 | N | N |
|  | Japan | Electronic health records | First referral level | Specialist Medical Practitioners | 12.3 | - | - | SD, 5.2 | N | N |
|  | Japan | Electronic health records | First referral level | Specialist Medical Practitioners | 10.5 | - | - | SD, 5.6 | N | N |
|  | Japan | Electronic health records | First referral level | Specialist Medical Practitioners | 10.2 | - | - | SD, 5.4 | N | N |
|  | Japan | Electronic health records | First referral level | Specialist Medical Practitioners | 9.4 | - | - | SD, 4.4 | N | N |
|  | Japan | Electronic health records | First referral level | Specialist Medical Practitioners | 9.1 | - | - | SD, 4.8 | N | N |
|  | Japan | Electronic health records | First referral level | Specialist Medical Practitioners | 12 | - | - | SD, 5.7 | N | N |
|  | Japan | Electronic health records | First referral level | Specialist Medical Practitioners | 10.1 | - | - | SD, 5.3 | N | N |
|  | Japan | Electronic health records | First referral level | Specialist Medical Practitioners | 11.4 | - | - | SD, 4.9 | N | N |
|  | Japan | Electronic health records | First referral level | Specialist Medical Practitioners | 10.3 | - | - | SD, 5.4 | N | N |
|  | Japan | Electronic health records | First referral level | Specialist Medical Practitioners | 10.7 | - | - | SD, 5.8 | N | N |
|  | Japan | Electronic health records | First referral level | Specialist Medical Practitioners | 9.2 | - | - | SD, 4.5 | N | N |
| Kagedan (149) | USA | Recording (video, audio, stopwatch) | First referral level | Specialist Medical Practitioners | NA | 49 | - | - | N | N |
|  | USA | Recording (video, audio, stopwatch) | First referral level | Specialist Medical Practitioners | NA | 43 | - | - | N | N |
|  | USA | Recording (video, audio, stopwatch) | First referral level | Specialist Medical Practitioners | NA | 53 | - | - | N | N |
|  | USA | Recording (video, audio, stopwatch) | First referral level | Specialist Medical Practitioners | NA | 50 | - | - | N | N |
| Kantor (150) | USA | Self-reported/questionnaire | First referral level | Specialist Medical Practitioners | 22 | - | - | - | N | N |
|  | USA | Self-reported/questionnaire | First referral level | Specialist Medical Practitioners | 25 | - | - | - | N | N |
| Karatas (151) | Turkey | Self-reported/questionnaire | First referral level | Specialist Medical Practitioners | 15.26 | 15 | - | SD, 3.55 | N | N |
| Karia (152) | Australia | Recording (video, audio, stopwatch) | Community-based services | Pharmacists | NA | 4.12 | 4 mins 7 seconds | - | N | N |
| Kasturi (153) | USA | Self-reported/questionnaire | First referral level | Specialist Medical Practitioners | 17 | - | - | - | N | N |
| Kelly (154) | Australia | Existing databases or guidelines | General outpatient services | Nursing Professionals | NA | NA | <10 mins (3.6%); 10-20 mins (58.5%); 20-40 mins (33.6%); >40 mins 4.3%) | - | N | N |
| Keyworth (155) | UK | Self-reported/questionnaire | General outpatient services | General Medical Practitioners | 31 | - | - | SD, 19.54 | N | N |
|  | UK | Self-reported/questionnaire | Prehospital emergency services | Ambulance Workers | 48 | - | - | SD, 17.15 | N | N |
|  | UK | Self-reported/questionnaire | Other, specify: | Health Professionals not elsewhere classified | 35 | - | - | SD, 20.15 | N | N |
|  | UK | Self-reported/questionnaire | General outpatient services | General Medical Practitioners | 22 | - | - | SD, 15.92 | N | N |
|  | UK | Self-reported/questionnaire | First referral level | Midwifery Professionals | 37 | - | - | SD, 16.87 | N | N |
|  | UK | Self-reported/questionnaire | General outpatient services | Nursing Professionals | 37 | - | - | SD, 19.96 | N | N |
|  | UK | Self-reported/questionnaire | General outpatient services | Nursing Professionals | 26 | - | - | SD, 18.03 | N | N |
|  | UK | Self-reported/questionnaire | First referral level | Health Professionals not elsewhere classified | 36 | - | - | SD, 20.15 | N | N |
|  | UK | Self-reported/questionnaire | First referral level | Health Professionals not elsewhere classified | 12 | - | - | SD, 19.75 | N | N |
|  | UK | Self-reported/questionnaire | First referral level | Specialist Medical Practitioners | 25 | - | - | SD, 14.13 | N | N |
| Khairat (156) | USA | Software tracking | Other, specify: | Specialist Medical Practitioners | 10.5 | - | - | SD, 4.9 | Y | Y |
|  | USA | Software tracking | Other, specify: | Specialist Medical Practitioners | 11 | - | - | SD, 5.2 | Y | Y |
|  | USA | Software tracking | Other, specify: | Specialist Medical Practitioners | 9.6 | - | - | SD, 4.1 | Y | Y |
| Kieran (157) | Ireland | Software tracking | First referral level | Specialist Medical Practitioners | NA | 18 | - | - | Y | Y |
| Kilduff (158) | UK | Software tracking | First referral level | Specialist Medical Practitioners | 12 | - | - | - | Y | Y |
| Kim (159) | South Korea | Self-reported/questionnaire | First referral level | Specialist Medical Practitioners | 12.3 | - | - | SD, 5.4 | N | N |
|  | South Korea | Self-reported/questionnaire | First referral level | Specialist Medical Practitioners | 4.8 | - | - | SD, 1.8 | N | N |
| Kim (160) | USA | Software tracking | Other, specify: | Nursing Professionals | NA | 9 | - | - | Y | N |
|  | USA | Software tracking | Other, specify: | Specialist Medical Practitioners | NA | 10 | - | - | Y | N |
| Kleven (161) | USA | Recording (video, audio, stopwatch) | Other, specify: | Pharmacists | 15.43 | - | Two groups - group 1: 15.43 min (3.27); group 2: 20 min (7.43) | SD, 3.27 | Y | N |
| Ko (162) | USA | Self-reported/questionnaire | First referral level | Specialist Medical Practitioners | 19.8 | - | On average, inpatient teams see approximately 3.7 new (initial inpatient evaluations) and 4.2 follow-up (subsequent inpatient evaluations) patients during daily rounds, which last an average of 2.6 hours. | - | N | N |
| Kohlert (163) | Canada | Self-reported/questionnaire | First referral level | Specialist Medical Practitioners | NA | NA | <10 mins (76.1%), <15 mins (97.2%), <20 mins (100%) | - | Y | N |
| Korn (164) | USA |  | Other, specify: | Specialist Medical Practitioners | 27.9 | 20 | - | - | Y | N |
| Kortlever (165) | USA | Recording (video, audio, stopwatch) | Second referral level (and above) | Specialist Medical Practitioners | 8.7 | - | - | SD, 5.3 | N | N |
| Kottak (166) | USA | Recording (video, audio, stopwatch) | First referral level | Specialist Medical Practitioners | 15.1 | - | 15 minutes, 6 seconds | - | N | N |
| Kruk (167) | Haiti | Existing databases or guidelines | General outpatient services | General Medical Practitioners | NA | 10 | - | - | N | N |
|  | Haiti, Kenya, Malawi, Namibia, Nepal, Rwanda, Senegal, Tanzania, and Uganda | Existing databases or guidelines | General outpatient services | Nursing Professionals | NA | 8 | - | - | N | N |
|  | Kenya | Existing databases or guidelines | General outpatient services | Nursing Professionals | NA | 8 | - | - | N | N |
|  | Malawi | Existing databases or guidelines | General outpatient services | Health Professionals not elsewhere classified | NA | 6 | - | - | N | N |
|  | Namibia | Existing databases or guidelines | General outpatient services | Nursing Professionals | NA | 10 | - | - | N | N |
|  | Nepal | Existing databases or guidelines | General outpatient services | Health Professionals not elsewhere classified | NA | 6 | - | - | N | N |
|  | Rwanda | Existing databases or guidelines | General outpatient services | Nursing Professionals | NA | 6 | - | - | N | N |
|  | Senegal | Existing databases or guidelines | General outpatient services | Nursing Professionals | NA | 9 | - | - | N | N |
|  | Tanzania | Existing databases or guidelines | General outpatient services | Health Professionals not elsewhere classified | NA | 12 | - | - | N | N |
|  | Uganda | Existing databases or guidelines | General outpatient services | Nursing Professionals | NA | 6 | - | - | N | N |
| Landon (168) | Australia | Existing databases or guidelines | General outpatient services | General Medical Practitioners | 14.4 | - | - | - | N | N |
|  | Australia | Existing databases or guidelines | General outpatient services | General Medical Practitioners | 15.2 | - | - | - | N | N |
|  | USA | Existing databases or guidelines | General outpatient services | General Medical Practitioners | 17.2 | - | - | - | N | N |
|  | USA | Existing databases or guidelines | General outpatient services | General Medical Practitioners | 22.9 | - | - | - | N | N |
| Laurenzi (169) | South Africa | Recording (video, audio, stopwatch) | Community-based services | Community Health Workers | 16.22 | - | Time = 16:13; SD = 7:15; min = 3:30; max = 33:54. | SD, 7.25 | N | N |
| Leask (170) | UK | Self-reported/questionnaire | General outpatient services | Nursing Professionals | 27 | 24 | - | SD, 14.2 | N | N |
| Lebanova (171) | Bulgaria | Self-reported/questionnaire | Community-based services | Pharmacists | NA | NA | 53% of the respondents state that the median duration of consultation for an OTC product is between 5 and 10 minutes and less than 5 minutes for 44% | - | N | N |
| Lee (172) | South Korea | Self-reported/questionnaire | General outpatient services | General Medical Practitioners | 5.23 | - | - | SD, 3.56 | N | N |
|  | South Korea | Self-reported/questionnaire | General outpatient services | General Medical Practitioners | 9.58 | - | - | SD, 5 | N | N |
| Lee (173) | Malaysia | Recording (video, audio, stopwatch) | General outpatient services | General Medical Practitioners | 14.3 | - | - | SD, 5.75 | N | N |
| Lehmann (174) | Netherlands | Recording (video, audio, stopwatch) | Second referral level (and above) | Specialist Medical Practitioners | 39.95 | - | Mean duration: 39:57. | SD, 12.1 | N | N |
| Leone (175) | Italy | Recording (video, audio, stopwatch) | Second referral level (and above) | Specialist Medical Practitioners | 37 | - | - | SD, 17.7 | N | N |
| Li (176) | China | Software tracking | First referral level | Specialist Medical Practitioners | 11 | - | - | SD, 8.9 | Y | Y |
| Lim (177) | Singapore | Self-reported/questionnaire | General outpatient services | General Medical Practitioners | NA | 10 | - | - | N | N |
|  | Singapore | Self-reported/questionnaire | Second referral level (and above) | Specialist Medical Practitioners | NA | 20 | - | - | N | N |
| Lin (178) | USA | Existing databases or guidelines | First referral level | Specialist Medical Practitioners | 21 | - | - | - | N | N |
| Lindberg (179) | Norway | Electronic health records | General outpatient services | General Medical Practitioners | NA | 12.1 | - | - | N | N |
|  | Norway | Electronic health records | General outpatient services | General Medical Practitioners | NA | 11.5 | - | - | N | N |
| Liu (180) | China | Software tracking | Second referral level (and above) | Specialist Medical Practitioners | 7.58 | - | - | SD, 6.17 | Y | N |
| Liu (181) | China | Self-reported/questionnaire | General outpatient services | General Medical Practitioners | 8.1 | - | - | SD, 6.98 | N | N |
| Liu (182) | USA | Other, specify: | Second referral level (and above) | Nursing Professionals | NA | NA | N/A | - | Y | N |
| Livori (183) | Australia | Recording (video, audio, stopwatch) | First referral level | Specialist Medical Practitioners | 13.5 | - | - | SD, 6.6 | N | N |
|  | Australia | Recording (video, audio, stopwatch) | First referral level | Specialist Medical Practitioners | 9.4 | - | - | SD, 3.2 | Y | N |
| Looi (184) | Australia | Existing databases or guidelines | First referral level | Specialist Medical Practitioners | NA | NA | <15min (6917); 15-30min (61,291); 30-45 min (187,043); 45–75min (168,717); 75min plus (10,565) | - | N | Y |
|  | Australia | Existing databases or guidelines | First referral level | Specialist Medical Practitioners | NA | NA | <15min (12,961); 15-30min (109,249); 30-45 min (308,666); 45–75min (302,723); 75min plus (17,065) | - | N | N |
|  | Australia | Existing databases or guidelines | First referral level | Specialist Medical Practitioners | NA | NA | <15min (2957); 15-30min (19087); 30-45 min (56,242); 45–75min (92,858); 75min plus (3214) | - | Y | Y |
|  | Australia | Existing databases or guidelines | First referral level | Specialist Medical Practitioners | NA | NA | <15min (18,561); 15-30min (66,075); 30-45 min (116,076); 45–75min (65,792); 75min plus (2,858) | - | Y | Y |
|  | Australia | Existing databases or guidelines | First referral level | Specialist Medical Practitioners | NA | NA | <15min (1088); 15-30min (11,672); 30-45 min (38,345); 45–75min (44,085); 75min plus (2150) | - | N | Y |
|  | Australia | Existing databases or guidelines | First referral level | Specialist Medical Practitioners | NA | NA | <15min (2275); 15-30min (25,421); 30-45 min (87,805); 45–75min (101,523); 75min plus (5147) | - | N | N |
|  | Australia | Existing databases or guidelines | First referral level | Specialist Medical Practitioners | NA | NA | <15min (880); 15-30min (6079); 30-45 min (20,622); 45–75min (40,885); 75min plus (1053) | - | Y | Y |
|  | Australia | Existing databases or guidelines | First referral level | Specialist Medical Practitioners | NA | NA | <15min (6146); 15-30min (24,810); 30-45 min (47,699); 45–75min (27,511); 75min plus (883) | - | Y | Y |
| Lopez (185) | USA |  | First referral level | Specialist Medical Practitioners | NA | 45 | - | - | N | N |
|  | USA |  | First referral level | Specialist Medical Practitioners | NA | 25 | - | - | Y | Y |
| Lun (186) | Canada | Self-reported/questionnaire | Second referral level (and above) | Specialist Medical Practitioners | NA | NA | 0 min (0%); < 10 min (0%); 10 - 20 min (31%); 20 - 30 min (52%) 30 - 40 min (5%); > 40 min (2%) | - | N | Y |
|  | Canada | Self-reported/questionnaire | Second referral level (and above) | Specialist Medical Practitioners | NA | NA | 0 min (0%); < 10 min (30%); 10 - 20 min (49%); 20 - 30 min (17%) 30 - 40 min (3%); > 40 min (0%) | - | Y | Y |
| Lurquin (187) | France | Recording (video, audio, stopwatch) | General outpatient services | General Medical Practitioners | 14.8 | - | - | SD, 7.3 | N | N |
|  | France | Recording (video, audio, stopwatch) | General outpatient services | General Medical Practitioners | 16 | - | - | SD, 8 | N | N |
|  | France | Recording (video, audio, stopwatch) | General outpatient services | General Medical Practitioners | 17.4 | - | - | SD, 8.5 | N | N |
| Ly (188) | USA | Existing databases or guidelines | General outpatient services | General Medical Practitioners | 22 | - | - | - | N | N |
|  | USA | Existing databases or guidelines | General outpatient services | General Medical Practitioners | 25 | - | - | - | N | N |
|  | USA | Existing databases or guidelines | General outpatient services | General Medical Practitioners | 24.8 | - | - | - | N | N |
|  | USA | Existing databases or guidelines | General outpatient services | General Medical Practitioners | 20.5 | - | - | - | N | N |
|  | USA | Existing databases or guidelines | General outpatient services | General Medical Practitioners | 20.5 | - | - | - | N | N |
|  | USA | Existing databases or guidelines | General outpatient services | General Medical Practitioners | 19.3 | - | - | - | N | N |
| Mabuto (189) | South Africa | Recording (video, audio, stopwatch) | General outpatient services | Social work and counselling professionals | NA | 29 | - | - | N | N |
|  | South Africa | Recording (video, audio, stopwatch) | General outpatient services | General Medical Practitioners | NA | 10 | - | - | N | N |
|  | South Africa | Recording (video, audio, stopwatch) | General outpatient services | Nursing Associate Professionals | NA | 5 | - | - | N | N |
| Mahmoud (190) | Egypt; Saudi Arabia | Self-reported/questionnaire | Second referral level (and above) | Specialist Medical Practitioners | 5 | - | - | SD, 3.5 | Y | Y |
|  | Egypt; Saudi Arabia | Self-reported/questionnaire | Second referral level (and above) | Specialist Medical Practitioners | 5.5 | - | - | SD, 2.9 | N | N |
| Makhlouf (191) | Qatar | Self-reported/questionnaire | Community-based services | Pharmacists | NA | NA | <=5 min (40.1%); 6–10 min (51.8%); 11–15 min (6.7%); 16–20 min (1.4%) | - | N | N |
| Mamo (192) | Ethiopia | Recording (video, audio, stopwatch) | First referral level | Pharmacists | 1.57 | - | - | - | N | N |
| Mandavia (193) | Nepal | Recording (video, audio, stopwatch) | First referral level | Specialist Medical Practitioners | NA | 6 | - | - | Y | N |
| Martin (194) | USA | Electronic health records | General outpatient services | Pharmacists | 30 | - | - | SD, 8.64 | N | N |
| Martinez (195) | USA | Software tracking | Other, specify: | Specialist Medical Practitioners | 6.6 | - | - | - | Y | N |
| Martinez (196) | USA | Software tracking | Other, specify: | Specialist Medical Practitioners | 6.5 | - | - | SD, 3.5 | Y | N |
|  | USA | Software tracking | Other, specify: | Specialist Medical Practitioners | 5.1 | - | - | SD, 2.9 | Y | N |
| Marty (197) | Switzerland | Self-reported/questionnaire | Other, specify: | Specialist Medical Practitioners | NA | NA | <15min(1%); 15-30min (5%); 31-60min (22%); 61-120min (50%); >120min (21%) | - | N | N |
| Mathijssen (198) | Netherlands | Recording (video, audio, stopwatch) | Second referral level (and above) | Specialist Medical Practitioners | NA | 9 | - | - | N | N |
| Matsushita (199) | Japan | Electronic health records | General outpatient services | General Medical Practitioners | 12.3 | - | - | SD, 11.2 | N | N |
|  | Japan | Electronic health records | General outpatient services | General Medical Practitioners | 10.4 | - | - | SD, 6.4 | N | N |
|  | Japan | Electronic health records | General outpatient services | General Medical Practitioners | 10.1 | - | - | SD, 8.4 | N | N |
|  | Japan | Electronic health records | General outpatient services | General Medical Practitioners | 13 | - | - | SD, 10.4 | N | N |
|  | Japan | Electronic health records | General outpatient services | Pharmacists | 7.6 | - | - | SD, 4.9 | N | N |
| Matta (200) | USA | Recording (video, audio, stopwatch) | General outpatient services | General Medical Practitioners | 20.6 | - | - | - | N | N |
| McBain (201) | Haiti | Recording (video, audio, stopwatch) | General outpatient services | Pharmaceutical Technicians and Assistants | 3 | - | - | - | N | N |
|  | Haiti | Recording (video, audio, stopwatch) | General outpatient services | General Medical Practitioners | 5 | - | - | - | N | N |
|  | Haiti | Recording (video, audio, stopwatch) | General outpatient services | Nursing Professionals | 4 | - | - | - | N | N |
|  | Haiti | Recording (video, audio, stopwatch) | General outpatient services | Social work and counselling professionals | 22 | - | - | - | N | N |
|  | Haiti | Recording (video, audio, stopwatch) | First referral level |  | 11 | - | - | - | N | N |
|  | Haiti | Recording (video, audio, stopwatch) | First referral level |  | 5 | - | - | - | N | N |
| McCabe (202) | UK | Recording (video, audio, stopwatch) | Second referral level (and above) | Specialist Medical Practitioners | 26.7 | - | - | - | N | N |
| McGloin (203) | Ireland | Self-reported/questionnaire | General outpatient services | Nursing Professionals | NA | NA | The mean length of the call per participant ranged from 4 min to 15 min | - | Y | N |
| McKenzie (204) | UK | Recording (video, audio, stopwatch) | General outpatient services | General Medical Practitioners | 9.85 | - | - | SD, 5.05 | N | N |
| McKirdy (205) | UK | Existing databases or guidelines | First referral level | Specialist Medical Practitioners | 13.73 | - | 13 minutes, 44 seconds | SD, 2.13 | N | N |
|  | UK | Existing databases or guidelines | First referral level | Specialist Medical Practitioners | 14.88 | - | 14 minutes 53 seconds | SD, 1.77 | Y | N |
| Mehra (206) | India | Self-reported/questionnaire | First referral level | Specialist Medical Practitioners | NA | NA | <5 min (63); 6-10 min (36); 11-15 min (158); 16-25 (241); >26 min (7) | - | N | N |
| Meijers (207) | Netherlands | Recording (video, audio, stopwatch) | General outpatient services | General Medical Practitioners | 9.24 | - | - | SD, 5 | N | N |
|  | Netherlands | Recording (video, audio, stopwatch) | General outpatient services | General Medical Practitioners | 11.28 | - | - | SD, 4.2 | N | N |
| Meijers (208) | Netherlands | Recording (video, audio, stopwatch) | General outpatient services | General Medical Practitioners | 10.7 | - | - | SD, 4.9 | N | N |
|  | Netherlands | Recording (video, audio, stopwatch) | General outpatient services | General Medical Practitioners | 13.4 | - | - | SD, 5.9 | N | N |
| Meleis (209) | USA | Existing databases or guidelines | Second referral level (and above) | Pharmacists | 22.4 | - | - | - | N | N |
| Melian (210) | New Zealand | Self-reported/questionnaire | First referral level | Specialist Medical Practitioners | 6.7 | - | - | SE, 0.18 | N | Y |
|  | New Zealand | Self-reported/questionnaire | First referral level | Specialist Medical Practitioners | 5.1 | - | - | SE, 0.17 | Y | Y |
|  | New Zealand | Self-reported/questionnaire | First referral level | Specialist Medical Practitioners | 8.5 | - | - | SE, 0.295% CI, 8.067 - 8.87 | Y | Y |
|  | New Zealand | Self-reported/questionnaire | First referral level | Specialist Medical Practitioners | 5 | - | - | SE, 0.1695% CI, 4.73 - 5.37 | Y | Y |
|  | New Zealand | Self-reported/questionnaire | First referral level | Specialist Medical Practitioners | 4.1 | - | - | SE, 0.2995% CI, 3.54 - 4.67 | Y | Y |
| Mensa (211) | Ethiopia |  | First referral level | Pharmacists | 3.82 | - | - | - | N | N |
|  | Ethiopia |  | First referral level | Pharmacists | 3.66 | - | - | - | N | N |
| Mercer (212) | UK | Recording (video, audio, stopwatch) | General outpatient services | General Medical Practitioners | 9.34 | - | - | SD, 10.55 | N | N |
|  | UK | Recording (video, audio, stopwatch) | General outpatient services | General Medical Practitioners | 12.79 | - | - | SD, 17.7 | N | N |
|  | UK | Recording (video, audio, stopwatch) | General outpatient services | General Medical Practitioners | 9.96 | - | - | SD, 13.4 | N | N |
|  | UK | Recording (video, audio, stopwatch) | General outpatient services | General Medical Practitioners | 9.94 | - | - | SD, 9.73 | N | N |
| Michael (213) | Nigeria | Self-reported/questionnaire | First referral level | Specialist Medical Practitioners | NA | NA | 1-10 min (196); 11-20 min (108); 21-60 min (30) | - | N | N |
|  | Nigeria | Self-reported/questionnaire | First referral level | Specialist Medical Practitioners | NA | NA | 1-10 min (138); 11-20 min (53); >20 min (11) | - | N | N |
|  | Nigeria | Self-reported/questionnaire | First referral level | Specialist Medical Practitioners | NA | NA | 1-10 min (58); 11-20 min (55); >20 min (19) | - | N | N |
| Michel (214) | France | Self-reported/questionnaire | General outpatient services | General Medical Practitioners | NA | 17 | - | - | N | N |
| Milford (215) | USA | Recording (video, audio, stopwatch) | General outpatient services | Medical Assistants | 8 | - | - | - | N | N |
|  | USA | Recording (video, audio, stopwatch) | General outpatient services | Medical Assistants | 0 | - | - | - | N | N |
|  | USA | Recording (video, audio, stopwatch) | General outpatient services | Medical Assistants | 6 | - | - | - | N | N |
|  | USA | Recording (video, audio, stopwatch) | General outpatient services | Medical Assistants | 1 | - | - | - | N | N |
|  | USA | Recording (video, audio, stopwatch) | General outpatient services | Medical Assistants | 8 | - | - | - | N | N |
|  | USA | Recording (video, audio, stopwatch) | General outpatient services | Medical Assistants | 1 | - | - | - | N | N |
|  | USA | Recording (video, audio, stopwatch) | General outpatient services | Medical Assistants | 7 | - | - | - | N | N |
|  | USA | Recording (video, audio, stopwatch) | General outpatient services | Medical Assistants | 4 | - | - | - | N | N |
|  | USA | Recording (video, audio, stopwatch) | General outpatient services | General Medical Practitioners | 14 | - | - | - | N | N |
|  | USA | Recording (video, audio, stopwatch) | General outpatient services | General Medical Practitioners | 18 | - | - | - | N | N |
|  | USA | Recording (video, audio, stopwatch) | General outpatient services | General Medical Practitioners | 16 | - | - | - | N | N |
|  | USA | Recording (video, audio, stopwatch) | General outpatient services | General Medical Practitioners | 15 | - | - | - | N | N |
| Mohamed Ibrahim (216) | Qatar | Self-reported/questionnaire | Community-based services | Pharmacists | NA | 7.5 | - | - | N | N |
|  | Qatar | Self-reported/questionnaire | Community-based services | Pharmacists | NA | 6.5 | - | - | N | N |
| Mohamoud (217) | Kenya | Recording (video, audio, stopwatch) | General outpatient services | General Medical Practitioners | NA | 7 | - | - | N | N |
|  | Kenya | Recording (video, audio, stopwatch) | General outpatient services | General Medical Practitioners | NA | 9 | - | - | N | N |
|  | Kenya | Recording (video, audio, stopwatch) | General outpatient services | General Medical Practitioners | NA | 4 | - | - | N | N |
| Mugada (218) | India |  | Second referral level (and above) | Pharmacists | 4.57 | - | - | SD, 1.9 | N | N |
| Murphy (219) | Canada | Self-reported/questionnaire | Community-based services | Pharmacists | NA | 15 | - | - | N | N |
| Murren-Boezem (220) | USA | Software tracking | Other, specify: | Specialist Medical Practitioners | 10.12 | - | - | SD, 4.42 | Y | N |
| Musser (221) | USA | Software tracking | First referral level | Health Professionals not elsewhere classified | 44.7 | 39 | - | SD, 27.6 | N | N |
|  | USA | Software tracking | First referral level | Health Professionals not elsewhere classified | 40.7 | 34 | - | SD, 27.2 | N | N |
|  | USA | Software tracking | First referral level | Specialist Medical Practitioners | 17.7 | 14 | - | SD, 13.7 | N | N |
|  | USA | Software tracking | First referral level | Specialist Medical Practitioners | 16.7 | 12 | - | SD, 13.8 | N | N |
|  | USA | Software tracking | First referral level | Health Professionals not elsewhere classified | 23.8 | 21 | - | SD, 13.6 | N | N |
|  | USA | Software tracking | First referral level | Health Professionals not elsewhere classified | 22.5 | 20 | - | SD, 14.5 | N | N |
|  | USA | Software tracking | First referral level | Health Professionals not elsewhere classified | 20.8 | 19 | - | SD, 12.9 | N | N |
|  | USA | Software tracking | First referral level | Health Professionals not elsewhere classified | 22.1 | 14.7 | - | SD, 20 | N | N |
| Mustafa (222) | USA |  | Second referral level (and above) | Specialist Medical Practitioners | 15 | - | - | - | Y | N |
|  | USA |  | Second referral level (and above) | Specialist Medical Practitioners | 60 | - | - | - | N | N |
| Mutemwa (223) | Kenya | Self-reported/questionnaire | General outpatient services | Health associate professionals not elsewhere classified | 22 | 16 | - | - | N | N |
| Nagykaldi (224) | USA | Recording (video, audio, stopwatch) | General outpatient services | General Medical Practitioners | 24 | - | Mentions: The average duration of AWVs and total talk time per AWV were the same in the pre- and post-intervention groups: 24 and 15 minutes, respectively | - | N | N |
| Nair (225) | India | Software tracking | Second referral level (and above) | Specialist Medical Practitioners | NA | 8 | - | - | Y | Y |
| Nakano (226) | Japan | Existing databases or guidelines | General outpatient services | Health Professionals not elsewhere classified | 20.2 | - | - | SD, 14.7 | Y | N |
| Nathan (227) | Israel | Existing databases or guidelines | General outpatient services | General Medical Practitioners | 7.6 | 7 | - | SD, 4.3 | N | N |
| Neke (228) | Tanzania | Self-reported/questionnaire | General outpatient services | Health Professionals not elsewhere classified | 29.4 | - | 0.49 hours | - | N | N |
|  | Tanzania | Self-reported/questionnaire | General outpatient services | Health Professionals not elsewhere classified | 27 | - | 0.45 hours | - | N | N |
|  | Tanzania | Self-reported/questionnaire | General outpatient services | Health Professionals not elsewhere classified | 28.8 | - | 0.48 hours | - | N | N |
| Newell (229) | USA | Electronic health records | First referral level | Nursing Professionals | 58.97 | - | - | - | N | N |
|  | USA | Electronic health records | First referral level | Nursing Professionals | 55.47 | - | - | - | N | N |
| Newman-Casey (230) | USA | Recording (video, audio, stopwatch) | Second referral level (and above) | Health Professionals not elsewhere classified | 118.2 | - | - | SD, 21.3 | N | N |
|  | USA | Recording (video, audio, stopwatch) | Second referral level (and above) | Health Professionals not elsewhere classified | 45.9 | - | - | SD, 24.5 | N | N |
|  | USA | Recording (video, audio, stopwatch) | Second referral level (and above) | Specialist Medical Practitioners | 17.8 | - | - | SD, 8.9 | N | N |
|  | USA | Recording (video, audio, stopwatch) | Second referral level (and above) | Specialist Medical Practitioners | 10.9 | - | - | SD, 7.4 | N | N |
| Nikjoo (231) | Iran | Recording (video, audio, stopwatch) | First referral level | Specialist Medical Practitioners | 25.5 | - | - | SD, 26.3 | N | N |
|  | Iran | Recording (video, audio, stopwatch) | First referral level | Specialist Medical Practitioners | 25.4 | - | - | SD, 24.9 | N | N |
| Nyabuti (232) | Kenya | Self-reported/questionnaire | General outpatient services | General Medical Practitioners | 4.1 | - | - | - | N | N |
| Ourth (233) | USA | Electronic health records |  | Pharmacists | 27.9 | - | - | SD, 19.5 | Y | N |
| Pappalardo (234) | Australia | Existing databases or guidelines | General outpatient services | General Medical Practitioners | 21 | - | - | SD, 10 | N | N |
|  | Australia | Existing databases or guidelines | General outpatient services | General Medical Practitioners | 19 | - | - | SD, 10 | N | N |
| Parikh (235) | USA | Self-reported/questionnaire | Second referral level (and above) | Specialist Medical Practitioners | 25.2 | - | - | - | N | N |
|  | USA | Self-reported/questionnaire | Second referral level (and above) | Specialist Medical Practitioners | 16.2 | - | - | - | N | N |
|  | USA | Self-reported/questionnaire | Second referral level (and above) | Specialist Medical Practitioners | 13.9 | - | - | - | N | N |
|  | USA | Self-reported/questionnaire | Second referral level (and above) | Specialist Medical Practitioners | 25.9 | - | - | - | Y | Y |
|  | USA | Self-reported/questionnaire | Second referral level (and above) | Specialist Medical Practitioners | 10.7 | - | - | - | Y | Y |
| Patel (236) | USA | Self-reported/questionnaire |  | Specialist Medical Practitioners | NA | NA | <5 minutes (773); 5–10 minutes (2,223); 10 minutes (6,581) | - | N | N |
|  | USA | Self-reported/questionnaire |  | General Medical Practitioners | NA | NA | <5 minutes (6,853); 5–10 minutes (19,108); 10 minutes (59,340) | - | N | N |
| Paudel (237) | Nepal | Self-reported/questionnaire | Second referral level (and above) | Specialist Medical Practitioners | 5.26 | - | - | SD, 2.31 | N | N |
|  | Nepal | Self-reported/questionnaire | Second referral level (and above) | Specialist Medical Practitioners | 2.36 | - | - | - | N | N |
|  | Nepal | Self-reported/questionnaire | Second referral level (and above) | Specialist Medical Practitioners | 4.36 | - | - | SD, 1.79 | N | N |
|  | Nepal | Self-reported/questionnaire | Second referral level (and above) | Specialist Medical Practitioners | 11.83 | - | - | - | N | N |
| Peek (238) | Australia | Self-reported/questionnaire | General outpatient services | Physiotherapists | 26 | - | - | SD, 9.22 | N | N |
| Peltzer (239) | Indonesia | Existing databases or guidelines | Community-based services | Traditional and Complementary Medicine Professionals | 60 | - | - | - | N | N |
| Perdoncini (240) | Brazil |  | General outpatient services | Dentists | 10.44 | - | - | SD, 2.88 | Y | N |
| Peters (241) | Netherlands | Recording (video, audio, stopwatch) | First referral level | Specialist Medical Practitioners | NA | 14.52 | Median was 14 minutes, 31 seconds | - | N | N |
|  | Netherlands | Recording (video, audio, stopwatch) | First referral level | Specialist Medical Practitioners | NA | 19.37 | Median was (min:sec) 19:22 | - | N | N |
|  | Netherlands | Recording (video, audio, stopwatch) | First referral level | Specialist Medical Practitioners | NA | 12.07 | Median was (min:sec) 12:04 | - | N | N |
|  | Netherlands | Recording (video, audio, stopwatch) | First referral level | Specialist Medical Practitioners | NA | 13.68 | Median was (min:sec) 13:41 | - | N | N |
| Pettersson (242) | Sweden | Recording (video, audio, stopwatch) | Second referral level (and above) | Nursing Professionals | NA | 27 | - | - | N | N |
| Peurois (243) | France | Existing databases or guidelines | General outpatient services | General Medical Practitioners | 16.7 | - | - | SD, 8.3 | N | N |
| Phadke (244) | USA | Software tracking | First referral level | Specialist Medical Practitioners | 11 | - | - | - | Y | N |
| Phommachanh (245) | Laos | Recording (video, audio, stopwatch) | Other, specify: | Nursing Professionals | 16.21 | - | - | SD, 4.28 | N | N |
| Pierse (246) | Ireland | Electronic health records | General outpatient services | General Medical Practitioners | 14.1 | - | - | SD, 10.6 | N | N |
| Pilote (247) | Canada | Recording (video, audio, stopwatch) | Second referral level (and above) | Nursing Professionals | 45 | - | - | SD, 16 | N | N |
| Pintova (248) | USA | Recording (video, audio, stopwatch) | First referral level | Specialist Medical Practitioners | 15 | - | - | - | N | N |
|  | USA | Recording (video, audio, stopwatch) | First referral level | Specialist Medical Practitioners | 15 | - | - | - | N | N |
|  | USA | Recording (video, audio, stopwatch) | First referral level | Specialist Medical Practitioners | 14 | - | - | - | N | N |
|  | USA | Recording (video, audio, stopwatch) | First referral level | Specialist Medical Practitioners | 18 | - | - | - | N | N |
|  | USA | Recording (video, audio, stopwatch) | First referral level | Specialist Medical Practitioners | 13 | - | - | - | N | N |
|  | USA | Recording (video, audio, stopwatch) | First referral level | Specialist Medical Practitioners | 10 | - | - | - | N | N |
|  | USA | Recording (video, audio, stopwatch) | First referral level | Specialist Medical Practitioners | 10 | - | - | - | N | N |
|  | USA | Recording (video, audio, stopwatch) | First referral level | Specialist Medical Practitioners | 21 | - | - | - | N | N |
|  | USA | Recording (video, audio, stopwatch) | First referral level | Specialist Medical Practitioners | 18 | - | - | - | N | N |
|  | USA | Recording (video, audio, stopwatch) | First referral level | Specialist Medical Practitioners | 9.5 | - | - | - | N | N |
|  | USA | Recording (video, audio, stopwatch) | First referral level | Specialist Medical Practitioners | 15 | - | - | - | N | N |
|  | USA | Recording (video, audio, stopwatch) | First referral level | Specialist Medical Practitioners | 15 | - | - | - | N | N |
|  | USA | Recording (video, audio, stopwatch) | First referral level | Specialist Medical Practitioners | 15 | - | - | - | N | N |
|  | USA | Recording (video, audio, stopwatch) | First referral level | Specialist Medical Practitioners | 17 | - | - | - | N | N |
|  | USA | Recording (video, audio, stopwatch) | First referral level | Specialist Medical Practitioners | 13 | - | - | - | N | N |
|  | USA | Recording (video, audio, stopwatch) | First referral level | Specialist Medical Practitioners | 9.5 | - | - | - | N | N |
|  | USA | Recording (video, audio, stopwatch) | First referral level | Specialist Medical Practitioners | 13.5 | - | - | - | N | N |
|  | USA | Recording (video, audio, stopwatch) | First referral level | Specialist Medical Practitioners | 9 | - | - | - | N | N |
|  | USA | Recording (video, audio, stopwatch) | First referral level | Specialist Medical Practitioners | 28 | - | - | - | N | N |
|  | USA | Recording (video, audio, stopwatch) | First referral level | Specialist Medical Practitioners | 9 | - | - | - | N | N |
|  | USA | Recording (video, audio, stopwatch) | First referral level | Specialist Medical Practitioners | 18 | - | - | - | N | N |
|  | USA | Recording (video, audio, stopwatch) | First referral level | Specialist Medical Practitioners | 16.5 | - | - | - | N | N |
|  | USA | Recording (video, audio, stopwatch) | First referral level | Specialist Medical Practitioners | 23 | - | - | - | N | N |
|  | USA | Recording (video, audio, stopwatch) | First referral level | Specialist Medical Practitioners | 22 | - | - | - | N | N |
|  | USA | Recording (video, audio, stopwatch) | First referral level | Specialist Medical Practitioners | 16.5 | - | - | - | N | N |
| Pochert (249) | Germany | Self-reported/questionnaire | General outpatient services | General Medical Practitioners | 14.8 | - | - | SD, 9.3; 95% CI, 14.5 - 15.1 | N | N |
|  | Germany | Self-reported/questionnaire | General outpatient services | General Medical Practitioners | 15 | - | - | SD, 8.8; 95% CI, 14.5 - 15.5 | N | N |
|  | Germany | Self-reported/questionnaire | General outpatient services | General Medical Practitioners | 16.2 | - | - | SD, 9.9; 95% CI, 15.4 - 17 | N | N |
|  | Germany | Self-reported/questionnaire | General outpatient services | General Medical Practitioners | 15.1 | - | - | SD, 9.9; 95% CI, 14.5 - 15.6 | N | N |
|  | Germany | Self-reported/questionnaire | General outpatient services | General Medical Practitioners | 12.8 | - | - | SD, 7.6; 95% CI, 12.2 - 13.3 | N | N |
| Polat (250) | USA |  | Other, specify: | Specialist Medical Practitioners | NA | 19 | - | - | Y | N |
|  | USA |  | Other, specify: | Specialist Medical Practitioners | NA | 32 | - | - | N | N |
| Popescu (251) | Canada | Recording (video, audio, stopwatch) | First referral level | General Medical Practitioners | 19.53 | - | - | SD, 6.09 | Y | Y |
|  | Canada | Recording (video, audio, stopwatch) | First referral level | General Medical Practitioners | 17.69 | - | - | SD, 10.12 | Y | Y |
|  | Canada | Recording (video, audio, stopwatch) | First referral level | General Medical Practitioners | 21.48 | - | - | SD, 10.69 | Y | Y |
| Pradhan (252) | UK | Software tracking | First referral level | Specialist Medical Practitioners | 8.9 | - | - | SD, 3.047 | Y | Y |
| Puchner (253) | Austria | Self-reported/questionnaire | First referral level | Specialist Medical Practitioners | NA | NA | Consultations lasted between 10 and 15 min' | - | N | N |
| Qiao (254) | Inner Mongolia Autonomous Region of People’s Republic of China | Self-reported/questionnaire | Second referral level (and above) | Specialist Medical Practitioners | NA | NA | ≤5 min (41.1%), 6–10 min (35.6%), >10 min (23.2%) | - | N | N |
| Rahhal (255) | USA | Electronic health records | First referral level | Specialist Medical Practitioners | NA | 22 | - | - | N | N |
|  | USA | Electronic health records | First referral level | Specialist Medical Practitioners | NA | 20 | - | - | N | N |
|  | USA | Electronic health records | First referral level | Specialist Medical Practitioners | NA | 29 | - | - | N | N |
|  | USA | Electronic health records | First referral level | Specialist Medical Practitioners | NA | 21 | - | - | N | N |
|  | USA | Electronic health records | First referral level | Specialist Medical Practitioners | NA | 18 | - | - | N | N |
|  | USA | Electronic health records | First referral level | Specialist Medical Practitioners | NA | 29 | - | - | N | N |
|  | USA | Electronic health records | First referral level | Specialist Medical Practitioners | NA | 23 | - | - | N | N |
|  | USA | Electronic health records | First referral level | Specialist Medical Practitioners | NA | 21 | - | - | N | N |
|  | USA | Electronic health records | First referral level | Specialist Medical Practitioners | NA | 29.5 | - | - | N | N |
| Rainey (256) | USA | Recording (video, audio, stopwatch) | First referral level | Specialist Medical Practitioners | NA | NA | The average length-of-visit between surgeons ranged from 9.78 to 12.38 min | - | N | N |
|  | USA | Recording (video, audio, stopwatch) | First referral level | Specialist Medical Practitioners | 5.48 | - | - | - | N | N |
|  | USA | Recording (video, audio, stopwatch) | First referral level | Specialist Medical Practitioners | 11.45 | - | - | - | N | N |
|  | USA | Recording (video, audio, stopwatch) | First referral level | Specialist Medical Practitioners | 11.8 | - | - | - | N | N |
| Rajput (257) | Pakistan | Self-reported/questionnaire | Second referral level (and above) | General Medical Practitioners | NA | NA | 10 to 15 minutes was the mean consultation time | - | N | N |
| Rao (258) | United States of America | Existing databases or guidelines | Other, specify: | General Medical Practitioners | 19.3 | - | - | - | N | N |
|  | United States of America | Existing databases or guidelines | Other, specify: | General Medical Practitioners | 21.6 | - | - | - | N | N |
| Ratwani (259) | USA | Software tracking | Other, specify: | Specialist Medical Practitioners | 8.8 | 8 | - | SD, 4.6 | Y | Y |
| Read-Brown (260) | USA | Recording (video, audio, stopwatch) | First referral level | Specialist Medical Practitioners | 11.2 | - | Note, 3.2 minutes were examination time | SD, 6.3 | N | N |
|  | USA | Recording (video, audio, stopwatch) | First referral level | Specialist Medical Practitioners | 11.4 | - | Note, 2.8 minutes were examination time | SD, 4.9 | N | N |
|  | USA | Recording (video, audio, stopwatch) | Second referral level (and above) | Specialist Medical Practitioners | 10.4 | - | Note, 3.4 minutes were examination time | SD, 6.3 | N | N |
|  | USA | Recording (video, audio, stopwatch) | Second referral level (and above) | Specialist Medical Practitioners | 10.1 | - | Note, 4.0 minutes were examination time | SD, 4.8 | N | N |
|  | USA | Recording (video, audio, stopwatch) | Second referral level (and above) | Specialist Medical Practitioners | 13.3 | - | Note, 3.4 minutes were examination time | SD, 7.3 | N | N |
|  | USA | Recording (video, audio, stopwatch) | Second referral level (and above) | Specialist Medical Practitioners | 10 | - | Note, 3.5 minutes were examination time | SD, 5.9 | N | N |
| Reblin (261) | USA | Recording (video, audio, stopwatch) | Second referral level (and above) | Nursing Professionals | 40.2 | - | - | SD, 20.69 | N | N |
| Rej (262) | UK | Self-reported/questionnaire | Second referral level (and above) | Dieticians and Nutritionists | NA | NA | <15min (3%); 15–30min (43%); 30–45min (41%); 45–60min (14%) | - | N | N |
|  | UK | Self-reported/questionnaire | Second referral level (and above) | Dieticians and Nutritionists | NA | NA | <15min (0%); 15–30min (44%); 30–45min (42%); 45–60min (14%) | - | N | N |
|  | UK | Self-reported/questionnaire | Second referral level (and above) | Dieticians and Nutritionists | NA | NA | <15min (0%); 15–30min (54%); 30–45min (37%); 45–60min (9%) | - | N | N |
| Riley (263) | USA | Electronic health records | Second referral level (and above) | Specialist Medical Practitioners | 23.36 | - | - | SD, 10.94 | N | N |
| Roll (264) | USA | Self-reported/questionnaire | General outpatient services | Pharmacists | NA | NA | < 15 minutes (n=17); 15-30 minutes (n=124); > 30 minutes (n=92) | - | N | N |
|  | USA | Self-reported/questionnaire | General outpatient services | Pharmacists | NA | NA | < 15 minutes (n=8); 15-30 minutes (n=75); > 30 minutes (n=72) | - | N | N |
| Rutten (265) | Netherlands | Self-reported/questionnaire | General outpatient services | Nursing Professionals | NA | NA | Duration of conversation ,25 min (56.9%) | - | N | N |
|  | Netherlands | Self-reported/questionnaire | General outpatient services | General Medical Practitioners | NA | NA | Duration of conversation ,25 min (72.8%) | - | N | N |
|  | Netherlands | Self-reported/questionnaire | General outpatient services | General Medical Practitioners | NA | NA | Duration of conversation ,25 min (80.4%) | - | N | N |
|  | Netherlands | Self-reported/questionnaire | General outpatient services | General Medical Practitioners | NA | NA | Duration of conversation ,25 min (72.0%) | - | N | N |
|  | Netherlands | Self-reported/questionnaire | Second referral level (and above) | Specialist Medical Practitioners | NA | NA | Duration of conversation ,25 min (65.6%) | - | N | N |
| Ryan (266) | Australia | Software tracking | Second referral level (and above) | Pharmacists | 15.8 | - | - | SD, 8.5 | Y | N |
|  | Australia | Software tracking | Second referral level (and above) | Pharmacists | 13.5 | - | - | SD, 6.8 | Y | N |
| Ryu (267) | USA | Self-reported/questionnaire | Second referral level (and above) | Health Professionals not elsewhere classified | NA | NA | <15 minutes (0); 15–30 minutes (3); 30–60 minutes (1) | - | Y | Y |
|  | USA | Self-reported/questionnaire | Second referral level (and above) | Specialist Medical Practitioners | NA | NA | <15 minutes (2); 15–30 minutes (8); 30–60 minutes (0) | - | Y | Y |
| Rzadkiewicz (268) | Poland | Existing databases or guidelines | General outpatient services | General Medical Practitioners | 15.83 | - | - | SD, 5.81 | N | N |
| Abbas (269) | Pakistan | Survey | General outpatient services |  | 10 |  |  | - |  |  |
|  | Pakistan | Survey | General outpatient services |  | 15 |  |  | - |  |  |
|  | Pakistan | Survey | General outpatient services |  | 5 |  |  | - |  |  |
| Saginela (270) | India | Self-reported/questionnaire | Second referral level (and above) | Specialist Medical Practitioners | NA | NA | > 15 mins (55.1%); 15-30 mins (32.7%); >30 minutes (12.2%) | - | N | N |
| Salisbury (271) | UK | Existing databases or guidelines | General outpatient services | General Medical Practitioners | 10.49 | - | - | - | N | N |
|  | UK | Existing databases or guidelines | General outpatient services | General Medical Practitioners | 9.64 | - | - | - | N | N |
| Sanders-Pinheiro (272) | Brazil | Self-reported/questionnaire | Second referral level (and above) | Health Professionals not elsewhere classified | NA | NA | 15 min (31.5%); 30 min (68.5%) | - | N | N |
|  | Brazil | Self-reported/questionnaire | Second referral level (and above) | Health Professionals not elsewhere classified | NA | NA | 16 min (35.3%); 30 min (64.7%) | - | N | N |
|  | Brazil | Self-reported/questionnaire | Second referral level (and above) | Health Professionals not elsewhere classified | NA | NA | 17 min (25.7%); 30 min (74.3%) | - | N | N |
| Cairns (273) | USA | Existing databases or guidelines | First referral level | Specialist Medical Practitioners | 23.5 | 19.4 | Reported as Time spent with the physician: 1–5 minutes (5,548); 6–10 minutes (81,968); 11–15 minutes (314,521); 16–30 minutes (464,032); 31–60 minutes (144,688); 61 minutes and over (14,029) | - | N | N |
|  | USA | Existing databases or guidelines | First referral level | General Medical Practitioners | 21.3 | 19.1 | - | - | N | N |
|  | USA | Existing databases or guidelines | First referral level | Specialist Medical Practitioners | 23.8 | 19.6 | - | - | N | N |
|  | USA | Existing databases or guidelines | First referral level | Specialist Medical Practitioners | 27 | 22.4 | - | - | N | N |
| Santo (274) | USA | Existing databases or guidelines | General outpatient services | General Medical Practitioners | NA | NA | Reported as Time spent with the physician: 1–5 minutes (140); 6–10 minutes (1819); 11–15 minutes (14767); 16–30 minutes (9954); 31–60 minutes (1488); 61 minutes and over (62) | - | N | N |
|  | USA | Existing databases or guidelines | General outpatient services | Health Professionals not elsewhere classified | NA | NA | Reported as Time spent with non-physician (classified as physician assistant, nurse practitioner or nurse midwife): 1–5 minutes (122); 6–10 minutes (1141); 11–15 minutes (10478); 16–30 minutes (7450); 31–60 minutes (1092); 61 minutes and over (63) | - | N | N |
|  | USA | Existing databases or guidelines | General outpatient services | Specialist Medical Practitioners | 22.8 | 14.4 | - | - | N | N |
|  | USA | Existing databases or guidelines | General outpatient services | Specialist Medical Practitioners | 34.3 | 19.9 | - | - | N | N |
|  | USA | Existing databases or guidelines | General outpatient services | Specialist Medical Practitioners | 28.9 | 15 | - | - | N | N |
|  | USA | Existing databases or guidelines | General outpatient services | Specialist Medical Practitioners | 28.8 | 14.5 | - | - | N | N |
|  | USA | Existing databases or guidelines | General outpatient services | Specialist Medical Practitioners | 22.8 | 14.4 | - | - | N | N |
|  | USA | Existing databases or guidelines | General outpatient services | Specialist Medical Practitioners | 22.6 | 14.5 | - | - | N | N |
|  | USA | Existing databases or guidelines | General outpatient services | Specialist Medical Practitioners | 22.2 | 14.5 | - | - | N | N |
|  | USA | Existing databases or guidelines | General outpatient services | Specialist Medical Practitioners | 22.1 | 14.3 | - | - | N | N |
|  | USA | Existing databases or guidelines | General outpatient services | Specialist Medical Practitioners | 21.7 | 14.3 | - | - | N | N |
|  | USA | Existing databases or guidelines | General outpatient services | Specialist Medical Practitioners | 21.7 | 14.4 | - | - | N | N |
|  | USA | Existing databases or guidelines | General outpatient services | Specialist Medical Practitioners | 21.1 | 14.3 | - | - | N | N |
|  | USA | Existing databases or guidelines | General outpatient services | General Medical Practitioners | 20.3 | 14.1 | - | - | N | N |
|  | USA | Existing databases or guidelines | General outpatient services | Specialist Medical Practitioners | 20.1 | 14.2 | - | - | N | N |
|  | USA | Existing databases or guidelines | General outpatient services | Specialist Medical Practitioners | 17.9 | 11.5 | - | - | N | N |
|  | USA | Existing databases or guidelines | General outpatient services | Specialist Medical Practitioners | 24 | 14.5 | - | - | N | N |
| Saunders (275) | Nicaragua |  | General outpatient services | Community Health Workers | 5.8 | - | - | SD, 2.1 | N | N |
|  | Nicaragua |  | General outpatient services | Community Health Workers | 4.3 | - | - | SD, 1.5 | N | N |
| Schäfer (276) | Austria | Existing databases or guidelines | General outpatient services | General Medical Practitioners | 11.7 | - | - | SD, 12.4 | N | N |
|  | Belgium | Existing databases or guidelines | General outpatient services | General Medical Practitioners | 17.7 | - | - | SD, 5.9 | N | N |
|  | Bulgaria | Existing databases or guidelines | General outpatient services | General Medical Practitioners | 17.6 | - | - | SD, 6 | N | N |
|  | Canada | Existing databases or guidelines | General outpatient services | General Medical Practitioners | 14.8 | - | - | SD, 9.2 | N | N |
|  | Cyprus | Existing databases or guidelines | General outpatient services | General Medical Practitioners | 18.3 | - | - | SD, 9 | N | N |
|  | Czech Republic | Existing databases or guidelines | General outpatient services | General Medical Practitioners | 10.9 | - | - | SD, 4.8 | N | N |
|  | Denmark | Existing databases or guidelines | General outpatient services | General Medical Practitioners | 14.3 | - | - | SD, 2.1 | N | N |
|  | England | Existing databases or guidelines | General outpatient services | General Medical Practitioners | 11.2 | - | - | SD, 2.1 | N | N |
|  | Estonia | Existing databases or guidelines | General outpatient services | General Medical Practitioners | 16.4 | - | - | SD, 3.8 | N | N |
|  | Finland | Existing databases or guidelines | General outpatient services | General Medical Practitioners | 23.8 | - | - | SD, 6.3 | N | N |
|  | FYR Macedonia | Existing databases or guidelines | General outpatient services | General Medical Practitioners | 13.4 | - | - | SD, 4.8 | N | N |
|  | Germany | Existing databases or guidelines | General outpatient services | General Medical Practitioners | 10.6 | - | - | SD, 4 | N | N |
|  | Greece | Existing databases or guidelines | General outpatient services | General Medical Practitioners | 14.7 | - | - | SD, 6.3 | N | N |
|  | Hungary | Existing databases or guidelines | General outpatient services | General Medical Practitioners | 8.2 | - | - | SD, 5.5 | N | N |
|  | Iceland | Existing databases or guidelines | General outpatient services | General Medical Practitioners | 19.2 | - | - | SD, 2.7 | N | N |
|  | Ireland | Existing databases or guidelines | General outpatient services | General Medical Practitioners | 12.8 | - | - | SD, 3.3 | N | N |
|  | Italy | Existing databases or guidelines | General outpatient services | General Medical Practitioners | 13.4 | - | - | SD, 4 | N | N |
|  | Latvia | Existing databases or guidelines | General outpatient services | General Medical Practitioners | 17.5 | - | - | SD, 6.6 | N | N |
|  | Lithuania | Existing databases or guidelines | General outpatient services | General Medical Practitioners | 15.9 | - | - | SD, 4.2 | N | N |
|  | Luxembourg | Existing databases or guidelines | General outpatient services | General Medical Practitioners | 17.6 | - | - | SD, 5 | N | N |
|  | Malta | Existing databases or guidelines | General outpatient services | General Medical Practitioners | 13 | - | - | SD, 4.2 | N | N |
|  | Netherlands | Existing databases or guidelines | General outpatient services | General Medical Practitioners | 11.1 | - | - | SD, 1.7 | N | N |
|  | New Zealand | Existing databases or guidelines | General outpatient services | General Medical Practitioners | 14.9 | - | - | SD, 3.9 | N | N |
|  | Norway | Existing databases or guidelines | General outpatient services | General Medical Practitioners | 18.6 | - | - | SD, 2.8 | N | N |
|  | Poland | Existing databases or guidelines | General outpatient services | General Medical Practitioners | 13.7 | - | - | SD, 5.1 | N | N |
|  | Portugal | Existing databases or guidelines | General outpatient services | General Medical Practitioners | 18.1 | - | - | SD, 6.6 | N | N |
|  | Romania | Existing databases or guidelines | General outpatient services | General Medical Practitioners | 16.6 | - | - | SD, 5.1 | N | N |
|  | Slovakia | Existing databases or guidelines | General outpatient services | General Medical Practitioners | 8.9 | - | - | SD, 3.5 | N | N |
|  | Slovenia | Existing databases or guidelines | General outpatient services | General Medical Practitioners | 9.6 | - | - | SD, 3.1 | N | N |
|  | Spain | Existing databases or guidelines | General outpatient services | General Medical Practitioners | 8.5 | - | - | SD, 4.5 | N | N |
|  | Sweden | Existing databases or guidelines | General outpatient services | General Medical Practitioners | 23.9 | - | - | SD, 5.5 | N | N |
|  | Switzerland | Existing databases or guidelines | General outpatient services | General Medical Practitioners | 19.5 | - | - | SD, 5.8 | N | N |
|  | Turkey | Existing databases or guidelines | General outpatient services | General Medical Practitioners | 9.3 | - | - | SD, 5.6 | N | N |
| Schifeling (277) | USA | Electronic health records | General outpatient services | Specialist Medical Practitioners | 33.6 | - | - | SD, 10.4 | N | Y |
|  | USA | Electronic health records | General outpatient services | Specialist Medical Practitioners | 30.3 | - | - | SD, 9.7 | N | Y |
|  | USA | Electronic health records | General outpatient services | Specialist Medical Practitioners | 37.3 | - | - | SD, 10 | N | Y |
| Schimmelfing (278) | USA | Self-reported/questionnaire | Community-based services | Pharmacists | 6 | - | - | - | N | N |
| Schnakenberg (279) | Germany | Self-reported/questionnaire | General outpatient services | General Medical Practitioners | 20.7 | - | - | SD, 12.2 | N | N |
|  | Germany | Self-reported/questionnaire | General outpatient services | General Medical Practitioners | 15.8 | - | - | SD, 10.6 | N | N |
| Sema (280) | Ethiopia | Self-reported/questionnaire | First referral level | Pharmacists | 5.35 | - | - | SD, 0.31 | N | N |
| Sfar-Gandoura (281) | UK | Electronic health records | First referral level | Nursing Professionals | 34.46 | - | - | SD, 13.95 | N | N |
| Shalihin (282) | Malaysia | Self-reported/questionnaire | General outpatient services | Pharmacists | 7.81 | - | - | SD, 2.86 | N | N |
|  | Malaysia | Self-reported/questionnaire | General outpatient services | Pharmacists | 7.2 | - | - | SD, 5.01 | N | N |
|  | Malaysia | Self-reported/questionnaire | General outpatient services | General Medical Practitioners | 10.29 | - | - | SD, 9.22 | N | N |
|  | Malaysia | Self-reported/questionnaire | General outpatient services | General Medical Practitioners | 9.33 | - | - | SD, 7.75 | N | N |
| Shapiro (283) | USA | Existing databases or guidelines | First referral level | Health Professionals not elsewhere classified | 64.5 | - | - | 95% CI, 61.2 - 67.7 | N | N |
|  | USA | Existing databases or guidelines | First referral level | Health Professionals not elsewhere classified | 68.6 | - | - | 95% CI, 64.6 - 72.6 | N | N |
|  | USA | Existing databases or guidelines | First referral level | Specialist Medical Practitioners | 19.8 | - | - | 95% CI, 18.7 - 20.9 | N | N |
|  | USA | Existing databases or guidelines | First referral level | Specialist Medical Practitioners | 20.1 | - | - | 95% CI, 19.2 - 21.1 | N | N |
| Shapiro (284) | USA | Self-reported/questionnaire |  | Specialist Medical Practitioners | 36.9 | - | - | SD, 12.6 | N | N |
| Shen (285) | China | Electronic health records | First referral level | Specialist Medical Practitioners | 6.52 | - | - | - | N | N |
|  | China | Electronic health records | First referral level | Specialist Medical Practitioners | 3.15 | - | - | - | N | N |
| Shim (286) | USA | Existing databases or guidelines | Second referral level (and above) | Specialist Medical Practitioners | NA | 12.65 | 12 minutes and 39 seconds | - | Y | Y |
| Shuaib (287) | USA | Electronic health records | First referral level | Specialist Medical Practitioners | 4 | - | It states: 'An average visit lasted 25.9min in the prescribe cohort and post-scribe visits averaged 23.2min' but this is not necessarily time with the patient | SD, 0.57 | N | N |
|  | USA | Electronic health records | First referral level | Specialist Medical Practitioners | 1.2 | - | It states: 'An average visit lasted 25.9min in the prescribe cohort and post-scribe visits averaged 23.2min' but this is not necessarily time with the patient | SD, 0.27 | N | N |
|  | USA | Electronic health records | First referral level | Specialist Medical Practitioners | 7.8 | - | It states: 'An average visit lasted 25.9min in the prescribe cohort and post-scribe visits averaged 23.2min' but this is not necessarily time with the patient | SD, 1.2 | N | N |
|  | USA | Electronic health records | First referral level | Specialist Medical Practitioners | 1.45 | - | It states: 'An average visit lasted 25.9min in the prescribe cohort and post-scribe visits averaged 23.2min' but this is not necessarily time with the patient | SD, 0.44 | N | N |
| Siele (288) | Eritrea | Recording (video, audio, stopwatch) | Second referral level (and above) | Pharmacists | 5.46 | - | - | - | N | N |
|  | Eritrea | Recording (video, audio, stopwatch) | Second referral level (and above) | Pharmacists | 2.67 | - | - | - | N | N |
|  | Eritrea | Recording (video, audio, stopwatch) | Second referral level (and above) | Pharmacists | 3.47 | - | - | - | N | N |
|  | Eritrea | Recording (video, audio, stopwatch) | Second referral level (and above) | Pharmacists | 3.9 | - | - | - | N | N |
|  | Eritrea | Recording (video, audio, stopwatch) | Second referral level (and above) | Pharmacists | 5.29 | - | - | - | N | N |
|  | Eritrea | Recording (video, audio, stopwatch) | Second referral level (and above) | Pharmacists | 5.7 | - | - | - | N | N |
|  | Eritrea | Recording (video, audio, stopwatch) | Second referral level (and above) | Pharmacists | 6.45 | - | - | - | N | N |
|  | Eritrea | Recording (video, audio, stopwatch) | Second referral level (and above) | Pharmacists | 7.31 | - | - | - | N | N |
|  | Eritrea | Recording (video, audio, stopwatch) | Second referral level (and above) | Pharmacists | 8.9 | - | - | - | N | N |
| Silver (289) | USA | Self-reported/questionnaire | First referral level | General Medical Practitioners | 17.11 | - | - | - | Y | Y |
|  | USA | Self-reported/questionnaire | First referral level | General Medical Practitioners | 14.51 | - | - | - | Y | Y |
|  | USA | Self-reported/questionnaire | First referral level | General Medical Practitioners | 14.88 | - | Note these are the results for all patients in cycle 4 of a 4 cycle quality improvement project. The results of previous cycles are cycle 1 (14.35), cycle 2 (14.39) and cycle 3 (14.69) | SD, 8.69 | Y | Y |
| Singh (290) | India | Self-reported/questionnaire | First referral level | Specialist Medical Practitioners | 2.8 | - | - | - | N | N |
| Sinha (291) | USA | Self-reported/questionnaire | Second referral level (and above) | Specialist Medical Practitioners | 3.75 | - | 3 minutes 45 seconds | - | Y | N |
| Sisay (292) | Ethiopia | Recording (video, audio, stopwatch) | First referral level | Pharmacists | 4.61 | - | Note, assuming the SD is in seconds although it does not specify | SD, 191.58 | N | N |
|  | Ethiopia | Recording (video, audio, stopwatch) | First referral level | Pharmacists | 4.27 | - | Note, assuming the SD is in seconds although it does not specify | SD, 117.64 | N | N |
|  | Ethiopia | Recording (video, audio, stopwatch) | First referral level | Pharmacists | 6.36 | - | Note, assuming the SD is in seconds although it does not specify | SD, 266.22 | N | N |
|  | Ethiopia | Recording (video, audio, stopwatch) | First referral level | Pharmacists | 3.2 | - | Note, assuming the SD is in seconds although it does not specify | SD, 83.88 | N | N |
| Skrepnek (293) | USA | Existing databases or guidelines | First referral level | Specialist Medical Practitioners | 26.1 | - | - | SD, 26.1 | N | N |
|  | USA | Existing databases or guidelines | First referral level | Specialist Medical Practitioners | 22.8 | - | - | SD, 20.5 | N | N |
|  | USA | Existing databases or guidelines | First referral level | Specialist Medical Practitioners | 21.6 | - | - | SD, 13.6 | N | N |
|  | USA | Existing databases or guidelines | First referral level | Specialist Medical Practitioners | 21.7 | - | - | SD, 13.5 | N | N |
| Smits (294) | Netherlands | Electronic health records | General outpatient services | General Medical Practitioners | 21.1 | - | - | SD, 10.4; 95% CI, 20.4 - 21.6 | N | N |
|  | Netherlands | Electronic health records | General outpatient services | Nursing Professionals | 34.1 | - | - | SD, 13.8; 95% CI, 31.9 - 34.8 | N | N |
| Soegaard (295) | USA | Software tracking | First referral level | Specialist Medical Practitioners | 8.6 | - | - | SD, 3.9 | Y | N |
| Søndergaard (296) | Denmark | Recording (video, audio, stopwatch) | Second referral level (and above) | Specialist Medical Practitioners | 22.08 | - | mean = 22 min, 5 s; range = 3 min, 23 s to 63 min, 44 s; SD = 13 min, 13 s | SD, 13.22 | N | N |
|  | Denmark | Recording (video, audio, stopwatch) | Second referral level (and above) | Specialist Medical Practitioners | 24.26 | - | Mean = 24 min, 16 s; range = 7 min, 2 s and the longest 38 min, 20 s; SD = 7 min, 23 s | SD, 7.38 | N | N |
|  | Denmark | Recording (video, audio, stopwatch) | Second referral level (and above) | Specialist Medical Practitioners | 34.67 | - | Mean = 34 min, 40 s | - | N | N |
|  | Denmark | Recording (video, audio, stopwatch) | Second referral level (and above) | Specialist Medical Practitioners | 39.97 | - | Mean = 39 min, 58 s | - | N | N |
|  | Denmark | Recording (video, audio, stopwatch) | Second referral level (and above) | Specialist Medical Practitioners | 27.03 | - | Mean = 27 min, 2 s; | - | N | N |
|  | Denmark | Recording (video, audio, stopwatch) | Second referral level (and above) | Specialist Medical Practitioners | 25.42 | - | Mean = 25 min, 25 s; | - | N | N |
|  | Denmark | Recording (video, audio, stopwatch) | Second referral level (and above) | Specialist Medical Practitioners | 15.42 | - | Mean = 15 min, 25 s | - | N | N |
|  | Denmark | Recording (video, audio, stopwatch) | Second referral level (and above) | Specialist Medical Practitioners | 17.93 | - | Mean = 17 min, 56 s | - | N | N |
| Stark (297) | USA | Existing databases or guidelines | First referral level | Specialist Medical Practitioners | 21.8 | - | - | - | N | N |
| Stevens (298) | UK | Existing databases or guidelines | General outpatient services | Nursing Professionals | 9.7 | - | - | SD, 9.21 | N | N |
|  | UK | Existing databases or guidelines | General outpatient services | Nursing Professionals | 5.73 | - | - | SD, 6.29 | Y | N |
|  | UK | Existing databases or guidelines | General outpatient services | General Medical Practitioners | 9.24 | - | - | SD, 8.06 | N | N |
|  | UK | Existing databases or guidelines | General outpatient services | General Medical Practitioners | 5.32 | - | - | SD, 6.21 | Y | N |
| Stewart (299) | USA | Self-reported/questionnaire | General outpatient services | Pharmacists | 58.7 | - | - | SD, 21.9 | N | N |
| Stewart (300) | USA | Software tracking | Second referral level (and above) | Specialist Medical Practitioners | 54 | - | - | - | Y | N |
|  | USA | Software tracking | Second referral level (and above) | Specialist Medical Practitioners | 33 | - | - | - | Y | N |
|  | USA | Software tracking | Second referral level (and above) | Optometrists and Ophthalmic Opticians | 80 | - | - | - | Y | N |
|  | USA | Software tracking | Second referral level (and above) | Optometrists and Ophthalmic Opticians | 43 | - | - | - | Y | N |
| Stewart (301) | Canada | Recording (video, audio, stopwatch) | Second referral level (and above) | Medical Imaging and Therapeutic Equipment Technicians | 21.73 | - | 21:44 | - | N | N |
|  | Canada | Recording (video, audio, stopwatch) | Second referral level (and above) | Medical Imaging and Therapeutic Equipment Technicians | 13.6 | - | 13:36 | - | N | N |
| Stime (302) | South Africa | Recording (video, audio, stopwatch) | First referral level |  | 2 | 3 | - | SD, 1 | N | N |
|  | South Africa | Recording (video, audio, stopwatch) | First referral level | Nursing Professionals | 7 | 6 | - | SD, 4 | N | N |
|  | South Africa | Recording (video, audio, stopwatch) | First referral level | Nursing Professionals | 169 | 172 | - | SD, 28 | N | N |
|  | South Africa | Recording (video, audio, stopwatch) | First referral level | Nursing Professionals | 7 | 7 | - | SD, 3 | N | N |
|  | South Africa | Recording (video, audio, stopwatch) | First referral level | Nursing Professionals | 7 | 8 | - | SD, 3 | N | N |
|  | South Africa | Recording (video, audio, stopwatch) | First referral level | Nursing Professionals | 9 | 8 | - | SD, 4 | N | N |
| Sumargono (303) | Indonesia | Recording (video, audio, stopwatch) | First referral level | Specialist Medical Practitioners | 9.35 | - | 9 minutes 21 seconds | - | Y | Y |
| Swami (304) | Australia | Existing databases or guidelines | General outpatient services | General Medical Practitioners | 15.82 | - | - | SD, 3.99 | N | N |
| Talyshinskii (305) | - | Electronic health records |  | Specialist Medical Practitioners | 10.9 | - | - | SD, 0.6 | N | N |
|  | - | Electronic health records |  | Specialist Medical Practitioners | 7.3 | - | - | SD, 0.5 | N | N |
| Tampi (306) | Zambia | Recording (video, audio, stopwatch) | First referral level | Specialist Medical Practitioners | NA | 3 | - | - | N | N |
|  | Zambia | Recording (video, audio, stopwatch) | First referral level | Social work and counselling professionals | NA | 4 | - | - | N | N |
|  | Zambia | Recording (video, audio, stopwatch) | First referral level | Nursing Professionals | NA | 3 | - | - | N | N |
|  | Zambia | Recording (video, audio, stopwatch) | First referral level | Pharmaceutical Technicians and Assistants | NA | 2 | - | - | N | N |
| Tassew (307) | Ethiopia | Recording (video, audio, stopwatch) | General outpatient services | General Medical Practitioners | 6.6 | - | - | SD, 3.5 | N | N |
|  | Ethiopia | Recording (video, audio, stopwatch) | General outpatient services | General Medical Practitioners | 3.66 | - | - | SD, 2.2 | N | N |
|  | Ethiopia | Recording (video, audio, stopwatch) | General outpatient services | General Medical Practitioners | 9.5 | - | - | SD, 4.8 | N | N |
| Taylor (308) | UK | Recording (video, audio, stopwatch) | First referral level | Nursing Professionals | NA | 29 | - | - | N | N |
|  | UK | Recording (video, audio, stopwatch) | First referral level | Nursing Professionals | NA | 12 | - | - | N | N |
| Teklemariam (309) | Ethiopia | Recording (video, audio, stopwatch) | First referral level | Pharmacists | 4.9 | - | - | - | N | N |
|  | Ethiopia | Recording (video, audio, stopwatch) | First referral level | Pharmacists | 4.9 | - | - | - | N | N |
|  | Ethiopia | Recording (video, audio, stopwatch) | First referral level | Pharmacists | 5.7 | - | - | - | N | N |
|  | Ethiopia | Recording (video, audio, stopwatch) | First referral level | Pharmacists | 4.3 | - | - | - | N | N |
| Tenforde (310) | USA | Self-reported/questionnaire | First referral level | Physiotherapists | NA | NA | 0-14 mins, 1 (0.5%); 15-29, 22 (10.7%); 30-44, 122 (59.5%); 45-59, 54 (26.3%); 60+, 6 (2.9%) | - | Y | Y |
|  | USA | Self-reported/questionnaire | First referral level | Specialist Medical Practitioners | NA | NA | 5-14 min, 14 (11.8%); 15–29 min, 67 (56.3%); 30-60 min, 34 (28.6%); 60+ min, 4 (3.4%) | - | Y | Y |
| Thapa (311) | India | Recording (video, audio, stopwatch) | Second referral level (and above) | Specialist Medical Practitioners | 6 | 5 | - | - | N | N |
|  | India | Recording (video, audio, stopwatch) | Second referral level (and above) | Specialist Medical Practitioners | 6.84 | 5 | - | - | N | N |
|  | India | Recording (video, audio, stopwatch) | Second referral level (and above) | Specialist Medical Practitioners | 9.6 | 7 | - | - | N | N |
|  | India | Recording (video, audio, stopwatch) | Second referral level (and above) | Specialist Medical Practitioners | 7.24 | 6 | - | - | N | N |
|  | India | Recording (video, audio, stopwatch) | Second referral level (and above) | Specialist Medical Practitioners | 6.04 | 5 | - | - | N | N |
|  | India | Recording (video, audio, stopwatch) | Second referral level (and above) | Specialist Medical Practitioners | 6.36 | 5 | - | - | N | N |
|  | India | Recording (video, audio, stopwatch) | Second referral level (and above) | Specialist Medical Practitioners | 26.92 | 25 | - | - | N | N |
|  | India | Recording (video, audio, stopwatch) | Second referral level (and above) | Specialist Medical Practitioners | 6.72 | 6 | - | - | N | N |
|  | India | Recording (video, audio, stopwatch) | Second referral level (and above) | Specialist Medical Practitioners | 8.88 | 8 | - | - | N | N |
|  | India | Recording (video, audio, stopwatch) | Second referral level (and above) | Specialist Medical Practitioners | 5.8 | 4 | - | - | N | N |
|  | India | Recording (video, audio, stopwatch) | Second referral level (and above) | Specialist Medical Practitioners | 6.72 | 4 | - | - | N | N |
|  | India | Recording (video, audio, stopwatch) | Second referral level (and above) | Specialist Medical Practitioners | 37.8 | 35 | - | - | N | N |
|  | India | Recording (video, audio, stopwatch) | Second referral level (and above) | Specialist Medical Practitioners | 76.84 | 75 | - | - | N | N |
|  | India | Recording (video, audio, stopwatch) | Second referral level (and above) | Specialist Medical Practitioners | 31.6 | 33 | - | - | N | N |
| Thotam (312) | USA | Self-reported/questionnaire | Second referral level (and above) | Nursing Professionals | 27 | - | - | - | N | N |
|  | USA | Self-reported/questionnaire | Second referral level (and above) | Specialist Medical Practitioners | 26 | - | - | - | N | N |
| Tilburt (313) | USA | Recording (video, audio, stopwatch) | Second referral level (and above) | Specialist Medical Practitioners | 23.3 | - | Note, the complementary or alternative medicine portion of the consultation was 77.8 seconds. | SD, 15.3 | N | N |
| Tilburt (314) | USA | Recording (video, audio, stopwatch) | Second referral level (and above) | Specialist Medical Practitioners | 28.4 | - | - | - | N | N |
|  | USA | Recording (video, audio, stopwatch) | Second referral level (and above) | Specialist Medical Practitioners | 25.6 | - | - | - | N | N |
|  | USA | Recording (video, audio, stopwatch) | Second referral level (and above) | Specialist Medical Practitioners | 23.3 | - | - | - | N | N |
|  | USA | Recording (video, audio, stopwatch) | Second referral level (and above) | Specialist Medical Practitioners | 22.4 | - | - | - | N | N |
| Timmer (315) | Germany, Austria | Self-reported/questionnaire | Other, specify: | Specialist Medical Practitioners | NA | NA | <10 min (6.6%) +30 min (21.3%) | - | N | N |
|  | Germany, Austria | Self-reported/questionnaire | Other, specify: | Specialist Medical Practitioners | NA | 15 | IQR 15 to 25 | - | N | N |
| Ting (316) | Australia | Recording (video, audio, stopwatch) | Second referral level (and above) | Specialist Medical Practitioners | 12.28 | - | 12:17 | SD, 8.2 | N | N |
|  | Australia | Recording (video, audio, stopwatch) | Second referral level (and above) | Specialist Medical Practitioners | 17.65 | - | 17:39 | SD, 8.27 | N | N |
|  | Australia | Recording (video, audio, stopwatch) | Second referral level (and above) | Specialist Medical Practitioners | 8.35 | - | 08:21 | SD, 4.8 | N | N |
|  | Australia | Recording (video, audio, stopwatch) | Second referral level (and above) | Specialist Medical Practitioners | 9.08 | - | 09:05 | SD, 6.43 | N | N |
|  | Australia | Recording (video, audio, stopwatch) | Second referral level (and above) | Specialist Medical Practitioners | 15.37 | - | 15:22 | SD, 9.13 | N | N |
| Tiruneh (317) | Ethiopia (Addis Abeba) | Self-reported/questionnaire | Other, specify: | General Medical Practitioners | NA | NA | <=10 min (56.4%) >10 min (43.6%) | - | N | N |
| Tornero-Molina (318) | Spain |  |  | Specialist Medical Practitioners | 9.64 |  | - | - | Y | Y |
| Tranter‐Entwistle(319) | New Zealand | Recording (video, audio, stopwatch) | Second referral level (and above) | Health Professionals not elsewhere classified | 3.97 | - | 3 min 58 seconds | - | N | N |
|  | New Zealand | Recording (video, audio, stopwatch) | Second referral level (and above) | Health Professionals not elsewhere classified | 3.8 | - | 3 min 48 seconds (note, there was a second post-intervention checklist assessment and that was 4 min 30 seconds) | - | N | N |
| Tsvyakh (320) | Ukraine |  | Second referral level (and above) | Specialist Medical Practitioners | 2.3 | - | - | SD, 0.4 | Y | N |
|  | Ukraine |  | Second referral level (and above) | Specialist Medical Practitioners | 12.6 | - | - | SD, 2.9 | N | N |
| Van Dril (321) | USA | Recording (video, audio, stopwatch) | First referral level | Pharmacists | 19.4 | - | Note, the results were presented as before, during and after visits & overall. I have extracted 'during' | - | N | N |
|  | USA | Recording (video, audio, stopwatch) | First referral level | Pharmacists | 21.2 | - | Note, the results were presented as before, during and after visits & overall. I have extracted 'during' | - | N | N |
|  | USA | Recording (video, audio, stopwatch) | First referral level | Pharmacists | 24.6 | - | Note, the results were presented as before, during and after visits & overall. I have extracted 'during' | - | N | N |
| Van Veenendaal (322) | Netherlands | Recording (video, audio, stopwatch) | First referral level | Specialist Medical Practitioners | 47.7 | - | - | SD, 30.5 | N | N |
| Vedanthan (323) | Kenya | Recording (video, audio, stopwatch) | Other, specify: | Nursing Professionals | 8.9 | - | - | - | N | N |
| Vilendrer (324) | USA | Existing databases or guidelines | First referral level | Nursing Professionals | 6.6 | - | - | SD, 13.6 | Y | Y |
|  | USA | Existing databases or guidelines | First referral level | Nursing Professionals | 2.3 | - | AM - 2.3; PM - 2.0 | SD, 0.3 | N | Y |
|  | USA | Existing databases or guidelines | First referral level | Nursing Professionals | 8.5 | - | AM - 8.5; PM - 6.8 | SD, 2.6 | N | Y |
|  | USA | Existing databases or guidelines | First referral level | Nursing Professionals | 2.4 | - | AM - 2.4; PM - 2.1 | SD, 0.2 | N | Y |
|  | USA | Existing databases or guidelines | First referral level | Nursing Professionals | 2.3 | - | AM - 2.3; PM - 2.1 | SD, 0.2 | N | Y |
| Vogt (325) | USA | Existing databases or guidelines | Second referral level (and above) | Specialist Medical Practitioners | 15 | - | - | - | Y | Y |
| von dem Knesebeck (326) | Germany | Self-reported/questionnaire | General outpatient services | General Medical Practitioners | 16.2 | - | - | SD, 6.9 | N | N |
|  | Germany | Self-reported/questionnaire | General outpatient services | General Medical Practitioners | 8.9 | - | - | SD, 3.9 | N | N |
|  | Germany | Self-reported/questionnaire | General outpatient services | General Medical Practitioners | 11.1 | - | - | SD, 5.3 | N | N |
|  | Germany | Self-reported/questionnaire | General outpatient services | General Medical Practitioners | 5.7 | - | - | SD, 3.2 | N | N |
|  | Germany | Self-reported/questionnaire | General outpatient services | General Medical Practitioners | 19.7 | - | - | SD, 8.9 | N | N |
|  | Germany | Self-reported/questionnaire | General outpatient services | General Medical Practitioners | 18 | - | - | SD, 8.6 | N | N |
| Vyas (327) | USA | Software tracking | Other, specify: | Specialist Medical Practitioners | 12.17 | - | 12 min 10 s | - | Y | N |
| Waller (328) | Australia | Recording (video, audio, stopwatch) | Second referral level (and above) | Midwifery Professionals | 52.07 | - | Uncertainty reported as 46.86–57.28 mins - assuming this is confidence interval but unsure | - | N | N |
| Walling (329) | USA | Recording (video, audio, stopwatch) | Second referral level (and above) | Nursing Professionals | 56.4 | - | - | - | N | N |
| Wang (330) | USA | Recording (video, audio, stopwatch) | Community-based services | Pharmacists | 24.8 | - | - | - | N | N |
|  | USA | Recording (video, audio, stopwatch) | Community-based services | Pharmacists | 18.5 | - | - | - | N | N |
|  | China | Self-reported/questionnaire | General outpatient services | General Medical Practitioners | 4.33 | - | - | 95% CI, 3.79 - 4.87 | N | N |
|  | China | Self-reported/questionnaire | General outpatient services | General Medical Practitioners | 6.28 | - | - | 95% CI, 5.58 - 6.97 | N | N |
| Wazaify (331) | Jordan | Recording (video, audio, stopwatch) | Community-based services | Pharmacists | NA | 2 | - | - | N | N |
| Wei (332) | China | Recording (video, audio, stopwatch) | General outpatient services | General Medical Practitioners | 3.8 | - | Length of time spent in physical examination was 0.8±0.4 minutes | SD, 3.4 | N | N |
| Weise (333) | Australia | Existing databases or guidelines | General outpatient services | General Medical Practitioners | 17.5 | - |  | 95% CI, 16 - 19.1 | N | N |
|  | Australia | Existing databases or guidelines | General outpatient services | General Medical Practitioners | 15.1 | - | 15.0–15.2 | 95% CI, 15 - 15.2 | N | N |
| Weiss (334) | USA | Recording (video, audio, stopwatch) | Second referral level (and above) | Specialist Medical Practitioners | 21 | 19 | - | - | N | N |
| Wendie (335) | Ethiopia | Recording (video, audio, stopwatch) | First referral level | Pharmacists | 4.7 | - | - | - | N | N |
|  | Ethiopia | Recording (video, audio, stopwatch) | First referral level | Pharmacists | 5.5 | - | - | - | N | N |
|  | Ethiopia | Recording (video, audio, stopwatch) | First referral level | Pharmacists | 4.2 | - | - | - | N | N |
|  | Ethiopia | Recording (video, audio, stopwatch) | First referral level | Pharmacists | 4.3 | - | - | - | N | N |
| Weyer (336) | USA | Recording (video, audio, stopwatch) | General outpatient services | Nursing Professionals | 18 | - | - | SD, 9.8 | N | N |
| Willems (337) | Australia | Existing databases or guidelines | General outpatient services | General Medical Practitioners | 20 | - | - | SD, 9 | N | N |
|  | Australia | Existing databases or guidelines | General outpatient services | General Medical Practitioners | 17 | - | - | SD, 8 | N | N |
| Willging (338) | USA | Recording (video, audio, stopwatch) | First referral level | Specialist Medical Practitioners | 19.47 | - | 19:28+8:55, range 7:59–32:01 | SD, 8.92 | N | Y |
| Williams (339) | UK | Recording (video, audio, stopwatch) | First referral level | Nursing Professionals | 95 | - | - | - | N | N |
|  | UK | Recording (video, audio, stopwatch) | First referral level | Specialist Medical Practitioners | 33 | - | - | - | N | N |
|  | UK | Recording (video, audio, stopwatch) | First referral level | Specialist Medical Practitioners | 51 | - | - | - | N | N |
| Winkelman (340) | USA | Software tracking | Second referral level (and above) | Nursing Professionals | NA | 22 | - | - | Y | Y |
| Wogayehu (341) | Ethiopia | Recording (video, audio, stopwatch) | First referral level | Pharmacists | 6.5 | - | - | - | N | N |
|  | Ethiopia | Recording (video, audio, stopwatch) | First referral level | Pharmacists | 6.6 | - | - | - | N | N |
| Wong (342) | Canada | Recording (video, audio, stopwatch) | General outpatient services | Health Professionals not elsewhere classified | NA | 10 | - | - | N | N |
|  | Canada | Recording (video, audio, stopwatch) | General outpatient services | Health Professionals not elsewhere classified | NA | 15 | - | - | N | N |
| Wood (343) | USA | Self-reported/questionnaire | Second referral level (and above) |  | 56.9 | - | - | SD, 29.24 | N | N |
| Xie (344) | China | Recording (video, audio, stopwatch) | First referral level | Specialist Medical Practitioners | 17.8 | 17.8 | - | SD, 13.5 | N | N |
| Yilma (345) | Ethiopia | Self-reported/questionnaire | First referral level | Pharmacists | 14.49 | - | - | - | N | N |
| Young (346) | USA | Recording (video, audio, stopwatch) | General outpatient services | General Medical Practitioners | 18.5 | - | - | SD, 10.5 | N | N |
| Yuan (347) | UK | Existing databases or guidelines | General outpatient services | Dentists | NA | NA | Ranged from 2 min 10 s to 29 min 18 s | - | N | N |
| Zakare-Fagbamila (348) | USA | Existing databases or guidelines | First referral level | Specialist Medical Practitioners | 64 | - | - | SD, 18.92 | N | N |
| Zhang (349) | USA | Software tracking | Second referral level (and above) | Specialist Medical Practitioners | 48.8 | 50 | - | SD, 25.8 | Y | Y |
|  | USA | Software tracking | Second referral level (and above) | Specialist Medical Practitioners | 34.9 | 31.9 | - | SD, 20.3 | Y | Y |
|  | USA | Software tracking | Second referral level (and above) | Specialist Medical Practitioners | 44.5 | 21.6 | - | SD, 44.8 | Y | Y |
|  | USA | Software tracking | Second referral level (and above) | General Medical Practitioners | 16.6 | 13.4 | - | SD, 17.9 | Y | Y |
|  | China | Recording (video, audio, stopwatch) | General outpatient services | General Medical Practitioners | 5.4 | - | - | SD, 3.52 | N | N |
| Zimenkovsky (350) | Ukraine | Recording (video, audio, stopwatch) | Community-based services | Pharmacists | 0.978 | - | 58.7 seconds With queue | - | N | N |
|  | Ukraine | Recording (video, audio, stopwatch) | Community-based services | Pharmacists | 0.822 | - | 49.3 seconds Without queue | - | N | N |
| Zivanovic (351) | Serbia | Electronic health records | Prehospital emergency services | General Medical Practitioners | 21.07 | - | - | 95% CI, 7.63 - 34.51 | N | N |
|  | Serbia | Electronic health records | Prehospital emergency services | General Medical Practitioners | 30.88 | - | - | - | N | N |
|  | Serbia | Electronic health records | Prehospital emergency services | General Medical Practitioners | 20.63 | - | - | - | N | N |
|  | Serbia | Electronic health records | Prehospital emergency services | General Medical Practitioners | 27.86 | - | - | - | N | N |
|  | Serbia | Electronic health records | Prehospital emergency services | General Medical Practitioners | 21.56 | - | - | - | N | N |
|  | Serbia | Electronic health records | Prehospital emergency services | General Medical Practitioners | 20.62 | - | - | - | N | N |
|  | Serbia | Electronic health records | Prehospital emergency services | General Medical Practitioners | 17.19 | - | - | - | N | N |
|  | Serbia | Electronic health records | Prehospital emergency services | General Medical Practitioners | 18.87 | - | - | - | N | N |
|  | Serbia | Electronic health records | Prehospital emergency services | General Medical Practitioners | 20.93 | - | - | - | N | N |
|  | Serbia | Electronic health records | Prehospital emergency services | General Medical Practitioners | 14.04 | - | - | - | N | N |
|  | Serbia | Electronic health records | Prehospital emergency services | General Medical Practitioners | 17.5 | - | - | - | N | N |
|  | Serbia | Electronic health records | Prehospital emergency services | General Medical Practitioners | 20.16 | - | - | - | N | N |
| Yuan (352) | UK | Recording (video, audio, stopwatch) | General outpatient services | Dentists | 19 | - | - | - | N | N |

Updated search results

| **Author** | **Country** | **Method of measurement** | **Service delivery platform** | **UHCC Health worker** | **Mean visit time (min)** | **Median visit time (min)** | **Other (qualitative)** | **Uncertainty (min)** | **Tele-medicine** | **COVID-19** |
| --- | --- | --- | --- | --- | --- | --- | --- | --- | --- | --- |
| Ahmed (353) | Mauritania | Self-reported/questionnaire | General outpatient services | Pharmacists | 21.94 | - | - | 19.56 – 24.31 (CI) | N | N |
| Ahmed(353) | Mauritania | Self-reported/questionnaire | General outpatient services | Pharmacists | 11.86 | - | - | 10.81 – 12.86 (CI) | N | N |
| Ahmed(353) | Mauritania | Self-reported/questionnaire | General outpatient services | Pharmacists | 18.17 | - | - | 16.11 – 20.3 (CI) | N | N |
| Ahmed (353) | Mauritania | Self-reported/questionnaire | General outpatient services | Pharmacists | 11.18 | - | - | 10.01 – 12.49 (CI) | N | N |
| Ahmed (353) | Mauritania | Self-reported/questionnaire | General outpatient services | Pharmacists | 18.61 | - | - | 15.54 – 21.97 (CI) | N | N |
| Ahmed (353) | Mauritania | Self-reported/questionnaire | General outpatient services | Pharmacists | 16.31 | - | - | 15.37 – 17.25 (CI) | N | N |
| Ah-Kye (8) | UK | Software tracking | Second referral level (and above) | Specialist Medical Practitioners | 8 | - | - | - | Y | Y |
| Anugraha (354) | UK | Unclear | First referral level | Specialist Medical Practitioners | 22 | - | Without preconsultation questionnaire, orthopaedic clinic | - | N | N |
| Anugraha (354) | UK | Unclear | First referral level | Specialist Medical Practitioners | 15 | - | With preconsultation questionnaire, orthopaedic clinic | - | N | N |
| Al Halabi (355) | Qatar | Recording (video, audio, stopwatch) | First referral level | Specialist Medical Practitioners | 12 | - | Ward round in hospital – assumed physician time but nurses and pharmacists also present | 7.1 (SD) | N | N |
| Aron (356) | Malawi | Recording (video, audio, stopwatch) | Community-based services | Community Health Workers | - | 34 | Monthly household visit | - | N | Y |
| Aron (356) | Malawi | Recording (video, audio, stopwatch) | Community-based services | Community Health Workers | - | 28 | Daily HIV patient visit | - | N | Y |
| Aron (356) | Malawi | Recording (video, audio, stopwatch) | Community-based services | Community Health Workers | - | 9 | Daily TB patient visit | - | N | Y |
| Aron (356) | Malawi | Recording (video, audio, stopwatch) | Community-based services | Community Health Workers | - | 43 | Postnatal care visit | - | N | Y |
| Aron (356) | Malawi | Recording (video, audio, stopwatch) | Community-based services | Community Health Workers | - | 7 | Referral follow-up visit | - | N | Y |
| Babalola (357) | South Africa | Recording (video, audio, stopwatch) | Community-based services | Community Health Workers | 13.54 | - | - | 9.8 (SD) | N | Y |
| Babayoff (358) | Israel | Existing databases or guidelines | First referral level | Specialist Medical Practitioners | 19.74 | 16.6 | - | 12.25(SD) | N | N |
| Baghus (359) | Netherlands | Self-reported/questionnaire | General outpatient services | General Medical Practitioners | 13.8 | - | - | 5.4 (SD) | N | N |
| Bos-van den Hoek (360) | Netherlands | Software tracking | Second referral level (and above) | Specialist Medical Practitioners | 30.72 | - | Palliative oncology | 6.68 (SD) | Y | Y |
| Bos-van den Hoek (360) | Netherlands | Software tracking | Second referral level (and above) | Specialist Medical Practitioners | 29.98 | - | Palliative oncology | 4.98 (SD) | Y | Y |
| Bryl (361) | US | Electronic health records | General outpatient services | Social work and counselling professionals | - | - | <10 mins (13%); 15-25 mins (41.6%); 30-45 mins (14.5%); 60 mins (3.4%); 75 mins (0.3%); > 90 mins (0.1%) | - | N | N |
| Bryl (361) | US | Electronic health records | Second referral level (and above) | Social work and counselling professionals | - | - | <10 mins (5.1%); 15-25 mins (11.3%); 30-45 mins (8%); 60 mins (11.1%); 75 mins (1.6%); > 90 mins (0.9%) | - | N | N |
| Chukwusa (362) | UK | Electronic health records | General outpatient services | General Medical Practitioners | 10.1 | - | - | - | N | N |
| Chukwusa (362) | UK | Electronic health records | General outpatient services | General Medical Practitioners | 11.5 | - | - | - | N | Y |
| Consolandi (363) | Italy | Recording (video, audio, stopwatch) | Second referral level (and above) | Specialist Medical Practitioners | 31.1 |  | All | 26.9, 35.4 (CI) | N | Y |
| Consolandi (363) | Italy | Recording (video, audio, stopwatch) | Second referral level (and above) | Specialist Medical Practitioners | 37.8 |  | Oncologists |  |  |  |
| Consolandi (363) | Italy | Recording (video, audio, stopwatch) | Second referral level (and above) | Specialist Medical Practitioners | 26.2 |  | Gastroenterology physician |  |  |  |
| Consolandi (363) | Italy | Recording (video, audio, stopwatch) | Second referral level (and above) | Specialist Medical Practitioners | 16 |  | Surgeons |  |  |  |
| Couch (364) | Australia | Self-reported/questionnaire | General outpatient services | Health Professionals not elsewhere classified | - | - | <10 min (1%), 11-15 min (2%), 12-60 min (18%), 21-30 min (57%), >31 min (22%) | - | N | N |
| Drennan (365) | US | Self-reported/questionnaire | Second referral level (and above) | Pharmacists | - | 17 | Child oncology | - | N | Y |
| Ebbers (366) | Netherlands | Recording (video, audio, stopwatch) | Second referral level (and above) | Specialist Medical Practitioners | 33.7 | - | - | 28.6, 39.1 (CI) | N | N |
| Ebbers (366) | Netherlands | Recording (video, audio, stopwatch) | Second referral level (and above) | Specialist Medical Practitioners | 30.4 | - | - | 24.4, 36.4 (CI) | N | N |
| Ebbers (366) | Netherlands | Recording (video, audio, stopwatch) | Second referral level (and above) | Specialist Medical Practitioners | 13.52 | - | - | 11.81, 15.23 (CI) | N | N |
| Ebbers (366) | Netherlands | Recording (video, audio, stopwatch) | Second referral level (and above) | Specialist Medical Practitioners | 11.09 | - | - | 9.33, 12.85 (CI) | N | N |
| El-Dahiyat (367) | United Arab Emirates | Electronic health records | Second referral level (and above) | - | 17 | - | - | - | N | N |
| Fisher (368) | Australia | Existing databases or guidelines | General outpatient services | General Medical Practitioners | 14.9 | - | Video | 7.7 (SD) | Y | Y |
|  | Australia | Existing databases or guidelines | General outpatient services | General Medical Practitioners | 12.8 | - | Telephone | 7.4 (SD) | Y | Y |
| Fisher (369) | Australia | Existing databases or guidelines | General outpatient services | General Medical Practitioners | 18.7 | - | - | 10.26 (SD) | N | Y |
| Fisher (369) | Australia | Existing databases or guidelines | General outpatient services | General Medical Practitioners | 12.91 | - | - | 7.42 (SD) | Y | Y |
| Fritz (370) | Switzerland | Self-reported/questionnaire | Community-based services | General Medical Practitioners | 23.9 | - | - | 12.9 (SD) | N | N |
| Goruntla (371) | Uganda | Self-reported/questionnaire | First referral level | Pharmacists | 5.41 | - | - | - | N | N |
| Harris (372) | US | Recording (video, audio, stopwatch) | First referral level | Specialist Medical Practitioners | - | 37 | - | - | N | N |
| Harrison (373) | Australia | Existing databases or guidelines | General outpatient services | General Medical Practitioners | - | - | 1-19 mins (74.5%); 20-39 mins (23.3%); >40 mins (2.2%) | - | N | N |
| Liljenquist (374) | USA | Recording (video, audio, stopwatch) | General outpatient services | General Medical Practitioners | - | 14 | Well child visit | - | N | N |
| Joshi (375) | India | Recording (video, audio, stopwatch) | Second referral level (and above) | Specialist Medical Practitioners | 9.88 | - | Both type of visit | 0.0007 (SD) | N | N |
| Joshi (375) | India | Recording (video, audio, stopwatch) | Second referral level (and above) | Specialist Medical Practitioners | 9.87 | - | Follow-up | 0.0007 (SD) | N | N |
| Joshi (375) | India | Recording (video, audio, stopwatch) | Second referral level (and above) | Specialist Medical Practitioners | 9.97 | - | Initial | 0.0005 (SD) | N | N |
| Katzan (376) | USA | Electronic health records | First referral level | Specialist Medical Practitioners | 36.3 | - | - | 23.8 (SD) | N | Y |
| Khan (377) | Pakistan | Recording (video, audio, stopwatch) | Second referral level (and above) | Specialist Medical Practitioners | 4 | - | Specialist Registrar Consultation – all rheumatoid arthritis | 6.2 | N | N |
| Khan (377) | Pakistan | Recording (video, audio, stopwatch) | Second referral level (and above) | Specialist Medical Practitioners | 8 | - | Rheumatologist Consultation - all rheumatoid arthritis | 5.7 | N | N |
| Khan (377) | Pakistan | Recording (video, audio, stopwatch) | Second referral level (and above) | Specialist Medical Practitioners | 4.5 | - | Rheumatologist Consultation – initial rheumatoid arthritis | 6.4 | N | N |
| Khan (377) | Pakistan | Recording (video, audio, stopwatch) | Second referral level (and above) | Specialist Medical Practitioners | 5 | - | Specialist Registrar Consultation – Follow up rheumatoid arthritis | 6.4 | N | N |
| Khan (377) | Pakistan | Recording (video, audio, stopwatch) | Second referral level (and above) | Specialist Medical Practitioners | 8 | - | Rheumatologist Consultation – Follow up rheumatoid arthritis | 5.7 | N | N |
| Khan (377) | Pakistan | Recording (video, audio, stopwatch) | Second referral level (and above) | Specialist Medical Practitioners | 3 | - | Specialist Registrar Consultation – all lupus | 3.8 | N | N |
| Khan (377) | Pakistan | Recording (video, audio, stopwatch) | Second referral level (and above) | Specialist Medical Practitioners | 4 | - | Rheumatologist Consultation – all lupus | 3 | N | N |
| Khan (377) | Pakistan | Recording (video, audio, stopwatch) | Second referral level (and above) | Specialist Medical Practitioners | 2.5 | - | Specialist Registrar Consultation– initial lupus | 2.1 | N | N |
| Khan (377) | Pakistan | Recording (video, audio, stopwatch) | Second referral level (and above) | Specialist Medical Practitioners | 5.4 | - | Rheumatologist Consultation – initial lupus | 2.8 | N | N |
| Khan (377) | Pakistan | Recording (video, audio, stopwatch) | Second referral level (and above) | Specialist Medical Practitioners | 3 | - | Specialist Registrar Consultation – follow-up lupus | 4.1 | N | N |
| Khan (377) | Pakistan | Recording (video, audio, stopwatch) | Second referral level (and above) | Specialist Medical Practitioners | 4 | - | Rheumatologist Consultation – follow-up lupus | 3.1 | N | N |
| Khan 2022 (378) | Pakistan | Self-reported/questionnaire | Second referral level (and above) | Specialist Medical Practitioners | - | - | 30 min (50.7%), 30-60 (30.6%), more than 60 min (18.7%) | - | N | Y |
| Kim (379) | Republic of Korea | Unclear | General outpatient services | General Medical Practitioners | 6.3 | - | - | - | N | N |
| Kim (379) | Taiwan (Province of China) | Unclear | General outpatient services | General Medical Practitioners | 5 | - | - | - | N | N |
| Klarman (380) | Haiti | Software tracking | Community-based services | Nursing Professionals | 20 | - | - | - | Y | N |
| Koch (381) | Sweden | Self-reported/questionnaire | General outpatient services | General Medical Practitioners | 9.3 | - | - | - | Y | N |
| Koch (381) | Sweden | Self-reported/questionnaire | General outpatient services | General Medical Practitioners | 20.8 | - | - | - | N | N |
| Mailankody (382) | India | Self-reported/questionnaire | Second referral level (and above) | Specialist Medical Practitioners | 6.1 | - | Oncology | - | N | Y |
| Parvu (383) | Romania | Existing databases or guidelines | General outpatient services | Specialist Medical Practitioners | 16.2 | - | Occupational medicine | 10 (SD) | N | N |
| Marshall (384) | US | Existing databases or guidelines | First referral level | Specialist Medical Practitioners | 22.92 | - | - | - | N | N |
| Marshall (384) | US | Existing databases or guidelines | First referral level | Specialist Medical Practitioners | 21.18 | - | - | - | N | N |
| Marshall (384) | US | Existing databases or guidelines | First referral level | Specialist Medical Practitioners | 21.77 | - | - | - | N | N |
| Marshall (384) | US | Existing databases or guidelines | First referral level | Specialist Medical Practitioners | 21.28 | - | - | - | N | N |
| Marshall (384) | US | Existing databases or guidelines | First referral level | Specialist Medical Practitioners | 22.38 | - | - | - | N | N |
| Marshall (384) | US | Existing databases or guidelines | First referral level | Specialist Medical Practitioners | 22.64 | - | - | - | N | N |
| Marshall (384) | US | Existing databases or guidelines | First referral level | Specialist Medical Practitioners | 23.78 | - | - | - | N | N |
| Marshall (384) | US | Existing databases or guidelines | First referral level | Specialist Medical Practitioners | 24.59 | - | - | - | N | N |
| Michel (385) | US | Electronic health records | Second referral level (and above) | Specialist Medical Practitioners | - | 29 | - | - | N | Y |
| Michel (385) | US | Electronic health records | Second referral level (and above) | Specialist Medical Practitioners | - | 28 | - | - | Y | Y |
| Mitaly (386) | US | Software tracking | Second referral level (and above) | Pharmacists | 26 | 20 | Semi-urgent  consults | - | Y | N |
| Mitaly (386) | US | Software tracking | Second referral level (and above) | Pharmacists | 14 | 20 | Clinically actionable consults | - | Y | N |
| Mukora (387) | South Africa | Recording (video, audio, stopwatch) | Community-based services | Community Health Workers | - | 6.7 | Peri-urban | - | N | N |
| Mukora (387) | South Africa | Recording (video, audio, stopwatch) | Community-based services | Community Health Workers | - | 12.1 | Urban | - | N | N |
| Mwamba (388) | Zambia | Recording (video, audio, stopwatch) | General outpatient services | Health Professionals not elsewhere classified | - | 7.48 | Majority clinical officers but also includes medical officers and nurses. | - | N | Y |
| Hill (389) | Germany | Recording (video, audio, stopwatch) | General outpatient services | General Medical Practitioners | 11 | - | Primary care walk-in clinics for refugees | 7 (SD) | N | N |
| Ospina (390) | US | Recording (video, audio, stopwatch) | Second referral level (and above) | Specialist Medical Practitioners | - | 17 | Thyroid nodules | - | N | N |
| Ozkardes (391) | US | Existing databases or guidelines | First referral level | Specialist Medical Practitioners | 21.4 | - | All | 20.9, 22 (CI) | N | N |
| Ozkardes (391) | US | Existing databases or guidelines | First referral level | Specialist Medical Practitioners | 22 | - | Private | 21.5, 22.6 (CI) | N | N |
| Ozkardes (391) | US | Existing databases or guidelines | First referral level | Specialist Medical Practitioners | 19.4 | - | Medicaid | 18.4, 20.3 (CI) | N | N |
| Ozkardes (391) | US | Existing databases or guidelines | First referral level | Specialist Medical Practitioners | 20.1 | - | Uninsured | 18.4, 21.7 (CI) | N | N |
| Papadopoulos (392) | Multiple (88 countries) | Self-reported/questionnaire | Second referral level (and above) | Specialist Medical Practitioners | - | 25 | - | - | N | N |
| Papadopoulos (392) | Multiple (88 countries) | Self-reported/questionnaire | Second referral level (and above) | Specialist Medical Practitioners | - | 25 | - | - | N | N |
| Prather (393) | US | - | First referral level | Specialist Medical Practitioners | 44.3 | - | Initial Chiari malformation consultation | 13.7 (SD) | N | N |
| Prather (393) | US | - | First referral level | Specialist Medical Practitioners | 29.8 | - | Follow-up Chiari malformation consultation | 9.2 (SD) | N | N |
| Rahaman (394) | Trinidad | Recording (video, audio, stopwatch) | General outpatient services | General Medical Practitioners | 8.4 | - | - | 4.429 (SD) | N | N |
| Schroder (395) | Germany | Software tracking | Emergency care | Specialist Medical Practitioners | 12.07 | - | - | - | Y | N |
| Schroder (395) | Germany | Software tracking | Emergency care | Specialist Medical Practitioners | 9.42 | - | - | - | Y | Y |
| Scott (396) | Australia | Existing databases or guidelines | General outpatient services | General Medical Practitioners | 17.17 | - | - | 6.29 (SD) | N | N |
| Sharma (397) | India | Self-reported/questionnaire | General outpatient services – free clinic | General Medical Practitioners | - | - | >5 min (41.0%), 5-10 min (59.0%), 10-20 min (0.0%), >20 min (0.0%) | - | N | N |
| Sharma (397) | India | Self-reported/questionnaire | General outpatient services – private clinic | General Medical Practitioners | - | - | >5 min (0.0%), 5-10 min (19.0%), 10-20 min (58.5%), >20 min (22.5%) | - | N | N |
| Shaw (398) | UK | Recording (video, audio, stopwatch) | Second referral level (and above) | Specialist Medical Practitioners | 19.6 | - | Diabetes | - | Y | N |
| Shaw (398) | UK | Recording (video, audio, stopwatch) | Second referral level (and above) | Specialist Medical Practitioners | 20.17 | - | Diabetes | - | N | N |
| Shaw (398) | UK | Recording (video, audio, stopwatch) | Second referral level (and above) | Specialist Medical Practitioners | 7.63 | - | Antenatal | - | Y | N |
| Shaw (398) | UK | Recording (video, audio, stopwatch) | Second referral level (and above) | Specialist Medical Practitioners | 8.22 | - | Antenetal | - | N | N |
| Shaw (398) | UK | Recording (video, audio, stopwatch) | Second referral level (and above) | Specialist Medical Practitioners | 11.83 | - | Cancer surgery | - | Y | N |
| Shaw (398) | UK | Recording (video, audio, stopwatch) | Second referral level (and above) | Specialist Medical Practitioners | 19.67 | - | Cancer surgery | - | N | N |
| Stewart (300) | US | Self-reported/questionnaire | Second referral level (and above) | Specialist Medical Practitioners | 54 | - | Pediatric Ophthalmology | - | Y | N |
| Stewart (300) | US | Self-reported/questionnaire | Second referral level (and above) | Optometrists and Ophthalmic Opticians | 26 | - | Pediatric Ophthalmology | - | Y | N |
| Stewart (300) | US | Self-reported/questionnaire | Second referral level (and above) | Specialist Medical Practitioners | 33 | - | Pediatric Ophthalmology | - | Y | N |
| Stewart (300) | US | Self-reported/questionnaire | Second referral level (and above) | Optometrists and Ophthalmic Opticians | 10 | - | Pediatric Ophthalmology | - | Y | N |
| Sugiura (399) | Japan | Electronic health records | Community-based services | Pharmacists | 23.5 | - | - | 10.9 (SD) | N | N |
| Sugiura (399) | Japan | Electronic health records | Community-based services | Pharmacists | 36.7 | - | - | 5.2 (SD) | N | N |
| Tan (400) | Singapore | Self-reported/questionnaire | General outpatient services | General Medical Practitioners | - | 6.32 | Telemedicine | - | Y | N |
| Tan (400) | Singapore | Self-reported/questionnaire | General outpatient services | General Medical Practitioners | - | 8.57 | In-person | - | N | N |
| Tanner (401) | US | Electronic health records | First referral level | Health Professionals not elsewhere classified (Occupational therapists – autism spectrum disorder) | - | - | 15 min (0%), 30 min (0%), 45 min (5.5%), 60 min (94.5%), 90 min (0%), 120 min (0%), 180 min (0%) | - | Y | N |
| Tanner (401) | US | Electronic health records | First referral level | Health Professionals not elsewhere classified (Occupational therapists – cerebral palsy) | - | - | 15 min (0%), 30 min (0%), 45 min (10.2%), 60 min (88.5%), 90 min (0%), 120 min (0%), 180 min (1.3%) | - | Y | N |
| Tanner (401) | US | Electronic health records | First referral level | Health Professionals not elsewhere classified (Occupational therapists – feeding disorder) | - | - | 15 min (0%), 30 min (0.8%), 45 min (7.5%), 60 min (91.7%), 90 min (0%), 120 min (0%), 180 min (0%) | - | Y | N |
| Tanner (401) | US | Electronic health records | First referral level | Health Professionals not elsewhere classified (Occupational therapists – autism spectrum disorder) | - | - | 15 min (0.2%), 30 min (5.5%), 45 min (5.6%), 60 min (88.6%), 90 min (0%), 120 min (0%), 180 min (0%) | - | N | Y |
| Tanner (401) | US | Electronic health records | First referral level | Health Professionals not elsewhere classified (Occupational therapists – cerebral palsy) | - | - | 15 min (1.2%), 30 min (1.5%), 45 min (6.4%), 60 min (83.7%), 90 min (0.1%), 120 min (5.1%), 180 min (2.0%) | - | N | Y |
| Tanner (401) | US | Electronic health records | First referral level | Health Professionals not elsewhere classified (Occupational therapists – feeding disorder) | - | - | 15 min (0%), 30 min (0%), 45 min (5.5%), 60 min (94.5%), 90 min (0%), 120 min (0%), 180 min (0%) | - | N | Y |
| Vucak (402) | Croatia | Recording (video, audio, stopwatch) | General outpatient services | General Medical Practitioners | 7.13 | - | - | 3.38 (SD) | N | Y |
| Vucak (402) | Croatia | Recording (video, audio, stopwatch) | General outpatient services | General Medical Practitioners | 4.01 | - | - | 2.09 (SD) | Y | Y |
| Walter (403) | Canada | Software tracking | Other | Specialist Medical Practitioners | 16.4 | - | - | - | Y | Y |
| Ward (404) | UK | Recording (video, audio, stopwatch) | General outpatient services | General Medical Practitioners | 13.28 | - | Overall | 5.45 (SD) | N | N |
| Ward (404) | UK | Recording (video, audio, stopwatch) | General outpatient services | General Medical Practitioners | 13.07 | - | Diabetes | - | N | N |
| Ward (404) | UK | Recording (video, audio, stopwatch) | General outpatient services | General Medical Practitioners | 12.02 | - | Cancer | - | N | N |
| Ward (404) | UK | Recording (video, audio, stopwatch) | General outpatient services | General Medical Practitioners | 13.55 | - | Chronic pain | - | N | N |
| Ward (404) | UK | Recording (video, audio, stopwatch) | General outpatient services | General Medical Practitioners | 9.5 | - | Cardiovascular | - | N | N |
| Ward (404) | UK | Recording (video, audio, stopwatch) | General outpatient services | General Medical Practitioners | 16.22 | - | Respiratory | - | N | N |
| Willging (338) | USA | Recording (video, audio, stopwatch) | First referral level | Specialist Medical Practitioners | 19.46 | - | Vascular surgeons | 8.92 (SD) | N | Y |
| Wisniewski (405) | USA | Existing databases or guidelines | General outpatient services | General Medical Practitioners | 38.6 | - | - | 15.3 (SD) | N | Y |
| Wisniewski (405) | USA | Existing databases or guidelines | General outpatient services | General Medical Practitioners | 39.5 | - | - | 13 (SD) | N | Y |
| Yoon (406) | USA | Self-reported/questionnaire | Community-based services | Pharmaceutical Technicians and Assistants | 15.5 | - | Pharmacy technicians - phone | 11.5 (SD) | Y | Y |
| Yoon (406) | USA | Self-reported/questionnaire | Community-based services | Pharmaceutical Technicians and Assistants | 43.1 | - | Pharmacy technicians - home | 14.4 (SD) | N | Y |
| Yoon (406) | USA | Self-reported/questionnaire | Community-based services | Pharmaceutical Technicians and Assistants | 30.6 | - | Pharmacy technicians - pharmacy | 16.8 (SD) | N | Y |
| Zhan (407) | China | Recording (video, audio, stopwatch) | Second referral level (and above) | Specialist Medical Practitioners | 4.85 | - | All | 3.09 (SD) | N | N |
| Zhan (407) | China | Recording (video, audio, stopwatch) | Second referral level (and above) | Specialist Medical Practitioners | 3.81 | - | Follow-up | 2.24 (SD) | N | N |
| Zhan (407) | China | Recording (video, audio, stopwatch) | Second referral level (and above) | Specialist Medical Practitioners | 3 | - | Administering the formalities requisite for patient admission | 1.92 (SD) | N | N |
| Zhan (407) | China | Recording (video, audio, stopwatch) | Second referral level (and above) | Specialist Medical Practitioners | 3.7 | - | Prescribing anti-tumor drugs | 2.3 (SD) | N | N |
| Zhan (407) | China | Recording (video, audio, stopwatch) | Second referral level (and above) | Specialist Medical Practitioners | 5.91 | - | Symptom control | 3.44 (SD) | N | N |
| Zhan (407) | China | Recording (video, audio, stopwatch) | Second referral level (and above) | Specialist Medical Practitioners | 7.75 | - | Medical insurance reimbursement | 4.63 (SD) | N | N |
| Zhan (407) | China | Recording (video, audio, stopwatch) | Second referral level (and above) | Specialist Medical Practitioners | 5.78 | - | Others | 3.1 (SD) | N | N |

## A5 AXIS results

| **Author** | **1** | **2** | **3** | **4** | **5** | **6** | **7** | **8** | **9** | **10** | **11** | **12** | **13** | **14** | **15** | **16** | **17** | **18** | **19** | **20** |
| --- | --- | --- | --- | --- | --- | --- | --- | --- | --- | --- | --- | --- | --- | --- | --- | --- | --- | --- | --- | --- |
| S Abbas (269) | Y | Y | N | Y | Y | Y | N | Y | N | N | N | Y | N | N | Y | Y | Y | Y | DK | Y |
| Abdu-Aguye (2) | Y | Y | N | Y | Y | DK | DK | Y | Y | Y | Y | Y | DK | N | Y | Y | Y | Y | N | Y |
| Abdulkader (3) | Y | Y | Y | Y | Y | DK | DK | Y | Y | Y | Y | Y | DK | N | Y | Y | Y | Y | N | Y |
| Abdus-Salam (4)RA | Y | Y | Y | Y | Y | DK | DK | Y | Y | Y | Y | Y | DK | N | Y | Y | Y | Y | N | Y |
| Abner (5) | Y | Y | N | Y | Y | Y | Y | Y | Y | Y | Y | Y | Y | N | Y | Y | Y | Y | N | Y |
| Aboueid (6) | Y | Y | N | Y | DK | DK | DK | Y | Y | Y | Y | Y | DK | N | Y | Y | Y | Y | N | Y |
| Abushaala (7) | Y | Y | N | Y | Y | Y | Y | Y | Y | Y | Y | Y | N | N | Y | Y | Y | N | N | N |
| Ahmad (9) BA | Y | Y | N | Y | Y | Y | Y | Y | Y | Y | Y | Y | Y | N | Y | Y | Y | Y | DK | N |
| Ahmed H | Y | Y | Y | Y | Y | Y | DK | Y | Y | Y | Y | Y | DK | N | Y | Y | Y | Y | DK | DK |
| Alarcon-Ruiz (10) | Y | Y | N | Y | Y | Y | Y | Y | Y | Y | Y | Y | N | N | Y | Y | Y | Y | N | N |
| Al-Habbal (11) | Y | Y | N | Y | Y | Y | N | Y | Y | Y | Y | Y | N | N | Y | Y | Y | Y | N | Y |
| Al-Harajin (12) | Y | Y | Y | Y | Y | DK | N | Y | Y | Y | Y | Y | DK | N | Y | Y | Y | Y | N | Y |
| Aljehani (13) | Y | Y | Y | Y | Y | DK | DK | Y | Y | Y | Y | Y | DK | N | Y | Y | Y | Y | DK | N |
| Allen (14) | Y | Y | N | Y | Y | Y | Y | Y | Y | Y | Y | Y | N | N | Y | Y | Y | Y | N | Y |
| Alsubeeh (15) | Y | Y | Y | Y | Y | Y | Y | Y | Y | Y | Y | Y | N | N | Y | Y | Y | Y | N | Y |
| Alzayer (16) | Y | Y | Y | Y | Y | DK | DK | Y | Y | Y | Y | Y | DK | N | Y | Y | Y | Y | N | Y |
| Andreyeva (17) | Y | Y | N | Y | Y | DK | DK | Y | Y | Y | Y | Y | DK | N | Y | Y | Y | Y | DK | N |
| Ann-Yi (18) | Y | Y | N | Y | Y | Y | Y | Y | Y | Y | Y | Y | N | N | Y | Y | Y | Y | N | N |
| Appiah (19) | Y | Y | N | Y | Y | DK | DK | Y | Y | Y | Y | Y | DK | N | Y | Y | Y | Y | N | N |
| Ariyo (20) | Y | Y | DK | Y | Y | Y | Y | Y | Y | Y | Y | Y | N | N | Y | Y | Y | Y | DK | DK |
| Asamani (21) | Y | Y | Y | Y | Y | DK | DK | Y | Y | Y | Y | Y | DK | N | Y | Y | Y | Y | N | Y |
| Asan (22) | Y | Y | Y | Y | Y | DK | DK | Y | Y | Y | Y | Y | DK | N | Y | Y | Y | Y | N | Y |
| Assefa (23) | Y | Y | N | Y | Y | DK | DK | Y | Y | Y | Y | Y | DK | N | Y | Y | Y | Y | DK | Y |
| Atanda (24) | Y | Y | N | Y | Y | Y | Y | Y | Y | Y | Y | Y | N | N | Y | Y | Y | Y | N | Y |
| Atia (25) | Y | Y | Y | Y | Y | Y | Y | Y | Y | Y | Y | Y | N | N | Y | Y | Y | Y | N | Y |
| Ayalew (26) | Y | Y | Y | Y | Y | DK | DK | Y | Y | Y | Y | Y | DK | N | Y | Y | Y | Y | N | Y |
| Ayalew Getahun (26) | Y | Y | Y | Y | Y | DK | DK | Y | Y | Y | Y | Y | DK | N | Y | Y | Y | Y | N | Y |
| Ayele (27) | Y | Y | Y | Y | Y | Y | Y | Y | Y | Y | Y | Y | N | N | Y | Y | Y | Y | N | Y |
| Barratt (28) | Y | Y | N | Y | Y | DK | DK | Y | Y | Y | Y | Y | DK | N | Y | Y | Y | Y | N | Y |
| Bauer (29) | Y | Y | N | Y | Y | Y | Y | Y | Y | Y | Y | Y | N | N | Y | Y | Y | Y | N | N |
| Beach (30) | Y | Y | N | Y | Y | Y | N | Y | Y | Y | Y | Y | N | N | Y | Y | Y | Y | N | Y |
| Bener (31) | Y | Y | N | Y | Y | DK | DK | Y | Y | Y | Y | Y | DK | N | Y | Y | Y | N | N | Y |
| Benski (32) (i) | Y | Y | N | Y | Y | Y | N | Y | Y | Y | Y | Y | N | N | Y | Y | Y | Y | N | Y |
| Benski (33) (ii) | Y | Y | N | Y | Y | Y | Y | Y | Y | Y | Y | Y | N | N | Y | Y | Y | N | Y | Y |
| Berasa (34) | Y | Y | Y | Y | Y | DK | DK | Y | Y | Y | Y | Y | DK | N | Y | Y | Y | Y | N | Y |
| Berk (35) | Y | Y | N | Y | Y | Y | Y | Y | Y | Y | Y | Y | N | N | Y | Y | Y | N | N | Y |
| Berkowitz (36) | Y | Y | N | Y | Y | DK | DK | Y | Y | Y | Y | Y | DK | N | Y | Y | Y | N | N | Y |
| Binyaruka (37) | Y | Y | N | Y | Y | DK | DK | Y | Y | Y | Y | Y | DK | N | Y | Y | Y | Y | N | Y |
| Birhanu (38) | Y | Y | N | Y | Y | DK | DK | Y | Y | Y | Y | Y | DK | N | Y | Y | Y | Y | N | Y |
| Bissessor (39) | Y | Y | N | Y | Y | Y | Y | Y | Y | Y | Y | Y | N | N | Y | Y | Y | Y | N | Y |
| Black (40) | Y | Y | Y | Y | Y | DK | DK | Y | Y | Y | Y | Y | DK | N | Y | Y | Y | Y | N | Y |
| Bock (41) | Y | Y | N | Y | Y | Y | Y | Y | Y | Y | Y | N | Y | N | Y | Y | Y | N | N | Y |
| Boffa (42) | Y | Y | N | N | N | DK | DK | Y | Y | Y | Y | Y | Y | N | Y | Y | Y | Y | N | Y |
| Bolívar (43) | Y | Y | N | Y | Y | DK | DK | Y | Y | Y | Y | Y | DK | N | Y | Y | Y | Y | N | Y |
| Bon (44) | Y | Y | N | Y | Y | Y | Y | Y | Y | Y | Y | Y | N | N | Y | Y | Y | N | N | Y |
| Bonney (45) | Y | Y | N | Y | Y | Y | Y | Y | Y | Y | Y | Y | N | N | Y | Y | Y | Y | N | Y |
| Bowen (46) | Y | Y | N | Y | Y | Y | Y | Y | Y | Y | Y | Y | N | N | Y | Y | Y | Y | DK | Y |
| Brenner (47) | Y | Y | N | Y | Y | DK | DK | Y | Y | Y | Y | Y | DK | N | Y | Y | Y | Y | N | Y |
| Breyer (48) | Y | Y | N | Y | Y | DK | DK | Y | Y | Y | Y | Y | Y | N | Y | Y | Y | Y | DK | N |
| Burger (49) | Y | Y | N | Y | Y | DK | DK | Y | Y | Y | Y | Y | DK | N | Y | Y | Y | Y | N | Y |
| Burnett (50)-Zeiman | Y | Y | N | Y | Y | Y | Y | Y | Y | Y | Y | Y | Y | N | Y | Y | Y | Y | N | Y |
| Cabrera-Rivadeneyra (51) | Y | Y | Y | Y | Y | DK | DK | Y | Y | Y | Y | Y | DK | N | Y | Y | Y | Y | N | DK |
| Calvitti (52) | Y | Y | N | Y | Y | Y | Y | Y | Y | Y | Y | Y | N | N | Y | Y | Y | Y | N | Y |
| Castner (53) | Y | Y | N | Y | Y | Y | Y | Y | Y | Y | Y | Y | N | N | Y | Y | Y | Y | DK | Y |
| Chao (54) | Y | Y | N | Y | Y | Y | Y | Y | Y | Y | Y | N | N | N | Y | Y | Y | Y | N | N |
| Chebolu-Subramanian (55) | Y | Y | N | Y | Y | DK | DK | Y | Y | Y | Y | Y | N | N | Y | Y | Y | Y | N | Y |
| Cheung (56) | Y | Y | N | Y | DK | DK | Y | Y | Y | Y | Y | Y | DK | N | Y | Y | Y | Y | N | Y |
| Chingombe (57) | Y | Y | N | Y | Y | Y | DK | Y | Y | Y | Y | Y | DK | Y | Y | Y | Y | Y | N | Y |
| Cho (58) | Y | Y | N | Y | Y | Y | Y | Y | Y | Y | Y | Y | N | N | Y | Y | Y | Y | N | DK |
| Ciocănel (59) | Y | Y | N | Y | DK | DK | DK | Y | Y | Y | Y | N | DK | N | Y | Y | Y | N | N | Y |
| Compère (60) | Y | Y | Y | Y | Y | Y | Y | Y | Y | Y | Y | Y | N | N | Y | Y | Y | Y | N | Y |
| Conley (61) | Y | Y | N | Y | Y | Y | Y | Y | Y | Y | Y | Y | N | N | Y | Y | Y | Y | N | N |
| Corn (62) | Y | Y | N | Y | Y | DK | DK | Y | Y | Y | Y | Y | DK | N | Y | Y | Y | Y | N | Y |
| Crocker-Buque (63) (1) | Y | Y | N | Y | Y | DK | DK | Y | Y | Y | Y | Y | DK | N | Y | Y | Y | Y | N | N |
| Crosbie (64) | Y | Y | N | Y | Y | DK | DK | Y | Y | Y | Y | Y | DK | N | Y | Y | Y | Y | N | Y |
| Cui (65) | Y | Y | N | Y | Y | Y | Y | Y | Y | Y | Y | Y | N | N | Y | Y | Y | Y | N | N |
| Dabaghzadeh (66) | Y | Y | N | Y | Y | Y | DK | Y | Y | Y | Y | Y | N | N | Y | Y | Y | Y | N | Y |
| Dakroub (67) | Y | Y | N | Y | Y | Y | Y | Y | Y | Y | Y | Y | N | N | Y | Y | Y | Y | DK | N |
| Daniel (68) | Y | Y | Y | Y | Y | DK | DK | Y | Y | Y | Y | Y | DK | N | Y | Y | Y | Y | Y | Y |
| Darlison (69) | Y | Y | N | Y | Y | Y | Y | Y | Y | Y | Y | Y | N | N | Y | Y | Y | Y | N | Y |
| Das (70) | Y | Y | Y | Y | Y | Y | DK | Y | Y | Y | Y | Y | N | N | Y | Y | Y | Y | N | Y |
| Dessie (71) | Y | Y | N | Y | Y | DK | DK | Y | Y | Y | Y | Y | DK | N | Y | Y | Y | Y | N | Y |
| DeWyer (72) | Y | Y | N | Y | Y | DK | DK | Y | Y | Y | Y | Y | DK | N | Y | Y | Y | Y | N | Y |
| Dingwall (73) | Y | Y | N | Y | Y | DK | DK | Y | Y | Y | Y | Y | DK | DK | Y | Y | Y | Y | N | N |
| Donahue (74) | Y | Y | N | Y | Y | DK | DK | Y | Y | Y | Y | Y | DK | N | Y | Y | Y | Y | DK | Y |
| Dubois (76) | Y | Y | N | Y | Y | DK | DK | Y | Y | Y | Y | Y | Y | N | Y | Y | Y | Y | N | Y |
| Ebbers (77) | Y | Y | Y | Y | Y | Y | Y | Y | Y | Y | Y | Y | N | N | Y | Y | Y | Y | N | Y |
| Ekberg (78) | Y | Y | N | Y | DK | DK | DK | Y | Y | Y | Y | Y | DK | N | Y | Y | Y | N | N | N |
| Driever (75) | Y | Y | N | Y | Y | DK | DK | Y | Y | Y | Y | Y | DK | N | Y | Y | Y | Y | N | Y |
| Ellington (79) | Y | Y | N | Y | Y | DK | DK | Y | Y | Y | Y | Y | DK | N | Y | Y | Y | Y | N | Y |
| Elliot (80) | Y | Y | N | Y | Y | Y | Y | Y | Y | Y | Y | Y | Y | N | Y | Y | Y | Y | N | Y |
| Ellis (81) | Y | Y | N | Y | Y | DK | DK | Y | Y | Y | Y | Y | DK | N | Y | Y | Y | Y | N | Y |
| Elrggal (82) | Y | Y | N | Y | Y | DK | DK | Y | Y | Y | Y | Y | DK | N | Y | Y | Y | Y | N | Y |
| Ewelukwa (83) | Y | Y | N | Y | Y | Y | Y | Y | Y | Y | Y | Y | N | N | Y | Y | Y | Y | N | N |
| Fang (84), Y. | Y | Y | N | Y | Y | DK | DK | Y | Y | Y | Y | Y | DK | N | Y | Y | Y | Y | DK | Y |
| Fatigante (85) | Y | Y | N | Y | Y | DK | DK | Y | Y | Y | Y | Y | DK | N | Y | Y | Y | N | N | Y |
| Fesler (86) | Y | Y | N | Y | Y | Y | Y | Y | Y | Y | Y | Y | N | N | Y | Y | Y | Y | DK | N |
| Fieux (87) | Y | Y | N | Y | Y | DK | DK | Y | Y | Y | Y | Y | DK | N | Y | Y | Y | Y | N | Y |
| Fisher (88) | Y | Y | N | Y | Y | Y | Y | Y | Y | Y | Y | Y | N | Y | Y | Y | Y | Y | N | Y |
| Folayan (89) | Y | Y | N | Y | Y | Y | Y | Y | Y | Y | Y | Y | N | N | Y | Y | Y | Y | DK | Y |
| Ensign (90) | Y | Y | N | Y | Y | Y | Y | Y | Y | Y | Y | Y | N | N | Y | Y | Y | N | N | N |
| Frye (92) | Y | Y | N | Y | Y | Y | Y | Y | Y | Y | Y | Y | N | N | Y | Y | Y | Y | N | N |
| Gaffney (93) | Y | Y | N | Y | Y | DK | DK | Y | Y | Y | Y | Y | DK | N | Y | Y | Y | Y | N | N |
| Galvão (94) | Y | Y | Y | Y | Y | DK | DK | Y | Y | Y | Y | Y | DK | N | Y | Y | Y | N | DK | Y |
| Gan (95) | Y | Y | N | Y | Y | Y | Y | Y | Y | Y | Y | Y | N | N | Y | Y | Y | Y | N | Y |
| Gao (96) | Y | Y | N | Y | Y | Y | Y | Y | Y | Y | Y | Y | N | N | Y | Y | Y | Y | N | Y |
| Garcia-Layana (97) | Y | Y | N | Y | Y | Y | DK | Y | Y | Y | Y | Y | N | N | Y | Y | Y | Y | N | N |
| Garg (98) | Y | Y | N | Y | Y | Y | Y | Y | Y | Y | Y | Y | N | N | Y | Y | Y | Y | N | N |
| Garg (98) | Y | Y | N | Y | Y | DK | DK | Y | Y | Y | Y | Y | DK | N | Y | Y | Y | Y | DK | Y |
| Gebramariam (99) | Y | Y | Y | Y | Y | DK | DK | Y | Y | Y | Y | Y | DK | N | Y | Y | Y | Y | N | Y |
| Geessink (100) | Y | Y | N | Y | Y | Y | Y | Y | Y | Y | Y | Y | N | N | Y | Y | Y | Y | N | Y |
| Gidlow (101) | Y | Y | Y | Y | Y | DK | DK | Y | Y | Y | Y | Y | DK | N | Y | Y | Y | Y | N | Y |
| Glinkowski (102) | Y | Y | DK | Y | Y | Y | Y | Y | Y | Y | Y | Y | N | N | Y | Y | Y | DK | DK | DK |
| Goldstein (103) | Y | Y | N | Y | Y | Y | Y | Y | Y | Y | Y | Y | N | N | Y | Y | Y | Y | N | Y |
| Gopfert (104) | Y | Y | N | Y | Y | Y | Y | Y | Y | Y | Y | Y | N | N | Y | Y | Y | Y | N | Y |
| Gormley (105) | Y | Y | N | Y | Y | DK | DK | Y | Y | Y | Y | Y | DK | N | Y | Y | Y | Y | DK | N |
| Grandizio (106) | Y | Y | N | Y | Y | Y | Y | Y | Y | Y | Y | Y | N | N | Y | Y | Y | Y | DK | Y |
| Greenfield (107) | Y | Y | Y | Y | Y | DK | DK | Y | Y | Y | Y | Y | N | N | Y | Y | Y | Y | N | Y |
| Gregório (108) | Y | Y | N | Y | Y | Y | Y | Y | Y | Y | Y | Y | N | N | Y | Y | Y | Y | N | Y |
| Gudeta (109) | Y | Y | Y | Y | Y | DK | DK | Y | Y | Y | Y | Y | DK | N | Y | Y | Y | Y | N | Y |
| Gunasekera (110) | Y | Y | N | Y | Y | DK | DK | Y | Y | Y | Y | Y | N | N | Y | Y | Y | Y | N | Y |
| Hafeez (111), H. | Y | Y | N | Y | Y | DK | DK | Y | Y | Y | Y | Y | DK | N | Y | Y | Y | N | N | N |
| Hajizadeh (112) | Y | Y | N | Y | Y | DK | DK | Y | Y | Y | Y | Y | DK | N | Y | Y | Y | Y | N | Y |
| Hallensleben (113) | Y | Y | N | Y | Y | DK | DK | Y | Y | Y | Y | Y | DK | N | Y | Y | Y | N | N | Y |
| Halloran (114) | Y | Y | Y | Y | Y | Y | DK | Y | Y | Y | Y | Y | N | N | Y | Y | Y | Y | N | DK |
| Hallquist (115) | Y | Y | Y | Y | Y | Y | Y | Y | Y | Y | Y | Y | N | N | Y | Y | Y | Y | N | DK |
| Hammad (117) | Y | Y | N | Y | Y | DK | DK | Y | Y | Y | Y | Y | DK | N | Y | Y | Y | Y | N | Y |
| Hammersley (118) | Y | Y | Y | Y | Y | DK | DK | Y | Y | Y | Y | Y | DK | N | Y | Y | Y | Y | N | Y |
| Hanna (119) | Y | Y | N | Y | Y | DK | DK | Y | Y | Y | Y | Y | DK | DK | Y | Y | Y | Y | N | Y |
| Hepp (120) | Y | Y | N | Y | Y | DK | DK | Y | Y | Y | Y | Y | DK | N | Y | Y | Y | N | N | Y |
| Hickey (121) | Y | Y | N | Y | Y | DK | DK | Y | Y | Y | Y | Y | DK | N | Y | Y | Y | N | N | Y |
| Higgins (122) | Y | Y | N | Y | Y | Y | Y | Y | Y | N | N | Y | Y | N | Y | Y | Y | N | DK | Y |
| Hilder (123) | Y | Y | N | Y | Y | DK | DK | Y | Y | Y | Y | Y | N | N | Y | Y | Y | Y | N | Y |
| Hirsch (124) | Y | Y | Y | Y | Y | DK | DK | Y | Y | Y | Y | Y | DK | N | Y | Y | Y | Y | N | N |
| Höglander (126) | Y | Y | N | Y | Y | DK | DK | Y | Y | Y | Y | Y | DK | N | Y | Y | Y | Y | N | Y |
| Hohman (127) | Y | Y | Y | Y | Y | Y | Y | Y | Y | Y | Y | Y | N | N | Y | Y | Y | Y | N | N |
| Hori (128) | Y | Y | Y | Y | Y | DK | DK | Y | Y | Y | Y | Y | DK | N | Y | Y | Y | Y | N | Y |
| Hsiao (129) | Y | Y | N | Y | Y | DK | DK | Y | Y | Y | Y | Y | DK | N | Y | Y | Y | Y | N | Y |
| Huang (130) | Y | Y | N | Y | Y | Y | Y | Y | Y | Y | Y | Y | N | N | Y | Y | Y | Y | N | DK |
| Humphries (131) | Y | Y | N | Y | Y | Y | Y | Y | Y | Y | Y | Y | N | N | Y | Y | Y | Y | DK | DK |
| Hunter (132) | Y | Y | N | Y | Y | DK | DK | Y | Y | Y | Y | Y | DK | N | Y | Y | Y | N | N | Y |
| Hutchings (133) | Y | Y | N | Y | Y | Y | N | Y | Y | Y | Y | Y | N | N | Y | Y | Y | Y | N | Y |
| Hwang (134) | Y | Y | N | Y | Y | Y | Y | Y | Y | Y | Y | Y | N | N | Y | Y | Y | Y | N | Y |
| Idrees (135) | Y | Y | Y | Y | Y | DK | DK | Y | Y | Y | Y | Y | DK | N | Y | Y | Y | N | N | Y |
| Iftikhar (136) | Y | Y | N | Y | Y | DK | DK | Y | Y | Y | Y | Y | DK | N | Y | Y | Y | N | DK | Y |
| Iwasa (137) | Y | Y | N | Y | Y | DK | DK | Y | Y | Y | Y | Y | DK | N | Y | Y | Y | Y | N | Y |
| Jabour (138) | Y | Y | N | Y | Y | DK | DK | Y | Y | Y | Y | Y | DK | N | Y | Y | Y | Y | N | Y |
| Jácome (139) | Y | Y | Y | Y | Y | DK | N | Y | Y | Y | Y | Y | Y | N | Y | Y | Y | Y | N | Y |
| Jahromi (140) | Y | Y | Y | Y | Y | DK | DK | Y | Y | Y | Y | Y | DK | N | Y | Y | Y | N | N | Y |
| James (141) | Y | Y | N | Y | Y | Y | Y | Y | Y | Y | Y | Y | N | N | Y | Y | Y | Y | N | Y |
| Janati (142) | Y | Y | N | Y | Y | DK | DK | Y | Y | Y | Y | Y | DK | N | Y | Y | Y | N | N | N |
| Jandovitz (143) | Y | Y | N | Y | Y | Y | Y | Y | Y | Y | Y | Y | N | N | Y | Y | Y | N | N | Y |
| Jegal (144) | Y | Y | N | Y | Y | DK | N | Y | Y | Y | Y | Y | Y | N | Y | Y | Y | Y | DK | Y |
| Jin (145) | Y | Y | Y | Y | Y | Y | DK | Y | Y | Y | Y | Y | DK | N | Y | Y | Y | Y | N | Y |
| Jones (146) | Y | Y | Y | Y | Y | DK | N | Y | Y | Y | Y | N | DK | N | Y | Y | Y | N | N | Y |
| Joshi (147) | Y | Y | N | Y | Y | Y | N | Y | Y | N | N | Y | DK | N | Y | DK | Y | N | Y | DK |
| Kabeya (148) | Y | Y | N | Y | Y | Y | N | Y | Y | Y | Y | Y | Y | N | Y | Y | Y | Y | N | Y |
| Kagedan (149) | Y | Y | N | Y | Y | Y | N | Y | Y | Y | Y | N | N | N | Y | Y | Y | Y | N | Y |
| Kantor (150) | Y | Y | N | Y | Y | Y | DK | Y | Y | Y | Y | N | DK | N | Y | Y | Y | Y | DK | N |
| Karatas (151) | Y | Y | Y | Y | Y | Y | DK | Y | Y | Y | Y | N | DK | N | Y | Y | Y | N | N | Y |
| Karia (152) | Y | Y | N | Y | Y | Y | DK | Y | Y | DK | Y | Y | DK | DK | Y | Y | Y | Y | N | Y |
| Kasturi (153) | Y | Y | Y | Y | Y | Y | Y | Y | Y | Y | Y | Y | N | Y | Y | Y | Y | Y | N | Y |
| Kelly (154) | Y | Y | N | Y | Y | Y | DK | Y | Y | N | N | Y | DK | DK | Y | Y | Y | Y | DK | Y |
| Keyworth (155) | Y | Y | N | Y | Y | Y | N | Y | Y | N | N | Y | N | N | Y | Y | Y | Y | N | Y |
| Khairat (156) | Y | Y | N | Y | Y | Y | N | Y | Y | N | DK | Y | N | DK | Y | Y | Y | Y | N | Y |
| Kieran (157) | Y | Y | N | Y | Y | Y | N | Y | Y | Y | Y | Y | Y | N | Y | Y | Y | N | N | Y |
| Kilduff (158) | Y | Y | N | Y | Y | Y | DK | Y | Y | N | DK | N | DK | DK | Y | Y | Y | Y | N | N |
| Kim (159) | Y | Y | N | Y | Y | DK | N | Y | Y | DK | Y | Y | Y | DK | Y | Y | Y | Y | N | N |
| Kim (160) | Y | Y | N | Y | Y | DK | DK | Y | Y | Y | Y | Y | DK | N | Y | Y | Y | Y | N | N |
| Kleven (161) | Y | Y | Y | Y | Y | DK | DK | Y | Y | Y | Y | Y | DK | N | Y | Y | Y | Y | N | Y |
| Ko (162) | Y | Y | N | Y | Y | Y | DK | Y | Y | Y | Y | Y | N | N | Y | Y | Y | Y | N | Y |
| Kohlert (163) | Y | Y | N | Y | Y | DK | DK | Y | Y | Y | Y | Y | DK | N | Y | Y | Y | Y | N | Y |
| Korn (164) | Y | Y | N | Y | Y | Y | Y | Y | Y | Y | Y | Y | Y | N | Y | Y | Y | Y | DK | Y |
| Kortlever (165) | Y | Y | N | Y | Y | Y | N | Y | Y | Y | Y | Y | N | N | Y | Y | Y | N | N | Y |
| Kottak (166) | Y | Y | N | Y | DK | DK | N | Y | Y | Y | Y | Y | Y | N | Y | Y | DK | Y | Y | Y |
| Kruk (167) | Y | Y | N | Y | Y | DK | DK | Y | Y | Y | Y | Y | DK | N | Y | Y | Y | Y | N | Y |
| Landon (168) | Y | Y | N | Y | Y | Y | N | Y | Y | N | N | Y | DK | N | Y | N | Y | Y | N | Y |
| Laura Ah-Kye (8) | Y | Y | N | Y | Y | Y | Y | Y | Y | Y | Y | Y | N | N | Y | Y | Y | Y | N | N |
| Laurenzi (169) | Y | Y | N | Y | DK | DK | N | Y | Y | N | N | Y | DK | N | Y | Y | Y | Y | N | Y |
| Leask (170) | Y | Y | N | Y | DK | DK | DK | Y | Y | N | Y | Y | DK | N | Y | Y | Y | Y | N | N |
| Lebanova (171) | Y | Y | N | Y | DK | DK | N | Y | Y | DK | Y | Y | Y | N | Y | Y | Y | N | DK | DK |
| Lee (172) | Y | Y | N | Y | Y | Y | N | Y | Y | Y | Y | Y | Y | N | Y | Y | Y | Y | N | Y |
| Lee (173) | Y | Y | N | Y | Y | Y | N | Y | Y | Y | Y | Y | DK | N | Y | Y | Y | Y | N | Y |
| Lehmann (174) | Y | Y | N | Y | Y | Y | Y | Y | Y | Y | Y | Y | N | Y | Y | Y | Y | Y | N | Y |
| Leone (175) | Y | Y | N | Y | Y | Y | Y | Y | Y | Y | Y | Y | N | Y | Y | Y | Y | Y | N | Y |
| Li (176) | Y | Y | N | Y | Y | DK | N | Y | Y | N | N | Y | Y | N | Y | Y | Y | Y | N | Y |
| Lim (177) | Y | Y | N | Y | Y | Y | N | Y | Y | Y | Y | Y | Y | N | Y | Y | Y | Y | N | N |
| Lin (178) | Y | Y | N | Y | Y | Y | N | Y | Y | Y | Y | Y | N | N | Y | Y | Y | Y | DK | DK |
| Lindberg (179) | Y | Y | N | Y | Y | Y | DK | Y | Y | Y | Y | Y | DK | N | Y | Y | Y | Y | N | Y |
| Liu (180) | Y | Y | N | Y | DK | DK | DK | Y | Y | N | N | Y | DK | N | Y | Y | Y | Y | N | Y |
| Liu (181) | Y | Y | N | Y | Y | Y | N | Y | Y | Y | Y | Y | N | N | Y | Y | Y | Y | N | DK |
| Liu (182) | Y | Y | N | Y | Y | Y | N | Y | Y | Y | Y | Y | DK | N | Y | Y | Y | Y | N | Y |
| Livori (183) | Y | Y | Y | Y | Y | Y | Y | Y | Y | Y | Y | Y | N | N | Y | Y | N | Y | N | Y |
| Looi (184) | Y | Y | N | Y | Y | Y | DK | Y | Y | Y | Y | Y | N | N | Y | Y | Y | Y | N | N |
| Lopez (185) | Y | Y | N | Y | Y | Y | DK | Y | N | N | N | Y | DK | N | Y | Y | Y | Y | N | N |
| Lun (186) | Y | Y | N | Y | Y | Y | N | Y | Y | Y | Y | Y | N | N | Y | Y | Y | Y | N | Y |
| Lurquin (187) | Y | Y | N | Y | Y | Y | DK | Y | Y | Y | Y | Y | DK | N | Y | Y | Y | Y | DK | Y |
| Ly (188) | Y | Y | N | Y | Y | Y | N | Y | Y | Y | Y | Y | DK | N | Y | Y | Y | Y | N | DK |
| Mabuto (189) | Y | Y | N | Y | Y | Y | DK | Y | Y | Y | Y | Y | DK | N | Y | Y | Y | Y | N | Y |
| Mahmoud (190) | Y | Y | N | Y | Y | Y | N | Y | Y | Y | Y | Y | N | N | Y | Y | Y | N | N | Y |
| Makhlouf (191) | Y | Y | Y | Y | Y | Y | N | Y | Y | Y | Y | Y | N | N | Y | Y | Y | Y | N | Y |
| Mamo (192) | Y | Y | N | Y | Y | DK | DK | Y | Y | Y | Y | Y | DK | N | Y | Y | Y | Y | N | Y |
| Mandavia (193) | Y | Y | N | Y | Y | Y | Y | Y | Y | Y | Y | Y | N | N | Y | Y | Y | Y | N | Y |
| Martin (194) | Y | Y | N | Y | Y | Y | Y | Y | Y | Y | Y | N | N | N | Y | Y | Y | N | DK | N |
| Martinez (195) | Y | Y | N | Y | Y | Y | Y | Y | Y | Y | N | Y | N | N | Y | Y | Y | Y | N | Y |
| Martinez (196) | Y | Y | N | Y | Y | Y | Y | Y | Y | Y | Y | N | N | N | Y | Y | N | Y | N | Y |
| Marty (197) | Y | Y | N | Y | Y | DK | N | Y | Y | Y | Y | N | Y | N | Y | Y | Y | Y | N | N |
| Mathijssen (198) | Y | Y | N | Y | Y | DK | DK | Y | Y | Y | Y | Y | DK | N | Y | Y | Y | Y | N | Y |
| Matsushita (199) | Y | Y | N | Y | Y | Y | N | Y | Y | Y | Y | Y | N | N | Y | Y | Y | Y | N | Y |
| Matta (200) | Y | Y | N | Y | Y | Y | DK | Y | Y | Y | Y | Y | DK | N | Y | Y | Y | Y | N | Y |
| McBain (201) | Y | Y | Y | Y | Y | Y | DK | Y | Y | N | N | Y | DK | N | Y | Y | Y | Y | N | DK |
| McCabe (202) | Y | Y | Y | Y | Y | Y | N | Y | Y | Y | Y | Y | N | Y | Y | Y | Y | Y | N | Y |
| McGloin (203) | Y | Y | Y | Y | Y | Y | N | Y | Y | Y | Y | Y | DK | N | Y | Y | Y | Y | N | Y |
| McKenzie (204) | Y | Y | N | Y | Y | Y | N | Y | Y | Y | Y | Y | Y | N | Y | Y | Y | Y | N | Y |
| McKirdy (205) | Y | Y | Y | Y | Y | Y | Y | Y | Y | Y | Y | N | N | DK | Y | Y | Y | Y | N | N |
| Mehra (206) | Y | Y | N | Y | Y | Y | N | Y | Y | Y | Y | Y | DK | N | Y | Y | Y | Y | N | Y |
| Meijers (207) | Y | Y | N | Y | Y | Y | DK | Y | Y | Y | Y | Y | DK | N | Y | Y | Y | Y | N | Y |
| Meijers (208) | Y | Y | N | Y | Y | DK | N | Y | Y | Y | Y | Y | Y | N | Y | Y | Y | Y | N | Y |
| Meleis (209) | Y | Y | N | Y | Y | Y | Y | Y | Y | Y | Y | Y | N | N | Y | Y | Y | Y | N | N |
| Melian (210) | Y | Y | N | Y | Y | Y | Y | Y | Y | Y | Y | Y | N | N | Y | Y | Y | Y | N | Y |
| Mensa (211) | Y | Y | Y | Y | Y | DK | DK | Y | Y | Y | Y | Y | DK | N | Y | Y | Y | Y | N | Y |
| Mercer (212) | Y | Y | N | Y | Y | Y | DK | Y | Y | Y | Y | Y | DK | N | Y | Y | Y | Y | N | Y |
| Michael (213) | Y | Y | Y | Y | Y | Y | DK | Y | Y | Y | Y | Y | DK | N | Y | Y | Y | Y | N | Y |
| Milford (215) | Y | Y | N | Y | Y | DK | DK | Y | Y | Y | Y | N | DK | N | Y | Y | Y | Y | N | N |
| Mohamed Ibrahim (216) | Y | Y | N | Y | Y | Y | Y | Y | Y | Y | Y | Y | N | N | Y | Y | Y | Y | N | Y |
| Mohamoud (217) | Y | Y | N | Y | Y | N | N | Y | Y | Y | Y | N | N | N | Y | Y | Y | Y | N | Y |
| Mugada (218) | Y | Y | Y | Y | Y | Y | N | Y | Y | Y | Y | N | N | N | Y | Y | Y | N | N | Y |
| Murphy (219) | Y | Y | N | Y | DK | DK | N | Y | Y | Y | Y | Y | DK | N | Y | Y | Y | Y | N | Y |
| Murren-Boezem (220) | Y | Y | N | Y | Y | Y | Y | Y | Y | Y | Y | Y | N | N | Y | Y | Y | Y | N | N |
| Musser (221) | Y | Y | Y | Y | Y | Y | Y | Y | Y | Y | Y | Y | N | N | Y | Y | Y | Y | N | Y |
| Mustafa (222) | Y | Y | DK | DK | DK | DK | DK | Y | Y | DK | DK | DK | DK | DK | DK | DK | DK | DK | DK | DK |
| Mutemwa (223) | Y | Y | N | N | DK | DK | DK | Y | Y | Y | Y | N | DK | N | Y | Y | Y | Y | N | Y |
| Nagykaldi (224) | Y | Y | N | Y | Y | Y | N | Y | Y | Y | Y | N | DK | N | Y | Y | Y | N | N | Y |
| Nair (225) | Y | Y | N | Y | Y | Y | Y | Y | Y | Y | Y | Y | N | Y | Y | Y | Y | Y | N | Y |
| Nakano (226) | Y | Y | N | Y | Y | Y | Y | Y | Y | Y | Y | Y | N | N | Y | Y | Y | N | DK | N |
| Nathan (227) | Y | Y | N | Y | Y | Y | Y | Y | Y | Y | Y | Y | N | N | Y | Y | Y | N | N | N |
| Neke (228) | Y | Y | N | Y | DK | DK | N | Y | Y | Y | Y | Y | DK | N | Y | Y | Y | Y | N | Y |
| Newell (229) | Y | Y | Y | Y | Y | Y | Y | Y | Y | Y | Y | Y | N | N | Y | Y | Y | Y | N | N |
| Newman-Casey (230) | Y | Y | N | Y | DK | DK | DK | Y | Y | Y | Y | N | DK | N | Y | Y | Y | Y | N | Y |
| NHAMCS (274) | Y | Y | Y | Y | Y | Y | DK | Y | Y | Y | Y | Y | DK | N | Y | Y | Y | N | DK | N |
| Nikjoo (231) | Y | Y | N | Y | DK | DK | DK | Y | Y | Y | Y | Y | DK | N | Y | Y | Y | N | N | Y |
| Nyabuti (232) | Y | Y | N | Y | Y | DK | N | Y | Y | Y | Y | Y | DK | N | Y | Y | Y | N | N | Y |
| Ourth (233) | Y | Y | N | Y | Y | Y | Y | Y | Y | Y | Y | Y | N | N | Y | Y | Y | Y | N | N |
| Pappalardo (234) | Y | Y | N | Y | Y | Y | Y | Y | Y | Y | Y | Y | N | N | Y | Y | Y | Y | N | Y |
| Parikh (235) | Y | Y | N | Y | DK | DK | Y | Y | Y | Y | Y | Y | N | N | Y | Y | Y | Y | N | N |
| Patel (236) | Y | Y | N | Y | Y | Y | DK | Y | Y | Y | Y | Y | DK | N | Y | Y | Y | Y | N | N |
| Paudel (237) | Y | Y | Y | Y | Y | Y | DK | Y | Y | Y | Y | N | DK | N | Y | Y | Y | Y | N | Y |
| Peek (238) | Y | Y | N | Y | DK | DK | N | Y | Y | Y | Y | Y | DK | N | Y | Y | Y | Y | DK | Y |
| Peltzer (239) | Y | Y | N | Y | Y | Y | Y | Y | Y | Y | Y | Y | N | N | Y | Y | Y | Y | N | Y |
| Perdoncini (240) | Y | Y | N | Y | Y | DK | DK | Y | Y | Y | Y | Y | DK | N | Y | Y | Y | Y | N | Y |
| Peters (241) | Y | Y | Y | Y | Y | Y | DK | Y | Y | Y | Y | Y | DK | N | Y | Y | Y | Y | N | Y |
| Pettersson (242) | Y | Y | N | Y | DK | DK | DK | Y | Y | Y | Y | Y | DK | N | Y | Y | Y | Y | N | Y |
| Peurois (243) | Y | Y | N | Y | Y | Y | Y | Y | Y | Y | Y | Y | N | N | Y | Y | Y | N | N | Y |
| Phadke (244) | Y | Y | N | Y | Y | DK | DK | Y | Y | Y | Y | Y | DK | N | Y | Y | Y | Y | N | Y |
| Phommachanh (245) | Y | Y | Y | Y | Y | DK | N | Y | Y | Y | Y | Y | DK | N | Y | Y | Y | Y | N | Y |
| Pierse (246) | Y | Y | N | Y | Y | Y | Y | Y | Y | Y | Y | Y | N | N | Y | Y | Y | Y | N | Y |
| Pilote (247) | Y | Y | Y | Y | Y | Y | N | Y | Y | Y | Y | Y | N | N | Y | Y | Y | Y | N | Y |
| Pintova (248) | Y | Y | N | Y | Y | DK | DK | Y | Y | Y | Y | Y | DK | N | Y | Y | Y | Y | N | Y |
| Pochert (249) | Y | Y | N | Y | Y | Y | Y | Y | Y | Y | Y | Y | N | Y | Y | Y | Y | Y | N | Y |
| Polat (250) | Y | Y | N | Y | Y | DK | DK | Y | Y | Y | Y | Y | DK | N | Y | Y | Y | Y | N | Y |
| Popescu (251) | Y | Y | Y | Y | Y | Y | Y | Y | Y | Y | Y | Y | DK | N | Y | Y | Y | Y | N | Y |
| Pradhan (252) | Y | Y | N | Y | Y | DK | N | Y | Y | Y | Y | Y | DK | N | Y | Y | Y | Y | N | N |
| Puchner (253) | Y | Y | N | Y | Y | Y | Y | Y | Y | Y | Y | Y | N | N | Y | Y | Y | Y | N | Y |
| Qiao (254) | Y | Y | Y | Y | Y | Y | DK | Y | Y | Y | Y | Y | DK | N | Y | Y | Y | Y | N | Y |
| Rahhal (255) | Y | Y | N | Y | Y | Y | Y | Y | Y | Y | Y | Y | N | N | Y | Y | Y | N | DK | N |
| Rainey (256) | Y | Y | N | Y | Y | Y | DK | Y | Y | Y | Y | Y | DK | N | Y | Y | Y | Y | DK | Y |
| Rajput (257) | Y | Y | N | Y | Y | Y | DK | Y | Y | Y | Y | Y | DK | N | Y | Y | Y | Y | DK | Y |
| Rao (258) | Y | Y | Y | Y | Y | Y | DK | Y | Y | Y | Y | Y | N | N | Y | Y | Y | Y | DK | DK |
| Ratwani (259) | Y | Y | N | Y | Y | Y | Y | Y | Y | Y | Y | Y | N | N | Y | Y | Y | Y | N | DK |
| Read-Brown (260) | Y | Y | N | Y | Y | Y | Y | Y | Y | Y | Y | N | Y | N | Y | Y | Y | Y | N | N |
| Reblin (261) | Y | Y | N | Y | Y | DK | DK | Y | Y | Y | Y | Y | DK | N | Y | Y | Y | Y | DK | Y |
| Rej (262) | Y | Y | N | Y | Y | Y | Y | Y | Y | Y | Y | Y | N | N | Y | Y | Y | Y | N | Y |
| Riley (263) | Y | Y | N | Y | Y | Y | Y | Y | Y | Y | Y | Y | N | N | Y | Y | Y | Y | DK | Y |
| Roll (264) | Y | Y | N | Y | Y | Y | Y | Y | Y | Y | Y | Y | N | N | Y | Y | Y | Y | N | N |
| Rutten (265) | Y | Y | N | Y | Y | DK | DK | Y | Y | Y | Y | Y | DK | N | Y | Y | Y | Y | N | N |
| Ryan (266) | Y | Y | N | Y | Y | DK | DK | Y | Y | Y | Y | N | DK | N | Y | Y | Y | Y | N | Y |
| Ryu (267) | Y | Y | N | Y | Y | DK | DK | Y | Y | Y | Y | Y | DK | N | Y | Y | Y | Y | N | Y |
| Rzadkiewicz (268) | Y | Y | N | Y | Y | DK | DK | Y | Y | Y | Y | Y | DK | N | Y | Y | Y | Y | N | Y |
| Saginela (270) | Y | Y | N | Y | Y | DK | DK | Y | Y | Y | Y | Y | DK | N | Y | Y | Y | N | N | Y |
| Salisbury (271) | Y | Y | N | Y | Y | DK | DK | Y | Y | Y | Y | Y | DK | N | Y | Y | Y | Y | N | Y |
| Sanders-Pinheiro (272) | Y | Y | N | Y | Y | DK | DK | Y | Y | Y | Y | Y | DK | N | Y | Y | Y | Y | DK | Y |
| Santo (274) | Y | Y | Y | Y | Y | Y | DK | Y | Y | Y | Y | Y | DK | N | Y | Y | Y | N | DK | N |
| Saunders (275) | Y | Y | N | Y | Y | Y | Y | Y | Y | Y | Y | Y | N | N | Y | Y | Y | Y | N | Y |
| Schäfer (276) | Y | Y | N | Y | Y | DK | DK | Y | Y | Y | Y | Y | DK | N | Y | Y | Y | Y | N | Y |
| Schifeling (277) | Y | Y | N | Y | Y | Y | Y | Y | Y | Y | Y | Y | N | N | Y | Y | Y | Y | N | N |
| Schimmelfing (278) | Y | Y | N | Y | Y | Y | Y | Y | Y | Y | Y | Y | N | N | Y | Y | Y | Y | N | N |
| Schnakenberg (279) | Y | Y | N | Y | Y | DK | N | Y | Y | Y | Y | Y | Y | N | Y | Y | Y | Y | N | Y |
| Sema (280) | Y | Y | Y | Y | Y | DK | DK | Y | Y | Y | Y | Y | DK | N | Y | Y | Y | Y | N | Y |
| Halls (116) | Y | Y | N | Y | Y | DK | DK | Y | Y | Y | Y | Y | DK | N | Y | Y | Y | Y | N | Y |
| Sfar-Gandoura (281) | Y | Y | Y | Y | Y | DK | N | Y | Y | Y | Y | Y | Y | N | Y | Y | Y | Y | Y | Y |
| Shalihin (282) | Y | Y | N | Y | Y | Y | Y | Y | Y | Y | Y | Y | N | N | Y | Y | Y | N | DK | N |
| Shapiro (283) | Y | Y | N | Y | Y | Y | Y | Y | Y | Y | Y | Y | N | N | Y | Y | Y | Y | N | N |
| Shapiro (284) | Y | Y | Y | Y | Y | DK | DK | Y | Y | Y | Y | Y | Y | N | Y | Y | Y | Y | N | Y |
| Shen (285) | Y | Y | N | Y | Y | Y | Y | Y | Y | Y | Y | Y | N | N | Y | Y | Y | Y | N | Y |
| Shim (286) | Y | Y | N | Y | Y | Y | Y | Y | Y | Y | Y | Y | N | Y | Y | Y | Y | Y | N | N |
| Shuaib (287) | Y | Y | N | Y | Y | Y | Y | Y | Y | Y | Y | Y | N | N | Y | Y | Y | Y | N | N |
| Siele (288) | Y | Y | Y | Y | Y | DK | DK | Y | Y | Y | Y | Y | DK | N | Y | Y | Y | Y | N | Y |
| Silver (289) | Y | Y | N | Y | Y | Y | Y | Y | Y | Y | Y | Y | N | N | Y | Y | Y | Y | N | N |
| Singh (290) | Y | Y | N | Y | Y | Y | Y | Y | Y | Y | Y | Y | N | N | Y | Y | Y | N | N | Y |
| Sinha (291) | Y | Y | N | Y | Y | Y | Y | Y | Y | Y | Y | Y | N | N | Y | Y | Y | Y | N | N |
| Sisay (292) | Y | Y | Y | Y | Y | DK | DK | Y | Y | Y | Y | Y | DK | N | Y | Y | Y | Y | N | Y |
| Skrepnek (293) | Y | Y | N | Y | Y | Y | Y | Y | Y | Y | Y | Y | N | N | Y | Y | Y | Y | N | N |
| Smits (294) | Y | Y | Y | Y | Y | Y | Y | Y | Y | Y | Y | Y | N | N | Y | Y | Y | Y | N | Y |
| Soegaard (295) | Y | Y | Y | Y | Y | Y | Y | Y | Y | Y | Y | Y | N | Y | Y | Y | Y | Y | DK | Y |
| Søndergaard (296) | Y | Y | N | Y | Y | DK | N | Y | Y | Y | Y | Y | DK | N | Y | Y | N | Y | N | Y |
| Stark (297) | Y | Y | N | Y | Y | Y | DK | Y | Y | Y | Y | Y | DK | N | Y | Y | Y | Y | DK | Y |
| Stevens (298) | Y | Y | N | Y | Y | Y | Y | Y | Y | Y | Y | Y | N | N | Y | Y | Y | Y | N | Y |
| Stewart (299) | Y | Y | N | Y | DK | DK | DK | Y | Y | Y | Y | Y | DK | N | Y | Y | Y | Y | N | N |
| Stewart (300) | Y | Y | N | Y | Y | Y | Y | Y | Y | Y | Y | Y | DK | N | Y | Y | Y | Y | N | Y |
| Stewart (301) | Y | Y | N | Y | Y | Y | Y | Y | Y | Y | Y | Y | N | N | Y | Y | Y | Y | N | Y |
| Stime (302) | Y | Y | N | Y | Y | Y | Y | Y | Y | Y | Y | Y | N | N | Y | Y | Y | N | N | Y |
| Sumargono (303) | Y | Y | N | Y | Y | DK | DK | Y | Y | Y | Y | Y | DK | N | Y | Y | Y | Y | N | Y |
| Swami (304) | Y | Y | N | Y | Y | Y | Y | Y | Y | Y | Y | Y | Y | N | Y | Y | Y | N | N | N |
| Talyshinskii (305) | Y | Y | N | Y | Y | Y | DK | Y | Y | Y | Y | Y | DK | N | Y | Y | Y | Y | N | Y |
| Tampi (306) | Y | Y | N | Y | Y | Y | DK | Y | Y | Y | Y | Y | DK | N | Y | Y | Y | Y | N | Y |
| Tassew (307) | Y | Y | Y | Y | Y | DK | DK | Y | Y | Y | Y | Y | DK | N | Y | Y | Y | Y | N | Y |
| Taylor (308) | Y | Y | N | Y | Y | DK | DK | Y | Y | Y | Y | Y | DK | N | Y | Y | Y | Y | Y | Y |
| Teklemariam (309) | Y | Y | Y | Y | Y | DK | DK | Y | Y | Y | Y | Y | DK | N | Y | Y | Y | N | DK | Y |
| Tenforde (310) | Y | Y | N | Y | Y | DK | DK | Y | Y | Y | Y | Y | DK | N | Y | Y | Y | Y | N | Y |
| Thapa (311) | Y | Y | N | Y | Y | Y | Y | Y | Y | Y | Y | Y | N | N | Y | Y | Y | N | N | N |
| Thotam (312) | Y | Y | N | Y | Y | DK | DK | Y | Y | Y | Y | Y | DK | N | Y | Y | Y | Y | DK | N |
| Tilburt (313) | Y | Y | Y | Y | Y | Y | Y | Y | Y | Y | Y | Y | N | N | Y | Y | Y | Y | N | Y |
| Tilburt (314) | Y | Y | N | Y | Y | Y | Y | Y | Y | Y | Y | Y | N | N | Y | Y | Y | N | DK | Y |
| Timmer (315) | Y | Y | N | Y | Y | Y | N | Y | Y | Y | Y | Y | DK | N | Y | Y | Y | Y | N | Y |
| Ting (316) | Y | Y | N | Y | Y | DK | N | Y | Y | Y | Y | Y | DK | N | Y | Y | Y | Y | N | Y |
| Tiruneh (317) | Y | Y | Y | Y | Y | Y | N | Y | Y | Y | Y | Y | DK | N | Y | Y | Y | Y | DK | Y |
| Tranter‐Entwistle (319) | Y | Y | N | Y | Y | DK | N | Y | Y | Y | Y | Y | DK | N | Y | Y | Y | Y | N | N |
| Tsvyakh (320) | Y | Y | N | Y | Y | Y | Y | Y | Y | Y | Y | Y | N | N | Y | Y | Y | N | N | Y |
| Van Dril (321) | Y | Y | N | Y | Y | DK | DK | Y | Y | Y | Y | Y | DK | N | Y | Y | Y | Y | N | Y |
| Van Veenendaal (322) | Y | Y | N | Y | Y | DK | DK | Y | Y | Y | Y | Y | DK | N | Y | Y | Y | Y | N | Y |
| Vedanthan (323) | Y | Y | N | Y | Y | DK | DK | Y | Y | Y | Y | Y | DK | N | Y | Y | Y | Y | N | Y |
| Vilendrer (324) | Y | Y | N | Y | Y | Y | Y | Y | Y | Y | Y | Y | N | N | Y | Y | Y | Y | N | N |
| Vogt (325) | Y | Y | N | Y | Y | Y | Y | Y | Y | Y | Y | Y | Y | N | Y | Y | Y | Y | Y | N |
| von dem Knesebeck (326) | Y | Y | N | Y | Y | DK | DK | Y | Y | Y | Y | Y | DK | N | Y | Y | Y | Y | N | N |
| Vyas (327) | Y | Y | N | Y | Y | Y | Y | Y | Y | Y | Y | Y | N | N | Y | Y | Y | Y | N | N |
| Waller (328) | Y | Y | N | Y | Y | Y | Y | Y | Y | Y | Y | Y | N | N | Y | Y | Y | Y | N | Y |
| Walling (329) | Y | Y | N | Y | Y | Y | Y | Y | Y | Y | Y | Y | N | N | Y | Y | Y | Y | N | N |
| Wang (330) | Y | Y | N | Y | Y | Y | Y | Y | Y | Y | Y | Y | N | N | Y | Y | Y | Y | N | Y |
| Wang (408) | Y | Y | N | Y | Y | DK | DK | Y | Y | Y | Y | Y | DK | N | Y | Y | Y | Y | N | N |
| Wazaify (331) | Y | Y | N | Y | Y | Y | Y | Y | Y | Y | Y | Y | N | N | Y | Y | Y | Y | N | Y |
| Wei (332) | Y | Y | N | Y | Y | DK | N | Y | Y | Y | Y | Y | N | N | Y | Y | Y | Y | N | Y |
| Weise (333) | Y | Y | N | Y | Y | Y | Y | Y | Y | Y | Y | Y | N | N | Y | Y | Y | Y | DK | Y |
| Weiss (334) | Y | Y | N | Y | Y | DK | DK | Y | Y | Y | Y | Y | Y | N | Y | Y | Y | Y | N | Y |
| Wendie (335) | Y | Y | Y | Y | Y | DK | DK | Y | Y | Y | Y | Y | DK | N | Y | Y | Y | Y | N | Y |
| Weyer (336) | Y | Y | Y | Y | Y | DK | DK | Y | Y | Y | Y | Y | N | N | Y | Y | Y | Y | N | Y |
| Willems (337) | Y | Y | N | Y | Y | Y | DK | Y | Y | Y | Y | Y | N | N | Y | Y | Y | Y | N | Y |
| Willging (338) | Y | Y | Y | Y | Y | Y | Y | Y | Y | Y | Y | Y | N | N | Y | Y | Y | Y | N | Y |
| Williams (339) | Y | Y | N | Y | Y | DK | DK | Y | Y | Y | Y | Y | DK | N | Y | Y | Y | Y | N | Y |
| Winkelman (340) | Y | Y | N | Y | Y | Y | Y | Y | Y | Y | Y | Y | N | N | Y | Y | Y | Y | N | N |
| Wogayehu (341) | Y | Y | N | Y | Y | Y | Y | Y | Y | Y | Y | Y | N | N | Y | Y | Y | Y | N | Y |
| Wong (342) | Y | Y | N | Y | Y | DK | DK | Y | Y | Y | Y | Y | Y | N | Y | Y | Y | Y | N | Y |
| Wood (343) | Y | Y | N | Y | Y | Y | Y | Y | Y | Y | Y | Y | N | N | Y | Y | Y | Y | N | N |
| Xie (344) | Y | Y | N | Y | Y | DK | DK | Y | Y | Y | Y | Y | DK | N | Y | Y | Y | Y | N | Y |
| Yilma (345) | Y | Y | N | Y | Y | DK | DK | Y | Y | Y | Y | Y | DK | N | Y | Y | Y | N | N | Y |
| Young (346) | Y | Y | N | Y | Y | DK | DK | Y | Y | Y | Y | Y | DK | N | Y | Y | Y | Y | N | Y |
| Yuan (347) | Y | Y | N | Y | Y | DK | DK | Y | Y | Y | Y | N | DK | N | Y | Y | Y | Y | N | Y |
| Yuan (352) | Y | Y | N | Y | Y | Y | Y | Y | Y | Y | Y | Y | N | Y | Y | Y | Y | Y | DK | Y |
| Zakare-Fagbamila (348) | Y | Y | N | Y | Y | Y | Y | Y | Y | Y | Y | N | N | N | Y | Y | Y | Y | N | Y |
| Zhang (349) | Y | Y | N | Y | Y | Y | Y | Y | Y | Y | Y | Y | N | N | Y | Y | Y | Y | N | N |
| Zhong (409) | Y | Y | Y | Y | Y | DK | DK | Y | Y | Y | Y | Y | DK | N | Y | Y | Y | Y | N | Y |
| Zimenkovsky (350) | Y | Y | N | Y | Y | Y | DK | Y | Y | Y | Y | Y | DK | N | Y | Y | Y | N | N | Y |
| Zivanovic (351) | Y | Y | Y | Y | Y | Y | Y | Y | Y | Y | Y | Y | N | N | Y | Y | Y | N | DK | DK |
| Abbreviations: DK, don’t know; N, No; Y, Yes. | | | | | | | | | | | | | | | | | | | | |

| **Author** | **1** | **2** | **3** | **4** | **5** | **6** | **7** | **8** | **9** | **10** | **11** | **12** | **13** | **14** | **15** | **16** | **17** | **18** | **19** | **20** |
| --- | --- | --- | --- | --- | --- | --- | --- | --- | --- | --- | --- | --- | --- | --- | --- | --- | --- | --- | --- | --- |
| Ahmed (353) | Y | Y | N | Y | Y | Y | DK | Y | Y | Y | Y | Y | DK | N | Y | Y | Y | Y | N | Y |
| Anugraha (354) | Y | DK | N | N | DK | DK | N | N | DK | N | N | N | DK | N | Y | DK | DK | Y | N | N |
| Al Halabi (355) | Y | Y | N | Y | Y | Y | Y | Y | Y | Y | Y | Y | N | N | Y | Y | Y | Y | N | N |
| Aron (356) | Y | Y | Y | Y | Y | Y | DK | Y | Y | Y | Y | Y | DK | DK | Y | Y | Y | Y | N | Y |
| Babalola (357) | Y | Y | N | Y | Y | Y | Y | Y | Y | Y | Y | Y | N | N | Y | Y | Y | Y | N | Y |
| Babayoff (358) | Y | Y | N | N | DK | DK | DK | Y | Y | N | Y | Y | N | DK | N | Y | Y | Y | N | Y |
| Baghus (359) | Y | Y | N | Y | DK | DK | Y | Y | Y | Y | Y | Y | N | N | Y | Y | Y | Y | N | Y |
| Bos-van den Hoek (360) | Y | Y | Y | Y | DK | DK | Y | Y | Y | Y | Y | Y | N | N | Y | Y | Y | Y | N | Y |
| Bryl (361) | Y | Y | Y | Y | Y | DK | Y | Y | Y | Y | Y | Y | N | N | Y | Y | Y | Y | N | Y |
| Chukwusa (362) | Y | Y | Y | Y | Y | Y | DK | Y | Y | Y | Y | Y | N | N | Y | Y | Y | Y | N | Y |
| Consolandi (363) | Y | Y | N | Y | Y | N | Y | Y | Y | Y | Y | N | N | Y | Y | Y | Y | Y | N | Y |
| Couch (364) | Y | Y | N | Y | Y | Y | Y | Y | Y | Y | Y | Y | N | Y | Y | Y | Y | Y | N | Y |
| Drennan (365) | Y | Y | N | Y | DK | DK | N | Y | Y | Y | Y | Y | N | N | Y | Y | Y | Y | N | Y |
| Ebbers (77) | Y | Y | N | Y | Y | DK | DK | Y | Y | Y | Y | Y | DK | N | Y | Y | Y | Y | DK | Y |
| El-Dahiyat (367) | Y | Y | Y | Y | Y | Y | DK | Y | Y | Y | Y | Y | Y | DK | N | Y | Y | Y | N | Y |
| Fisher (368) | Y | Y | N | N | Y | Y | N | Y | Y | Y | Y | Y | DK | N | Y | Y | Y | Y | N | N |
| Fisher 2023 (369) | Y | Y | Y | Y | Y | Y | N | Y | Y | Y | Y | Y | DK | N | Y | Y | Y | Y | N | Y |
| Fritz (370) | Y | Y | Y | Y | Y | Y | Y | Y | Y | Y | Y | Y | N | Y | Y | Y | Y | Y | N | Y |
| Goruntla (371) | Y | Y | Y | Y | Y | Y | DK | Y | Y | Y | Y | Y | DK | N | Y | Y | Y | Y | N | Y |
| Harris (372) | Y | Y | N | Y | DK | DK | DK | Y | Y | Y | Y | Y | DK | N | Y | Y | Y | Y | N | Y |
| Harrison (373) | Y | Y | Y | Y | Y | Y | DK | Y | Y | N | Y | N | N | N | Y | Y | Y | Y | N | Y |
| Joshi (147) | Y | Y | Y | Y | Y | Y | Y | Y | Y | Y | Y | Y | N | N | Y | Y | Y | Y | N | Y |
| Katzan (376) | Y | Y | N | N | DK | DK | DK | Y | Y | Y | Y | N | DK | N | Y | Y | Y | N | N | N |
| Khan (378) | Y | Y | N | Y | DK | DK | DK | Y | Y | Y | Y | Y | DK | N | Y | Y | Y | Y | N | Y |
| Khan 2022 (378) | Y | Y | Y | Y | Y | DK | Y | Y | Y | Y | Y | Y | DK | N | Y | Y | Y | Y | N | Y |
| Kim (379) | Y | Y | DK | DK | DK | DK | DK | DK | DK | Y | N | N | DK | N | DK | Y | Y | Y | N | Y |
| Klarman (380) | Y | Y | N | Y | Y | DK | DK | Y | Y | Y | Y | Y | DK | N | Y | Y | Y | Y | N | Y |
| Koch (381) | Y | Y | N | Y | DK | DK | DK | Y | Y | Y | Y | Y | DK | N | Y | Y | Y | N | DK | DK |
| Liljenquist (374) | Y | Y | N | Y | DK | DK | DK | Y | Y | Y | Y | Y | DK | N | Y | Y | Y | N | N | N |
| Mailankody (382) | Y | Y | N | Y | Y | Y | Y | Y | Y | Y | Y | Y | N | N | Y | Y | Y | Y | N | Y |
| Maria (383) | Y | Y | Y | Y | Y | Y | N | Y | Y | Y | Y | Y | N | N | Y | Y | Y | N | N | Y |
| Marshall (384) | Y | Y | Y | Y | Y | Y | Y | Y | Y | Y | Y | Y | N | N | Y | Y | Y | Y | N | N |
| Michel (385) | Y | Y | N | Y | Y | Y | Y | Y | Y | Y | Y | Y | N | N | Y | Y | Y | Y | N | Y |
| Mitaly (386) | Y | Y | N | Y | Y | Y | Y | Y | Y | N | Y | N | N | N | Y | Y | Y | N | N | N |
| Mukora (387) | Y | Y | N | Y | Y | Y | Y | Y | Y | Y | Y | Y | N | N | Y | Y | Y | Y | N | Y |
| Mwamba (388) | Y | Y | N | DK | DK | DK | Y | Y | Y | DK | Y | Y | N | N | Y | Y | Y | Y | N | Y |
| Nana Hill (389) | Y | Y | N | Y | Y | Y | N | Y | Y | Y | Y | Y | DK | N | Y | Y | Y | Y | N | Y |
| Ospina (390) | Y | Y | N | Y | DK | DK | DK | Y | Y | Y | Y | Y | DK | N | Y | Y | Y | Y | N | Y |
| Ozkardes (391) | Y | Y | N | Y | Y | Y | Y | Y | Y | Y | Y | Y | N | Y | Y | Y | Y | Y | N | Y |
| Papadopoulos (392) | Y | Y | N | Y | Y | Y | N | Y | Y | Y | Y | Y | DK | N | Y | Y | Y | Y | N | Y |
| Prather (393) | - | - | - | - | - | - | - | - | - | - | - | - | - | - | - | - | - | - | - | - |
| Rahaman (394) | Y | Y | N | Y | DK | DK | DK | Y | Y | Y | Y | Y | DK | N | Y | Y | Y | Y | N | Y |
| Schroder (395) | Y | Y | N | N | Y | Y | DK | Y | Y | Y | Y | Y | N | Y | Y | Y | Y | Y | N | Y |
| Scott (396) | Y | Y | N | Y | Y | Y | DK | Y | Y | Y | Y | Y | DK | Y | Y | Y | Y | Y | N | Y |
| Sharma (397) | Y | Y | N | Y | Y | Y | DK | Y | Y | Y | Y | Y | DK | N | Y | Y | Y | Y | N | Y |
| Shaw (398) | Y | Y | N | Y | Y | DK | DK | Y | Y | Y | Y | Y | DK | N | Y | Y | Y | Y | N | Y |
| Stewart (300) | Y | Y | N | DK | DK | DK | DK | Y | Y | Y | Y | Y | DK | N | Y | Y | Y | Y | N | Y |
| Sugiura (399) | Y | Y | Y | Y | Y | Y | Y | Y | Y | Y | Y | Y | N | N | Y | Y | Y | Y | N | Y |
| Sumargono (303) | Y | Y | N | N | DK | DK | Y | Y | Y | N | Y | Y | N | N | Y | Y | Y | Y | N | N |
| Tan (400) | Y | Y | N | Y | Y | Y | DK | DK | Y | N | Y | Y | N | Y | Y | Y | Y | Y | N | Y |
| Tanner (401) | Y | Y | N | Y | Y | Y | Y | Y | Y | Y | Y | Y | N | N | Y | Y | Y | Y | N | Y |
| Vucak (402) | Y | Y | Y | Y | Y | Y | DK | Y | Y | Y | Y | Y | DK | Y | Y | Y | Y | Y | N | Y |
| Ward(404) | Y | Y | N | Y | Y | Y | Y | Y | Y | Y | Y | Y | N | N | Y | Y | Y | Y | N | Y |
| Willging (338) | Y | Y | N | N | DK | DK | DK | Y | Y | Y | Y | Y | N | N | Y | Y | Y | Y | N | Y |
| Wisniewski (405) | Y | Y | N | Y | Y | Y | DK | Y | Y | Y | Y | Y | DK | N | Y | Y | Y | Y | N | N |
| Yoon (406) | Y | Y | N | N | DK | DK | N | Y | Y | Y | Y | Y | N | N | Y | Y | Y | Y | N | Y |
| Zhan (407) | Y | Y | N | Y | Y | Y | Y | Y | Y | Y | Y | Y | N | N | Y | Y | Y | Y | N | Y |

PRISMA diagram – all results


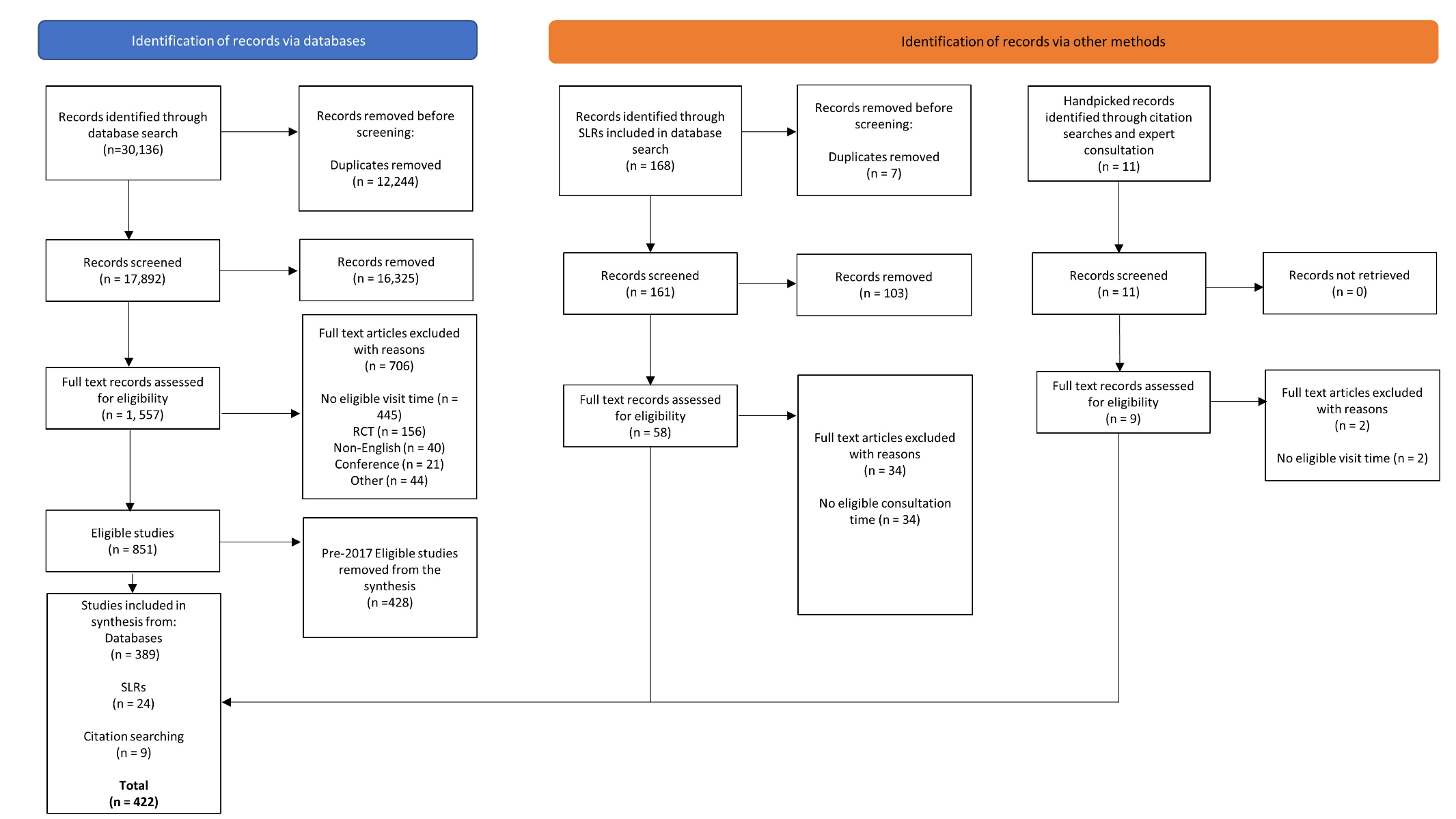


PRISMA diagram – Original search


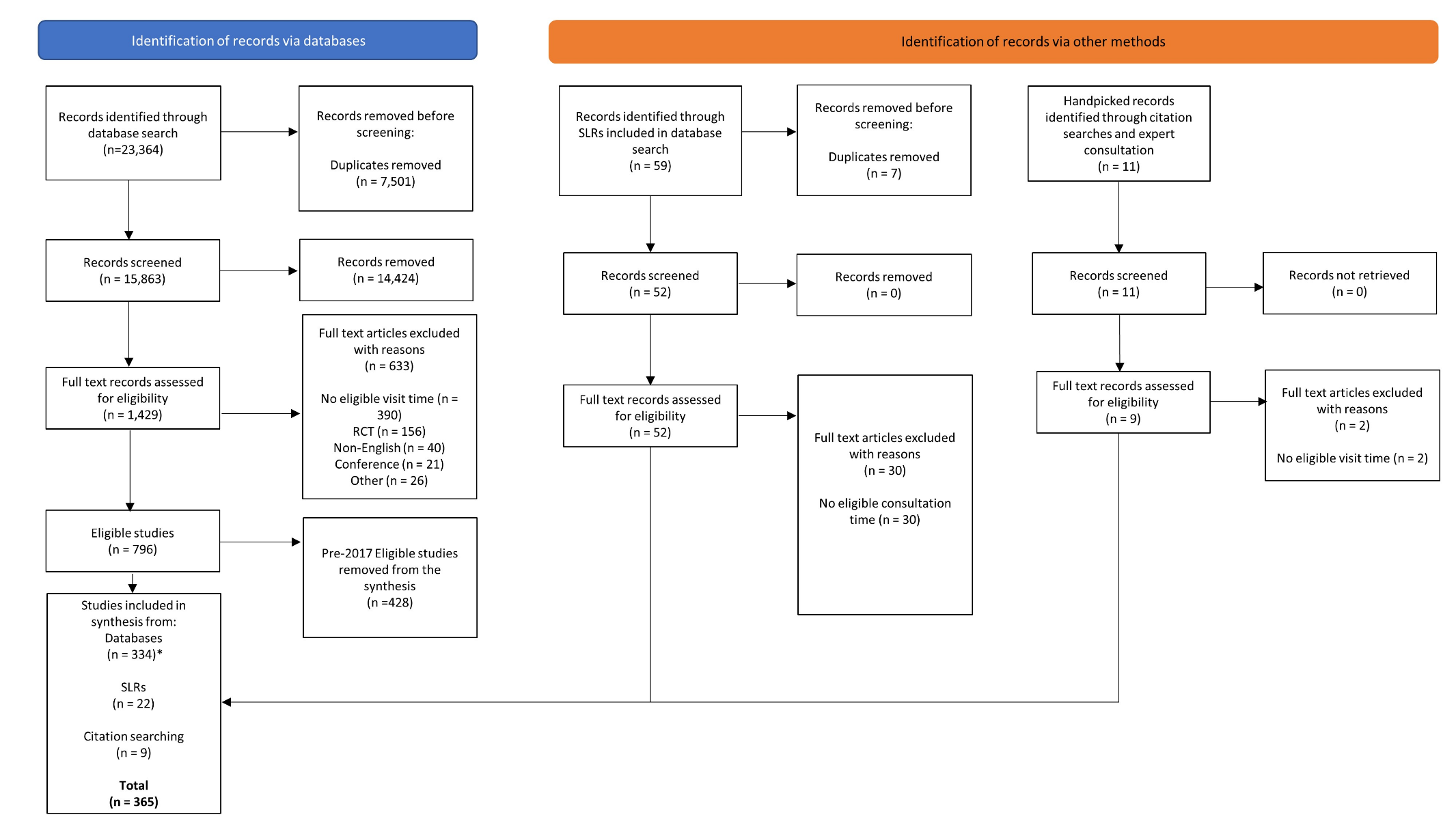


PRISMA diagram – Updated search


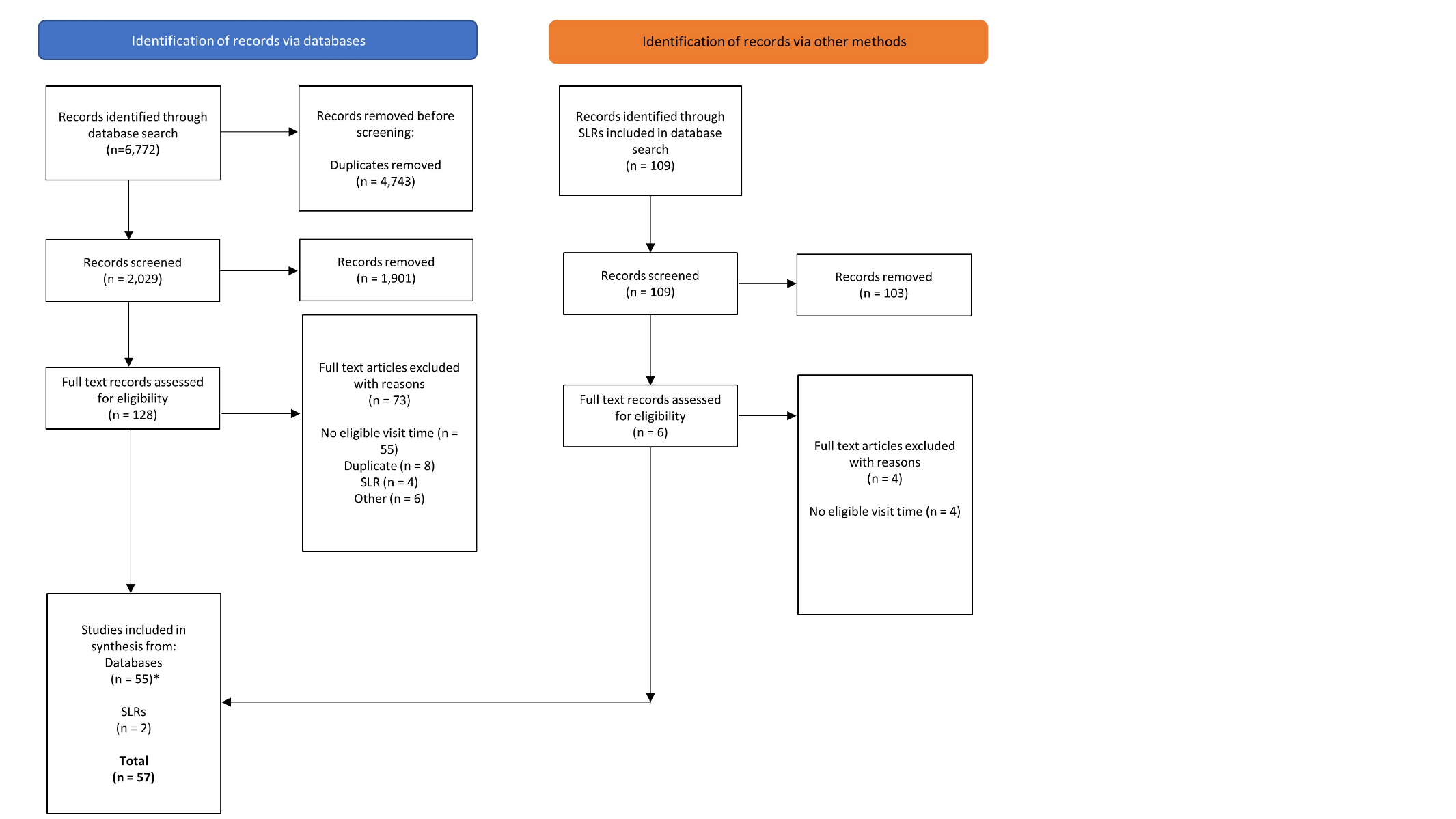


## A6 Evidence density plots for meta-analytic studies

Evidence map, studies included in the meta-analysis – service delivery platform


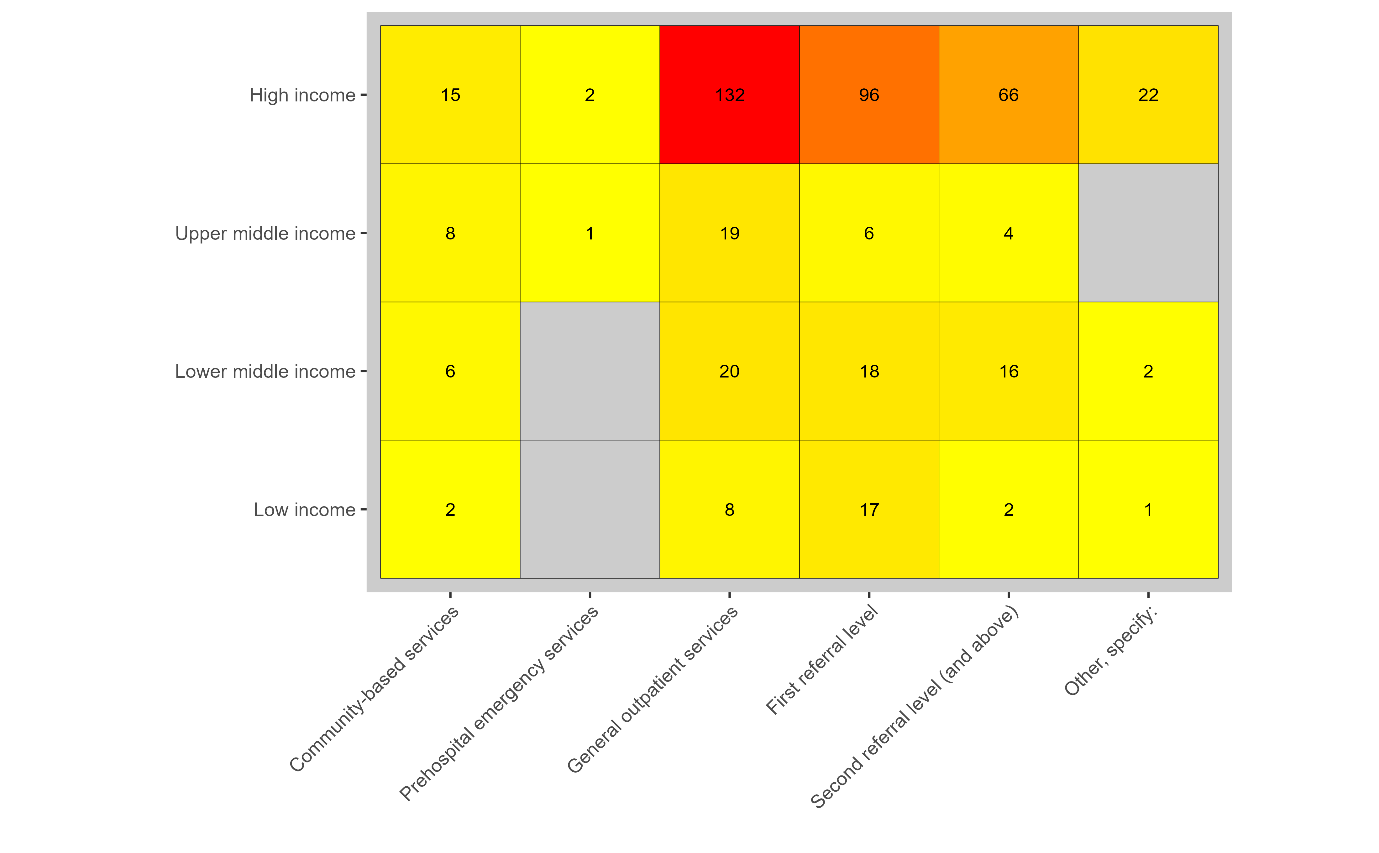


## A7 Forest plots

**Forest plot of the visit times for GPs**


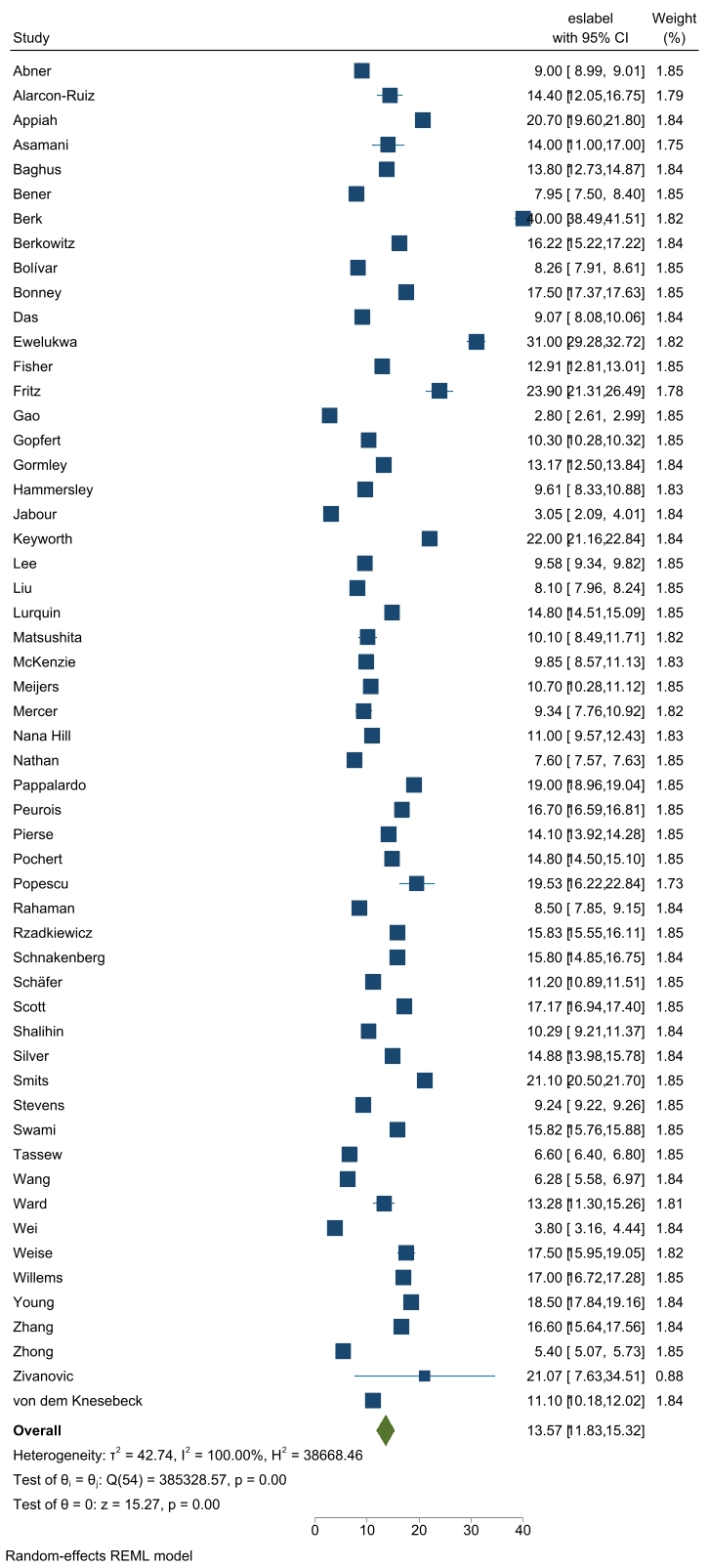


**Figure 17 – Forest plot of the visit times for specialist physicians**


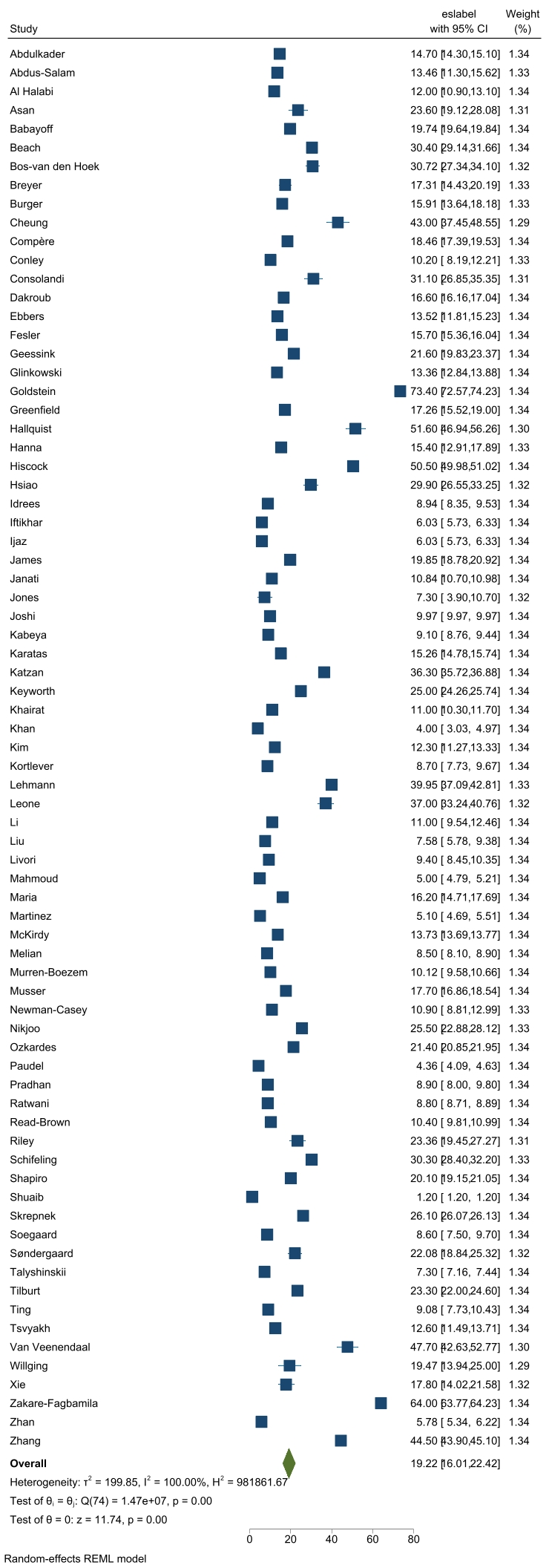


**Figure 18 – Forest plot of the visit times for nurses**


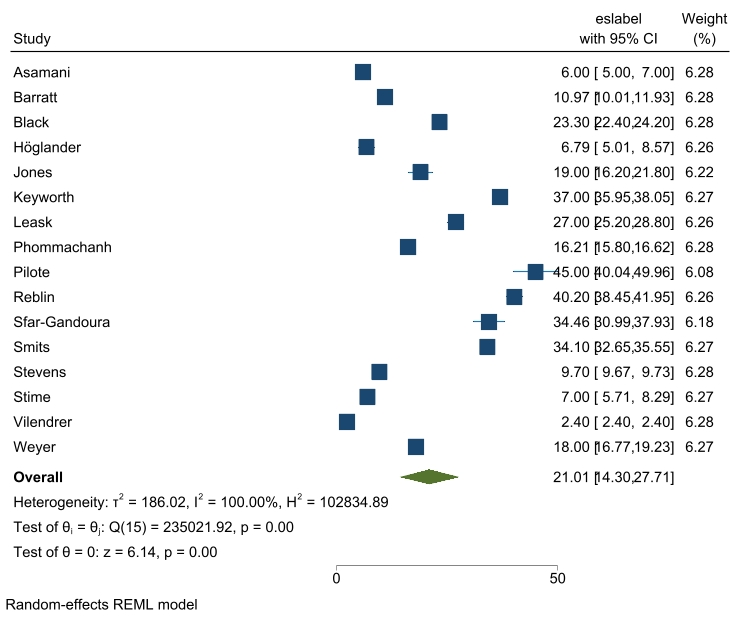


**Figure 19 – Forest plot of the visit times for community-based services**


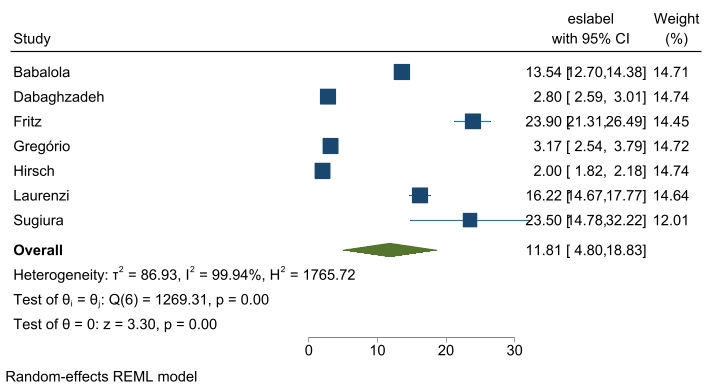


**Figure 20 – Forest plot of the visit times for general outpatient services**


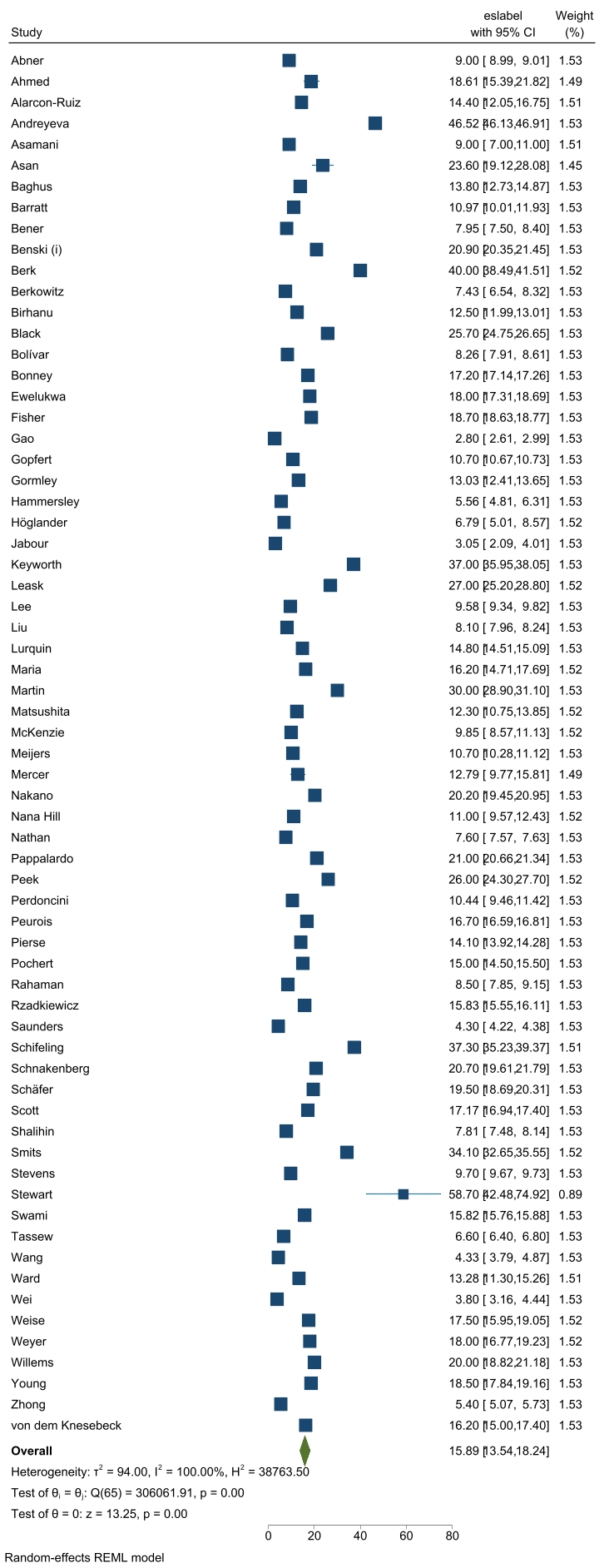


**Figure 21 – Forest plot of the visit times for first-referral level**


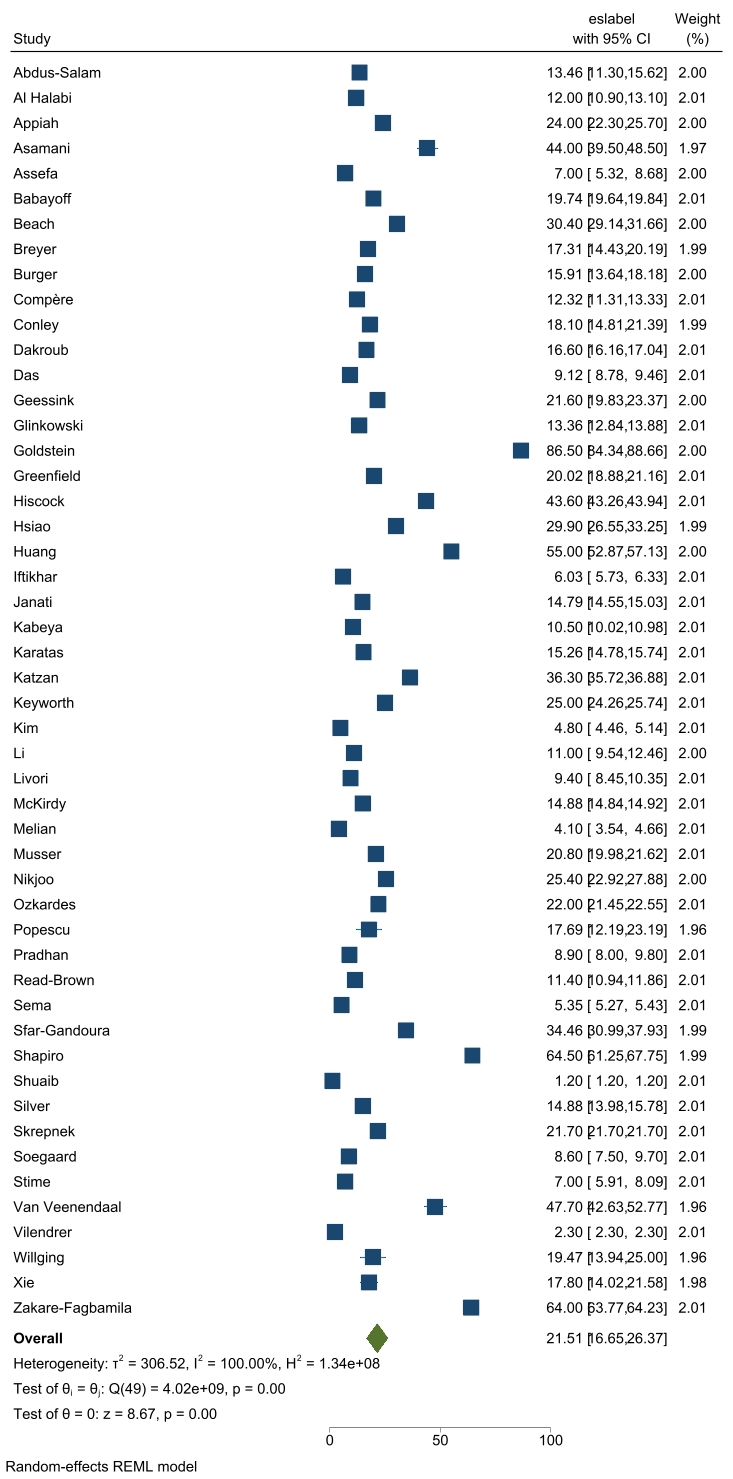


**Figure 22 – Forest plot of the visit times for second-referral level**


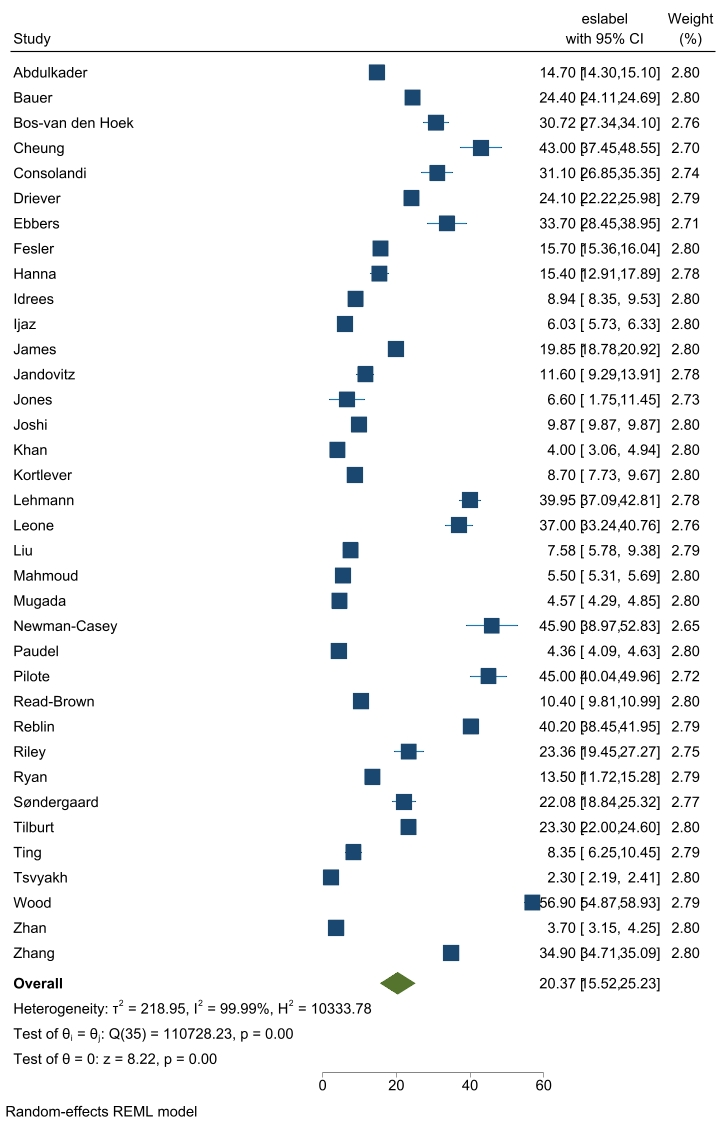


**Figure 23 – Forest plot of the visit times for initial visits**


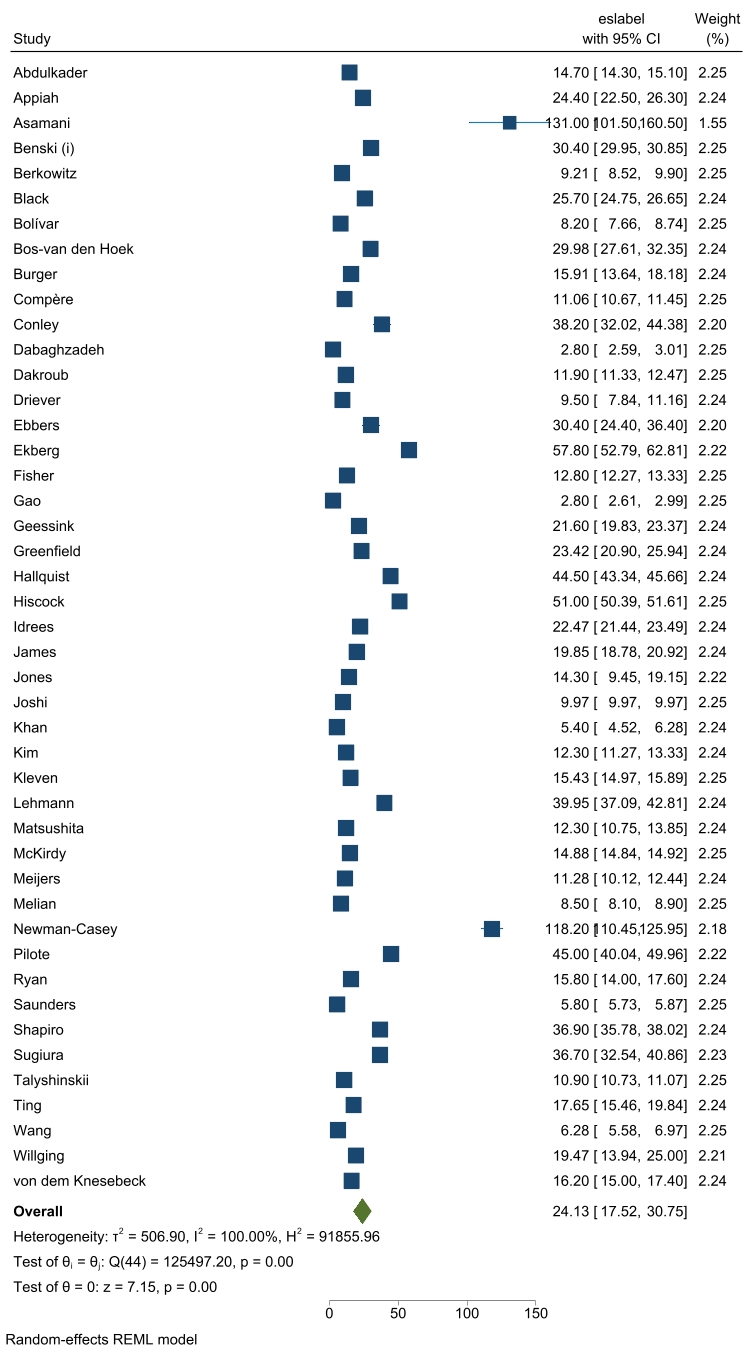


**Figure 24 – Forest plot of the visit times for follow-up visits**


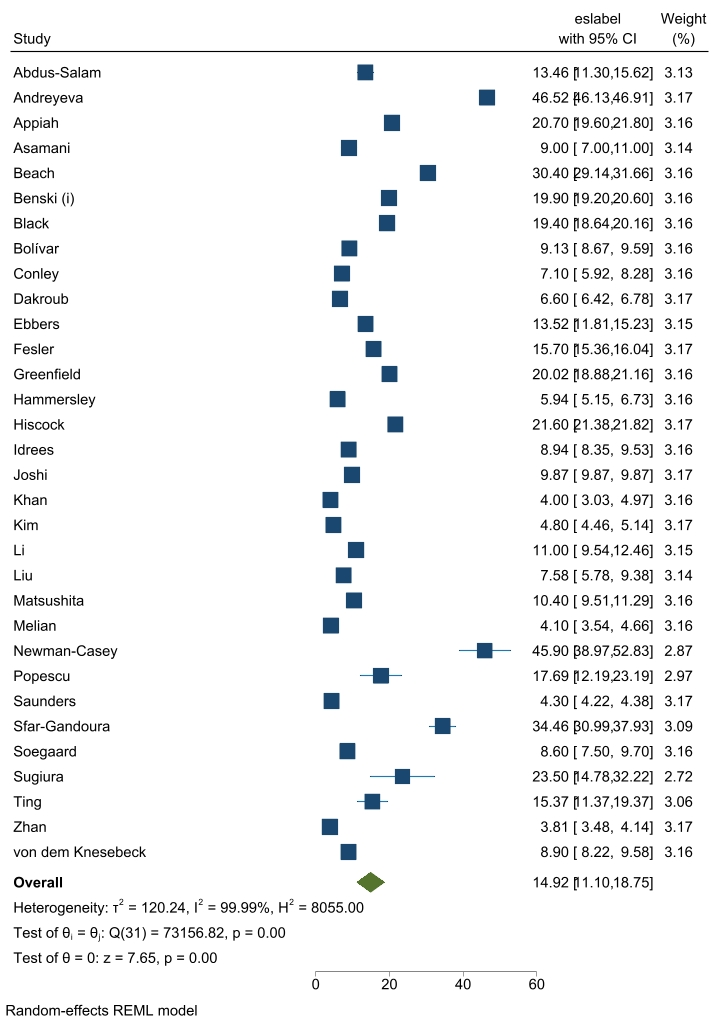


## A8 Meta regression models

Service delivery platform - Model 1

$${visit time}_{ij} = const + \beta_{1}{Income}_{ij} + \beta_{1}{SDP}_{ij} + \beta_{1}{VT}_{ij}+ \beta_{1}{Tele}_{ij}+ u_{ij}$$

Service delivery platform - Model 2

$${visit time}_{ij} = const + \beta_{1}{Income}_{ij} + \beta_{1}{SDP}_{ij} + \beta_{1}{VT}_{ij} + \beta_{1}{Tele}_{ij}+ \beta_{1}{COVID}_{ij} + u_{ij}$$

Service delivery platform - Model 3

$${visit time}_{ij} = const + \beta_{1}{Income}_{ij} + \beta_{1}{SDP}_{ij}+ \beta_{1}{Tele}_{ij} + \beta_{1}{COVID}_{ij} + u_{ij}$$

Service delivery platform - Model 4

$${visit time}_{ij} = const + \beta_{1}{Income}_{ij} + \beta_{1}{SDP}_{ij} + \beta_{1}{VT}_{ij} + u_{ij}$$

Heath worker – Model 1

$${visit time}_{ij} = const + \beta_{1}{Income}_{ij} + \beta_{1}{HCW}_{ij} + \beta_{1}{VT}_{ij}+ \beta_{1}{Tele}_{ij}+ u_{ij}$$

Heath worker – Model 2

$${visit time}_{ij} = const + \beta_{1}{Income}_{ij} + \beta_{1}{HCW}_{ij} + \beta_{1}{VT}_{ij} + \beta_{1}{Tele}_{ij} + \beta_{1}{COVID}_{ij} + u_{ij}$$

Heath worker – Model 3

$${visit time}_{ij} = const + \beta_{1}{Income}_{ij} + \beta_{1}{HCW}_{ij} +\beta_{1}{Tele}_{ij} + \beta_{1}{COVID}_{ij} + u_{ij}$$

Heath worker – Model 4

$${visit time}_{ij} = const + \beta_{1}{Income}_{ij} + \beta_{1}{HCW}_{ij} + u_{ij}$$

The following estimations are limited to the data on doctor visit times only.

Doctor-only – model 1

$${visit time}_{ij} = const + \beta_{1}{Income}_{ij} + \beta_{1}{SDP}_{ij} + \beta_{1}{VT}_{ij} + \beta_{1}{Tele}_{ij} + u_{ij}$$

Doctor-only – model 2

$${visit time}_{ij} = const + \beta_{1}{Income}_{ij} + \beta_{1}{SDP}_{ij} + \beta_{1}{VT}_{ij} + \beta_{1}{Tele}_{ij} + \beta_{1}{COVID}_{ij} + u_{ij}$$

Doctor-only – model 3

$${visit time}_{ij} = const + \beta_{1}{Income}_{ij} + \beta_{1}{SDP}_{ij} + \beta_{1}{Tele}_{ij}+ \beta_{1}{COVID}_{ij} + u_{ij}$$

Doctor-only – model 4

$${visit time}_{ij} = const + \beta_{1}{Income}_{ij} + \beta_{1}{SDP}_{ij} + \beta_{1}{Tele}_{ij} + u_{ij}$$

Doctor-only – model 5

$${visit time}_{ij} = const + \beta_{1}{Income}_{ij} + \beta_{1}{SDP}_{ij} + \beta_{1}{VT}_{ij} + u_{ij}$$

Where visit time_ij_*is the*i*-th estimate* of visit time reported in the j-th study. Income represents the country income classification; SDP represents the service delivery platform; HCW represents the health worker; tele represents studies which occurred via telemedicine and COVID represents which the visit time happened during the COVID-19 pandemic.

**Model results**

|  | **SDP – model 1** |  |  |  |  |  |
| --- | --- | --- | --- | --- | --- | --- |
|  | Coef | Std Error | dfs | p-value | 95%CI L | 95%CI U |
| HIC, UMIC | 4.8953 | 1.8759 | 27.5204 | 0.0145 | 1.0496 | 8.7409 |
| LMIC, LIC | . | . | . | . | . | . |
| Community | -7.1828 | 3.6546 | 9.9437 | 0.0779 | -15.3319 | 0.9663 |
| GOP | -2.4326 | 3.3625 | 11.5343 | 0.4838 | -9.7918 | 4.9265 |
| 1st & 2nd referral | -1.1338 | 2.8323 | 10.1727 | 0.6972 | -7.4301 | 5.1626 |
| Other | . | . | . | . | . | . |
| GP |  |  |  |  |  |  |
| SP |  |  |  |  |  |  |
| Nurse |  |  |  |  |  |  |
| Other | . | . | . | . | . | . |
| Initial | 2.7213 | 1.8164 | 27.7327 | 0.1454 | -1.001 | 6.4436 |
| Follow-up | 0.2852 | 1.5729 | 19.5246 | 0.858 | -3.001 | 3.5713 |
| Not reported | . | . | . | . | . | . |
| Telemedicine | -1.4365 | 2.3283 | 24.1443 | 0.543 | -6.2403 | 3.3673 |
| In-person | . | . | . | . | . | . |
| Covid Y |  |  |  |  |  |  |
| No covid | . | . | . | . | . | . |
| Constant | 9.6373 | 2.8723 | 10.2021 | 0.0071 | 3.2547 | 16.02 |
| tau-squared | 16 | - | - | - | - | - |
| n level 1 | 674 |  |  |  |  |  |
| n level 2 | 168 |  |  |  |  |  |

|  | **SDP – model 2** |  |  |  |  |  |
| --- | --- | --- | --- | --- | --- | --- |
|  | Coef | Std Error | dfs | p-value | 95%CI L | 95%CI U |
| HIC, UMIC | 4.5708 | 1.8826 | 28.1927 | 0.0218 | 0.7156 | 8.426 |
| LMIC, LIC | . | . | . | . | . | . |
| Community | -7.2467 | 3.5815 | 9.8804 | 0.0709 | -15.24 | 0.7465 |
| GOP | -2.3826 | 3.3849 | 11.4605 | 0.4956 | -9.7964 | 5.0312 |
| 1st & 2nd referral | -1.4415 | 2.8464 | 10.2807 | 0.6232 | -7.7604 | 4.8774 |
| Other | . | . | . | . | . | . |
| GP |  |  |  |  |  |  |
| SP |  |  |  |  |  |  |
| Nurse |  |  |  |  |  |  |
| Other | . | . | . | . | . | . |
| Initial | 2.9351 | 1.8492 | 27.9426 | 0.1237 | -0.8533 | 6.7234 |
| Follow-up | 0.6987 | 1.55 | 20.3136 | 0.6569 | -2.5314 | 3.9288 |
| Not reported | . | . | . | . | . | . |
| Telemedicine | -2.3952 | 2.5472 | 19.6166 | 0.3585 | -7.7152 | 2.9248 |
| In-person | . | . | . | . | . | . |
| Covid Y | -2.9881 | 2.3099 | 27.6945 | 0.2065 | -7.722 | 1.7459 |
| No covid | . | . | . | . | . | . |
| Constant | 12.8204 | 3.6708 | 14.5043 | 0.0034 | 4.9729 | 20.6679 |
| tau-squared | 16 | - | - | - | - | - |
| n level 1 | 669 |  |  |  |  |  |
| n level 2 | 167 |  |  |  |  |  |

|  | **SDP model 3** |  |  |  |  |  |
| --- | --- | --- | --- | --- | --- | --- |
|  | Coef | Std Error | dfs | p-value | 95%CI L | 95%CI U |
| HIC, UMIC | 8.42 | 2.1237 | 32.5522 | 0.0004 | 4.10 | 12.74 |
| LMIC, LIC | . | . | . | . | . | . |
| Community | -9.76 | 5.1297 | 14.0797 | 0.0777 | -20.76 | 1.24 |
| GOP | -7.90 | 4.004 | 14.9989 | 0.0673 | -16.43 | 0.64 |
| 1st & 2nd referral | -3.17 | 3.755 | 13.2276 | 0.4134 | -11.27 | 4.93 |
| Other | . | . | . | . | . | . |
| GP |  |  |  |  |  |  |
| SP |  |  |  |  |  |  |
| Nurse |  |  |  |  |  |  |
| Other | . | . | . | . | . | . |
| Initial |  |  |  |  |  |  |
| Follow-up |  |  |  |  |  |  |
| Not reported | . | . | . | . | . | . |
| Telemedicine | -6.05 | 2.623 | 29.1563 | 0.0284 | -11.41 | -0.69 |
| In-person | . | . | . | . | . | . |
| Covid Y | -1.45 | 2.5709 | 28.7843 | 0.5770 | -6.71 | 3.81 |
| No covid | . | . | . | . | . | . |
| Constant | 16.10 | 4.6226 | 20.8101 | 0.0022 | 6.48 | 25.72 |
| tau-squared | 180 | - | - | - | - | - |
| n level 1 | 674.00 |  |  |  |  |  |
| n level 2 | 169.00 |  |  |  |  |  |

|  | **SDP - model 4** |  |  |  |  |  |
| --- | --- | --- | --- | --- | --- | --- |
|  | Coef | Std Error | dfs | p-value | 95%CI L | 95%CI U |
| HIC, UMIC | 4.6348 | 1.5041 | 28.2701 | 0.0046 | 1.5552 | 7.7145 |
| LMIC, LIC | . | . | . | . | . | . |
| Community | -6.2758 | 3.5205 | 8.8378 | 0.1089 | -14.262 | 1.7104 |
| GOP | -1.4077 | 2.6661 | 7.8627 | 0.6121 | -7.5745 | 4.7592 |
| 1st & 2nd referral | -0.2962 | 2.647 | 7.8388 | 0.9137 | -6.4221 | 5.8296 |
| Other | . | . | . | . | . | . |
| GP |  |  |  |  |  |  |
| SP |  |  |  |  |  |  |
| Nurse |  |  |  |  |  |  |
| Other | . | . | . | . | . | . |
| Initial | 2.7589 | 1.7592 | 28.8059 | 0.1277 | -0.8401 | 6.358 |
| Follow-up | -0.1188 | 1.6547 | 16.8854 | 0.9436 | -3.6117 | 3.3742 |
| Not reported | . | . | . | . | . | . |
| Telemedicine |  |  |  |  |  |  |
| In-person | . | . | . | . | . | . |
| Covid Y |  |  |  |  |  |  |
| No covid | . | . | . | . | . | . |
| Constant | 8.7088 | 2.8475 | 8.6764 | 0.0142 | 2.2305 | 15.1872 |
| tau-squared | 16 | - | - | - | - | - |
| n level 1 | 693 |  |  |  |  |  |
| n level 2 | 169 |  |  |  |  |  |

|  | **Health worker - model 1** |  |  |  |  |  |
| --- | --- | --- | --- | --- | --- | --- |
|  | Coef | Std Error | dfs | p-value | 95%CI L | 95%CI U |
| HIC, UMIC | 6.0036 | 1.8042 | 29.2321 | 0.0024 | 2.3149 | 9.6923 |
| LMIC, LIC | . | . | . | . | . | . |
| Community |  |  |  |  |  |  |
| GOP |  |  |  |  |  |  |
| 1st & 2nd referral |  |  |  |  |  |  |
| Other | . | . | . | . | . | . |
| GP | -2.6827 | 2.4047 | 36.9078 | 0.2718 | -7.5555 | 2.1902 |
| SP | -0.1662 | 2.2082 | 31.5333 | 0.9405 | -4.6667 | 4.3343 |
| Nurse | 3.3618 | 3.7316 | 13.3583 | 0.3836 | -4.6778 | 11.4015 |
| Other | . | . | . | . | . | . |
| Initial | 2.8391 | 1.9543 | 30.5077 | 0.1565 | -1.1494 | 6.8276 |
| Follow-up | 0.4057 | 1.5663 | 20.8164 | 0.7982 | -2.8533 | 3.6648 |
| Not reported | . | . | . | . | . | . |
| Telemedicine | -1.6466 | 1.8773 | 40.1145 | 0.3856 | -5.4404 | 2.1471 |
| In-person | . | . | . | . | . | . |
| Covid Y |  |  |  |  |  |  |
| No covid | . | . | . | . | . | . |
| Constant | 8.0316 | 2.206 | 15.6054 | 0.0023 | 3.3455 | 12.7176 |
| tau-squared | 23 | - | - | - | - | - |
| n level 1 | 671 |  |  |  |  |  |
| n level 2 | 166 |  |  |  |  |  |

|  | **Health worker – model 2** |  |  |  |  |  |
| --- | --- | --- | --- | --- | --- | --- |
|  | Coef | Std Error | dfs | p-value | 95%CI L | 95%CI U |
| HIC, UMIC | 5.81 | 1.8218 | 30.4289 | 0.0033 | 2.09 | 9.53 |
| LMIC, LIC | . | . | . | . | . | . |
| Community |  |  |  |  |  |  |
| GOP |  |  |  |  |  |  |
| 1st & 2nd referral |  |  |  |  |  |  |
| Other | . | . | . | . | . | . |
| GP | -2.88 | 2.3819 | 37.4167 | 0.2336 | -7.71 | 1.94 |
| SP | -0.60 | 2.1984 | 32.9761 | 0.7883 | -5.07 | 3.88 |
| Nurse | 2.91 | 3.8106 | 13.664 | 0.4579 | -5.28 | 11.10 |
| Other |  |  |  |  |  |  |
| Initial | 3.03 | 1.976 | 30.9936 | 0.1358 | -1.00 | 7.06 |
| Follow-up | 0.83 | 1.5322 | 22.0976 | 0.5914 | -2.34 | 4.01 |
| Not reported | . | . | . | . | . | . |
| Telemedicine | -2.72 | 2.0831 | 28.2511 | 0.2022 | -6.99 | 1.55 |
| In-person | . | . | . | . | . | . |
| Covid Y | -3.11 | 2.4061 | 29.232 | 0.2064 | -8.03 | 1.81 |
| No covid | . | . | . | . | . | . |
| Constant | 11.40 | 3.1437 | 33.1653 | 0.0010 | 5.00 | 17.79 |
| tau-squared | 24 | - | - | - | - | - |
| n level 1 | 666.00 |  |  |  |  |  |
| n level 2 | 165.00 |  |  |  |  |  |

|  | **Health worker – model 3** |  |  |  |  |  |
| --- | --- | --- | --- | --- | --- | --- |
|  | Coef | Std Error | dfs | p-value | 95%CI L | 95%CI U |
| HIC, UMIC | 10.8932 | 2.1174 | 32.861 | 0 | 6.5845 | 15.2018 |
| LMIC, LIC | . | . | . | . | . | . |
| Community |  |  |  |  |  |  |
| GOP |  |  |  |  |  |  |
| 1st & 2nd referral |  |  |  |  |  |  |
| Other | . | . | . | . | . | . |
| GP | -10.71 | 3.0135 | 55.0554 | 0.0008 | -16.7495 | -4.6712 |
| SP | -3.9907 | 2.9582 | 50.8312 | 0.1833 | -9.93 | 1.9487 |
| Nurse | -1.3687 | 4.6344 | 24.6487 | 0.7702 | -10.9202 | 8.1828 |
| Other | . | . | . | . | . | . |
| Initial |  |  |  |  |  |  |
| Follow-up |  |  |  |  |  |  |
| Not reported | . | . | . | . | . | . |
| Telemedicine | -6.5323 | 2.25 | 35.1537 | 0.0063 | -11.0993 | -1.9652 |
| In-person | . | . | . | . | . | . |
| Covid Y | -1.8601 | 2.679 | 27.8329 | 0.4932 | -7.3493 | 3.6291 |
| No covid | . | . | . | . | . | . |
| Constant | 15.6507 | 3.9677 | 38.1723 | 0.0003 | 7.6196 | 23.6817 |
| tau-squared | 450 | - | - | - | - | - |
| n level 1 | 671 |  |  |  |  |  |
| n level 2 | 167 |  |  |  |  |  |

|  | **Heath worker – model 4** |  |  |  |  |  |
| --- | --- | --- | --- | --- | --- | --- |
|  | Coef | Std Error | dfs | p-value | 95%CI L | 95%CI U |
| HIC, UMIC | 5.6055 | 1.4931 | 28.0873 | 0.0008 | 2.5475 | 8.6635 |
| LMIC, LIC | . | . | . | . | . | . |
| Community |  |  |  |  |  |  |
| GOP |  |  |  |  |  |  |
| 1st & 2nd referral |  |  |  |  |  |  |
| Other | . | . | . | . | . | . |
| GP | -2.1997 | 2.3137 | 34.2831 | 0.3484 | -6.9002 | 2.5009 |
| SP | -0.0905 | 2.1687 | 31.308 | 0.967 | -4.5119 | 4.3309 |
| Nurse | 3.8369 | 3.6779 | 12.9507 | 0.3159 | -4.1117 | 11.7855 |
| Other | . | . | . | . | . | . |
| Initial | 2.944 | 1.926 | 30.632 | 0.1366 | -0.986 | 6.8739 |
| Follow-up | 0.0543 | 1.6467 | 18.7454 | 0.974 | -3.3955 | 3.5042 |
| Not reported | . | . | . | . | . | . |
| Telemedicine |  |  |  |  |  |  |
| In-person | . | . | . | . | . | . |
| Covid Y |  |  |  |  |  |  |
| No covid | . | . | . | . | . | . |
| Constant | 7.8244 | 2.1431 | 15.5634 | 0.0022 | 3.2708 | 12.378 |
| tau-squared | 24 | - | - | - | - | - |
| n level 1 | 671 |  |  |  |  |  |
| n level 2 | 166 |  |  |  |  |  |

|  | 2 | 3 | 4 | 5 | 6 | 7 |
| --- | --- | --- | --- | --- | --- | --- |
|  | **Doctor only - model 1** |  |  |  |  |  |
|  | Coef | Std Error | dfs | p-value | 95%CI L | 95%CI U |
| HIC, UMIC | 6.6733 | 1.5873 | 17.5714 | 0.0006 | 3.3327 | 10.0138 |
| LMIC, LIC | . | . | . | . | . | . |
| GOP | -3.7156 | 1.7352 | 48.1888 | 0.0373 | -7.204 | -0.2271 |
| 1st & 2nd referral | . | . | . | . | . | . |
| Initial | 1.5435 | 2.2052 | 24.5791 | 0.4905 | -3.0021 | 6.0892 |
| Follow-up | -1.7328 | 1.6976 | 21.3839 | 0.3188 | -5.2594 | 1.7937 |
| Other | . | . | . | . | . | . |
| Telemedicine | -1.9032 | 1.9849 | 23.445 | 0.3474 | -6.005 | 2.1986 |
| In-person | . | . | . | . | . | . |
| Covid Y |  |  |  |  |  |  |
| No covid | . | . | . | . | . | . |
| Constant | 8.8495 | 0.9859 | 10.3274 | 0 | 6.6622 | 11.0368 |
| tau-squared | 28.8361 | - | - | - | - | - |
| n level 1 | 567 |  |  |  |  |  |
| n level 2 | 134 |  |  |  |  |  |

|  | **Doctor only – model 2** |  |  |  |  |  |
| --- | --- | --- | --- | --- | --- | --- |
|  | Coef | Std Error | dfs | p-value | 95%CI L | 95%CI U |
| HIC, UMIC | 6.0856 | 1.5888 | 18.4696 | 0.0012 | 2.7537 | 9.4176 |
| LMIC, LIC | . | . | . | . | . | . |
| GOP | -3.2163 | 1.753 | 45.2677 | 0.0731 | -6.7464 | 0.3137 |
| 1st & 2nd referral | . | . | . | . | . | . |
| Initial | 1.8449 | 2.2036 | 24.9029 | 0.4104 | -2.6945 | 6.3843 |
| Follow-up | -0.6577 | 1.7027 | 24.066 | 0.7027 | -4.1714 | 2.8559 |
| Other | . | . | . | . | . | . |
| Telemedicine | -4.2273 | 2.1014 | 16.4766 | 0.0609 | -8.6717 | 0.2171 |
| In-person | . | . | . | . | . | . |
| Covid Y | -4.5812 | 2.9899 | 19.8319 | 0.1413 | -10.8215 | 1.659 |
| No covid | . | . | . | . | . | . |
| Constant | 13.3431 | 3.0988 | 26.2998 | 0.0002 | 6.977 | 19.7093 |
| tau-squared | 28.0744 | - | - | - | - | - |
| n level 1 | 562 |  |  |  |  |  |
| n level 2 | 133 |  |  |  |  |  |

|  | **Doctor only model 3** |  |  |  |  |  |
| --- | --- | --- | --- | --- | --- | --- |
|  | Coef | Std Error | dfs | p-value | 95%CI L | 95%CI U |
| HIC, UMIC | 9.127 | 2.2392 | 20.6457 | 0.0006 | 4.4655 | 13.7885 |
| LMIC, LIC | . | . | . | . | . | . |
| GOP | -7.1412 | 1.7978 | 91.2214 | 0.0001 | -10.7122 | -3.5702 |
| 1st & 2nd referral | . | . | . | . | . | . |
| Initial |  |  |  |  |  |  |
| Follow-up |  |  |  |  |  |  |
| Other | . | . | . | . | . | . |
| Telemedicine | -6.694 | 3.1384 | 18.9744 | 0.0462 | -13.2633 | -0.1247 |
| In-person | . | . | . | . | . | . |
| Covid Y | -3.1153 | 3.4996 | 17.4883 | 0.3854 | -10.4832 | 4.2525 |
| No covid | . | . | . | . | . | . |
| Constant | 15.3261 | 3.9355 | 29.3632 | 0.0005 | 7.2814 | 23.3707 |
| tau-squared | 817.7207 | - | - | - | - | - |
| n level 1 | 563 |  |  |  |  |  |
| n level 2 | 134 |  |  |  |  |  |

|  | **Doctor only – model 4** |  |  |  |  |  |
| --- | --- | --- | --- | --- | --- | --- |
|  | Coef | Std Error | dfs | p-value | 95%CI L | 95%CI U |
| HIC, UMIC | 9.4698 | 2.19 | 20.0177 | 0.0003 | 4.9019 | 14.0378 |
| LMIC, LIC | . | . | . | . | . | . |
| GOP | -7.3888 | 1.7497 | 96.917 | 0.0001 | -10.8615 | -3.9162 |
| 1st & 2nd referral | . | . | . | . | . | . |
| Initial |  |  |  |  |  |  |
| Follow-up |  |  |  |  |  |  |
| Other | . | . | . | . | . | . |
| Telemedicine | -5.0282 | 2.2992 | 28.6198 | 0.0371 | -9.7333 | -0.3231 |
| In-person | . | . | . | . | . | . |
| Covid Y |  |  |  |  |  |  |
| No covid | . | . | . | . | . | . |
| Constant | 12.1664 | 1.7071 | 13.9221 | 0 | 8.5032 | 15.8296 |
| tau-squared | 805.6449 | - | - | - | - | - |
| n level 1 | 568 |  |  |  |  |  |
| n level 2 | 135 |  |  |  |  |  |

|  | **Doctor only – model 5** |  |  |  |  |  |
| --- | --- | --- | --- | --- | --- | --- |
|  | Coef | Std Error | dfs | p-value | 95%CI L | 95%CI U |
| HIC, UMIC | 6.2745 | 1.4708 | 17.0258 | 0.0005 | 3.1718 | 9.3773 |
| LMIC, LIC | . | . | . | . | . | . |
| GOP | -3.3183 | 1.6275 | 54.152 | 0.0464 | -6.5809 | -0.0556 |
| 1st & 2nd referral | . | . | . | . | . | . |
| Initial | 1.6781 | 2.1341 | 24.6185 | 0.4392 | -2.7207 | 6.0769 |
| Follow-up | -2.2472 | 1.6838 | 16.8686 | 0.1997 | -5.8019 | 1.3075 |
| Other | . | . | . | . | . | . |
| Telemedicine |  |  |  |  |  |  |
| In-person | . | . | . | . | . | . |
| Covid Y |  |  |  |  |  |  |
| No covid | . | . | . | . | . | . |
| Constant | 8.7321 | 0.9637 | 10.1426 | 0 | 6.5889 | 10.8753 |
| tau-squared | 28.0959 | - | - | - | - | - |
| n level 1 | 567 |  |  |  |  |  |
| n level 2 | 134 |  |  |  |  |  |

## A9 Doctor vs. nurse table mean visit time

|  | HIC, UMIC | LMIC, LIC |
| --- | --- | --- |
| GP | 14.33 minutes | 8.52 minutes |
| SP | 16.61 minutes | 10.80 minutes |
| Nurse | 20.12 minutes | 14.31 minutes |

Abbreviations: GP, general practitioner; SP, specialist physician; HIC, high income country; LIC, low income country; LMIC, lower middle income country; UMIC, upper middle income country

# REFERENCES

1. Kruk ME, Gage AD, Arsenault C, Jordan K, Leslie HH, Roder-DeWan S, et al. High-quality health systems in the Sustainable Development Goals era: time for a revolution. The Lancet global health. 2018;6(11):e1196-e252.

2. Abdu-Aguye SN, Shehu A, Ahmad UI. Management of musculoskeletal pain in retail drug outlets within a Nigerian community: a descriptive study. Pharmacy Practice (Granada). 2017;15(1).

3. Abdulkader RS, Venugopal D, Jeyashree K, Al Zayer Z, Senthamarai Kannan K, Jebitha R. The intricate relationship between client perceptions of physician empathy and physician self-assessment: lessons for reforming clinical practice. Journal of Patient Experience. 2022;9:23743735221077537.

4. Abdus-Salam R, Adeniyi A, Bello F. Antenatal Clinic Waiting Time, Patient Satisfaction, and Preference for Staggered Appointment–A Cross-Sectional Study. Journal of Patient Experience. 2021;8:23743735211060802.

5. Abner S, Gillies CL, Shabnam S, Zaccardi F, Seidu S, Davies MJ, et al. Consultation rates in people with type 2 diabetes with and without vascular complications: a retrospective analysis of 141,328 adults in England. Cardiovascular Diabetology. 2022;21:1-11.

6. Aboueid S, Bourgeault I, Giroux I. Nutrition and obesity care in multidisciplinary primary care settings in Ontario, Canada: short duration of visits and complex health problems perceived as barriers. Preventive medicine reports. 2018;10:242-7.

7. Abushaala A, Sargent H, McLean J, Grech-Marguerat D, Khalil H. A new approach to managing follow-up patients with sino-nasal disorders: the rhinology panel. British Journal of Healthcare Management. 2020;26(3):88-93.

8. Ah-Kye L, Butt A, Gupta A, Timlin H, Daniel C, Uddin J, et al. Introducing the ‘Benign Eyelid Lesion Pathway’: 1 year experience of synchronous tele-oculoplastics in a tertiary hospital. Eye. 2023;37(7):1458-63.

9. Ahmad B, Khairatul K, Farnaza A. An assessment of patient waiting and consultation time in a primary healthcare clinic. Malaysian family physician: the official journal of the Academy of Family Physicians of Malaysia. 2017;12(1):14.

10. Alarcon-Ruiz CA, Heredia P, Taype-Rondan A. Association of waiting and consultation time with patient satisfaction: secondary-data analysis of a national survey in Peruvian ambulatory care facilities. BMC health services research. 2019;19:1-9.

11. Al-Habbal K, Djoundourian A, Nassar E, Tayara Z, Mercer SW, Abi-Habib R. Reliability and validity of the Arabic version of the Consultation and Relational Empathy (CARE) measure. Family Practice. 2022;39(6):1176-82.

12. Al-Harajin RS, Al-Subaie SA, Elzubair AG. The association between waiting time and patient satisfaction in outpatient clinics: Findings from a tertiary care hospital in Saudi Arabia. Journal of family & community medicine. 2019;26(1):17.

13. Aljehani NF, Kayal S, Albeshry AM, Qutub MM. Resilience and Coping Self-Efficacy among Family Medicine Residents in times of COVID-19 pandemic: Interplay and contribution of sociodemographic, professional, lifestyle and health-related factors. Middle East Journal of Family Medicine. 2022;7(10):39.

14. Allen S, Harris R, Brown SL, Humphris G, Zhou Y, Rogers SN. High levels of socioeconomic deprivation do not inhibit patients’ communication of concerns in head and neck cancer review clinics. British Journal of Oral and Maxillofacial Surgery. 2018;56(6):536-9.

15. Alsubeeh NA, AlSaqr MA, Alkarzae M, Aldosari B. Prevalence of considering revision rhinoplasty in Saudi patients and its associated factors. Maxillofacial Plastic and Reconstructive Surgery. 2019;41:1-10.

16. Alzayer ZM, Abdulkader RS, Jeyashree K, Alselihem A. Patient-rated physicians' empathy and its determinants in Riyadh, Saudi Arabia. Journal of Family & Community Medicine. 2019;26(3):199.

17. Andreyeva E, David G, Song H. The effects of home health visit length on hospital readmission. National Bureau of Economic Research; 2018.

18. Ann-Yi S, Bruera E, Wu J, Liu DD, Agosta M, Williams JL, et al. Characteristics and outcomes of psychology referrals in a palliative care department. Journal of pain and symptom management. 2018;56(3):344-51.

19. Appiah J, Barlow L, Mmonu NA, Makarov DV, Sugarman A, Matulewicz RS. A National Assessment of the Association Between Patient Race and Physician Visit Time During New Outpatient Urology Consultations. Urology. 2022;162:63-9.

20. Ariyo O, Kinney O, Brookhart A, Nadpara P. Medication therapy problems and vaccine needs identified during initial appointment-based medication synchronization visits. Journal of the American Pharmacists Association. 2019;59(4):S67-S71.

21. Asamani JA, Christmals CD, Reitsma GM, editors. Health service activity standards and standard workloads for primary healthcare in Ghana: A cross-sectional survey of health professionals. Healthcare; 2021: MDPI.

22. Asan O, Tyszka J, Crotty B. The electronic health record as a patient engagement tool: mirroring clinicians’ screen to create a shared mental model. Jamia Open. 2018;1(1):42-8.

23. Assefa T, Abera B, Bacha T, Beedemariam G. Prescription completeness and drug use pattern in the University Teaching Hospital, Addis Ababa, Ethiopia. J Basic Clin Pharm. 2018;9(4).

24. Atanda Jr A, Pelton M, Fabricant PD, Tucker A, Shah SA, Slamon N. Telemedicine utilisation in a paediatric sports medicine practice: decreased cost and wait times with increased satisfaction. Journal of ISAKOS. 2018;3(2):94-7.

25. Atia A. Information provided to customers about over-the-counter medications dispensed in community pharmacies in Libya: A crosssectional study. Eastern Mediterranean Health Journal. 2020;26(7):828-33.

26. Ayalew Getahun K, Sitotie Redia A, Jemere Aragaw T. Evaluation of medicine-use pattern using World Health Organization’s core drug-use indicators and completeness of prescription at University of Gondar Comprehensive Specialized Hospital, Gondar, Ethiopia: cross-sectional study. Integrated Pharmacy Research and Practice. 2020:219-27.

27. Ayele AA, Mekuria AB, Tegegn HG, Gebresillassie BM, Mekonnen AB, Erku DA. Management of minor ailments in a community pharmacy setting: Findings from simulated visits and qualitative study in Gondar town, Ethiopia. PloS one. 2018;13(1):e0190583.

28. Barratt J, Thomas N. Nurse practitioner consultations in primary health care: a case study-based survey of patients’ pre-consultation expectations, and post-consultation satisfaction and enablement. Primary health care research & development. 2019;20:e36.

29. Bauer Z, Sherwin J, Smith S, Radowsky J. A Review of Brooke Army Medical Center Chaplaincy Service During the SARS-COV2 Pandemic: Implications for Service Structure and Patient Needs. Military Medicine. 2023;188(3-4):e824-e8.

30. Beach MC, Park J, Han D, Evans C, Moore RD, Saha S. Clinician response to patient emotion: impact on subsequent communication and visit length. The Annals of Family Medicine. 2021;19(6):515-20.

31. Bener A, Alayoglu N, Çatan F, Torun P, Yilmaz ES. Health services management in Turkey: Failure or success? International journal of preventive medicine. 2019;10.

32. Benski AC, Schmidt NC, Viviano M, Stancanelli G, Soaroby A, Reich MR. Improving the quality of antenatal care using mobile health in Madagascar: five-year cross-sectional study. JMIR mHealth and uHealth. 2020;8(7):e18543.

33. Benski AC, Stancanelli G, Scaringella S, Herinainasolo JL, Jinoro J, Vassilakos P, et al. Usability and feasibility of a mobile health system to provide comprehensive antenatal care in low-income countries: PANDA mHealth pilot study in Madagascar. Journal of telemedicine and telecare. 2017;23(5):536-43.

34. Berasa MS. ASSESSMENT OF DRUG USE PATTERN BY USING WHO PATIENT CARE AND HEALTH FACILITY INDICATORS IN FIVE PUBLIC HOSPITALS IN SOUTHERN ETHIOPIA. International Journal of Innovative Pharmaceutical Sciences and Research. 2017;5(10):18-26.

35. Berk SI. Time to care: primary care visit duration and value-based healthcare. The American Journal of Medicine. 2020;133(6):655-6.

36. Berkowitz RL, Bui L, Shen Z, Pressman A, Moreno M, Brown S, et al. Evaluation of a social determinants of health screening questionnaire and workflow pilot within an adult ambulatory clinic. BMC Family Practice. 2021;22(1):1-14.

37. Binyaruka P, Borghi J. An equity analysis on the household costs of accessing and utilising maternal and child health care services in Tanzania. Health economics review. 2022;12(1):36.

38. Birhanu B, Siraneh Y, Gelana B, Tsega G. Quality of family planning services and associated factors in Jimma Town public hospitals, Southwest Ethiopia. Ethiopian journal of health sciences. 2019;29(5).

39. Bissessor M, Bradshaw CS, Fairley CK, Chen MY, Chow EP. Provision of HIV test results by telephone is both safe and efficient for men who have sex with men. International journal of STD & AIDS. 2017;28(1):39-44.

40. Black A, Courtenay M, Norton C, Dean Franklin B, Murrells T, Gage H. Independent nurse medication provision: A mixed method study assessing impact on patients' experience, processes, and costs in sexual health clinics. Journal of advanced nursing. 2022;78(1):239-51.

41. Bock MJ, Kakavand K, Careaga D, Gozalians S. Shifting from in-person to virtual home visiting in Los Angeles County: Impact on programmatic outcomes. Maternal and Child Health Journal. 2021;25:1025-30.

42. Boffa J, Moyo S, Chikovore J, Salomon A, Daniels B, Kwan AT, et al. Quality of care for tuberculosis and HIV in the private health sector: a cross-sectional, standardised patient study in South Africa. BMJ global health. 2021;6(5):e005250.

43. Bolívar FJV, González MP, Martos HM, García IC, Durántez JT. Communication with patients and the duration of family medicine consultations. Atención Primaria. 2018;50(10):621-8.

44. Bon V, Ghemame M, Fantou P, Philliponnet A, Mouriaux F. Feedback on ophthalmologic telemedicine in a nursing home. Journal francais d'ophtalmologie. 2020;43(9):e293-e7.

45. Bonney A, Morgan S, Tapley A, Henderson K, Holliday E, Davey A, et al. Older patients' consultations in an apprenticeship model‐based general practice training program: A cross‐sectional study. Australasian journal on ageing. 2017;36(1):E1-E7.

46. Bowen S, Gheewala R, Paez W, Lucke-Wold B, Mitin T, Ciporen J. Telemedicine visits in an established multidisciplinary central nervous system clinic for radiation oncology and neurosurgery (RADIANS) in a community hospital setting. Bratislavske lekarske listy. 2021;122(9):680.

47. Brenner AT, Malo TL, Margolis M, Lafata JE, James S, Vu MB, Reuland DS. Evaluating shared decision making for lung cancer screening. JAMA internal medicine. 2018;178(10):1311-6.

48. Breyer M, Farley H, Neyman G, Nichols W, Kendall J. Emergency physicians who produce higher relative value units per hour spend similar amounts of time at patient bedsides as their colleagues. The Journal of Emergency Medicine. 2017;53(5):765-70.

49. Burger BB, Veerman MM, Tellier MA, Leclercq WK, Mouës-Vink CM, Werker PM. Insight in Information Provision Prior to Obtaining Surgical Informed Consent—by Audiotaping Outpatient Consultations. World Journal of Surgery. 2019;43:425-30.

50. Burnett-Zieman B, Abuya T, Mwanga D, Wanyugu J, Warren CE, Sripad P. Community-based postnatal care services for women and newborns in Kenya: an opportunity to improve quality and access? Journal of Global Health. 2021;11.

51. Cabrera-Rivadeneyra ZE, Sánchez-Vélez MA. Factors associated with the external consultation package in the physical therapy and rehabilitation service of the Lambayeque Regional Hospital.

52. Calvitti A, Hochheiser H, Ashfaq S, Bell K, Chen Y, El Kareh R, et al. Physician activity during outpatient visits and subjective workload. Journal of biomedical informatics. 2017;69:135-49.

53. Castner J, Suffoletto H. Emergency department crowding and time at the bedside: a wearable technology feasibility study. Journal of Emergency Nursing. 2018;44(6):624-31. e2.

54. Chao AH, Yaney A, Skoracki RJ, Kearns PN. The impact of physician assistants on a breast reconstruction practice: outcomes and cost analysis. Annals of Plastic Surgery. 2017;79(3):249-52.

55. Chebolu-Subramanian V, Sule N, Sharma R, Mistry N. A time motion study of community mental health workers in rural India. BMC health services research. 2019;19:1-7.

56. Cheung KL, Smoger S, Tamura MK, Stapleton RD, Rabinowitz T, LaMantia MA, Gramling R. Content of Tele-Palliative Care Consultations with Patients Receiving Dialysis. Journal of Palliative Medicine. 2022;25(8):1208-14.

57. Chingombe I, Mapingure MP, Balachandra S, Chipango TN, Gambanga F, Mushavi A, et al. Patient costs for prevention of mother-to-child HIV transmission and antiretroviral therapy services in public health facilities in Zimbabwe. Plos one. 2021;16(8):e0256291.

58. Cho J, Sanchez K, Ganor O, Afshar S, Ruditsky A, Bierman A, Taghinia AH. Utilizing a Physician Scribe in a Pediatric Plastic Surgical Practice: A Time-driven Activity-based Costing Study. Plast Reconstr Surg Glob Open. 2019 Oct;7(10):e2460. PubMed PMID: 31772889. PMCID: PMC6846305. Epub 20191021. eng.

59. Ciocănel A, Rughiniș C, Flaherty MG. Argumentative time work for legitimizing homeopathy: Temporal reasons for the acceptance of an alternative medical practice. Time & Society. 2021;30(1):100-25.

60. Compère V, Froemer B, Clavier T, Selim J, Burey J, Dureuil B, et al. Evaluation of the duration of preanesthesia consultation: prospective and multicenter study. Anesthesia & Analgesia. 2022;134(3):496-504.

61. Conley K, Chambers C, Elnahal S, Choflet A, Williams K, DeWeese T, et al. Using a real-time location system to measure patient flow in a radiation oncology outpatient clinic. Practical radiation oncology. 2018;8(5):317-23.

62. Corn CE, Klepser DG, Dering-Anderson AM, Brown TG, Klepser ME, Smith JK. Observation of a pharmacist-conducted group A streptococcal pharyngitis point-of-care test: a time and motion study. Journal of Pharmacy Practice. 2018;31(3):284-91.

63. Crocker-Buque T, Edelstein M, Mounier-Jack S. A process evaluation of how the routine vaccination programme is implemented at GP practices in England. Implementation science. 2018;13(1):1-19.

64. Crosbie B, O’Callaghan ME, O’Flanagan S, Brennan D, Keane G, Behan W. A real-time measurement of general practice workload in the Republic of Ireland: a prospective study. British Journal of General Practice. 2020;70(696):e489-e96.

65. Cui F, He X, Zhai Y, Lyu M, Shi J, Sun D, et al. Application of telemedicine services based on a regional telemedicine platform in China from 2014 to 2020: longitudinal trend analysis. Journal of medical Internet research. 2021;23(7):e28009.

66. Dabaghzadeh F, Hajjari R. Practice of community pharmacists related to multivitamin supplements: a simulated patient study in Iran. International Journal of Clinical Pharmacy. 2018;40:190-5.

67. Dakroub M, Halawy M, Bashshur Z, Ghannam AB, Noureddine B, Al-Haddad C. A comparison of the workload between pediatric and adult ophthalmology encounters. Journal of American Association for Pediatric Ophthalmology and Strabismus. 2021;25(3):160. e1-. e5.

68. Daniel SR, McDermott Jr JD, Le C, Pierce CA, Ziskind MA, Ellis LA. A real-world, multi-site, observational study of infusion time and treatment satisfaction with rheumatoid arthritis patients treated with intravenous golimumab or infliximab. Journal of Medical Economics. 2018;21(7):724-31.

69. Darlison Shepherd PR, Krejany CJ, Jiwa M. How does the duration of consults vary for upper respiratory tract infections in general practice where an antibiotic has been prescribed? Family Practice. 2020;37(2):213-8.

70. Das MC, Zakaria M, Cheng F, Xu J, editors. Appointment length with patients in medical consultations in Bangladesh: A hospital-based cross-sectional study. Healthcare; 2021: MDPI.

71. Dessie B, Atalaye G, Diress E, Getahun A. Practice towards rational drug use at Finotselam and Asirade Zewudie Hospitals based on WHO core drug use indicators, Northwest Ethiopia. The Scientific World Journal. 2020;2020.

72. DeWyer A, Scheel A, Kamarembo J, Akech R, Asiimwe A, Beaton A, et al. Establishment of a cardiac telehealth program to support cardiovascular diagnosis and care in a remote, resource-poor setting in Uganda. PLoS One. 2021;16(8):e0255918.

73. Dingwall S, Henderson J, Britt H, Harrison C. Adequacy of Australia’s GP workforce: estimating supply and demand, 2005–06 to 2015–16. Australian Health Review. 2019;44(2):328-33.

74. Donahue ML, Eberly MD, Rajnik M. Tele-TB: using TeleMedicine to increase access to directly observed therapy for latent tuberculosis infection. Military medicine. 2021;186(Supplement_1):25-31.

75. Driever EM, Stiggelbout AM, Brand PL. Shared decision-making in different types of decisions in medical specialist consultations. Journal of General Internal Medicine. 2022:1-7.

76. Dubois J, Bill A-S, Pasquier J, Keberle S, Burnand B, Rodondi P-Y. Characteristics of complementary medicine therapists in Switzerland: A cross-sectional study. PloS one. 2019;14(10):e0224098.

77. Ebbers T, Kool RB, Smeele LE, Takes RP, van den Broek GB, Dirven R. Quantifying the electronic health record burden in head and neck cancer care. Applied Clinical Informatics. 2022;13(04):857-64.

78. Ekberg K, Barr C, Hickson L. Difficult conversations: Talking about cost in audiology consultations with older adults. International Journal of Audiology. 2017;56(11):854-61.

79. Ellington L, Clayton MF, Reblin M, Donaldson G, Latimer S. Communication among cancer patients, caregivers, and hospice nurses: Content, process and change over time. Patient education and counseling. 2018;101(3):414-21.

80. Elliott M, Jones S, Johnson C, Wallace C. What are the benefits of nurse-led triage in primary care? Primary Health Care. 2020;30(3).

81. Ellis B, Melady D, Foster N, Sinha S, Lau V, Saraga S, McLeod SL. Using volunteers to improve the experience of older patients in the emergency department. Canadian Journal of Emergency Medicine. 2020;22(4):514-8.

82. Elrggal ME, Alamer SI, Alkahtani SA, Alshrahili MA, Alharbi A, Alghamdi BA, Zaitoun MF. Dispensing Practices for Weight Management Products in Eastern Saudi Arabia: A Survey of Community Pharmacists. International Journal of Environmental Research and Public Health. 2021;18(24):13146.

83. Ewelukwa O, Perez R, Carter LE, Fernandez A, Glover S. Incorporation of scribes into the inflammatory bowel disease clinic improves quality of care and physician productivity. Inflammatory Bowel Diseases. 2018;24(3):552-7.

84. Fang Y, Tung KD, Beleno-Sanchez J, Forrest JL, Roll SC. Characterization of tasks and time efficiency of dental hygiene students during clinical training. American Dental Hygienists' Association. 2020;94(5):30-7.

85. Fatigante M, Zucchermaglio C, Alby F. Being in place: a multimodal analysis of the contribution of the patient's companion to “first time” oncological visits. Frontiers in Psychology. 2021;12:664747.

86. Fesler JR, Stanton S, Merner K, Ross L, McGinley MP, Bena J, et al. Bridging the gap in epilepsy care: A single‐center experience of 3700 outpatient tele‐epilepsy visits. Epilepsia. 2020;61(8):e95-e100.

87. Fieux M, Duret S, Bawazeer N, Denoix L, Zaouche S, Tringali S. Telemedicine for ENT: Effect on quality of care during Covid-19 pandemic. European Annals of Otorhinolaryngology, Head and Neck Diseases. 2020;137(4):257-61.

88. Fisher C, Adams J, Frawley J, Hickman L, Sibbritt D. Western herbal medicine consultations for common menstrual problems; practitioner experiences and perceptions of treatment. Phytotherapy Research. 2018;32(3):531-41.

89. Folayan MO, Oyedele TA, Oziegbe E. Time expended on managing molar incisor hypomineralization in a pediatric dental clinic in Nigeria. Brazilian Oral Research. 2018;32.

90. Ensign SF, Baca-Motes K, Steinhubl SR, Topol EJ. Characteristics of the modern-day physician house call. Medicine. 2019;98(8).

91. Frost TP, Klepser DG, Small DC, Doyle IC. Time and motion study of pharmacist prescribing of oral hormonal contraceptives in Oregon community pharmacies. Journal of the American Pharmacists Association. 2019;59(2):222-7.

92. Frye WS, Cucchiaro G, Nguyen ATH, Householder K, Kuhn B. Preappointment Surveys and Reminder Calls to Improve Show Rate. American Journal of Managed Care. 2022;28(8).

93. Gaffney A, Himmelstein DU, Dickman S, McCormick D, Cai C, Woolhandler S. Trends and disparities in the distribution of outpatient physicians’ annual face time with patients, 1979–2018. Journal of General Internal Medicine. 2023;38(2):434-41.

94. Galvão TF, Tiguman GMB, Costa Filho DBd, Silva MT. Waiting time and medical consultation length in the Manaus metropolitan region, Brazil: a cross-sectional, population-based study, 2015. Epidemiologia e Serviços de Saúde. 2020;29.

95. Gan Z, Lee SY, Weiss DA, Van Batavia J, Siu S, Frazier J, et al. Single institution experience with telemedicine for pediatric urology outpatient visits: Adapting to COVID-19 restrictions, patient satisfaction, and future utilization. Journal of Pediatric Urology. 2021;17(4):480. e1-. e7.

96. Gao Q, Liu M, Peng L, Zhang Y, Shi Y, Teuwen DE, Yi H. Patient satisfaction and its health provider-related determinants in primary health facilities in rural China. BMC Health Services Research. 2022 2022/07/26;22(1):946.

97. Garcia-Layana A, García-Arumí J, Figueroa MS, Barquet LA, Ruíz-Moreno JM, Monclús-Arbona L, Group TSAM. Management of wet age-related macular degeneration in Spain: challenges for treat and extend implementation in routine clinical practice. Journal of Ophthalmology. 2019;2019.

98. Garg A, Goyal S, Thati R, Thati N. Implementation of telemedicine in a tertiary hospital–based ambulatory practice in detroit during the COVID-19 pandemic: observational study. JMIR public health and surveillance. 2021;7(1):e21327.

99. Gebramariam E, Ahmed M. Evaluation of rational medicine use based on WHO core drug use indicators in public hospitals in West Shoa Zone, Oromia, Ethiopia. Adv Pharmacoepidemiol Drug Saf. 2019;8(1):2167.

100. Geessink NH, Ofstad EH, Rikkert MGO, van Goor H, Kasper J, Schoon Y. Shared decision-making in older patients with colorectal or pancreatic cancer: Determinants of patients’ and observers’ perceptions. Patient education and counseling. 2018;101(10):1767-74.

101. Gidlow CJ, Ellis NJ, Cowap L, Riley VA, Crone D, Cottrell E, et al. Quantitative examination of video-recorded NHS Health Checks: comparison of the use of QRISK2 versus JBS3 cardiovascular risk calculators. BMJ open. 2020;10(9):e037790.

102. Glinkowski W, M. Telemedicine Orthopedic Consultations Duration and Timing in Outpatient Clinical Practice During the COVID-19 Pandemic. Telemedicine and e-Health. 2023;29(5):778-87.

103. Goldstein IH, Hribar MR, Read-Brown S, Chiang MF. Association of the presence of trainees with outpatient appointment times in an ophthalmology clinic. JAMA ophthalmology. 2018;136(1):20-6.

104. Gopfert A, Deeny SR, Fisher R, Stafford M. Primary care consultation length by deprivation and multimorbidity in England: an observational study using electronic patient records. British Journal of General Practice. 2021;71(704):e185-e92.

105. Gormley MJ, Meadows TJ, Hostermans SJ, Kettlewell PW, Kirchner HL, O'Dell SM. The relationship between integrated pediatric psychology and primary care visit length, revenue, content over 24 months. Families, Systems, & Health. 2020;38(3):278.

106. Grandizio LC, Mettler AW, Caselli ME, Pavis EJ. Telemedicine after upper extremity surgery: a prospective study of program implementation. The Journal of hand surgery. 2020;45(9):795-801.

107. Greenfield PT, Manz WJ, DeMaio EL, Duddleston SH, Xerogeanes JW, Scott Maughon T, et al. Telehealth can be implemented across a musculoskeletal service line without compromising patient satisfaction. HSS Journal®. 2021;17(1):36-45.

108. Gregório J, Cavaco AM, Lapão LV. How to best manage time interaction with patients? Community pharmacist workload and service provision analysis. Research in Social and Administrative Pharmacy. 2017;13(1):133-47.

109. Gudeta T, Mechal D. Patient knowledge of correct dosage regimen; the need for good dispensing practice. Journal of Primary Care & Community Health. 2019;10:2150132719876522.

110. Gunasekera S, Seneviratne S, Jalink M, Joseph N, Ariyarathna Y, Booth CM, Wijeratne DT. Clinical oncology workload in Sri Lanka: Infrastructure, supports, and delivery of clinical care. JCO Global Oncology. 2021;7:1703-10.

111. Hafeez H, Akbar J, Munir AB, Qamar-uz-Zaman M, Faiz S, Abbas SMM, Riaz I. Evaluation of rational drug use among pediatrics by using WHO core drug use indicators in selected hospitals of Pakistan. International Journal of Pharmaceutical Research. 2020 (1).

112. Alireza Hajizadeh, Edris Kakemam, Milad Khodavandi, Khodayari-Zarnaq R. Factors Affecting Outpatient Consultation Length among Specialists in Tabriz, Iran. Evidence Based Health Policy, Management and Economics. 2019.

113. Hallensleben J, Wöhler C. [Influence of the COVID-19 pandemic on the number and duration of care counselling provided by outpatient care services]. HeilberufeScience. 2021;12(3-4):79-91. PubMed PMID: 34745811. PMCID: PMC8561680. Epub 20211102. Einfluss der COVID-19-Pandemie auf Anzahl und Dauer der von ambulanten Pflegediensten erbrachten Pflegeberatungen nach § 37 Abs. 3 SGB XI in Bayern. ger.

114. Halloran SM, Coleman BC, Kawecki T, Long CR, Goertz C, Lisi AJ. Characteristics and Practice Patterns of U.S. Veterans Health Administration Doctors of Chiropractic: A Cross-sectional Survey. J Manipulative Physiol Ther. 2021 Sep;44(7):535-45. PubMed PMID: 35282855. PMCID: PMC8959397. eng.

115. Hallquist MLG, Tricou EP, Hallquist MN, Savatt JM, Rocha H, Evans AE, et al. Positive impact of genetic counseling assistants on genetic counseling efficiency, patient volume, and cost in a cancer genetics clinic. Genet Med. 2020 Aug;22(8):1348-54. PubMed PMID: 32350418. Epub 20200430. eng.

116. Halls S, Thomas R, Stott H, Cupples ME, Kersten P, Cramp F, et al. Provision of first contact physiotherapy in primary care across the UK: a survey of the service. Physiotherapy. 2020 Sep;108:2-9. PubMed PMID: 32693238. Epub 20200427. eng.

117. Hammad EA, Al-Aqeel S, Elayah E, Jaber D. Assessing content and factors influencing responses to information requests in community pharmacies in Jordan: A simulated patients study. PLoS One. 2022;17(2):e0264224. PubMed PMID: 35180273. PMCID: PMC8856514. Epub 20220218. eng.

118. Hammersley V, Donaghy E, Parker R, McNeilly H, Atherton H, Bikker A, et al. Comparing the content and quality of video, telephone, and face-to-face consultations: a non-randomised, quasi-experimental, exploratory study in UK primary care. Br J Gen Pract. 2019 Sep;69(686):e595-e604. PubMed PMID: 31262846. PMCID: PMC6607843. Epub 20190829. eng.

119. Hanna G, Siddiqui B, Jernigan EA, Edobor-Osula F. Telemedicine for pediatric orthopedic visits: evaluating usability and satisfaction. J Pediatr Orthop B. 2022 Jan 1;31(1):e75-e80. PubMed PMID: 34456289. eng.

120. Hepp P, Osterhoff G, Melcher P, Henkelmann R, Theopold J. Online consultation in an orthopedic trauma surgery outpatient clinic: is there a learning curve? BMC Musculoskeletal Disorders. 2022;23(1):196.

121. Hickey S, Gomez J, Meller B, Schneider JC, Cheney M, Nejad S, et al. Interactive home telehealth and burns: a pilot study. Burns. 2017;43(6):1318-21.

122. Higgins KL, Hauck FR, Tanabe K, Tingen J. Role of the ambulatory care clinical pharmacist in management of a refugee patient population at a university-based refugee healthcare clinic. Journal of Immigrant and Minority Health. 2020;22:17-21.

123. Hilder J, Stubbe M, Macdonald L, Abels P, Dowell AC. Communication in high risk ante-natal consultations: a direct observational study of interactions between patients and obstetricians. BMC Pregnancy and Childbirth. 2020;20(1):1-13.

124. Hirsch JD, San Agustin K, Barnes C, Agarwal A, Allen K, Rivera S, et al. Impact of a contactless prescription pickup kiosk on prescription abandonment, patient experience, and pharmacist consultations. Journal of the American Pharmacists Association. 2021;61(2):151-7. e1.

125. Hiscock H, Danchin MH, Efron D, Gulenc A, Hearps S, Freed GL, et al. Trends in paediatric practice in Australia: 2008 and 2013 national audits from the Australian Paediatric Research Network. Journal of Paediatrics and Child Health. 2017;53(1):55-61.

126. Höglander J, Eklund JH, Spreeuwenberg P, Eide H, Sundler AJ, Roter D, Holmström IK. Exploring patient-centered aspects of home care communication: a cross-sectional study. BMC nursing. 2020;19(1):1-10.

127. Hohman JA, Martinez KA, Anand A, Rood M, Martyn T, Rose S, Rothberg MB. Use of direct-to-consumer telemedicine to access mental health services. Journal of General Internal Medicine. 2022;37(11):2759-67.

128. Hori H, Yamato K. Assessment of current clinical practices for major depression in Japan using a web-based questionnaire. Neuropsychiatric Disease and Treatment. 2019:2821-32.

129. Hsiao B, Binder‐Finnema P, Nowell WB, Michel G, Wiedmeyer C, Fraenkel L. Preference Phenotypes in Support of Shared Decision–Making at Point‐of‐Care for Patients With Rheumatoid Arthritis: A Proof‐of‐Concept Study. Arthritis care & research. 2019;71(5):629-37.

130. Huang J, Tattersall R, Morse K, Nickerson-Troy J, Clements E, Celauro L, Lovell A. Assessment of reimbursement in a community hospital–based pharmacist-managed outpatient transition clinic. American Journal of Health-System Pharmacy. 2017;74(11_Supplement_2):S30-S4.

131. Humphries LS, Shenaq DS, Teven CM, Park JE, Song DH. SSET project: cost-effectiveness analysis of surgical specialty emergency trays in the emergency department. Plastic and Reconstructive Surgery Global Open. 2018;6(1).

132. Hunter WG, Zafar SY, Hesson A, Davis JK, Kirby C, Barnett JA, Ubel PA. Discussing health care expenses in the oncology clinic: analysis of cost conversations in outpatient encounters. Journal of oncology practice. 2017;13(11):e944-e56.

133. Hutchings OR, Dearing C, Jagers D, Shaw MJ, Raffan F, Jones A, et al. Virtual health care for community management of patients with COVID-19 in Australia: observational cohort study. Journal of medical Internet research. 2021;23(3):e21064.

134. Hwang CJ, Eftekhari K, Schwarcz RM, Massry GG. The aesthetic oculoplastic surgery video teleconference consult. Aesthetic Surgery Journal. 2019;39(7):714-8.

135. Idrees M, Ali Shah SN, Iqbal W, Bazaz SA, Bukhari F. Analysis Of Consultation Length In Khyber Pakhtunkhwa, Pakistan. Journal of Ayub Medical College Abbottabad-Pakistan. 2021;33(3).

136. Iftikhar Z, Naeem M, Farooq U. A Study on the Patients Satisfaction and Experiences from the Surgical Out Patient Department. Pakistan Journal of Medical and Health Sciences. 2021;15(4):1200-2.

137. Iwasa Y, Suzuki M, Saito I. Home Health Nursing Care Time for Patients with Parkinson’s Disease. Journal of Personalized Medicine. 2022;12(5):714.

138. Jabour AM. The impact of electronic health records on the duration of patients’ visits: Time and motion study. JMIR Medical Informatics. 2020;8(2):e16502.

139. Jácome C, Pereira AM, Amaral R, Alves-Correia M, Almeida R, Mendes S, Fonseca JA. The use of remote care during the coronavirus disease 2019 pandemic-a perspective of Portuguese and Spanish physicians. Eur Ann Allergy Clin Immunol. 2020.

140. Jahromi VK, Mehrolhassani MH, Dehnavieh R, Anari HS. Continuity of care evaluation: The view of patients and professionals about urban family physician program. International journal of preventive medicine. 2017;8.

141. James JP, Thampi SM. Time spent by patients in a pre-anaesthetic clinic and the factors affecting it: An audit from a tertiary care teaching hospital. Indian Journal of Anaesthesia. 2018;62(1):16.

142. Janati A, Hasanpoor E, Aslani F, HaghGoshayie E, Hassanzadeh E. Evaluating visit quality in plan of health sector evolution in Iran: A local survey from Tabriz. Epidemiology and Health System Journal. 2017;4(1):69-77.

143. Jandovitz N, Li H, Watts B, Monteiro J, Kohlberg D, Tsapepas D. Telemedicine pharmacy services implementation in organ transplantation at a metropolitan academic medical center. Digital Health. 2018;4:2055207618789322.

144. Jegal KH, Ko MM, Kim B-Y, Son MJ, Kim S. A national survey on current clinical practice pattern of Korean Medicine doctors for treating obesity. PLoS One. 2022;17(3):e0266034.

145. Jin G, Chen C, Liu Y, Zhao Y, Chen L, Du J, et al. Prescribing patterns of encounters in fourteen general practice clinics in rural Beijing: a cross-sectional study. BMC health services research. 2019;19:1-12.

146. Jones AH, Jacobs MB, October TW. Communication skills and practices vary by clinician type. Hospital Pediatrics. 2020;10(4):325-30.

147. Joshi SR, Wangnoo SK, Chowdhury S, Chandalia HB, Sethi B, Unnikrishnan AG, et al. Indian reality of clinical practice and patient profile in diabetes care: lessons from the IMPACT survey. J Assoc Physicians India. 2022;70:11-2.

148. Kabeya Y, Uchida J, Toyoda M, Katsuki T, Oikawa Y, Kato K, et al. Factors affecting consultation length in a Japanese diabetes practice. Diabetes research and clinical practice. 2017;126:54-9.

149. Kagedan DJ, Edge SB, Takabe K. Behind the clock: elucidating factors contributing to longer clinic appointment duration and patient wait time. BMC Health Services Research. 2021;21:1-9.

150. Kantor O, Schneider AB, Rojnica M, Benjamin AJ, Schindler N, Posner MC, et al. Implementing a resident acute care surgery service: improving resident education and patient care. Surgery. 2017;161(3):876-83.

151. Karatas M, Kartalci S, Selcuk EB, Yetis H, Karatas T, Gozukara H. Evaluation of psychiatrist’s communication with psychosis patients. Acta Bioethica. 2022;28(1).

152. Karia A, Norman R, Robinson S, Lehnbom E, Laba T-L, Durakovic I, et al. Pharmacist’s time spent: space for Pharmacy-based Interventions and Consultation TimE (SPICE)—an observational time and motion study. BMJ open. 2022;12(3):e055597.

153. Kasturi S, Price LL, LeClair A, Patel N, Shetty S, Sheira D, et al. Clinical integration of patient-reported outcome measures to enhance the care of patients with SLE: a multi-centre prospective cohort study. Rheumatology (Oxford). 2022 Nov 28;61(12):4763-74. PubMed PMID: 35357445. PMCID: PMC9707322. eng.

154. Kelly J, Garvey D, Biro MA, Lee S. Managing medical service delivery gaps in a socially disadvantaged rural community: A Nurse Practitioner led clinic. The Australian Journal of Advanced Nursing. 2017;34(4):42-9. PubMed PMID: ielapa.948044021377151. English.

155. Keyworth C, Epton T, Goldthorpe J, Calam R, Armitage CJ. Are healthcare professionals delivering opportunistic behaviour change interventions? A multi-professional survey of engagement with public health policy. Implementation Science. 2018 2018/09/21;13(1):122.

156. Khairat S, Pillai M, Edson B, Gianforcaro R. Evaluating the Telehealth Experience of Patients With COVID-19 Symptoms: Recommendations on Best Practices. J Patient Exp. 2020 Oct;7(5):665-72. PubMed PMID: 33294596. PMCID: PMC7705823. Epub 20200904. eng.

157. Kieran R, Murphy C, Maher E, Buchalter J, Sukor S, Alken S. A permanent legacy of the pandemic? Outcomes of and staff views on the introduction of virtual clinics to an Irish oncology service. Ir J Med Sci. 2023 Feb;192(1):33-40. PubMed PMID: 35098430. PMCID: PMC8801271. Epub 20220131. eng.

158. Kilduff CL, Thomas AA, Dugdill J, Casswell EJ, Dabrowski M, Lovegrove C, et al. Creating the Moorfields' virtual eye casualty: video consultations to provide emergency teleophthalmology care during and beyond the COVID-19 pandemic. BMJ Health Care Inform. 2020 Aug;27(3). PubMed PMID: 32796085. PMCID: PMC7430180. eng.

159. Kim HA, Kim MG. A Survey Study on Rheumatologist Consultation Time in Korean Hospitals. J Rheum Dis. 2020;27(1):45-50. Epub 2020/01/01. eng.

160. Kim JW, Friedman J, Clark S, Hafeez B, Listman D, Lame M, et al. Implementation of a Pediatric Emergency Telemedicine Program. Pediatr Emerg Care. 2020 Feb;36(2):e104-e7. PubMed PMID: 31929395. eng.

161. Kleven C, Postolski J, Hein B, Brown B. Reducing 30-Day All-Cause Rehospitalizations: A Pharmacist-Led Telehealth Service. Sr Care Pharm. 2020 Jul 1;35(7):324-30. PubMed PMID: 32600511. eng.

162. Ko LN, Kroshinsky D. Dermatology hospitalists: a multicenter survey study characterizing the infrastructure of consultative dermatology in select American hospitals. International Journal of Dermatology. 2018 May;57(5):553-8. PubMed PMID: WOS:000429546200009.

163. Kohlert S, Murphy P, Tse D, Liddy C, Afkham A, Keely E. Improving access to otolaryngology-head and neck surgery expert advice through eConsultations. Laryngoscope. 2018 Feb;128(2):350-5. PubMed PMID: 28573644. Epub 20170602. eng.

164. Korn RE, Wagle Shukla A, Katz M, Keenan HT, Goldenthal S, Auinger P, et al. Virtual visits for Parkinson disease: A multicenter noncontrolled cohort. Neurol Clin Pract. 2017 Aug;7(4):283-95. PubMed PMID: 28840919. PMCID: PMC5566796. eng.

165. Kortlever JTP, Ottenhoff JSE, Vagner GA, Ring D, Reichel LM. Visit Duration Does Not Correlate with Perceived Physician Empathy. J Bone Joint Surg Am. 2019 Feb 20;101(4):296-301. PubMed PMID: 30801368. eng.

166. Kottak N, Tesser J, Leibowitz E, Rosenberg M, Parenti D, DeHoratius R. Ethnographic Observational Study of the Biologic Initiation Conversation Between Rheumatologists and Biologic-Naive Rheumatoid Arthritis Patients. Arthritis Care Res (Hoboken). 2018 Jul;70(7):997-1004. PubMed PMID: 29381835. PMCID: PMC6033042. Epub 20180528. eng.

167. Kruk ME, Gage AD, Mbaruku GM, Leslie HH. Content of care in 15,000 sick child consultations in nine lower‐income countries. Health services research. 2018;53(4):2084-98.

168. Landon BE, Bayram C, Harrison C. Primary Care Visits in the USA and Australia 2000-2016. J Gen Intern Med. 2023 Feb;38(3):675-82. PubMed PMID: 35879536. PMCID: PMC9971376. Epub 20220725. eng.

169. Laurenzi CA, Gordon S, Skeen S, Coetzee BJ, Bishop J, Chademana E, Tomlinson M. The home visit communication skills inventory: Piloting a tool to measure community health worker fidelity to training in rural South Africa. Res Nurs Health. 2020 Jan;43(1):122-33. PubMed PMID: 31793678. Epub 20191203. eng.

170. Leask CF, Tennant H. Evaluation of an unscheduled care model delivered by advanced nurse practitioners in a primary-care setting. J Res Nurs. 2019 Dec;24(8):696-709. PubMed PMID: 34394595. PMCID: PMC7932326. Epub 20190723. eng.

171. Lebanova H, Balkanski S, Naseva E, Getov IN. What does self-medication counseling in Bulgarian community pharmacies look like – a field study. Pharmacia. 2020;67(4):289-93.

172. Lee EW, Kim HS, Yoo BN, Lee EJ, Hyun Park J. Effect of a Primary Care-Based Chronic Disease Management Program for Hypertension Patients in South Korea. Iran J Public Health. 2022 Mar;51(3):624-33. PubMed PMID: 35865058. PMCID: PMC9276591. eng.

173. Lee YK, Chor YY, Tan MY, Ngio YC, Chew AW, Tiew HW, et al. Factors associated with level of shared decision making in Malaysian primary care consultations. Patient Educ Couns. 2020 May;103(5):1049-51. PubMed PMID: 31866195. Epub 20191210. eng.

174. Lehmann V, Smets EMA, de Jong M, de Vos FYF, Stouthard JM, Hillen MA. Patient-provider communication during second opinion consultations in oncology. Patient Educ Couns. 2021 Oct;104(10):2490-7. PubMed PMID: 33744055. Epub 20210309. eng.

175. Leone D, Borghi L, Del Negro S, Becattini C, Chelo E, Costa M, et al. Doctor-couple communication during assisted reproductive technology visits. Hum Reprod. 2018 May 1;33(5):877-86. PubMed PMID: 29635461. eng.

176. Li HL, Chan YC, Huang JX, Cheng SW. Pilot Study Using Telemedicine Video Consultation for Vascular Patients' Care During the COVID-19 Period. Ann Vasc Surg. 2020 Oct;68:76-82. PubMed PMID: 32562832. PMCID: PMC7836856. Epub 20200617. eng.

177. Lim I, Saffari SE, Neo S. A cross-sectional study of knowledge and practices in the management of patients with Parkinson's disease amongst public practice-based general practitioners and geriatricians. BMC Health Serv Res. 2022 Jan 20;22(1):91. PubMed PMID: 35057812. PMCID: PMC8780393. Epub 20220120. eng.

178. Lin WC, Goldstein IH, Hribar MR, Huang A, Chiang MF. Secondary Use of Electronic Health Record Data for Prediction of Outpatient Visit Length in Ophthalmology Clinics. AMIA Annu Symp Proc. 2018;2018:1387-94. PubMed PMID: 30815183. PMCID: PMC6371379. Epub 20181205. eng.

179. Lindberg BH, Gjelstad S, Foshaug M, Høye S. Antibiotic prescribing for acute respiratory tract infections in Norwegian primary care out-of-hours service. Scand J Prim Health Care. 2017 Jun;35(2):178-85. PubMed PMID: 28569649. PMCID: PMC5499318. Epub 20170601. eng.

180. Liu L, Duan S, Zhang Y, Wu Y, Zhang L. Initial Experience of the Synchronized, Real-Time, Interactive, Remote Transthoracic Echocardiogram Consultation System in Rural China: Longitudinal Observational Study. JMIR Med Inform. 2019 Jul 8;7(3):e14248. PubMed PMID: 31287062. PMCID: PMC6643765. Epub 20190708. eng.

181. Liu Y, Chen C, Jin G, Zhao Y, Chen L, Du J, Lu X. Reasons for encounter and health problems managed by general practitioners in the rural areas of Beijing, China: A cross-sectional study. PLoS One. 2017;12(12):e0190036. PubMed PMID: 29267362. PMCID: PMC5739459. Epub 20171221. eng.

182. Liu YM, Mathews K, Vardanian A, Bozkurt T, Schneider JC, Hefner J, et al. Urban Telemedicine: The Applicability of Teleburns in the Rehabilitative Phase. J Burn Care Res. 2017 Jan/Feb;38(1):e235-e9. PubMed PMID: 27294853. PMCID: PMC9968417. eng.

183. Livori AC, Bishop JL, Ping SE, Oqueli E, Aldrich R, Fitzpatrick AM, Kong DCM. Towards OPtimising Care of Regionally-Based Cardiac Patients With a Telehealth Cardiology Pharmacist Clinic (TOPCare Cardiology). Heart Lung Circ. 2021 Jul;30(7):1023-30. PubMed PMID: 33622616. Epub 20210222. eng.

184. Looi JCL, Allison S, Kisely SR, Pring W, Reay RE, Bastiampillai T. Greatly increased Victorian outpatient private psychiatric care during the COVID-19 pandemic: new MBS-telehealth-item and face-to-face psychiatrist office-based services from April-September 2020. Australas Psychiatry. 2021 Aug;29(4):423-9. PubMed PMID: 33847167. Epub 20210413. eng.

185. Lopez JJ, Svetanoff WJ, Rosen JM, Carrasco A, Rentea RM. Leveraging Collaboration in Pediatric Multidisciplinary Colorectal Care Using a Telehealth Platform. Am Surg. 2022 Sep;88(9):2320-6. PubMed PMID: 34105388. Epub 20210609. eng.

186. Lun R, Walker G, Daham Z, Ramsay T, Portela de Oliveira E, Kassab M, et al. Transition to virtual appointments for interventional neuroradiology due to the COVID-19 pandemic: a survey of satisfaction. J Neurointerv Surg. 2020 Dec;12(12):1153-6. PubMed PMID: 33028673. Epub 20201007. eng.

187. Lurquin B, Kellou N, Colin C, Letrilliart L. Comparison of rural and urban French GPs' activity: a cross-sectional study. Rural Remote Health. 2021 Sep;21(3):5865. PubMed PMID: 34469693. Epub 20210901. eng.

188. Ly DP. Racial and Ethnic Disparities in the Evaluation and Management of Pain in the Outpatient Setting, 2006-2015. Pain Med. 2019 Feb 1;20(2):223-32. PubMed PMID: 29688509. PMCID: PMC6374136. eng.

189. Mabuto T, Hansoti B, Kerrigan D, Mshweshwe-Pakela N, Kubeka G, Charalambous S, Hoffmann C. HIV testing services in healthcare facilities in South Africa: a missed opportunity. J Int AIDS Soc. 2019 Oct;22(10):e25367. PubMed PMID: 31599495. PMCID: PMC6785782. eng.

190. Mahmoud MA, Daboos M, Gouda S, Othman A, Abdelmaboud M, Hussein ME, Akl M. Telemedicine (virtual clinic) effectively delivers the required healthcare service for pediatric ambulatory surgical patients during the current era of COVID-19 pandemic: A mixed descriptive study. J Pediatr Surg. 2022 Apr;57(4):630-6. PubMed PMID: 34953564. PMCID: PMC8626150. Epub 20211127. eng.

191. Makhlouf AM, Mohamed Ibrahim MI, Awaisu A, Vyas SK, Yusuff KB. Determinants of community pharmacists' information gathering and counseling practices during the management of minor ailments. Saudi Pharm J. 2021 Sep;29(9):992-8. PubMed PMID: 34588845. PMCID: PMC8463464. Epub 20210721. eng.

192. Mamo DB, Alemu BK. Rational Drug-Use Evaluation Based on World Health Organization Core Drug-Use Indicators in a Tertiary Referral Hospital, Northeast Ethiopia: A Cross-Sectional Study. Drug Healthc Patient Saf. 2020;12:15-21. PubMed PMID: 32021478. PMCID: PMC6970620. Epub 20200116. eng.

193. Mandavia R, Lapa T, Smith M, Bhutta MF. A cross-sectional evaluation of the validity of a smartphone otoscopy device in screening for ear disease in Nepal. Clin Otolaryngol. 2018 Feb;43(1):31-8. PubMed PMID: 28485038. Epub 20170528. eng.

194. Martin R, Tram K, Le L, Simmons C. Financial performance and reimbursement of pharmacist-led chronic care management. Am J Health Syst Pharm. 2020 Nov 16;77(23):1973-9. PubMed PMID: 32995845. eng.

195. Martinez KA, Rood M, Jhangiani N, Kou L, Rose S, Boissy A, Rothberg MB. Patterns of Use and Correlates of Patient Satisfaction with a Large Nationwide Direct to Consumer Telemedicine Service. J Gen Intern Med. 2018 Oct;33(10):1768-73. PubMed PMID: 30112737. PMCID: PMC6153236. Epub 20180815. eng.

196. Martinez KA, Rothberg MB. Physician Gender and Its Association With Patient Satisfaction and Visit Length: An Observational Study in Telemedicine. Cureus. 2022 Sep;14(9):e29158. PubMed PMID: 36258932. PMCID: PMC9572933. Epub 20220914. eng.

197. Marty S, Jaeger M, Moetteli S, Theodoridou A, Seifritz E, Hotzy F. Characteristics of Psychiatric Emergency Situations and the Decision-Making Process Leading to Involuntary Admission. Front Psychiatry. 2018;9:760. PubMed PMID: 30713511. PMCID: PMC6345710. Epub 20190118. eng.

198. Mathijssen EGE, Vriezekolk JE, Popa CD, van den Bemt BJF. Shared decision making in routine clinical care of patients with rheumatoid arthritis: an assessment of audio-recorded consultations. Ann Rheum Dis. 2020 Feb;79(2):170-5. PubMed PMID: 31662320. Epub 20191029. eng.

199. Matsushita A, Haruta J, Tsutumi M, Sato T, Maeno T. Validity of medical history taken by pharmacists using a medical history taking tool. J Gen Fam Med. 2017 Dec;18(6):403-8. PubMed PMID: 29264072. PMCID: PMC5729314. Epub 20170630. eng.

200. Matta GY, Khoong EC, Lyles CR, Schillinger D, Ratanawongsa N. Finding Meaning in Medication Reconciliation Using Electronic Health Records: Qualitative Analysis in Safety Net Primary and Specialty Care. JMIR Med Inform. 2018 May 7;6(2):e10167. PubMed PMID: 29735477. PMCID: PMC5962827. Epub 20180507. eng.

201. McBain RK, Jerome G, Leandre F, Browning M, Warsh J, Shah M, et al. Activity-based costing of health-care delivery, Haiti. Bull World Health Organ. 2018 Jan 1;96(1):10-7. PubMed PMID: 29403096. PMCID: PMC5791872. Epub 20171116. eng.

202. McCabe R, Pavlickova H, Xanthopoulou P, Bass NJ, Livingston G, Dooley J. Patient and companion shared decision making and satisfaction with decisions about starting cholinesterase medication at dementia diagnosis. Age Ageing. 2019 Sep 1;48(5):711-8. PubMed PMID: 31081513. eng.

203. McGloin H, O'Connell D, Glacken M, Mc Sharry P, Healy D, Winters-O'Donnell L, et al. Patient Empowerment Using Electronic Telemonitoring With Telephone Support in the Transition to Insulin Therapy in Adults With Type 2 Diabetes: Observational, Pre-Post, Mixed Methods Study. J Med Internet Res. 2020 May 14;22(5):e16161. PubMed PMID: 32406854. PMCID: PMC7256748. Epub 20200514. eng.

204. McKenzie KJ, Pierce D, Mercer SW, Gunn JM. Do GPs use motivational interviewing skills in routine consultations with patients living with mental-physical multimorbidity? An observational study of primary care in Scotland. Chronic Illn. 2021 Mar;17(1):29-40. PubMed PMID: 30580557. Epub 20181222. eng.

205. McKirdy A, Imbuldeniya AM. The clinical and cost effectiveness of a virtual fracture clinic service: An interrupted time series analysis and before-and-after comparison. Bone Joint Res. 2017 May;6(5):259-69. PubMed PMID: 28473333. PMCID: PMC5457647. eng.

206. Mehra P. Does Consultation Time Influence Patient Perception of Provider Communication Style, Patient Satisfaction, and Word of Mouth Recommendation in India? A mediation analysis. Asia Pacific Journal of Health Management. 2018.

207. Meijers MC, Noordman J, Spreeuwenberg P, Olde Hartman TC, van Dulmen S. Shared decision-making in general practice: an observational study comparing 2007 with 2015. Fam Pract. 2019 May 23;36(3):357-64. PubMed PMID: 30169562. eng.

208. Meijers MC, Potappel A, Kloek C, Olde Hartman T, Spreeuwenberg P, van Dulmen S, Noordman J. Shifts in patients' question-asking behaviour between 2007 and 2016: An observational study of video-recorded general practice consultations. Patient Educ Couns. 2020 Jun;103(6):1168-75. PubMed PMID: 31983476. Epub 20200123. eng.

209. Meleis LA, Patel MP, DeCoske M, Moorman M, Bush PW, Barbour S. Evaluation of the Role and Impact of Ambulatory Clinical Pharmacists in an Academic Comprehensive Cancer Center. J Adv Pract Oncol. 2020 Nov-Dec;11(8):817-24. PubMed PMID: 33489422. PMCID: PMC7810271. Epub 20201101. eng.

210. Melian C, Frampton C, Wyatt MC, Kieser D. Teleconsultation in the Management of Elective Orthopedic and Spinal Conditions During the COVID-19 Pandemic: Prospective Cohort Study of Patient Experiences. JMIR Form Res. 2021 Jun 15;5(6):e28140. PubMed PMID: 34048355. PMCID: PMC8208469. Epub 20210615. eng.

211. Mensa M, Tadesse T, Ayele A. Assessment of Drug Use Pattern by Using WHO Core Drug Use Indicators at Public Hospitals in Ethiopia. Journal of community medicine & health education. 2017;7:1-10.

212. Mercer SW, Zhou Y, Humphris GM, McConnachie A, Bakhshi A, Bikker A, et al. Multimorbidity and Socioeconomic Deprivation in Primary Care Consultations. Ann Fam Med. 2018 Mar;16(2):127-31. PubMed PMID: 29531103. PMCID: PMC5847350. eng.

213. Michael GC, Grema BA, Aliyu I, Usman MB, Umar ZA. Outpatient prevention counseling for malaria in northwest Nigeria: A single-centre descriptive cross-sectional study. Asian Pacific Journal of Tropical Medicine. 2020;13(5):214-20. PubMed PMID: 01542552-202013050-00006.

214. Michel P, Brami J, Chanelière M, Kret M, Mosnier A, Dupie I, et al. Patient safety incidents are common in primary care: A national prospective active incident reporting survey. PLoS One. 2017;12(2):e0165455. PubMed PMID: 28196076. PMCID: PMC5308773. Epub 20170214. eng.

215. Milford J, Strasser MR, Sinsky CA. TEAM approach reduced wait time, improved "face" time. J Fam Pract. 2018 Aug;67(8):E1-e8. PubMed PMID: 30110501. eng.

216. Mohamed Ibrahim MI, Awaisu A, Palaian S, Radoui A, Atwa H. Do community pharmacists in Qatar manage acute respiratory conditions rationally? A simulated client study. Journal of Pharmaceutical Health Services Research. 2018;9(1):33-9.

217. Mohamoud G, Mash R. Communication skills of general practitioners in Nairobi, Kenya: a descriptive observational study. BJGP Open. 2022 Sep;6(3). PubMed PMID: 35545265. PMCID: PMC9680749. Epub 20220928. eng.

218. Mugada V, Kolakota RK, Rasheed A, Kamireddi SS. Assessment of rational use of drugs using World Health Organization prescribing and patient care indicators in a tertiary care hospital. J Res Pharm. 2020;24(3):361-7.

219. Murphy AL, Gardner DM, Jacobs LM. Patient care activities by community pharmacists in a capitation funding model mental health and addictions program. BMC Psychiatry. 2018 Jun 14;18(1):192. PubMed PMID: 29898682. PMCID: PMC6000927. Epub 20180614. eng.

220. Murren-Boezem J, Solo-Josephson P, Zettler-Greeley CM. A Pediatric Telemedicine Response to a Natural Disaster. Telemed J E Health. 2020 Jun;26(6):720-4. PubMed PMID: 31549909. Epub 20190924. eng.

221. Musser JA, Cho J, Cohn A, Niziol LM, Ballouz D, Burke DT, Newman-Casey PA. Measuring impact of a quality improvement initiative on glaucoma clinic flow using an automated real-time locating system. BMC Ophthalmol. 2022 Jun 28;22(1):283. PubMed PMID: 35764976. PMCID: PMC9238160. Epub 20220628. eng.

222. Mustafa SS, Staicu ML, Yang L, Baumeister T, Vadamalai K, Ramsey A. Inpatient Electronic Consultations (E-consults) in Allergy/Immunology. J Allergy Clin Immunol Pract. 2020 Oct;8(9):2968-73. PubMed PMID: 32585408. PMCID: PMC7307996. Epub 20200622. eng.

223. Mutemwa R, Mayhew SH, Warren CE, Abuya T, Ndwiga C, Kivunaga J. Does service integration improve technical quality of care in low-resource settings? An evaluation of a model integrating HIV care into family planning services in Kenya. Health Policy Plan. 2017 Nov 1;32(suppl_4):iv91-iv101. PubMed PMID: 29194543. PMCID: PMC5886058. eng.

224. Nagykaldi ZJ, Dave A, Kristof CJ, Watts TN, Utpala S, Wickersham E. Improving Patient-Clinician Conversations During Annual Wellness Visits. J Am Board Fam Med. 2017 Mar-Apr;30(2):161-9. PubMed PMID: 28379822. PMCID: PMC5444902. eng.

225. Nair PP, Aghoram R, Thomas B, Bharadwaj B, Chinnakali P. Video teleconsultation services for persons with epilepsy during COVID-19 pandemic: An exploratory study from public tertiary care hospital in Southern India on feasibility, satisfaction, and effectiveness. Epilepsy Behav. 2021 Apr;117:107863. PubMed PMID: 33677363. PMCID: PMC9760560. Epub 20210304. eng.

226. Nakano Y, Hishikawa N, Sakamoto K, Myoraku Y, Ozaki Y, Takemoto M, et al. A unique telephone support system for dementia patients and their caregivers managed in Japan (Okayama Dementia Call Center, ODCC). Neurology and Clinical Neuroscience. 2018;6(4):100-3.

227. Nathan TA, Cohen AD, Vinker S. A new marker of primary care utilization - annual accumulated duration of time of visits. Isr J Health Policy Res. 2017 Aug 10;6(1):35. PubMed PMID: 28793928. PMCID: PMC5550929. Epub 20170810. eng.

228. Neke N, Reifferscheid A, Buchberger B, Wasem J. Time and cost associated with utilization of services at mobile health clinics among pregnant women. BMC Health Serv Res. 2018 Dec 3;18(1):920. PubMed PMID: 30509269. PMCID: PMC6276179. Epub 20181203. eng.

229. Newell CF, Nelson LD. Over-the-Counter Medication Prescribing in a Pediatric Emergency Department: Health Records Review. J Emerg Nurs. 2022 Jan;48(1):94-101.e1. PubMed PMID: 34656362. Epub 20211013. eng.

230. Newman-Casey PA, Musser JA, Niziol LM, Heisler MM, Kamat SS, Shah MM, et al. Integrating Patient Education Into the Glaucoma Clinical Encounter: A Lean Analysis. J Glaucoma. 2019 May;28(5):415-22. PubMed PMID: 30640805. PMCID: PMC6499667. eng.

231. Gholamzadeh Nikjoo R, Sokhanvar M, Motahari K, Partovi Y, Khodayari MT. Comparison of Visit Length and Waiting Time of Patients in Public and Private Clinics in the North of Iran. Health Scope. 2021;10(4):e112698. Epub 2021-10-01.

232. Nyabuti AO, Okalebo FA, Guantai EM. Examination of WHO/INRUD Core Drug Use Indicators at Public Primary Healthcare Centers in Kisii County, Kenya. Adv Pharmacol Pharm Sci. 2020;2020:3173847. PubMed PMID: 32647831. PMCID: PMC7321503. Epub 20200619. eng.

233. Ourth H, Nelson J, Spoutz P, Morreale AP. Development of a Pharmacoeconomic Model to Demonstrate the Effect of Clinical Pharmacist Involvement in Diabetes Management. J Manag Care Spec Pharm. 2018 May;24(5):449-57. PubMed PMID: 29694293. PMCID: PMC10398278. eng.

234. Pappalardo E, Magin P, Tapley A, Davey A, Holliday EG, Ball J, et al. General practice registrars' experiences of antenatal care: A cross-sectional analysis. Aust N Z J Obstet Gynaecol. 2020 Apr;60(2):188-95. PubMed PMID: 31424574. Epub 20190819. eng.

235. Parikh NR, Chang EM, Kishan AU, Kaprealian TB, Steinberg ML, Raldow AC. Time-Driven Activity-Based Costing Analysis of Telemedicine Services in Radiation Oncology. Int J Radiat Oncol Biol Phys. 2020 Oct 1;108(2):430-4. PubMed PMID: 32890526. PMCID: PMC7462887. eng.

236. Patel I, Chapman T, Camacho F, Shrestha S, Chang J, Balkrishnan R, Feldman SR. Satisfied patients and pediatricians: a cross-sectional analysis. Patient Relat Outcome Meas. 2018;9:299-307. PubMed PMID: 30214333. PMCID: PMC6118272. Epub 20180828. eng.

237. Paudel S, Bahadur G.C. K, Subedi M, Arjyal A, Daha SK, Lamichhane B, Sharma PK. Communication Skills of Physicians during Consultation in Out-Patient Settings at a Tertiary Hospital in Nepal. World Family Medicine. 2019;17(7):28-35.

238. Peek K, Carey M, Mackenzie L, Sanson-Fisher R. An observational study of Australian private practice physiotherapy consultations to explore the prescription of self-management strategies. Musculoskeletal Care. 2017 Dec;15(4):356-63. PubMed PMID: 28156062. Epub 20170203. eng.

239. Peltzer K, Pengpid S. Traditional Health Practitioners in Indonesia: Their Profile, Practice and Treatment Characteristics. Complement Med Res. 2019;26(2):93-100. PubMed PMID: 30572336. Epub 20181215. eng.

240. Perdoncini NN, Schussel JL, Amenábar JM, Torres-Pereira CC. Use of smartphone video calls in the diagnosis of oral lesions: Teleconsultations between a specialist and patients assisted by a general dentist. J Am Dent Assoc. 2021 Feb;152(2):127-35. PubMed PMID: 33494867. eng.

241. Peters LJ, Stubenrouch FE, Thijs JB, Klemm PL, Balm R, Ubbink DT. Predictors of the Level of Shared Decision Making in Vascular Surgery: A Cross Sectional Study. Eur J Vasc Endovasc Surg. 2022 Jul;64(1):65-72. PubMed PMID: 35537640. Epub 20220507. eng.

242. Pettersson ME, Öhlén J, Friberg F, Hydén LC, Wallengren C, Sarenmalm EK, Carlsson E. Prepared for surgery - Communication in nurses' preoperative consultations with patients undergoing surgery for colorectal cancer after a person-centred intervention. J Clin Nurs. 2018 Jul;27(13-14):2904-16. PubMed PMID: 29446494. Epub 20180530. eng.

243. Peurois M, Chopin M, Texier-Legendre G, Angoulvant C, Bellanger W, Bègue C, Ramond-Roquin A. To which non-physician health professionals do French general practitioners refer their patients to and what factors are associated with these referrals? Secondary analysis of the French national cross-sectional ECOGEN study. BMC Health Serv Res. 2022 Jan 5;22(1):25. PubMed PMID: 34983505. PMCID: PMC8729109. Epub 20220105. eng.

244. Phadke NA, Wolfson AR, Mancini C, Fu X, Goldstein SA, Ngo J, et al. Electronic Consultations in Allergy/Immunology. J Allergy Clin Immunol Pract. 2019 Nov-Dec;7(8):2594-602. PubMed PMID: 31170540. Epub 20190603. eng.

245. Phommachanh S, Essink DR, Wright EP, Broerse JEW, Mayxay M. Do health care providers give sufficient information and good counseling during ante-natal care in Lao PDR?: an observational study. BMC Health Serv Res. 2019 Jul 4;19(1):449. PubMed PMID: 31272432. PMCID: PMC6611023. Epub 20190704. eng.

246. Pierse T, Barry L, Glynn L, Quinlan D, Murphy A, O'Neill C. A pilot study of the duration of GP consultations in Ireland. Pilot Feasibility Stud. 2019;5:142. PubMed PMID: 31819804. PMCID: PMC6886172. Epub 20191201. eng.

247. Pilote L, Côté L, Chipenda Dansokho S, Brouillard É, Giguère AMC, Légaré F, et al. Talking about treatment benefits, harms, and what matters to patients in radiation oncology: an observational study. BMC Med Inform Decis Mak. 2019 Apr 11;19(1):84. PubMed PMID: 30975132. PMCID: PMC6460774. Epub 20190411. eng.

248. Pintova S, Leibrandt R, Smith CB, Adelson KB, Gonsky J, Egorova N, et al. Conducting Goals-of-Care Discussions Takes Less Time Than Imagined. JCO Oncol Pract. 2020 Dec;16(12):e1499-e506. PubMed PMID: 32749930. Epub 20200804. eng.

249. Pochert M, Voigt K, Bortz M, Sattler A, Schübel J, Bergmann A. The workload for home visits by German family practitioners: an analysis of regional variation in a cross-sectional study. BMC Fam Pract. 2019 Jan 4;20(1):3. PubMed PMID: 30609917. PMCID: PMC6318834. Epub 20190104. eng.

250. Polat JK, Hughes EL, Brown EN, Conner IP. Teleglaucoma Initiative at a Veterans Affairs Hospital: Pilot Safety Data and Early Experience. Ophthalmol Glaucoma. 2021 Nov-Dec;4(6):632-7. PubMed PMID: 33839331. Epub 20210409. eng.

251. Popescu C, Golden G, Benrimoh D, Tanguay-Sela M, Slowey D, Lundrigan E, et al. Evaluating the Clinical Feasibility of an Artificial Intelligence-Powered, Web-Based Clinical Decision Support System for the Treatment of Depression in Adults: Longitudinal Feasibility Study. JMIR Form Res. 2021 Oct 25;5(10):e31862. PubMed PMID: 34694234. PMCID: PMC8576598. Epub 20211025. eng.

252. Pradhan R, Peeters W, Boutong S, Mitchell C, Patel R, Faroug R, Roussot M. Virtual phone clinics in orthopaedics: evaluation of clinical application and sustainability. BMJ Open Qual. 2021 Oct;10(4). PubMed PMID: 34645613. PMCID: PMC8515471. eng.

253. Puchner R, Schaffer G, Sautner J, Alkin A, Machold K, Stamm T. Rheumatological care in rural areas : The Rheuma-Bus project 2018. Wien Klin Wochenschr. 2021 Feb;133(3-4):131-6. PubMed PMID: 32189120. Epub 20200318. eng.

254. Qiao T, Fan Y, Geater AF, Chongsuvivatwong V, McNeil EB. Factors associated with the doctor-patient relationship: doctor and patient perspectives in hospital outpatient clinics of Inner Mongolia Autonomous Region, China. Patient Prefer Adherence. 2019;13:1125-43. PubMed PMID: 31409976. PMCID: PMC6643492. Epub 20190716. eng.

255. Rahhal R, Goad L, Bishop W. Impact of a Medical Scribe Program on Outpatient Pediatric Gastroenterology Clinic. J Pediatr Gastroenterol Nutr. 2021 Feb 1;72(2):220-5. PubMed PMID: 32969957. eng.

256. Rainey R, Theiss L, Lopez E, Wood T, Wood L, Marques I, et al. Characterizing the impact of verbal communication and health literacy in the patient-surgeon encounter. Am J Surg. 2022 Sep;224(3):943-8. PubMed PMID: 35527045. Epub 20220430. eng.

257. Rajput AH, Khan A, Aqeel A, Muneeb M. PHYSICIAN EMPATHY LEVELS AT LIAQUAT UNIVERSITY HOSPITAL, HYDERABAD. INDO AMERICAN JOURNAL OF PHARMACEUTICAL SCIENCES. 2018;5(6):5101-6.

258. Rao A, Shi Z, Ray KN, Mehrotra A, Ganguli I. National Trends in Primary Care Visit Use and Practice Capabilities, 2008-2015. Ann Fam Med. 2019 Nov;17(6):538-44. PubMed PMID: 31712292. PMCID: PMC6846275. eng.

259. Ratwani RM, Brennan D, Sheahan W, Fong A, Adams K, Gordon A, et al. A descriptive analysis of an on-demand telehealth approach for remote COVID-19 patient screening. J Telemed Telecare. 2022 Aug;28(7):494-7. PubMed PMID: 32698650. PMCID: PMC9272041. Epub 20200723. eng.

260. Read-Brown S, Hribar MR, Reznick LG, Lombardi LH, Parikh M, Chamberlain WD, et al. Time Requirements for Electronic Health Record Use in an Academic Ophthalmology Center. JAMA Ophthalmol. 2017 Nov 1;135(11):1250-7. PubMed PMID: 29049512. PMCID: PMC5710390. eng.

261. Reblin M, Clayton MF, Xu J, Hulett JM, Latimer S, Donaldson GW, Ellington L. Caregiver, patient, and nurse visit communication patterns in cancer home hospice. Psychooncology. 2017 Dec;26(12):2285-93. PubMed PMID: 28029712. PMCID: PMC5489378. Epub 20170210. eng.

262. Rej A, Buckle RL, Shaw CC, Trott N, Urwin H, McGough N, et al. National survey evaluating the provision of gastroenterology dietetic services in England. Frontline Gastroenterol. 2021;12(5):380-4. PubMed PMID: 35401953. PMCID: PMC8989012. Epub 20200616. eng.

263. Riley AR, Paternostro JK, Walker BL, Wagner DV. The impact of behavioral health consultations on medical encounter duration in pediatric primary care: A retrospective match-controlled study. Fam Syst Health. 2019 Jun;37(2):162-6. PubMed PMID: 31058527. PMCID: PMC6557675. Epub 20190506. eng.

264. Roll A, Pattison D, Baumgartner R, Sublett L, Brown B. The design and evaluation of a pilot covisit model: Integration of a pharmacist into a primary care team. J Am Pharm Assoc (2003). 2020 May-Jun;60(3):491-6. PubMed PMID: 31889652. Epub 20191227. eng.

265. Rutten G, van Vugt HA, de Weerdt I, de Koning E. Implementation of a Structured Diabetes Consultation Model to Facilitate a Person-Centered Approach: Results From a Nationwide Dutch Study. Diabetes Care. 2018 Apr;41(4):688-95. PubMed PMID: 29363538. Epub 20180123. eng.

266. Ryan M, Carrington C, Ward EC, Burns CL, Cuff K, Mackinnon M, Snoswell CL. Changing from telephone to videoconference for pre-treatment pharmacist consults in cancer services: Impacts to funding and time efficiency. J Telemed Telecare. 2021 Dec;27(10):680-4. PubMed PMID: 34726996. eng.

267. Ryu WHA, Kerolus MG, Traynelis VC. Clinicians' User Experience of Telemedicine in Neurosurgery During COVID-19. World Neurosurg. 2021 Feb;146:e359-e67. PubMed PMID: 33203534. PMCID: PMC9760258. Epub 20201024. eng.

268. Rzadkiewicz M, Haugan G, Włodarczyk D. Mature Adults at the GP: Length of Visit and Patient Satisfaction-Associations with Patient, Doctor, and Facility Characteristics. Medicina (Kaunas). 2022 Jan 20;58(2). PubMed PMID: 35208483. PMCID: PMC8874721. Epub 20220120. eng.

269. Abbas S, Denholm J, Kermode M, Xiaoguang Y, Kane S. Receiving healthcare for drug-resistant TB: a cross-sectional survey from Pakistan. Public Health Action. 2021 Sep 21;11(3):114-9. PubMed PMID: 34567986. PMCID: PMC8455020. eng.

270. Saginela S, Madri A, Desu R, Bola V. Assessment of Satisfaction Levels of the Outpatients Attending Outpatient Departments in a Quaternary Care Hospital. Int J Res Foundation Hosp Healthc Adm. 2019;7(2):91-8.

271. Salisbury C, Lay-Flurrie S, Bankhead CR, Fuller A, Murphy M, Caddick B, et al. Measuring the complexity of general practice consultations: a Delphi and cross-sectional study in English primary care. Br J Gen Pract. 2021 Jun;71(707):e423-e31. PubMed PMID: 33824162. PMCID: PMC8049201. Epub 20210527. eng.

272. Sanders-Pinheiro H, Colugnati FAB, Denhaerynck K, Marsicano EO, Medina JOP, De Geest S. Multilevel Correlates of Immunosuppressive Nonadherence in Kidney Transplant Patients: The Multicenter ADHERE BRAZIL Study. Transplantation. 2021 Jan 1;105(1):255-66. PubMed PMID: 32150041. eng.

273. Cairns C, Kang K. National hospital ambulatory medical care survey: 2019 emergency department summary tables. Centre for Disease Control and Prevention; 2022.

274. Santo L, Okeyode T, Schappert S. National Ambulatory Medical Care Survey–Community Health Centers: 2020 National Summary Tables. Centre for Disease Control and Prevention; 2022.

275. Saunders JE, Bessen S, Magro I, Cowan D, Quiroz MG, Mojica-Alvarez K, et al. Community health workers and mHealth systems for hearing screening in rural Nicaraguan schoolchildren. J Glob Health. 2022 Aug 9;12:04060. PubMed PMID: 35938885. PMCID: PMC9359107. Epub 20220809. eng.

276. Schäfer WLA, van den Berg MJ, Groenewegen PP. The association between the workload of general practitioners and patient experiences with care: results of a cross-sectional study in 33 countries. Hum Resour Health. 2020 Oct 16;18(1):76. PubMed PMID: 33066776. PMCID: PMC7565810. Epub 20201016. eng.

277. Schifeling CH, Shanbhag P, Johnson A, Atwater RC, Koljack C, Parnes BL, et al. Disparities in Video and Telephone Visits Among Older Adults During the COVID-19 Pandemic: Cross-Sectional Analysis. JMIR Aging. 2020 Nov 10;3(2):e23176. PubMed PMID: 33048821. PMCID: PMC7674139. Epub 20201110. eng.

278. Schimmelfing JT, Brookhart AL, Fountain KMB, Goode JKR. Pharmacist intervention in patient selection of nonprescription and self-care products. J Am Pharm Assoc (2003). 2017 Jan-Feb;57(1):86-9.e1. PubMed PMID: 27843108. Epub 20161111. eng.

279. Schnakenberg R, Radbruch L, Kersting C, Frank F, Wilm S, Becka D, et al. More counselling for end-of-life decisions by GPs with own advance directives: A postal survey among German general practitioners. Eur J Gen Pract. 2018 Dec;24(1):131-7. PubMed PMID: 29547013. PMCID: PMC5917308. eng.

280. Sema FD, Asres ED, Wubeshet BD. Evaluation of Rational Use of Medicine Using WHO/INRUD Core Drug Use Indicators at Teda and Azezo Health Centers, Gondar Town, Northwest Ethiopia. Integr Pharm Res Pract. 2021;10:51-63. PubMed PMID: 34189113. PMCID: PMC8232866. Epub 20210621. eng.

281. Sfar-Gandoura H, Ryan GS, Melvin G. Evaluation of a drop-in clinic for young people with attention deficit hyperactivity disorder. Nurs Child Young People. 2017 Jun 12;29(5):24-32. PubMed PMID: 28604214. eng.

282. Shalihin S, Firzada I, Din M. Assessment of Diabetic Patient Waiting Time in A Primary Healthcare Clinic. IIUM Medical Journal Malaysia. 2020 07/01;19(2).

283. Shapiro DJ, King LM, Tsay SV, Hicks LA, Hersh AL. Association between antibiotic prescribing and visit duration among patients with respiratory tract infections. Infect Control Hosp Epidemiol. 2022 Sep;43(9):1238-41. PubMed PMID: 34112273. PMCID: PMC8900143. Epub 20210611. eng.

284. Shapiro WL, Yu EL, Arin JC, Murray KF, Ali S, Desai NK, et al. Clinical Practice Approach to Nonalcoholic Fatty Liver Disease by Pediatric Gastroenterologists in the United States. J Pediatr Gastroenterol Nutr. 2019 Feb;68(2):182-9. PubMed PMID: 30640271. PMCID: PMC8053385. eng.

285. Shen J, Zhang J, He Q, Pan H, Wu Z, Nie L, et al. "Without the need for a second visit" initiative improves patient satisfaction with updated services of outpatient clinics in China. BMC Health Serv Res. 2021 Mar 23;21(1):267. PubMed PMID: 33757490. PMCID: PMC7986498. Epub 20210323. eng.

286. Shim JY, Kaur R, Laufer MR, Grimstad FW. The Use of Telemedicine in Pediatric and Adolescent Gynecology. J Pediatr Adolesc Gynecol. 2022 Apr;35(2):133-7. PubMed PMID: 34619357. Epub 20211004. eng.

287. Shuaib W, Hilmi J, Caballero J, Rashid I, Stanazai H, Ajanovic A, et al. Impact of a scribe program on patient throughput, physician productivity, and patient satisfaction in a community-based emergency department. Health Informatics J. 2021 Jan-Mar;27(1):1460458217692930. PubMed PMID: 29239230. Epub 20170301. eng.

288. Siele SM, Abdu N, Ghebrehiwet M, Hamed MR, Tesfamariam EH. Drug prescribing and dispensing practices in regional and national referral hospitals of Eritrea: Evaluation with WHO/INRUD core drug use indicators. PLoS One. 2022;17(8):e0272936. PubMed PMID: 35984825. PMCID: PMC9390936. Epub 20220819. eng.

289. Silver SL, Lewis MN, Ledford CJW. A Stepwise Transition to Telemedicine in Response to COVID-19. J Am Board Fam Med. 2021 Feb;34(Suppl):S152-s61. PubMed PMID: 33622831. eng.

290. Singh T, Banerjee B, Garg S, Sharma S. A prescription audit using the World Health Organization-recommended core drug use indicators in a rural hospital of Delhi. J Educ Health Promot. 2019;8:37. PubMed PMID: 30993130. PMCID: PMC6432812. Epub 20190215. eng.

291. Sinha N, Cornell M, Wheatley B, Munley N, Seeley M. Looking Through a Different Lens: Patient Satisfaction With Telemedicine in Delivering Pediatric Fracture Care. J Am Acad Orthop Surg Glob Res Rev. 2019 Sep;3(9):e100. PubMed PMID: 31773080. PMCID: PMC6860133. Epub 20190923. eng.

292. Sisay M, Mengistu G, Molla B, Amare F, Gabriel T. Evaluation of rational drug use based on World Health Organization core drug use indicators in selected public hospitals of eastern Ethiopia: a cross sectional study. BMC Health Serv Res. 2017 Feb 23;17(1):161. PubMed PMID: 28231833. PMCID: PMC5324210. Epub 20170223. eng.

293. Skrepnek GH, Mills JL, Sr., Lavery LA, Armstrong DG. Health Care Service and Outcomes Among an Estimated 6.7 Million Ambulatory Care Diabetic Foot Cases in the U.S. Diabetes Care. 2017 Jul;40(7):936-42. PubMed PMID: 28495903. Epub 20170511. eng.

294. Smits M, Peters Y, Ranke S, Plat E, Laurant M, Giesen P. Substitution of general practitioners by nurse practitioners in out-of-hours primary care home visits: A quasi-experimental study. Int J Nurs Stud. 2020 Apr;104:103445. PubMed PMID: 32105972. Epub 20190928. eng.

295. Soegaard Ballester JM, Scott MF, Owei L, Neylan C, Hanson CW, Morris JB. Patient preference for time-saving telehealth postoperative visits after routine surgery in an urban setting. Surgery. 2018 Apr;163(4):672-9. PubMed PMID: 29398042. Epub 20180203. eng.

296. Søndergaard SR, Madsen PH, Hilberg O, Bechmann T, Jakobsen E, Jensen KM, et al. The impact of shared decision making on time consumption and clinical decisions. A prospective cohort study. Patient Educ Couns. 2021 Jul;104(7):1560-7. PubMed PMID: 33390303. Epub 20201223. eng.

297. Stark T, Shoag JE, Nicolas J, Patel N, Taylor B, Scherr DS. Ambulatory Bladder Cancer Care in the United States. Urol Pract. 2019 May;6(3):165-73. PubMed PMID: 37300105. Epub 20190423. eng.

298. Stevens S, Bankhead C, Mukhtar T, Perera-Salazar R, Holt TA, Salisbury C, Hobbs FDR. Patient-level and practice-level factors associated with consultation duration: a cross-sectional analysis of over one million consultations in English primary care. BMJ Open. 2017 Nov 16;7(11):e018261. PubMed PMID: 29150473. PMCID: PMC5701995. Epub 20171116. eng.

299. Stewart AE, Lovato JF, Zimmer R, Stewart AP, Hinely MT, Yang M. Development of a screening tool to identify patients likely to benefit from clinical pharmacist review in a home-based primary care population. J Am Pharm Assoc (2003). 2020 Sep-Oct;60(5):750-6. PubMed PMID: 32482500. PMCID: PMC8867744. Epub 20200529. eng.

300. Stewart C, Coffey-Sandoval J, Souverein EA, Ho TC, Lee TC, Nallasamy S. Patient and Provider Experience in Real-Time Telemedicine Consultations for Pediatric Ophthalmology. Clin Ophthalmol. 2022;16:2943-53. PubMed PMID: 36071727. PMCID: PMC9444028. Epub 20220901. eng.

301. Stewart E, Sun I, Kim C, Giddings A, Silverio F, Taruc O, Nica L. Examining Radiation Treatment Appointment Times at a Canadian Cancer Centre: A Timing Study. J Med Imaging Radiat Sci. 2019 Dec;50(4):536-42. PubMed PMID: 31629674. Epub 20191016. eng.

302. Stime KJ, Garrett N, Sookrajh Y, Dorward J, Dlamini N, Olowolagba A, et al. Clinic flow for STI, HIV, and TB patients in an urban infectious disease clinic offering point-of-care testing services in Durban, South Africa. BMC Health Serv Res. 2018 May 11;18(1):363. PubMed PMID: 29751798. PMCID: PMC5948731. Epub 20180511. eng.

303. Sumargono E, Anastasia M, Saleh I, Kholinne E. The Role of Virtual Clinics in Postoperative Total Knee Replacement Surgery Follow-Up during COVID-19 Pandemic. Adv Orthop. 2022;2022:9558511. PubMed PMID: 35756355. PMCID: PMC9225886. Epub 20220616. eng.

304. Swami M, Gravelle H, Scott A, Williams J. Hours worked by general practitioners and waiting times for primary care. Health Econ. 2018 Oct;27(10):1513-32. PubMed PMID: 29920838. Epub 20180619. eng.

305. Talyshinskii A, Guliev B, Komyakov B, Galfano A. Patient Counseling Through the Pelvicalyceal-shaped Labyrinth: In Search of an Easy Understanding of the Upcoming Stone Removal: A Pilot Study. Urology. 2020 Sep;143:75-9. PubMed PMID: 32473936. PMCID: PMC7263277. Epub 20200528. eng.

306. Tampi RP, Tembo T, Mukumba-Mwenechanya M, Sharma A, Dowdy DW, Holmes CB, et al. Operational characteristics of antiretroviral therapy clinics in Zambia: a time and motion analysis. BMC Health Serv Res. 2019 Apr 24;19(1):244. PubMed PMID: 31018846. PMCID: PMC6480736. Epub 20190424. eng.

307. Tassew SG, Abraha HN, Gidey K, Gebre AK. Assessment of drug use pattern using WHO core drug use indicators in selected general hospitals: a cross-sectional study in Tigray region, Ethiopia. BMJ Open. 2021 Oct 27;11(10):e045805. PubMed PMID: 34706944. PMCID: PMC8552154. Epub 20211027. eng.

308. Taylor SK, Andrzejowski JC, Wiles MD, Bland S, Jones GL, Radley SC. A prospective observational study of the impact of an electronic questionnaire (ePAQ-PO) on the duration of nurse-led pre-operative assessment and patient satisfaction. PLoS One. 2018;13(10):e0205439. PubMed PMID: 30339687. PMCID: PMC6195264. Epub 20181019. eng.

309. Teklemariam T. Drug Prescribing and Despensing Practice in Public Hospitals in Tigry Regional State, Ethiopia. Global Journal of Medical Research. 2018;18(4).

310. Tenforde AS, Borgstrom H, Polich G, Steere H, Davis IS, Cotton K, et al. Outpatient Physical, Occupational, and Speech Therapy Synchronous Telemedicine: A Survey Study of Patient Satisfaction with Virtual Visits During the COVID-19 Pandemic. Am J Phys Med Rehabil. 2020 Nov;99(11):977-81. PubMed PMID: 32804713. PMCID: PMC7526401. eng.

311. Thapa R, Saldanha S, Bucker N, Rishith P. AN ASSESSMENT OF PATIENT WAITING AND CONSULTATION TIME IN THE OUTPATIENT DEPARTMENT AT A SELECTED TERTIARY CARE TEACHING HOSPITAL. Journal of Evolution of Medical and Dental Sciences. 2018 2018/02/19/

//;7:984+. English.

312. Thotam SM, Buhse M. Patient Satisfaction with Physicians and Nurse Practitioners in Multiple Sclerosis Centers. Int J MS Care. 2020 May-Jun;22(3):129-35. PubMed PMID: 32607075. PMCID: PMC7307871. Epub 20191008. eng.

313. Tilburt J, Yost KJ, Lenz HJ, Zúñiga ML, O'Byrne T, Branda ME, et al. A Multicenter Comparison of Complementary and Alternative Medicine (CAM) Discussions in Oncology Care: The Role of Time, Patient-Centeredness, and Practice Context. Oncologist. 2019 Nov;24(11):e1180-e9. PubMed PMID: 31101701. PMCID: PMC6853106. Epub 20190517. eng.

314. Tilburt JC, T OB, Branda ME, Phelan S. Higher BMI associated with shorter visits in male oncology patients: An exploratory analysis. Patient Educ Couns. 2019 Dec;102(12):2353-7. PubMed PMID: 31331706. PMCID: PMC6851463. Epub 20190712. eng.

315. Timmer A, de Sordi D, Menke E, Peplies J, Claßen M, Koletzko S, Otto-Sobotka F. Modeling determinants of satisfaction with health care in youth with inflammatory bowel disease: a cross-sectional survey. Clin Epidemiol. 2018;10:1289-305. PubMed PMID: 30310323. PMCID: PMC6165738. Epub 20180925. eng.

316. Ting YY, Reid JL, Treloar E, Lee WSB, Tee JY, Cong WJP, et al. The doctor will see you now: eye gaze, conversation and patient engagement in the surgical outpatient clinic. An Australian observational cross-sectional study. ANZ J Surg. 2021 Nov;91(11):2376-81. PubMed PMID: 34427041. Epub 20210823. eng.

317. Tiruneh CT, Woldeyohannes FW. Antiretroviral Therapy Service Quality and Associated Factors at Selected Public Hospitals, Addis Ababa, Ethiopia, 2021. HIV AIDS (Auckl). 2022;14:129-42. PubMed PMID: 35370425. PMCID: PMC8964440. Epub 20220325. eng.

318. Tornero-Molina J, Sánchez-Alonso F, Fernández-Prada M, Bris-Ochaita ML, Sifuentes-Giraldo A, Vidal-Fuentes J. Tele-Rheumatology during the COVID-19 pandemic. Reumatol Clin (Engl Ed). 2022 Mar;18(3):157-63. PubMed PMID: 34088655. PMCID: PMC8169323. Epub 20210602. eng.

319. Tranter-Entwistle I, Best K, Ianev R, Beresford T, McCombie A, Laws P. Introduction and validation of a surgical ward round checklist to improve surgical ward round performance in a tertiary vascular service. ANZ J Surg. 2020 Jul;90(7-8):1358-63. PubMed PMID: 32356576. Epub 20200501. eng.

320. Tsvyakh AI, Hospodarskyy AJ. Telerehabilitation of Patients with Injuries of the Lower Extremities. Telemed J E Health. 2017 Dec;23(12):1011-5. PubMed PMID: 28525311. Epub 20170519. eng.

321. Van Dril E, Schumacher C, Kliethermes MA, Borchert JS, Buros Stein A. Workload evaluation of clinical pharmacists in the ambulatory care setting. JACCP: JOURNAL OF THE AMERICAN COLLEGE OF CLINICAL PHARMACY. 2020;3(6):1015-27.

322. van Veenendaal H, Voogdt-Pruis H, Ubbink DT, Hilders C. Effect of a multilevel implementation programme on shared decision-making in breast cancer care. BJS Open. 2021 Mar 5;5(2). PubMed PMID: 33688949. PMCID: PMC7944508. eng.

323. Vedanthan R, Lee DJ, Kamano JH, Herasme OI, Kiptoo P, Tulienge D, et al. Hypertension management in rural western Kenya: a needs-based health workforce estimation model. Hum Resour Health. 2019 Jul 16;17(1):57. PubMed PMID: 31311561. PMCID: PMC6636021. Epub 20190716. eng.

324. Vilendrer S, Lough ME, Garvert DW, Lambert MH, Lu JH, Patel B, et al. Nursing Workflow Change in a COVID-19 Inpatient Unit Following the Deployment of Inpatient Telehealth: Observational Study Using a Real-Time Locating System. J Med Internet Res. 2022 Jun 17;24(6):e36882. PubMed PMID: 35635840. PMCID: PMC9208574. Epub 20220617. eng.

325. Vogt EL, Welch BM, Bunnell BE, Barrera JF, Paige SR, Owens M, et al. Quantifying the Impact of COVID-19 on Telemedicine Utilization: Retrospective Observational Study. Interact J Med Res. 2022 Jan 28;11(1):e29880. PubMed PMID: 34751158. PMCID: PMC8797150. Epub 20220128. eng.

326. von dem Knesebeck O, Koens S, Marx G, Scherer M. Perceptions of time constraints among primary care physicians in Germany. BMC Fam Pract. 2019 Oct 22;20(1):142. PubMed PMID: 31640573. PMCID: PMC6805618. Epub 20191022. eng.

327. Vyas S, Murren-Boezem J, Solo-Josephson P. Analysis of a Pediatric Telemedicine Program. Telemed J E Health. 2018 Apr 24. PubMed PMID: 29688823. Epub 20180424. eng.

328. Waller A, Bryant J, Cameron E, Galal M, Symonds I, Sanson-Fisher R. Screening for recommended antenatal risk factors: How long does it take? Women Birth. 2018 Dec;31(6):489-95. PubMed PMID: 29366711. Epub 20180201. eng.

329. Walling AM, D'Ambruoso SF, Malin JL, Hurvitz S, Zisser A, Coscarelli A, et al. Effect and Efficiency of an Embedded Palliative Care Nurse Practitioner in an Oncology Clinic. J Oncol Pract. 2017 Sep;13(9):e792-e9. PubMed PMID: 28813191. Epub 20170816. eng.

330. Wang AB, Bacci JL, Amoo M, Ree C, Firebaugh R, Odegard P. Impact of an immunization platform in community pharmacies. J Am Pharm Assoc (2003). 2019 Jul-Aug;59(4s):S151-s5. PubMed PMID: 31255521. Epub 20190627. eng.

331. Wazaify M, Elayeh E, Tubeileh R, Hammad EA. Assessing insomnia management in community pharmacy setting in Jordan: A simulated patient approach. PLoS One. 2019;14(12):e0226076. PubMed PMID: 31834888. PMCID: PMC6910704. Epub 20191213. eng.

332. Wei Y, Wang F, Pan Z, Wang M, Jin G, Lu X. Physical examination performed by general practitioners in 5 community health service institutions in Beijing: an observational study. BMC Prim Care. 2022 Jan 14;23(1):7. PubMed PMID: 35172736. PMCID: PMC8759261. Epub 20220114. eng.

333. Weise J, Pollack A, Britt H, Trollor JN. Primary health care for people with an intellectual disability: an exploration of consultations, problems identified, and their management in Australia. J Intellect Disabil Res. 2017 May;61(5):399-410. PubMed PMID: 28116807. Epub 20170124. eng.

334. Weiss R, Vittinghoff E, Fang MC, Cimino JEW, Chasteen KA, Arnold RM, et al. Associations of Physician Empathy with Patient Anxiety and Ratings of Communication in Hospital Admission Encounters. J Hosp Med. 2017 Oct;12(10):805-10. PubMed PMID: 28991945. Epub 20170906. eng.

335. Wendie TF, Ahmed A, Mohammed SA. Drug use pattern using WHO core drug use indicators in public health centers of Dessie, North-East Ethiopia. BMC Med Inform Decis Mak. 2021 Jun 25;21(1):197. PubMed PMID: 34172067. PMCID: PMC8228957. Epub 20210625. eng.

336. Weyer SM, Cook ML, Riley L. The Direct Observation of Nurse Practitioner Care study: An overview of the NP/patient visit. J Am Assoc Nurse Pract. 2017 Jan;29(1):46-57. PubMed PMID: 27973706. Epub 20161214. eng.

337. Willems A, Tapley A, Fielding A, Tng V, Holliday EG, van Driel ML, et al. General Practice Registrars' Management of and Specialist Referral Patterns for Atopic Dermatitis. Dermatol Pract Concept. 2021 Jan;11(1):e2021118. PubMed PMID: 33614210. PMCID: PMC7875659. Epub 20210129. eng.

338. Willging AM, Castro E, Xu J. Physician-patient communication in vascular surgery: Analysis of encounters in academic practice. SAGE Open Med. 2022;10:20503121221122414. PubMed PMID: 36093425. PMCID: PMC9459473. Epub 20220906. eng.

339. Williams D, Edwards A, Wood F, Lloyd A, Brain K, Thomas N, et al. Ability of observer and self-report measures to capture shared decision-making in clinical practice in the UK: a mixed-methods study. BMJ Open. 2019 Aug 18;9(8):e029485. PubMed PMID: 31427333. PMCID: PMC6701565. Epub 20190818. eng.

340. Winkelman AJ, Beller HL, Morgan KE, Corbett ST, Leroy SV, Noona SW, et al. Benefits and barriers to pediatric tele-urology during the COVID-19 pandemic. J Pediatr Urol. 2020 Dec;16(6):840.e1-.e6. PubMed PMID: 33077389. PMCID: PMC7543732. Epub 20201008. eng.

341. Wogayehu B, Chisha Y, Tekabe B, Adinew A, Asefaw M. A cross sectional comparison of drug use indicators using WHO methodology in primary level hospitals participating in an Auditable Pharmaceutical Transactions and Services program versus non-APTS primary hospitals in Southern Ethiopia. PLoS One. 2019;14(10):e0223523. PubMed PMID: 31589641. PMCID: PMC6779258. Epub 20191007. eng.

342. Wong JJ, Hogg-Johnson S, Bussières AE, French SD, Mior SA. The association between chiropractors' view of practice and patient encounter-level characteristics in Ontario, Canada: a cross-sectional study. Chiropr Man Therap. 2021 Sep 28;29(1):41. PubMed PMID: 34583730. PMCID: PMC8477501. Epub 20210928. eng.

343. Wood AC, de Mitchell CAG, Kaushik R. Improvement in Total and Face-to-Face Provider Time in a Multidisciplinary Craniofacial Team Clinic: An Interventional Study. Cleft Palate Craniofac J. 2022 Jun;59(6):779-84. PubMed PMID: 34165000. Epub 20210624. eng.

344. Xie Z, Or C. Associations Between Waiting Times, Service Times, and Patient Satisfaction in an Endocrinology Outpatient Department: A Time Study and Questionnaire Survey. Inquiry. 2017 Jan-Dec;54:46958017739527. PubMed PMID: 29161947. PMCID: PMC5798665. eng.

345. Yilma Z, Mekonnen T, Siraj EA, Agmassie Z, Yehualaw A, Debasu Z, et al. Assessment of Prescription Completeness and Drug Use Pattern in Tibebe-Ghion Comprehensive Specialized Hospital, Bahir Dar, Ethiopia. Biomed Res Int. 2020;2020:8842515. PubMed PMID: 33134391. PMCID: PMC7593717. Epub 20201019. eng.

346. Young RA, Burge SK, Kumar KA, Wilson JM, Ortiz DF. A Time-Motion Study of Primary Care Physicians' Work in the Electronic Health Record Era. Fam Med. 2018 Feb;50(2):91-9. PubMed PMID: 29432623. eng.
[truncated: 17,660 more chars]
